# Integrative multi‐omics approach for mechanism of humidifier disinfectant‐associated lung injury

**DOI:** 10.1002/ctm2.562

**Published:** 2021-11-04

**Authors:** Jeong‐Hyun Kim, Kwoneel Kim, Jeonghun Yeom, Eun Lee, Mi‐Jin Kang, Seung‐Hwa Lee, Kyunggon Kim, So‐Yeon Lee, Sang‐Bum Hong, Dong Kyu Oh, Kyuhong Lee, Seong‐Jin Choi, Mi‐Jin Yang, Jiyeon Kim, Soo‐Jong Hong

**Affiliations:** ^1^ Department of Medicine University of Ulsan College of Medicine Seoul Republic of Korea; ^2^ Department of Biology Kyung Hee University Seoul Republic of Korea; ^3^ Convergence Medicine Research Center Asan Institute for Life Science Asan Medical Center University of Ulsan College of Medicine Seoul South Korea; ^4^ Department of Pediatrics Chonnam National University Hospital Chonnam National University Medical School Gwangju Republic of Korea; ^5^ Asan Medical Center Humidifier Disinfectant Health Center Seoul Republic of Korea; ^6^ Department of Convergence Medicine Asan Institute for Life Science Asan Medical Center University of Ulsan College of Medicine Seoul South Korea; ^7^ Department of Pediatrics Childhood Asthma Atopy Center Humidifier Disinfectant Health Center Asan Medical Center University of Ulsan College of Medicine Seoul Republic of Korea; ^8^ Department of Pulmonary and Critical Care Medicine Asan Medical Center University of Ulsan College of Medicine Seoul Republic of Korea; ^9^ National Center for Efficacy Evaluation of Respiratory Disease Product Korea Institute of Toxicology Jeollabuk‐do Republic of Korea; ^10^ Department of Human and Environmental Toxicology University of Science & Technology Daejeon Republic of Korea; ^11^ Department of Inhalation Toxicology Research Korea Institute of Toxicology Jeollabuk‐do Republic of Korea; ^12^ Department of Pathology Research Korea Institute of Toxicology Jeollabuk‐do Republic of Korea; ^13^ Department of Biomedical and Pharmaceutical Sciences Kyung Hee University Seoul Republic of Korea

Dear Editor,

Inhalational exposure to toxic chemicals present in humidifier disinfectant (HD), such as polyhexamethylene guanidine (PHMG), was identified as a cause of the serious lung injury,^1^, ^2^, ^3^ and this fatal humidifier disinfectant‐associated lung injury (HDLI), as characterized by rapid progression of respiratory failure with lung fibrosis and frequent air leak syndrome with high mortality, was classified as a subcategory of interstitial lung diseases (ILDs).^4^, ^5^ However, differences in clinical progression, high mortality (44–58%), and pathology between HDLI and previously identified ILDs have aroused interest over the mechanisms of these serious lung diseases.^1^, ^2^, ^6^ The aim of the present study was to explore regulatory molecules and gain insight into the comprehensive biological processes associated with HDLI using integrated multispecies multi‐omics of human and rat lung tissues exposed to PHMG. Study subjects and their clinical characteristics are summarized in Tables S1 and S2. The most common symptoms at admission were coughing and tachypnea in children with HDLI, and chest wall retraction and cyanosis in adult patients with HDLI.

A global expression showed a distinct clustering of patients with HDLI from controls (Figure 1A,B). Among the differentially expressed genes (DEGs) as shown in Table S3, given the overproduction of collagen during pulmonary fibrosis, significant up‐regulation of collagen‐related gene of top *COL1A2* (log_2_ fold change = 2.58, *P*_FDR_ = 0.04) in the present study was notable. Further comparison of gene expressions between human and rat lung tissues (Tables S4 and S5, Figure S1) relative to controls identified eight overlapping genes (Table S6, Figure 1C). Among them, several genes (including *MMP2*, *SERPINF1*, and *A2M*) have been previously reported to be associated with lung diseases such as idiopathic pulmonary fibrosis (IPF). In the ingenuity pathway analysis (IPA), significant networks, canonical pathway, and disease/bio‐function were predicted in human lung tissues with HDLI and rat lung tissues exposed to PHMG (Tables S7–S9). Notably, hepatic "fibrosis" in canonical pathway and respiratory disease in network and disease/bio‐function were predicted to be related to HDLI.

**FIGURE 1:**
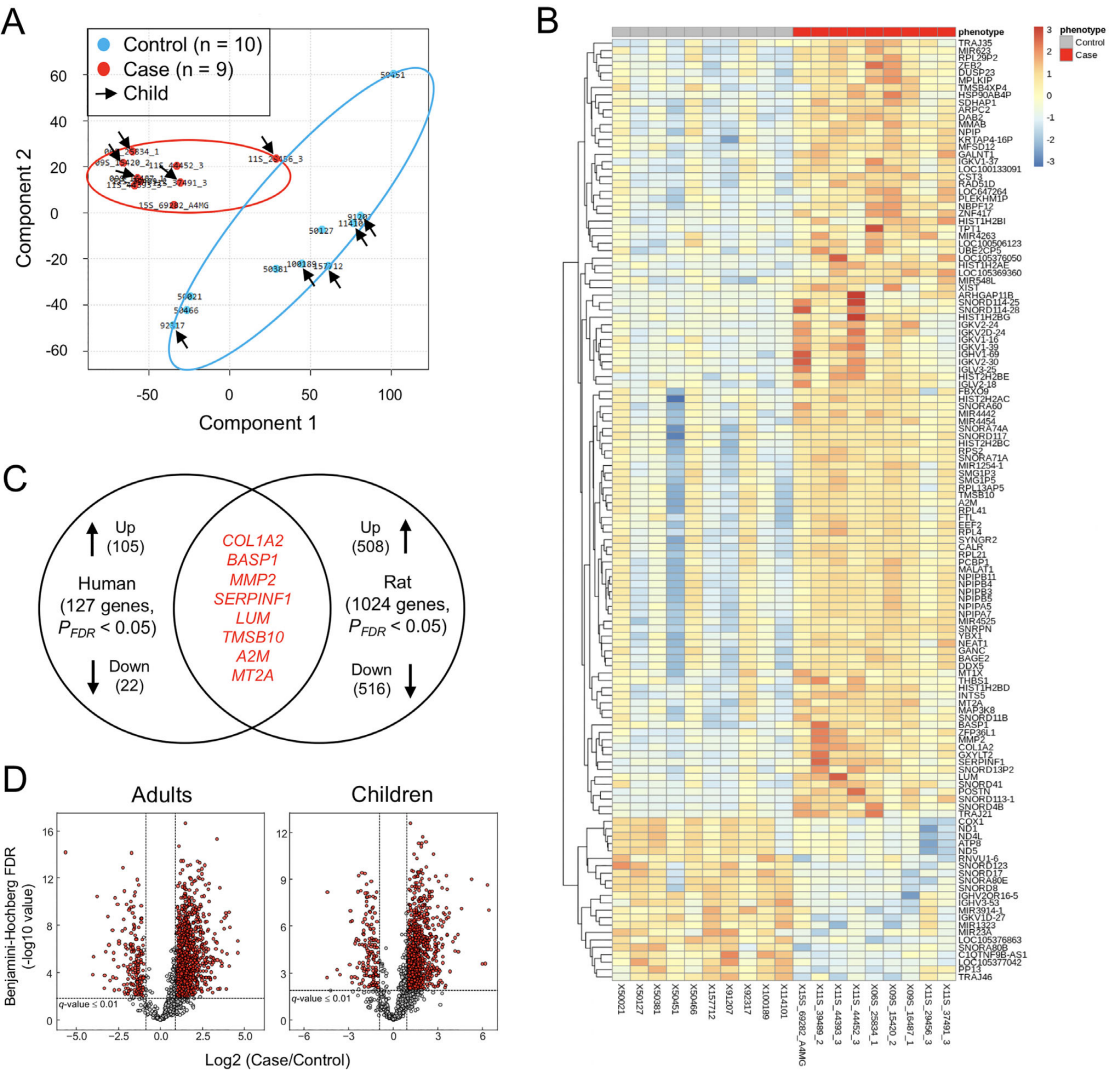
Transcriptome and proteome analyses of human lung tissues from patients with HDLI and rat lung tissues from animals exposed to PHMG. (A) A multidimensional scaling (MDS) plot corresponding to gene expression showing a relatively clear separation between patients with HDLI (red, n = 9) and unaffected controls (bright blue, n = 10). (B) Heat map of 127 genes differentially expressed between the two groups (fold change ≥ 2, PFDR < 0.05). The maximum value (red) of each gene is set to 3, the minimum value (blue) is set to ‐3, and the remaining values are linearly fitted. (C) Genes overlapping between human lung tissues with HDLI and rat lung tissues exposed to PHMG. All eight overlapping genes were up‐regulated. Genes associated with lung diseases such as IPF have been previously reported (see the Online references). (D) Volcano plot of proteomic data. Proteins are ranked according to q‐value (y‐axis) and their relative abundance ratio (Log2 fold change) between patients vs. controls (x‐axis). Proteins with a q‐value ≤ 0.01 (horizontal dotted line) and fold change ≥ 2 (vertical dotted lines) were considered as DEPs.

Interactome network analysis revealed higher DEG enrichment in interacting together rather than control genes (Figure S2), indicating that genes engaging in significant interactions with DEGs might play a disease‐associated role. Moreover, this interaction landscape reflected the proteome results (Figure 1D, Figure S3, Table S10): the interaction enrichment of DEGs showed a strong positive correlation (*r* = 0.637, *p* < 0.001) with interaction enrichment of differentially expressed proteins (DEPs) based on protein expression analysis (Figure S4, Table S11). Intriguingly, MMP2, which was a core gene and consistently identified in both human and rat transcriptome datasets, was found to interact strong with DEGs in both children and adults (Figure 2A), and increased MMP2 protein levels were observed in formalin‐fixed paraffin‐embedded (FFPE) specimens, along with hypo‐methylation in transcription start site and promoter regions of *MMP2* (Figure 2B, Figure S5).

**FIGURE 2:**
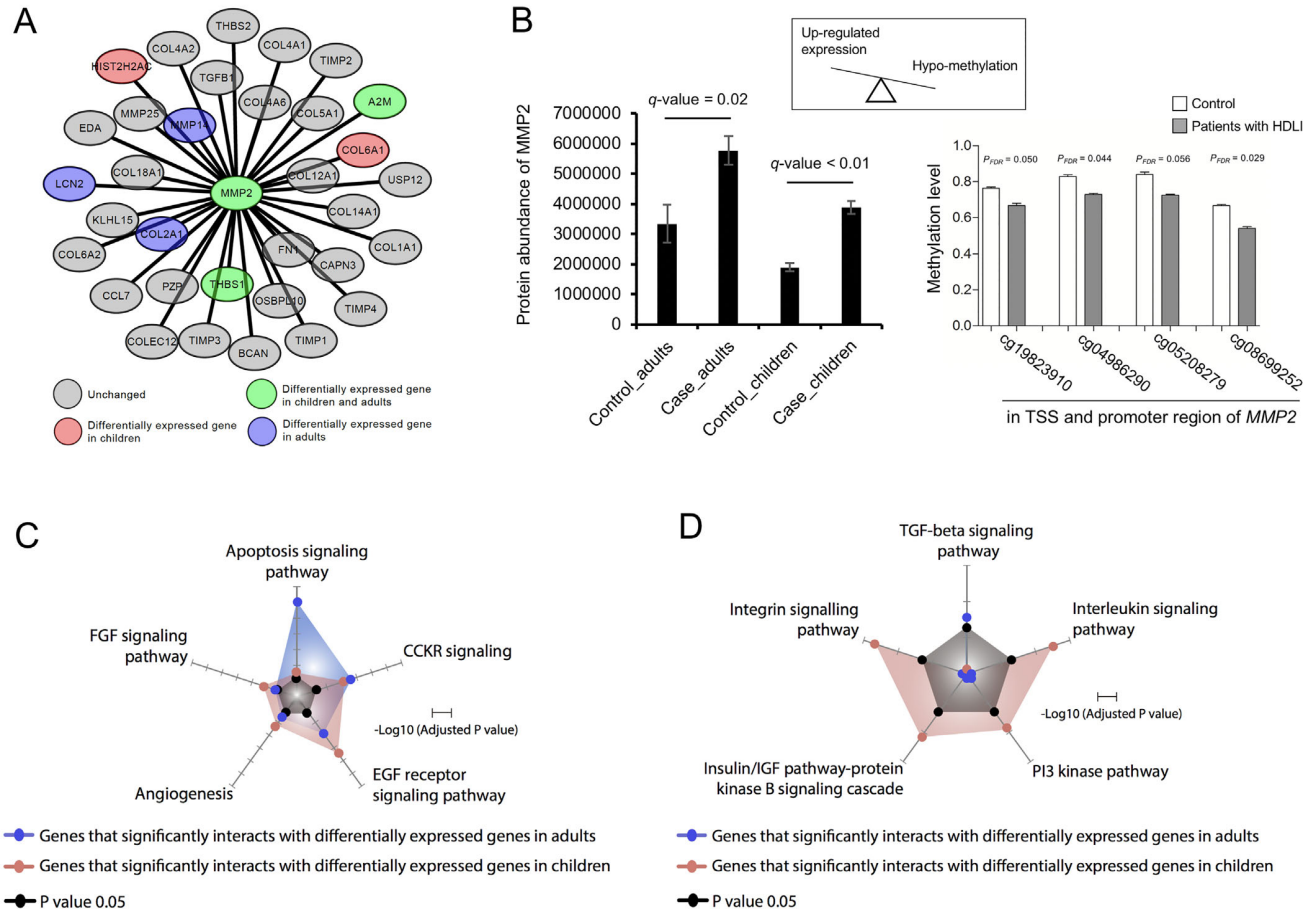
Interactome network and protein expression/methylation analyses. (A) MMP2, which was significantly increased at the protein level in humidifier disinfectant‐associated lung injury (HDLI) cases and at the gene expression in rats exposed to humidifier disinfectant (HD), was identified as a core gene interacting with other child‐specific (red) and adult‐specific (blue) differentially expressed genes (DEGs). Genes differentially expressed in both children and adults are colored as green. (B) MMP2 protein abundance in human lung FFPE tissue from adults (*n* = 4 in cases vs. *n* = 3 in controls) and children (*n* = 3 in cases vs. *n* = 2 in controls), and hypo‐methylation in TSS and promoter regions of *MMP2*. *q*‐Value from Benjamini–Hochberg FDR. TSS, transcription start site. (C) Pathways enriched in both children and adults. (D) Pathways enriched in either children or adults. All pathways were predicted from genes significantly interacting with DEGs (*p*‐values corrected for multiple testing). These DEGs were systematically expanded by using the protein interactome network

In further re‐analysis based on gene expression profiles from children (Table S12) and adults (Table S13), similarly and differentially enriched pathways between children and adults were predicted (Figure 2C,D, Table S14). The well‐established fibrosis‐related TGF‐β/SMAD signaling was the major pathway identified in adults (Figures 2D and 3A,B, Tables S14 and S15), while other potential pathways were identified in children, of which integrin signaling (Figures 2D and 3C,D; *p* = 0.002, adjusted *p* = 0.02 in Tables S14 and S16) appeared to be the most important among child‐specific signaling pathways, as also shown in gene set enrichment analysis (GSEA) analysis (Figure 3E). Based on our transcriptomic/proteomic profiling and network analysis results, different MMP2‐mediated mechanisms appear to operate in children and adults during HDLI development, suggesting that pre‐exposure to toxic materials in the environment may affect apoptosis signaling in adults. Furthermore, exposure to toxic PHMG‐containing HD may promote activation of TGF‐β/SMAD signaling in adults. Meanwhile, different signaling pathways were observed in children with HDLI and integrin (also known as a potential activator of MMPs and mediator of fibrosis and TGF‐β activation)^7^ was identified to be crucial in children with HDLI (Figure 4A).

**FIGURE 3:**
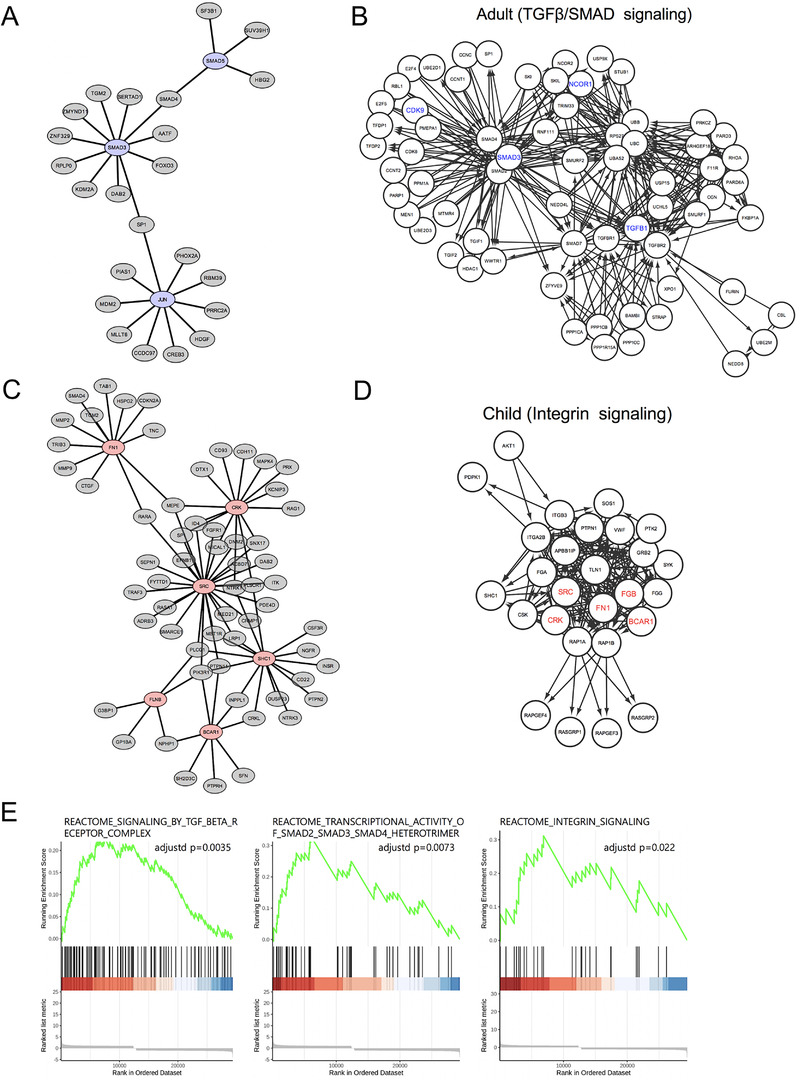
Subnetwork and signaling pathway analyses in children and adults. (A) The largest subnetwork of genes (blue nodes) significantly interacting with adult‐specific differentially expressed genes (DEGs) (gray nodes) in the interactome network. (B) Genes (blue labels) significantly interacting with adult‐specific DEGs in TGF‐β (Table S15) and SMAD3 signaling pathways in the Reactome. (C) The largest subnetwork of genes (red nodes) significantly interacting with child‐specific DEGs (gray nodes) in the interactome network. (D) Genes (red labels) significantly interacting with child‐specific DEGs in the integrin signaling pathway in the Reactome (Table S16). (E) Gene set enrichment analysis (GSEA) of the identified signaling pathways. Pathways of TGF‐β, SMAD3, and integrin signaling were significantly enriched with the pattern of DEGs in the relative groups of children and adults

**FIGURE 4:**
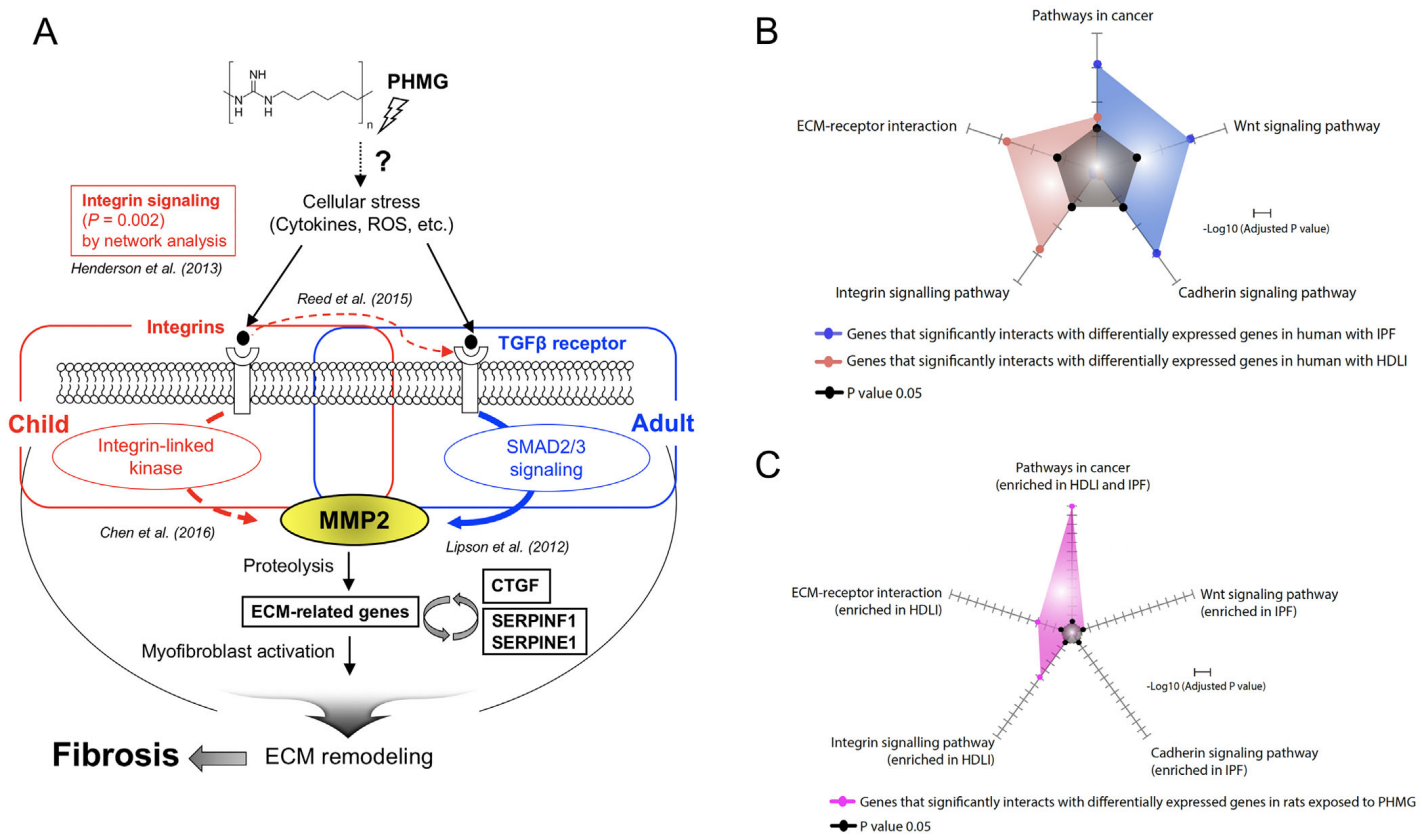
A possible model for humidifier disinfectant‐associated lung injury (HDLI) and interactome network comparisons with idiopathic pulmonary fibrosis (IPF) and rats exposed to polyhexamethylene guanidine (PHMG). (A) PHMG‐containing humidifier disinfectant may induce cellular stress in immune cells, activating the TGF‐β/SMAD‐independent pathway in adults with HDLI and integrin signaling in children, respectively. However, pathways in children and adults may be partially cross‐linked, leading to activation of extracellular matrix (ECM)‐related genes, which induces fibrosis via ECM remodeling. In the case of TGF‐β/SMAD signaling in adults with HDLI, pre‐exposure to toxic materials in the environment in adults may have already promoted the activation of TGF‐β/SMAD signaling, a key pathway in lung fibrosis. If so, exposure to toxic PHMG may further stimulate the TGF‐β/SMAD pathway as previously demonstrated in a mouse model [*Inhal Toxicol*. 2020; 32: 419‐430]. By contrast, several potential pathways were predicted in children with HDLI (Table S14). Increased expression of integrin, a potential activator of MMPs, is associated with lung fibrosis in young mice including in a bleomycin‐induced animal model [*Transl Res*. 2015; 166: 554‐567], suggesting that the integrin signaling pathway might be the most prominent pathway in children with HDLI. See additional online references. (B) Enriched pathways resulting from interactome network analysis of HDLI and IPF. All pathways were predicted from genes significantly interacting with differentially expressed genes (DEGs) related to HDLI or IPF. (C) Enriched pathways resulting from interactome network analysis of rats exposed to PHMG. Pathway analysis performed in humans with HDLI and IPF was carried out for genes significantly interacting with the DEGs in the lung tissues of rats exposed to PHMG

The present study further compared pathways identified from interactome network analysis of HDLI and IPF (the most common subtype of ILDs in adults) using previously reported IPF transcriptome data (Table S17) compared to public datasets.^8^ Extracellular matrix (ECM)–receptor interaction and integrin signaling pathways were enriched in the patients with HDLI (Figure 4B). The integrin signaling pathway plays a role in fibrosis associated with human diseases and has a possible relationship to the CCKR and EFG receptor pathways (highly significant in both adults and children, Table S14) associated with fibrosis.^9^, ^10^ These differences in pathways relevant to HDLI and IPF, along with highly replicated enrichment of transcriptomes in the lung tissues of humans and rats exposed to PHMG (Figure 4C), imply that the potential mechanisms related to HDLI may differ between IPF and other types of pulmonary fibrosis. The predictions from the lung transcriptomes of PHMG‐exposed rats revealed similar signaling pathways with those previously reported in pulmonary fibrosis (Table S18); however, there were differences between the PHMG‐exposed rats and clinical cases of fibroblast activation and pulmonary fibrosis regarding STAT3, endothelin‐1, and Wnt/β‐catenin signaling pathways, suggesting that PHMG exposure has a distinct pathogenesis from other fibrotic lung diseases.

Limitations of the present study are the relatively small number of samples, due to the scarcity of HDLI patients and the low availability of human lung tissues, and insufficient evaluation of the functions of the identified candidates. Although disease severity, stage, and progression during tissue acquisition might affect gene expression, we were unable to account for this factor in the present work. However, to minimize this, we analyzed lung samples during the acute phase, and replicated human expression changes in the rat model following exposure to PHMG. In addition, we integrated multispecies multi‐omics (human transcriptomics, methylation, proteomics, and rat transcriptomics) approaches, and performed interactome network analysis, to avoid issues with its low coverage in analyzed data. In summary, this study identified several DEGs and further validated DEPs in HDLI, along with differences in mechanisms between children and adults and human–rat commonality, hoping that these results may provide new insight into the HDLI pathogenesis and chemical‐induced lung injury and fibrosis.

## CONFLICT OF INTEREST

The authors declare that there is no conflict of interest.

## Supporting information

SUPPORTING INFORMATION

SUPPORTING INFORMATION
