# Supplementary material for: Integrative multi‐omics approach for mechanism of humidifier disinfectant‐associated lung injury
Source: Clin Transl Med. 2021 Nov 4;11(11):e562. doi: 10.1002/ctm2.562 (PMC8567041; doi:10.1002/ctm2.562)
Supplement: Supplementary file 1 — SUPPORTING INFORMATION [file CTM2-11-e562-s002.docx]

**Online Methods**

**Human subjects**

This study included lung tissues from five children and four adults, all with HDLI (Tables S1-S2). The study protocol was approved by the Institutional Review Board (IRB) of Asan Medical Center (IRB no. 2016-0885). All patients were confirmed to have HDLI based on both pathological and radiological findings. Acute HDLI patients used HD products containing only PHMG. As controls, adjacent normal tissues of lung tumors from five children and five adults were obtained from the Bio-Resource Center, Asan Medical Center, with permission. The mean age of children in the control group was 11.4 years, and that of adults was 53.2 years.

**Animals**

Twelve-week-old nulliparous female and male Sprague-Dawley rats were purchased from Orient Bio (Seoul, Korea). All animals used in this study were approved by the Institutional Animal Care and Use Committee (IACUC, approval no. 1612-0461) of the Korea Institute of Toxicology. PHMG phosphate was generated using an NB-2N mist generator (Sibata, Japan), and supplied using a WITC1.5 whole-body inhalation chamber (HCT, Korea). Generated PHMG phosphate was diluted with dilution air to achieve the target concentration. Two groups, each comprising 13 pregnant female rats, were exposed to whole-body target PHMG phosphate concentrations of 0 (Control) or 3.20 mg/m^3^ for 6 hours per day, 7 days per week, from day 6 to day 20 of gestation.

**Gene expression profiling of human and rat subjects, and data analysis**

Twenty FFPE of human lung tissues and 20 frozen rat lung tissues were used for mRNA extraction with a miRNeasy FFPE kit (Qiagen, Hilden, Germany) and TRIzol reagent (Invitrogen, Carlsbad, CA, USA) according to the manufacturer’s protocol. Among the 20 FFPEs, one patient sample failed to meet mRNA quality control and was excluded from further analysis. For human FFPE lung tissue, cDNA was synthesized using a GeneChip WT Pico Reagent kit (Affymetrix, Santa Clara, CA, USA) as described by the manufacturer, and hybridized onto a GeneChip® Human Gene 2.0 ST Array (Affymetrix). For transcriptome analysis of frozen lung tissue from PHMG-exposed (n = 10) and unexposed control (n = 10) rats, cDNA was synthesized using a GeneChip WT amplification kit (Affymetrix) and hybridized onto a GeneChip® Rat Gene 2.0 ST Array (Affymetrix). Array data export processing and analysis were performed using Affymetrix® GeneChip Command Console software, with further RMA analysis for background correction, summarization, and normalization. After filtering of control probes, 44,628 probes among 53,617 probes for the human chip and 29,489 probes among 36,685 probes for the rat chip were analyzed. All transcriptome data of human and rat was deposited in the GEO of public NCBI (accession numbers: GSE179445, GSE179446, GSE179447). Genes exhibiting both a fold change ≥ 2 and a *t*-test *P-*value < 0.05 were considered as significantly differentially expressed. Multiple dimensional scaling (MDS) was calculated by the degree of similarity (Euclidean distance) between each sample using the normalized signal of all detected probe sets in the chip, and then plotted based on the first two components that best explain the variability of the entire data.

**Proteomic analysis of proteins from human lung FFPE tissues**

Deparaffinization and protein extraction were performed using the lung FFPE tissues with a Qproteome FFPE tissue kit (Qiagen, Hilden, Germany), and proteins were proteolyzed using a trypsin/lysC mixture (Promega, Madison, WI, USA). Peptide samples were analyzed by Q Exactive plus mass spectrometer coupled with Dionex UltiMate 3000 RSLCnano system (Thermo Fisher Scientific, Bremen, Germany). The SequestHT search algorithm within Proteome discoverer (version 2.2, Thermo Fisher Scientific) against the SwissProt database (May 2017). Briefly, precursor mass tolerance was set to ± 10 ppm and MS/MS tolerance was set at 0.02 Da. The search parameters were set as default including cysteine carbamidomethylation as a fixed modification, N-terminal/lysine acetylation, methionine oxidation, phospho- serine, -threonine and -tyrosine as variable modifications with 2 miscleavages. False discovery rates (FDRs) were set for 1% for each analysis using “Percolator”. From the Sequest search output, Peptide filters which peptide confidence, peptide rank, score versus charge state and search engine rank were default value of proteome discoverer. And label free quantitation was performed using peak intensity for unique and razor peptide of each protein. Normalization was done using total peptide amount. The mass spectral dataset (PXD026832) representing the proteomics data has been deposited in the ProteomeXchange Consortium (http://proteomecentral.proteomexchange.org) via the PRIDE (*Nucleic Acids Res. 2019;47(D1):D442-D450*) partner repository.

**DNA methylation profiling**

DNAs extracted from lung tissues from three adults with HDLI and that from tumor-adjacent normal tissues from four adults with lung cancer were analyzed by using the Infinium HumanMethylationEPIC BeadChip (Illumina, San Diego, CA, USA). Threshold for statistical significance for the differentially methylated CpG sites was set at |Δβ| > 0.2 and *P* < 0.05 in a *t*-tests.

**Network analysis**

To represent the functional networks of differentially expressed genes (DEGs), the quantitation data were uploaded onto the ingenuity pathways analysis (IPA; Ingenuity Systems, Redwood City, CA, USA) program. The upstream analysis and regulator effects were generated through IPA (QIAGEN Inc., <https://www.qiagenbioinformatics.com/products/ingenuity-pathway-analysis>). The IPA analysis, which calculates two distinct statistics as part of a core analysis from https://www.qiagen.com/, was performed according to the manufacturer’s instructions; briefly, all up- and down-regulated genes with significance at |logFC| ≥ 1.5 were input, and *P*-value was calculated using the Right-Trailed Fisher’s Exact Test. Furthermore, integrated network analysis was performed on child- and adult-specific DEGs using protein-protein interaction (PPI) and pathway databases. An interactome map encompassing two major physical interaction networks, referred to as BioPlex 2.0 and HI-II-14, was used. These two interaction networks were created by detecting associated proteins by mass spectrometry and merging yeast two-hybrid-based proteome-scale interacting pairs. After identifying genes that interacting with DEGs using interactome map, the Enrichr package was used to analyze significantly interacting genes on the basis of pathways and functional terms retrieved from Panther, Reactome, Kyoto Encyclopedia of Genes and Genomes (KEGG), and Gene Ontology (GO) databases.

**Gene set enrichment analysis (GSEA)**

GSEA for the DEGs identified from child and adult HDLI patients was performed using the R package ClusterProfiler (v.3.14.3) (*OMICS. 2012;16(5):284-287*) with default parameters. For the pathway mapping, H (hallmark gene set), C2 (curated gene set), and C5 (ontology gene set) were retrieved from the database of the R package msigdb (v.0.2.0) (*Bioinformatics. 2011;27(12):1739-1740*). Only significantly enriched pathways (adjusted *P*-value < 0.05) were searched to be compared with the TGFβ, SMAD3, and integrin signaling pathways that were identified from our network analysis.

**Statistical analysis**

Statistical significance (corrected *P* < 0.05) was determined using a *t*-tests. For child- and adult-specific transcriptome analysis, raw *P*-values were used to define statistical significance. DEGs were then projected onto the interactome map, and the extent of interactions between was estimated using hypergeometric tests. Corrected *P*-values of false discovery rate (FDR) < 0.05 were used for each hypergeometric test to define significant interactions of corresponding genes.

For differential analysis of the relative abundance of proteins between samples, the free software Perseus (version 1.6.5.0) was used (*Nat Methods. 2016;13(9):731-740*). The values of the abundance of normalized proteins were transformed into log_2_ scale. Three technical replicates of each sample were grouped and a minimum of three valid values was required in each group. To find statistically significant differences between the patient and control groups, *t*-test was performed using Benjamini-Hochberg FDR (cut-off = 0.01). For volcano plot, these data were plotted on a log_2_ scale on the x-axis and the calculated probability (p-value) on a -log_10_ scale on the y-axis using InstantClue software (*Sci Rep. 2018;8(1):12648*).

**Online references**

(A) Figure 1. Genes associated with lung diseases such as IPF have been previously reported:

Kim YI, Shin HW, Chun YS, et al. Epithelial cell-derived cytokines CST3 and GDF15 as potential therapeutics for pulmonary fibrosis. *Cell Death Dis.* 2018; **9**: 506.

Kim JY, Choeng HC, Ahn C, et al. Early and late changes of MMP-2 and MMP-9 in bleomycin-induced pulmonary fibrosis. *Yonsei Med J.* 2009; **50**: 68-77.

Cosgrove GP, Brown KK, Schiemann WP, et al. Pigment epithelium-derived factor in idiopathic pulmonary fibrosis: a role in aberrant angiogenesis. *Am J Respir Crit Care Med.* 2004; **170**: 242-251.

Zhang X, Liu H, Hock T, et al. Histone deacetylase inhibition downregulates collagen 3A1 in fibrotic lung fibroblasts. *Int J Mol Sci.* 2013; **14**: 19605-19617.

Craig-Barnes HA, Doumouras BS & Palaniyar N Surfactant protein D interacts with alpha2-macroglobulin and increases its innate immune potential. *J Biol Chem.* 2010; **285**: 13461-13470.

Liu Q, Jiang JX, Liu YN, et al. Grape seed extract ameliorates bleomycin-induced mouse pulmonary fibrosis. *Toxicol Lett.* 2017; **273**: 1-9.

(B) References for integrin signaling in Fig. 4A:

Levine D, Rockey DC, Milner TA, et al. Expression of the integrin alpha8beta1 during pulmonary and hepatic fibrosis. *Am J Pathol.* 2000; **156**: 1927-1935.

Knoblauch A, Will C, Goncharenko G, et al. The binding of Mss4 to alpha-integrin subunits regulates matrix metalloproteinase activation and fibronectin remodeling. *FASEB J.* 2007; **21**: 497-510.

(C) Reference for correlation between integrin and TGF-beta in Fig. 4A:

Munger JS, Huang X, Kawakatsu H, et al. The integrin alpha v beta 6 binds and activates latent TGF beta 1: a mechanism for regulating pulmonary inflammation and fibrosis. *Cell.* 1999; **96**: 319-328.

**Supplementary figure S1**


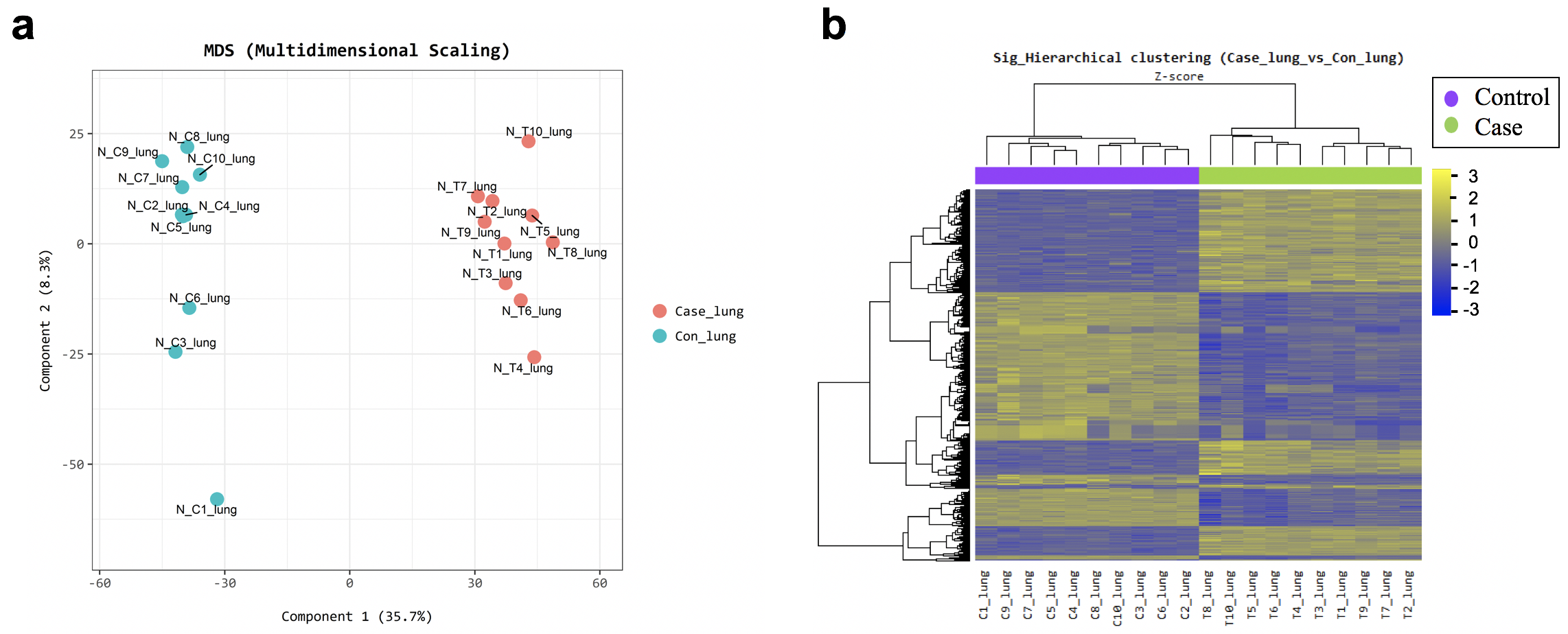


**a,** The MDS plot presents a relatively clear separation between rats exposed to PHMG (bright red) and unexposed controls (bright blue). **b,** Heat map of 1028 differentially expressed genes between the two groups (fold change ≥ 2, *P* < 0.05 by *t*-test). The maximum value (yellow) of each gene was set to 3, the minimum value (blue) was set to -3, and the remaining values were linearly fitted.

**Supplementary figure S2**

**
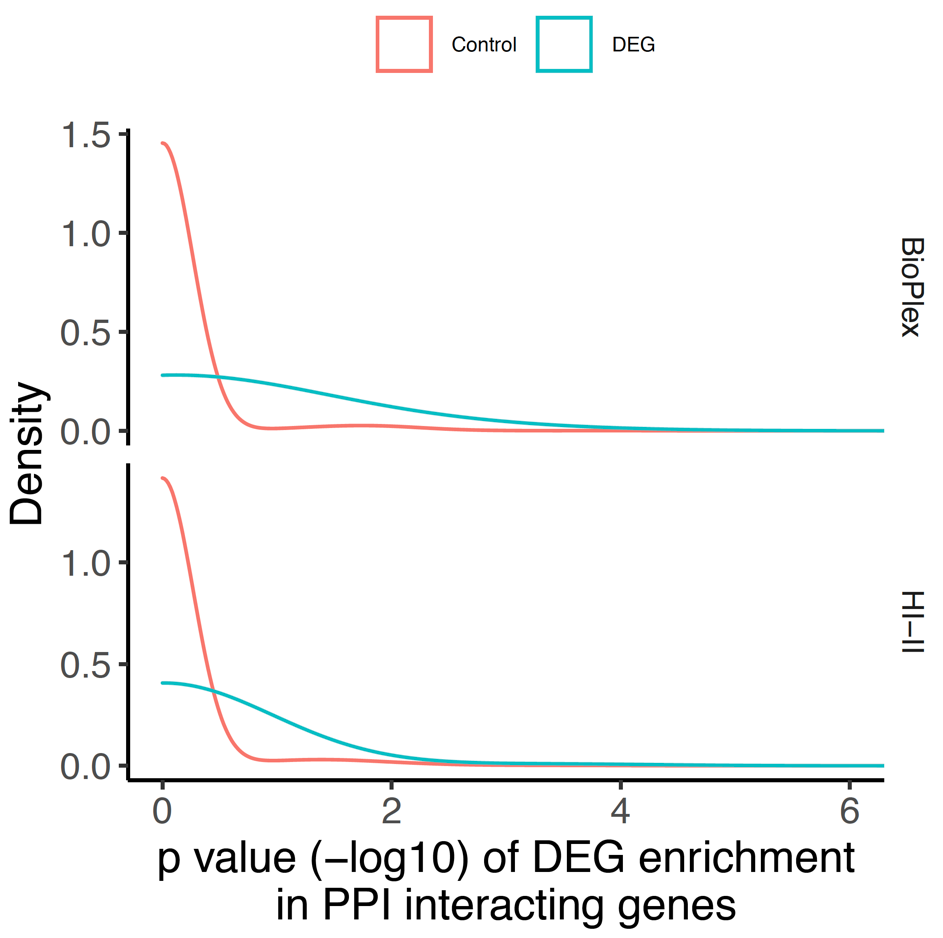
**

DEGs were tested whether they interact with other DEGs more frequently than interactions by chance. Significance of the enrichment of interactions was calculated by hypergeometric test. Non-DEGs were also tested as a control to validate how DEGs interact each other frequently. All genes that were detected by our microarray analysis were used as the background gene set. BioPlex 2.0 and HI-II-14 were used to count the interactions as interactome networks.

**Supplementary figure S3**

**
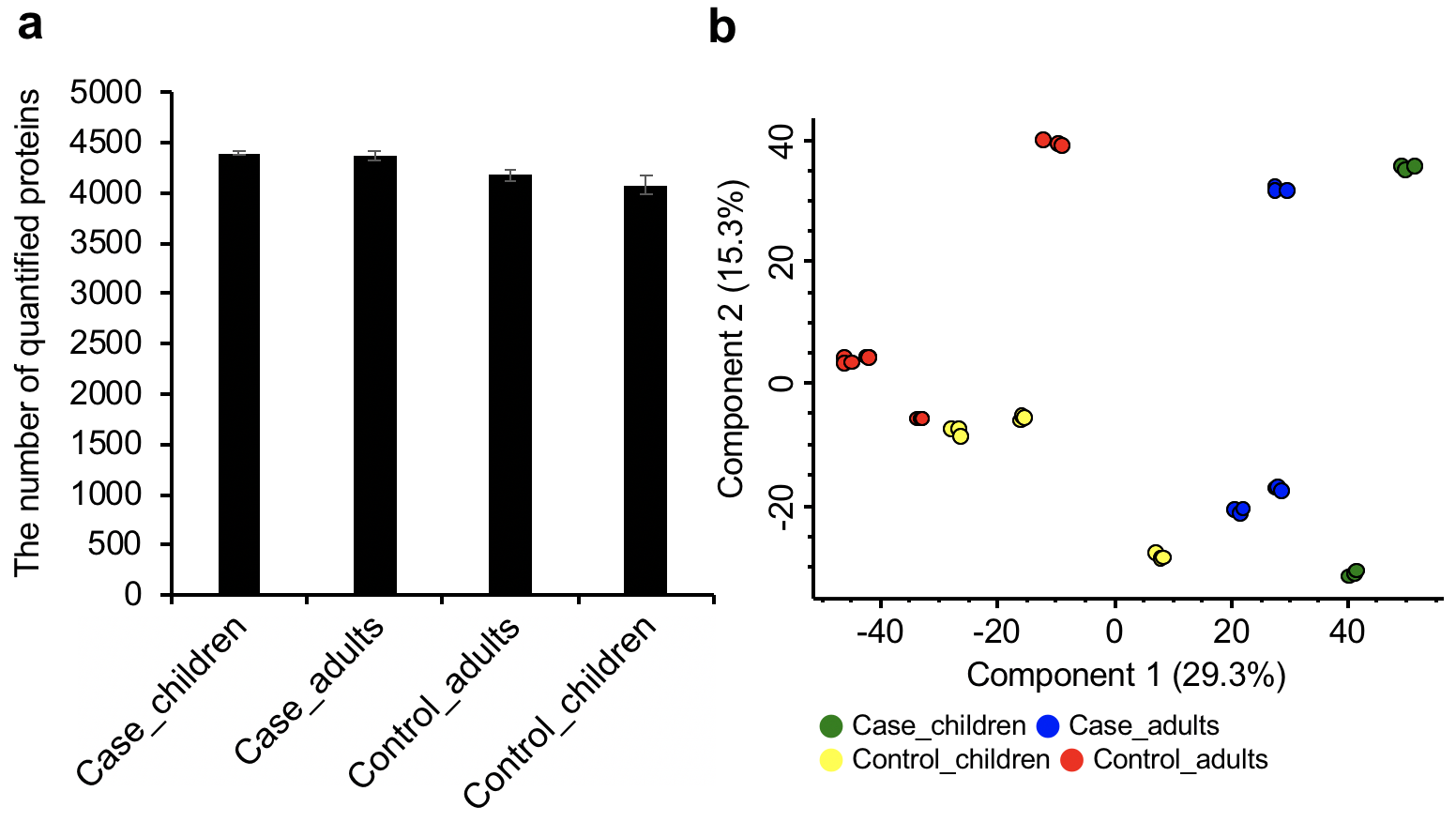
**

**a,** The number of proteins identified and quantified between patients of children and adults with HDLI and control groups. **b,** Two-dimensional scatter plot of principal component analysis showing components 1 and 2, which account for 29.3% and 15.3%, respectively, of the variability of all 3108 proteins. (red, patients; blue, control).

**Supplementary figure S4**

**
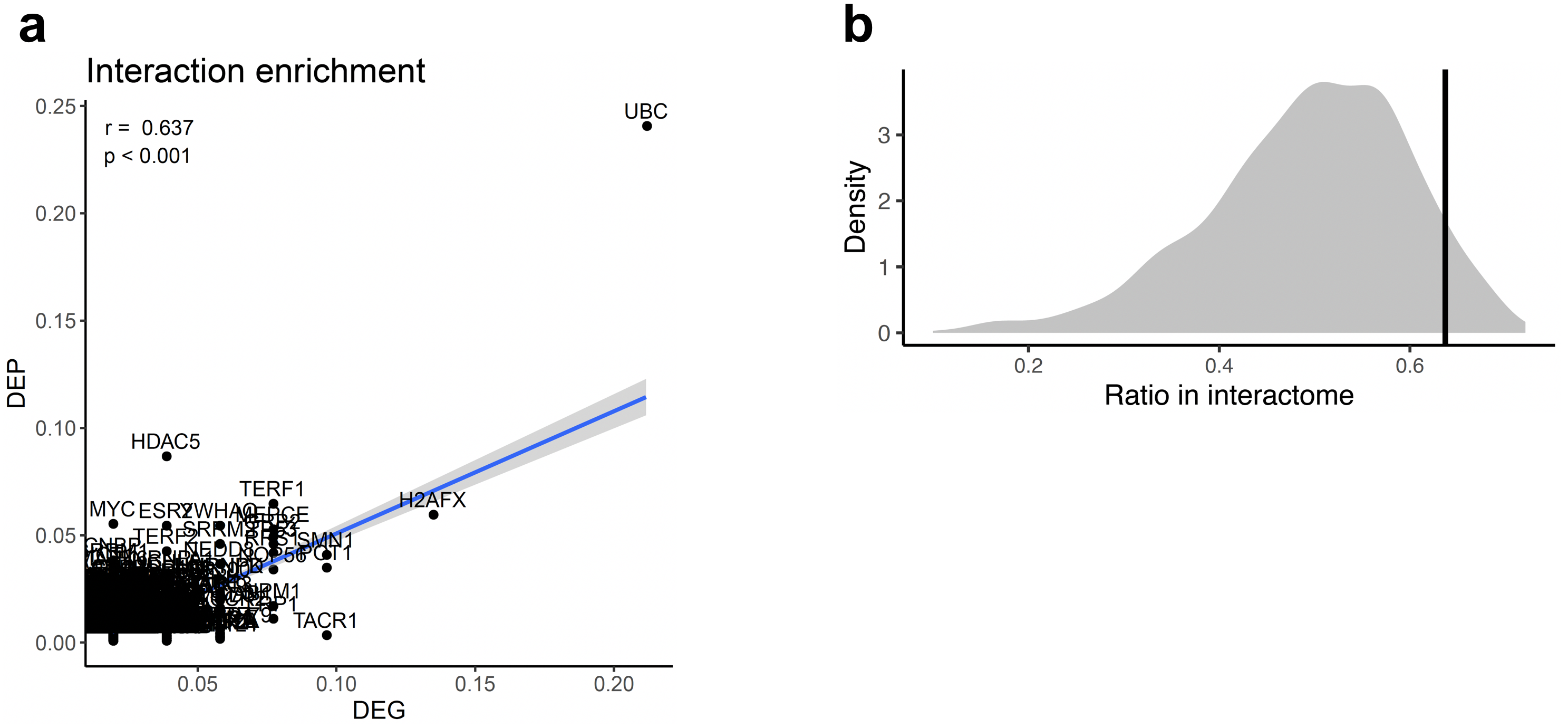
**

**a,** Enrichment of interactions for all analyzed genes was compared between DEGs and DEPs. Pearson correlation coefficient was calculated as r value. **b,** Comparison analysis between DEGs and DEPs was simulated by 1000 random permutations of respective genes and proteins. A red line indicates the correlation coefficient of our test set.

**Supplementary figure S5**

**
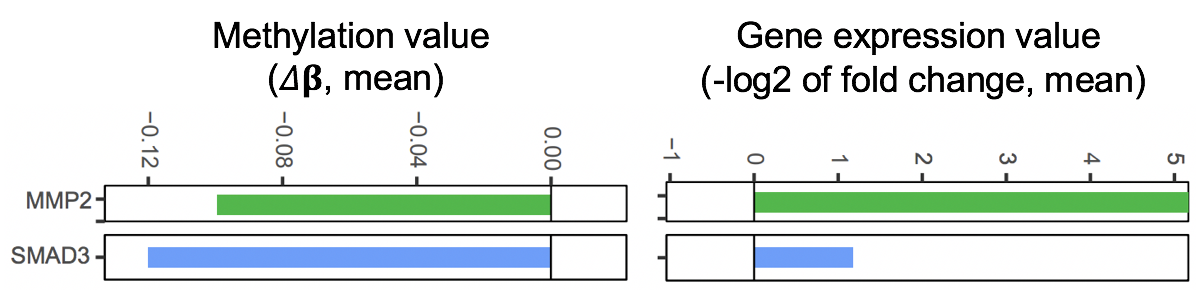
**

CpGs in promoter regions on *MMP2* and *SMAD3* were significantly hypo-methylated on our methylome profile of lung tissues from adults with HDLI and unaffected controls, and their relative gene expressions were up-regulated.

**Supplementary table S1.** List of study subjects

| No. | Subject ID | Case/Control | Adult/Child | Age (year) |
| --- | --- | --- | --- | --- |
| 1 | 06S_25834_1 | Case | Child | 12 |
| 2 | 09S_15420_2 | Case | Child | 8 |
| 3 | 09S_16487_1 | Case | Child | 9 |
| 4 | 11S_29456_3 | Case | Child | 7 |
| 5 | 11S_37491_3 | Case | Child | 5 |
| 6 | 15S_69282_A4MG | Case | Adult | 32 |
| 7 | 11S_39489_2 | Case | Adult | 35 |
| 8 | 11S_44393_3 | Case | Adult | 32 |
| 9 | 11S_44452_3 | Case | Adult | 36 |
| 10 | 157712 | Control | Child | 9 |
| 11 | 91207 | Control | Child | 13 |
| 12 | 92317 | Control | Child | 9 |
| 13 | 100189 | Control | Child | 19 |
| 14 | 114101 | Control | Child | 7 |
| 15 | 50021 | Control | Adult | 46 |
| 16 | 50127 | Control | Adult | 47 |
| 17 | 50381 | Control | Adult | 48 |
| 18 | 50451 | Control | Adult | 69 |
| 19 | 50466 | Control | Adult | 56 |

**Supplementary table S2.** Clinical profiles of patients with humidifier disinfectant-associated interstitial lung disease and unaffected controls

| Items | Cases | |  | Unaffected controls | |
| --- | --- | --- | --- | --- | --- |
|  | Children | Adults |  | Children | Adults |
| Number of subjects | 5 | 4 |  | 5 | 5 |
| Age, mean (range) | 2.6 (1.7-3.6) | 35.0 (32.0-37.0) |  | 11.4 (7.0-19.0) | 53.2 (46.0-69.0) |
| Sex (male/female) | 2/3 | 0/4 |  | NA | NA |
| Symptoms and signs at admission |  |  |  |  |  |
| Cough | 5/5 | 2/4 |  | 0/5 | 1/5 |
| Tachypnea or dyspnea | 5/5 | 3/4 |  | 0/5 | 0/5 |
| Sputum | 3/5 | 1/4 |  | 0/5 | 2/5 |
| Chest wall retraction | 3/5 | 3/4 |  | 0/5 | 0/5 |
| Fever | 0/5 | 2/4 |  | 1/5 | 0/5 |
| Rhinorrhea | 0/5 | 0/4 |  | 0/5 | 0/5 |
| Nasal ﬂaring | 0/5 | 0/4 |  | 0/5 | 0/5 |
| Cyanosis | 1/5 | 3/4 |  | 0/5 | 0/5 |
| Underlying diseases |  |  |  |  |  |
| Chronic bronchitis | 0/4 | 0/4 |  | 0/5 | 0/5 |
| Asthma | 0/4 | 0/4 |  | 0/5 | 0/5 |
| Other allergic diseases | 0/4 | 0/4 |  | 0/5 | 0/5 |
| Cardiovascular disease | 0/4 | 0/4 |  | 0/5 | 0/5 |
| Autoimmune disease | 0/4 | 0/4 |  | 0/5 | 0/5 |
| Family history of acute lung injury similar to the patient (Yes) | 2/5 | 1/4 |  | 0/5 | 0/5 |
| Pa_O2_/Fi_O2_ at admission |  |  |  |  |  |
| <200 | 1/5 | - |  | - | - |
| 200–299 | 4/5 | 4/4 |  | - | - |
| >300 | - | - |  | 5/5 | 5/5 |
| Treatment for respiratory insufﬁciency or failure |  |  |  |  |  |
| Oxygen supplementation | 5/5 | 4/4 |  | 0/5 | 0/5 |
| Ventilator care | 1/5 | 4/4 |  | 0/5 | 0/5 |
| Extracorporeal membrane oxygenation | 0/5 | 4/4 |  | 0/5 | 0/5 |
| Lung transplantation | 0/5 | 1/4 |  | 0/5 | 0/5 |
| Outcomes (Mortality) | 0/5 | 3/4 |  | 0/5 | 0/5 |

NA, not available.

**Supplementary table S3.** Differentially expressed annotated genes (fold change ≥ 2, *P_FDR_* < 0.05) in the HD-exposed human FFPE lung tissue, adjusted by age

| No | Up-/Down-  regulation | Gene | mRNA accession ID | Expression level (average, log2) | | Fold change  (Case/Control) | *P* | *P_FDR_* |
| --- | --- | --- | --- | --- | --- | --- | --- | --- |
|  |  |  |  | Control  (n = 10) | Case  (n = 9) |  |  |  |
| 1 | Up | *COL1A2* | NM_000089 | 9.82 | 25.31 | 2.58 | 6.39E-06 | 0.040 |
| 2 | Up | *MIR548L* | NR_031630 | 8.21 | 24.31 | 2.96 | 6.55E-06 | 0.034 |
| 3 | Down | *IGHV2OR16-5* | OTTHUMT00000328352 | 58.69 | 28.79 | -2.04 | 9.65E-06 | 0.012 |
| 4 | Down | *LOC105377042* | XR_937145 | 15.72 | 6.08 | -2.58 | 1.02E-05 | 0.010 |
| 5 | Up | *SNORD113-1* | NR_003229 | 2.41 | 31.63 | 13.13 | 1.07E-05 | 0.010 |
| 6 | Up | *IGKV1-39* | OTTHUMT00000323476 | 11.55 | 30.20 | 2.61 | 1.13E-05 | 0.010 |
| 7 | Up | *CST3* | NM_000099 | 21.66 | 53.14 | 2.45 | 1.23E-05 | 0.009 |
| 8 | Up | *HIST2H2BC* | NR_036461 | 49.32 | 310.50 | 6.30 | 1.49E-05 | 0.008 |
| 9 | Down | *LOC105376863* | XR_935252 | 21.73 | 9.37 | -2.32 | 1.72E-05 | 0.007 |
| 10 | Up | *SNORA71A* | NR_002911 | 73.17 | 295.55 | 4.04 | 1.89E-05 | 0.006 |
| 11 | Down | *SNORD123* | NR_003689 | 249.50 | 101.08 | -2.47 | 2.09E-05 | 0.006 |
| 12 | Up | *IGKV2-24* | OTTHUMT00000323404 | 2.35 | 11.19 | 4.76 | 2.13E-05 | 0.006 |
| 13 | Down | *SNORD17* | NR_003045 | 191.57 | 94.33 | -2.03 | 2.19E-05 | 0.006 |
| 14 | Up | *IGKV2-30* | OTTHUMT00000323491 | 4.17 | 10.34 | 2.48 | 2.50E-05 | 0.007 |
| 15 | Up | *MMAB* | ENST00000537236 | 9.13 | 60.45 | 6.62 | 2.60E-05 | 0.007 |
| 16 | Up | *BASP1* | NM_001271606 | 10.14 | 26.35 | 2.60 | 2.66E-05 | 0.007 |
| 17 | Up | *SNORD114-28* | NR_003221 | 2.11 | 6.19 | 2.93 | 2.74E-05 | 0.007 |
| 18 | Down | *IGKV1D-27* | OTTHUMT00000323279 | 87.75 | 35.22 | -2.49 | 3.01E-05 | 0.007 |
| 19 | Up | *HIST2H2BE* | NM_003528 | 2.95 | 6.99 | 2.37 | 3.08E-05 | 0.007 |
| 20 | Up | *GALNT1* | NM_020474 | 4.50 | 9.05 | 2.01 | 3.71E-05 | 0.007 |
| 21 | Up | *MPLKIP* | ENST00000306984 | 5.64 | 20.28 | 3.60 | 3.73E-05 | 0.007 |
| 22 | Up | *HIST1H2AE* | NM_021052 | 2.16 | 4.95 | 2.29 | 4.39E-05 | 0.007 |
| 23 | Up | *MIR4454* | NR_039659 | 11.98 | 40.07 | 3.34 | 4.41E-05 | 0.007 |
| 24 | Up | *POSTN* | NM_001135934 | 3.84 | 8.28 | 2.16 | 4.63E-05 | 0.007 |
| 25 | Up | *LOC105369360* | AK091996 | 2.78 | 7.53 | 2.71 | 5.10E-05 | 0.007 |
| 26 | Down | *SNORA80E* | NR_002974 | 1864.01 | 603.92 | -3.09 | 5.45E-05 | 0.007 |
| 27 | Up | *MMP2* | NM_001127891 | 6.14 | 20.34 | 3.31 | 5.54E-05 | 0.009 |
| 28 | Up | *IGLV2-18* | OTTHUMT00000321836 | 6.47 | 38.08 | 5.89 | 6.66E-05 | 0.007 |
| 29 | Down | *C1QTNF9B-AS1* | ENST00000626707 | 15.38 | 5.95 | -2.59 | 6.81E-05 | 0.007 |
| 30 | Up | *GXYLT2* | NM_001080393 | 4.29 | 9.59 | 2.23 | 6.88E-05 | 0.007 |
| 31 | Up | *MFSD12* | NM_001287529 | 3.46 | 7.25 | 2.10 | 6.98E-05 | 0.007 |
| 32 | Up | *MIR4525* | NR_039751 | 14.65 | 101.18 | 6.91 | 7.03E-05 | 0.007 |
| 33 | Up | *IGKV1-16* | OTTHUMT00000323398 | 14.26 | 59.74 | 4.19 | 7.06E-05 | 0.007 |
| 34 | Up | *HSP90AB4P* | NR_073415 | 2.81 | 6.25 | 2.22 | 7.23E-05 | 0.007 |
| 35 | Up | *KRTAP4-16P* | OTTHUMT00000257694 | 9.63 | 19.99 | 2.08 | 7.72E-05 | 0.007 |
| 36 | Up | *MIR4442* | NR_039644 | 55.87 | 114.17 | 2.04 | 7.78E-05 | 0.007 |
| 37 | Down | *PP13* | XR_243769 | 17.62 | 8.76 | -2.01 | 8.24E-05 | 0.007 |
| 38 | Up | *ARPC2* | NM_005731 | 20.18 | 46.60 | 2.31 | 9.52E-05 | 0.008 |
| 39 | Up | *ZFP36L1* | NM_001244698 | 8.74 | 18.98 | 2.17 | 9.60E-05 | 0.008 |
| 40 | Up | *SNORD114-25* | NR_003218 | 2.03 | 5.60 | 2.75 | 0.0001 | 0.008 |
| 41 | Up | *IGKV1-37* | OTTHUMT00000323488 | 7.21 | 16.05 | 2.23 | 0.0001 | 0.008 |
| 42 | Up | *SNORD41* | NR_002751 | 5.71 | 30.79 | 5.39 | 0.0001 | 0.008 |
| 43 | Up | *IGLV3-25* | BC022823 | 52.27 | 168.96 | 3.23 | 0.0001 | 0.008 |
| 44 | Down | *SNORD8* | NR_002916 | 2554.44 | 1219.93 | -2.09 | 0.0001 | 0.008 |
| 45 | Up | *NPIP* | XR_917060 | 49.14 | 113.12 | 2.30 | 0.0001 | 0.008 |
| 46 | Up | *IGKV2D-24* | OTTHUMT00000323290 | 3.37 | 11.80 | 3.51 | 0.0001 | 0.008 |
| 47 | Up | *RPS2* | NM_002952 | 223.67 | 548.97 | 2.45 | 0.0001 | 0.009 |
| 48 | Up | *EEF2* | NM_001961 | 9.71 | 22.22 | 2.29 | 0.0002 | 0.009 |
| 49 | Up | *HIST2H2AC* | NM_003517 | 27.81 | 79.15 | 2.85 | 0.0002 | 0.009 |
| 50 | Up | *SERPINF1* | NM_002615 | 4.40 | 10.62 | 2.41 | 0.0002 | 0.009 |
| 51 | Up | *NPIPA7* | NM_001282507 | 48.99 | 154.33 | 3.15 | 0.0002 | 0.009 |
| 52 | Up | *NPIPB5* | NM_001135865 | 23.29 | 66.05 | 2.84 | 0.0002 | 0.009 |
| 53 | Down | *TRAJ46* | OTTHUMT00000410952 | 20.14 | 9.46 | -2.13 | 0.0002 | 0.009 |
| 54 | Up | *TPT1* | NM_001286272 | 3.36 | 8.37 | 2.49 | 0.0002 | 0.009 |
| 55 | Up | *CALR* | NM_004343 | 10.42 | 30.90 | 2.96 | 0.0002 | 0.009 |
| 56 | Up | *NBPF12* | ENST00000439206 | 2.73 | 7.20 | 2.64 | 0.0002 | 0.010 |
| 57 | Up | *SNRPN* | AF400487 | 11.39 | 110.01 | 9.65 | 0.0002 | 0.010 |
| 58 | Up | *SNORD13P2* | X58060 | 28.08 | 62.04 | 2.21 | 0.0002 | 0.010 |
| 59 | Up | *RPL29P2* | NR_002778 | 9.06 | 18.77 | 2.07 | 0.0002 | 0.010 |
| 60 | Up | *DUSP23* | NM_017823 | 10.21 | 20.63 | 2.02 | 0.0002 | 0.010 |
| 61 | Up | *MAP3K8* | XM_011519310 | 49.32 | 304.26 | 6.17 | 0.0002 | 0.011 |
| 62 | Up | *SNORD4B* | NR_000009 | 2.41 | 6.05 | 2.51 | 0.0002 | 0.011 |
| 63 | Up | *SMG1P5* | NR_002453 | 13.14 | 26.88 | 2.05 | 0.0002 | 0.011 |
| 64 | Up | *NPIPB11* | NM_001310137 | 53.15 | 195.47 | 3.68 | 0.0002 | 0.011 |
| 65 | Up | *MIR623* | NR_030353 | 5.33 | 24.88 | 4.67 | 0.0002 | 0.011 |
| 66 | Up | *SNORA60* | NR_002986 | 39.08 | 171.67 | 4.39 | 0.0003 | 0.011 |
| 67 | Up | *ARHGAP11B* | OTTHUMT00000430733 | 2.73 | 5.70 | 2.09 | 0.0003 | 0.011 |
| 68 | Up | *LOC105376050* | XR_929616 | 2.00 | 4.73 | 2.36 | 0.0003 | 0.011 |
| 69 | Up | *IGHV1-69* | AF035025 | 58.18 | 148.31 | 2.55 | 0.0003 | 0.011 |
| 70 | Up | *ZNF417* | ENST00000594396 | 3.26 | 6.75 | 2.07 | 0.0003 | 0.011 |
| 71 | Up | *HIST1H2BI* | NM_003525 | 3.79 | 12.06 | 3.18 | 0.0004 | 0.012 |
| 72 | Up | *LUM* | NM_002345 | 5.31 | 10.71 | 2.02 | 0.0004 | 0.012 |
| 73 | Down | *COX1* | ENST00000361624 | 853.32 | 212.02 | -4.02 | 0.0004 | 0.013 |
| 74 | Up | *DAB2* | NM_001244871 | 4.99 | 10.58 | 2.12 | 0.0004 | 0.013 |
| 75 | Up | *NPIPB4* | ENST00000520915 | 54.13 | 178.45 | 3.30 | 0.0004 | 0.013 |
| 76 | Up | *SDHAP1* | NR_003264 | 30.68 | 94.45 | 3.08 | 0.0004 | 0.013 |
| 77 | Up | *YBX1* | NM_004559 | 18.48 | 37.27 | 2.02 | 0.0004 | 0.013 |
| 78 | Down | *SNORA80B* | NR_028374 | 88.45 | 40.76 | -2.17 | 0.0004 | 0.014 |
| 79 | Up | *RAD51D* | ENST00000345365 | 10.78 | 36.79 | 3.41 | 0.0005 | 0.014 |
| 80 | Up | *NPIPB3* | NM_130464 | 25.50 | 75.18 | 2.95 | 0.0005 | 0.014 |
| 81 | Up | *ZEB2* | ENST00000628473 | 4.88 | 13.69 | 2.80 | 0.0005 | 0.014 |
| 82 | Down | *IGHV3-53* | OTTHUMT00000324612 | 20.44 | 9.08 | -2.25 | 0.0005 | 0.014 |
| 83 | Up | *NPIPA5* | NM_001277325 | 27.77 | 70.66 | 2.54 | 0.0005 | 0.015 |
| 84 | Up | *HIST1H2BD* | NM_021063 | 5.22 | 19.53 | 3.74 | 0.0006 | 0.015 |
| 85 | Up | *MALAT1* | NR_002819 | 28.33 | 93.91 | 3.32 | 0.0006 | 0.015 |
| 86 | Down | *MIR3914-1* | NR_037477 | 16.15 | 7.32 | -2.21 | 0.0007 | 0.017 |
| 87 | Up | *PLEKHM1P* | OTTHUMT00000445597 | 20.05 | 43.11 | 2.15 | 0.0007 | 0.017 |
| 88 | Up | *BAGE2* | OTTHUMT00000157418 | 21.85 | 82.74 | 3.79 | 0.0008 | 0.018 |
| 89 | Up | *RPL4* | NM_000968 | 22.04 | 47.38 | 2.15 | 0.0008 | 0.018 |
| 90 | Up | *SNORD117* | NR_003140 | 428.09 | 1592.89 | 3.72 | 0.0009 | 0.019 |
| 91 | Up | *DDX5* | NM_004396 | 22.97 | 54.09 | 2.35 | 0.0009 | 0.019 |
| 92 | Up | *RPL21* | NM_000982 | 28.16 | 80.41 | 2.86 | 0.0009 | 0.020 |
| 93 | Up | *LOC100506123* | uc002sxv.4 | 8.79 | 20.29 | 2.31 | 0.0010 | 0.020 |
| 94 | Up | *PCBP1* | NM_006196 | 9.64 | 20.88 | 2.16 | 0.0010 | 0.020 |
| 95 | Up | *NEAT1* | NR_131012 | 31.03 | 184.66 | 5.95 | 0.0010 | 0.020 |
| 96 | Down | *ATP8* | ENST00000361851 | 3015.38 | 1000.35 | -3.01 | 0.0010 | 0.021 |
| 97 | Up | *SNORD11B* | NR_003694 | 6.48 | 22.93 | 3.54 | 0.0011 | 0.021 |
| 98 | Up | *INTS5* | NM_030628 | 8.80 | 22.94 | 2.61 | 0.0011 | 0.021 |
| 99 | Up | *MIR4263* | NR_036230 | 2.96 | 9.75 | 3.29 | 0.0012 | 0.022 |
| 100 | Up | *FTL* | NM_000146 | 396.16 | 1048.04 | 2.65 | 0.0014 | 0.024 |
| 101 | Up | *MIR1254-1* | NR_031655 | 7.41 | 21.52 | 2.90 | 0.0014 | 0.024 |
| 102 | Up | *TRAJ21* | OTTHUMT00000410977 | 3.83 | 20.75 | 5.41 | 0.0014 | 0.024 |
| 103 | Down | *MIR1323* | NR_031568 | 27.77 | 12.35 | -2.25 | 0.0014 | 0.024 |
| 104 | Up | *LOC647264* | XR_915039 | 7.69 | 15.52 | 2.02 | 0.0016 | 0.026 |
| 105 | Up | *UBE2CP5* | AK131243 | 5.44 | 13.31 | 2.45 | 0.0018 | 0.028 |
| 106 | Up | *SNORA74A* | NR_002915 | 68.81 | 236.45 | 3.44 | 0.0018 | 0.028 |
| 107 | Up | *THBS1* | NM_003246 | 16.37 | 40.29 | 2.46 | 0.0019 | 0.028 |
| 108 | Up | *XIST* | NR_001564 | 2.98 | 9.29 | 3.11 | 0.0020 | 0.029 |
| 109 | Up | *TRAJ35* | OTTHUMT00000410963 | 5.75 | 35.38 | 6.16 | 0.0020 | 0.029 |
| 110 | Down | *ND4L* | ENST00000361335 | 1582.49 | 508.62 | -3.11 | 0.0020 | 0.029 |
| 111 | Up | *TMSB10* | NM_021103 | 194.90 | 615.43 | 3.16 | 0.0020 | 0.030 |
| 112 | Down | *RNVU1-6* | NR_104085 | 34.05 | 16.98 | -2.01 | 0.0021 | 0.030 |
| 113 | Up | *LOC100133091* | NR_029411 | 23.29 | 72.99 | 3.13 | 0.0021 | 0.030 |
| 114 | Up | *GANC* | ENST00000318010 | 9.79 | 37.69 | 3.85 | 0.0023 | 0.031 |
| 115 | Up | *A2M* | NM_000014 | 16.66 | 53.56 | 3.21 | 0.0024 | 0.032 |
| 116 | Down | *MIR23A* | NR_029495 | 25.52 | 11.11 | -2.30 | 0.0027 | 0.035 |
| 117 | Down | *ND1* | ENST00000361390 | 569.72 | 180.46 | -3.16 | 0.0028 | 0.035 |
| 118 | Up | *RPL13AP5* | NR_026712 | 217.44 | 577.28 | 2.65 | 0.0030 | 0.036 |
| 119 | Up | *FBXO9* | AK095315 | 85.09 | 197.64 | 2.32 | 0.0031 | 0.036 |
| 120 | Up | *HIST1H2BG* | NM_003518 | 2.26 | 6.26 | 2.77 | 0.0031 | 0.037 |
| 121 | Down | *ND5* | ENST00000361567 | 990.98 | 364.26 | -2.72 | 0.0034 | 0.039 |
| 122 | Up | *SMG1P3* | NR_027155 | 9.66 | 23.15 | 2.40 | 0.0035 | 0.039 |
| 123 | Up | *MT2A* | NM_005953 | 23.28 | 75.07 | 3.23 | 0.0036 | 0.040 |
| 124 | Up | *TMSB4XP4* | OTTHUMT00000054417 | 7.68 | 18.57 | 2.42 | 0.0038 | 0.041 |
| 125 | Up | *SYNGR2* | NM_004710 | 28.57 | 66.77 | 2.34 | 0.0040 | 0.042 |
| 126 | Up | *MT1X* | NM_005952 | 25.53 | 83.54 | 3.27 | 0.0040 | 0.042 |
| 127 | Up | *RPL41* | AF026844 | 1025.16 | 2350.48 | 2.29 | 0.0052 | 0.048 |

**Supplementary table S4.** Quantitative histopathological assessment of the lung tissues from rats exposed to PHMG

| Group | Control | PHMG exposure  (3.20 mg/m^3^) |
| --- | --- | --- |
| Number of animals | 13 | 13 |
|  |  |  |
| Granulomatous inflammation, terminal bronchioles/alveolar duct | (0) | (13) |
| Minimal | 0 | 0 |
| Slight | 0 | 1 |
| Moderate | 0 | 12 |
| Marked | 0 | 0 |
| Severe | 0 | 0 |
| **Mean of Severity Scoring** | **0** | **2.92** |
|  |  |  |
| Alveolar macrophage aggregates | (0) | (13) |
| Minimal | 0 | 0 |
| Slight | 0 | 6 |
| Moderate | 0 | 7 |
| Marked | 0 | 0 |
| Severe | 0 | 0 |
| **Mean of Severity Scoring** | **0** | **2.54** |
|  |  |  |
| Degeneration/regeneration, bronchiolar epithelium | (0) | (13) |
| Minimal | 0 | 0 |
| Slight | 0 | 0 |
| Moderate | 0 | 4 |
| Marked | 0 | 9 |
| Severe | 0 | 0 |
| **Mean of Severity Scoring** | **0** | **3.69** |
|  |  |  |
| Bronchioloalveolar hyperplasia | (0) | (10) |
| Minimal | 0 | 8 |
| Slight | 0 | 2 |
| Moderate | 0 | 0 |
| Marked | 0 | 0 |
| Severe | 0 | 0 |
| **Mean of Severity Scoring** | **0** | **0.92** |
|  |  |  |
| Squamous metaplasia | (0) | (7) |
| Minimal | 0 | 7 |
| Slight | 0 | 0 |
| Moderate | 0 | 0 |
| Marked | 0 | 0 |
| Severe | 0 | 0 |
| **Mean of Severity Scoring** | **0** | **0.54** |
|  |  |  |
| Infiltration, neutrophils | (0) | (2) |
| Minimal | 0 | 2 |
| Slight | 0 | 0 |
| Moderate | 0 | 0 |
| Marked | 0 | 0 |
| Severe | 0 | 0 |
| **Mean of Severity Scoring** | **0** | **0.15** |

Score = 0 for no symptoms; 1 for minimal; 2 for slight; 3 for moderate; 4 for marked; 5 for severe.

**Supplementary table S5.** Differentially expressed annotated genes (fold change ≥ 2, *P_FDR_* < 0.05) in the HD-exposed rat lung tissue

| No | Up-/Down-regulation | Gene | mRNA accession ID | Expression level (average) | | Fold change (Case/Control) | *P* | *P_FDR_* |
| --- | --- | --- | --- | --- | --- | --- | --- | --- |
|  |  |  |  | Case  (n = 10) | Control  (n = 10) |  |  |  |
| 1 | Up | *Serpine1* | NM_012620 | 472.79 | 52.25 | 9.0 | 3.72E-16 | 1.36E-11 |
| 2 | Down | *Tril* | NM_001034010 | 41.84 | 185.52 | -4.4 | 9.85E-16 | 1.81E-11 |
| 3 | Down | *Gsn* | NM_001004080 | 1011.71 | 3033.84 | -3.0 | 3.26E-15 | 2.99E-11 |
| 4 | Up | *C1qb* | NM_019262 | 322.09 | 55.26 | 5.8 | 5.68E-15 | 3.86E-11 |
| 5 | Up | *C1qa* | NM_001008515 | 617.17 | 110.59 | 5.6 | 6.32E-15 | 3.86E-11 |
| 6 | Up | *Slc7a5* | NM_017353 | 384.50 | 83.27 | 4.6 | 7.74E-15 | 4.06E-11 |
| 7 | Up | *C1qc* | NM_001008524 | 266.08 | 59.93 | 4.4 | 1.00E-14 | 4.59E-11 |
| 8 | Up | *Il24* | NM_133311 | 213.35 | 17.38 | 12.3 | 1.66E-14 | 5.10E-11 |
| 9 | Down | *Pgm2l1* | NM_001109454 | 73.16 | 195.96 | -2.7 | 1.81E-14 | 5.10E-11 |
| 10 | Up | *Col8a1* | NM_001107100 | 159.69 | 38.65 | 4.1 | 2.38E-14 | 5.70E-11 |
| 11 | Down | *Clec1a* | NM_001109253 | 114.34 | 272.12 | -2.4 | 2.39E-14 | 5.70E-11 |
| 12 | Up | *Pabpc4* | NM_001100538 | 592.22 | 248.04 | 2.4 | 2.72E-14 | 5.74E-11 |
| 13 | Up | *Prrx1* | NM_153821 | 156.13 | 22.19 | 7.0 | 2.82E-14 | 5.74E-11 |
| 14 | Down | *Gprin3* | XM_002726370 | 41.20 | 128.51 | -3.1 | 3.84E-14 | 7.40E-11 |
| 15 | Up | *Serpinf1* | NM_177927 | 455.48 | 61.55 | 7.4 | 6.08E-14 | 1.06E-10 |
| 16 | Down | *Myzap* | NM_001014211 | 155.76 | 471.25 | -3.0 | 1.24E-13 | 1.63E-10 |
| 17 | Up | *Tgfbi* | NM_053802 | 395.31 | 76.74 | 5.2 | 1.27E-13 | 1.63E-10 |
| 18 | Down | *Esyt3* | XM_001070598 | 44.29 | 93.39 | -2.1 | 1.29E-13 | 1.63E-10 |
| 19 | Up | *F3* | NM_013057 | 2247.87 | 366.19 | 6.1 | 1.31E-13 | 1.63E-10 |
| 20 | Down | *Hykk* | NM_001106823 | 48.35 | 128.83 | -2.7 | 1.42E-13 | 1.64E-10 |
| 21 | Up | *Cdkn1a* | NM_080782 | 476.26 | 115.61 | 4.1 | 1.45E-13 | 1.64E-10 |
| 22 | Down | *Cped1* | XM_001059531 | 140.53 | 351.55 | -2.5 | 1.48E-13 | 1.64E-10 |
| 23 | Up | *Slc7a11* | NM_001107673 | 53.13 | 9.50 | 5.6 | 1.61E-13 | 1.74E-10 |
| 24 | Down | *Tek* | NM_001105737 | 421.18 | 1422.02 | -3.4 | 1.86E-13 | 1.95E-10 |
| 25 | Down | *Mettl24* | NM_001025038 | 48.42 | 120.78 | -2.5 | 2.18E-13 | 2.22E-10 |
| 26 | Up | *Lgals3* | NM_031832 | 1744.36 | 643.03 | 2.7 | 2.27E-13 | 2.25E-10 |
| 27 | Up | *Atp6v1d* | NM_199386 | 513.15 | 214.20 | 2.4 | 2.59E-13 | 2.40E-10 |
| 28 | Up | *Vwa1* | NM_001013938 | 165.88 | 41.43 | 4.0 | 2.63E-13 | 2.40E-10 |
| 29 | Up | *Tnfrsf22* | XM_003753403 | 131.12 | 34.55 | 3.8 | 2.66E-13 | 2.40E-10 |
| 30 | Down | *Gata2* | NM_033442 | 104.60 | 278.61 | -2.7 | 2.68E-13 | 2.40E-10 |
| 31 | Up | *Eda2r* | XM_006227365 | 91.87 | 18.11 | 5.1 | 2.93E-13 | 2.56E-10 |
| 32 | Up | *Ms4a6a* | XM_001075502 | 311.47 | 67.54 | 4.6 | 3.03E-13 | 2.58E-10 |
| 33 | Up | *Prrg4* | NM_001109203 | 159.60 | 32.28 | 4.9 | 3.42E-13 | 2.85E-10 |
| 34 | Down | *Efcc1* | NM_001163921 | 53.11 | 132.06 | -2.5 | 4.32E-13 | 3.37E-10 |
| 35 | Up | *Cxcl6* | NM_022214 | 124.16 | 25.08 | 5.0 | 4.77E-13 | 3.58E-10 |
| 36 | Up | *Fcgr2b* | NM_175756 | 304.68 | 86.84 | 3.5 | 4.97E-13 | 3.65E-10 |
| 37 | Up | *Areg* | NM_017123 | 156.16 | 31.66 | 4.9 | 5.25E-13 | 3.78E-10 |
| 38 | Up | *Bhlhe40* | NM_053328 | 741.45 | 230.74 | 3.2 | 5.61E-13 | 3.96E-10 |
| 39 | Down | *Tmem100* | NM_001017479 | 447.31 | 1743.29 | -3.9 | 6.23E-13 | 4.31E-10 |
| 40 | Up | *Uck2* | ENSRNOT00000076125 | 262.02 | 112.93 | 2.3 | 6.85E-13 | 4.60E-10 |
| 41 | Up | *Cfi* | NM_024157 | 175.57 | 41.75 | 4.2 | 6.90E-13 | 4.60E-10 |
| 42 | Up | *C1qbp* | NM_019259 | 184.92 | 87.84 | 2.1 | 7.20E-13 | 4.71E-10 |
| 43 | Up | *Stk38l* | NM_001083336 | 115.87 | 51.14 | 2.3 | 7.92E-13 | 4.98E-10 |
| 44 | Down | *Plcb4* | NM_024353 | 185.66 | 496.81 | -2.7 | 9.05E-13 | 5.53E-10 |
| 45 | Down | *Tppp3* | NM_001009639 | 261.09 | 1157.76 | -4.4 | 9.90E-13 | 5.85E-10 |
| 46 | Down | *Cdc14a* | NM_001107718 | 171.26 | 352.64 | -2.1 | 1.01E-12 | 5.85E-10 |
| 47 | Down | *Ankrd44* | NM_001191807 | 170.70 | 350.99 | -2.1 | 1.01E-12 | 5.85E-10 |
| 48 | Up | *Cemip* | XM_001063314 | 136.53 | 26.00 | 5.3 | 1.02E-12 | 5.85E-10 |
| 49 | Up | *Ccng1* | NM_012923 | 1092.69 | 314.89 | 3.5 | 1.14E-12 | 6.25E-10 |
| 50 | Down | *Cadps2* | XM_001060172 | 59.09 | 142.64 | -2.4 | 1.15E-12 | 6.25E-10 |
| 51 | Up | *Gja1* | NM_012567 | 778.49 | 306.16 | 2.5 | 1.16E-12 | 6.25E-10 |
| 52 | Down | *Adgrl3* | NM_130822 | 222.80 | 536.19 | -2.4 | 1.33E-12 | 6.98E-10 |
| 53 | Up | *Lgmn* | NM_022226 | 1305.56 | 546.55 | 2.4 | 1.47E-12 | 7.47E-10 |
| 54 | Up | *Mafb* | NM_019316 | 142.95 | 46.23 | 3.1 | 1.51E-12 | 7.47E-10 |
| 55 | Down | *Adgrf5* | NM_139110 | 965.93 | 2221.03 | -2.3 | 1.53E-12 | 7.48E-10 |
| 56 | Up | *Rhoc* | NM_001106461 | 588.77 | 272.98 | 2.2 | 1.73E-12 | 8.34E-10 |
| 57 | Up | *Dap* | NM_022526 | 637.30 | 315.17 | 2.0 | 1.92E-12 | 9.12E-10 |
| 58 | Up | *Ntrk2* | NM_001163168 | 240.02 | 35.06 | 6.8 | 2.04E-12 | 9.59E-10 |
| 59 | Down | *Bmpr2* | NM_080407 | 1090.70 | 2238.48 | -2.1 | 2.16E-12 | 9.98E-10 |
| 60 | Down | *Arhgap31* | NM_001105879 | 267.95 | 585.56 | -2.2 | 2.19E-12 | 9.98E-10 |
| 61 | Up | *Inhba* | NM_017128 | 147.81 | 26.75 | 5.5 | 2.24E-12 | 9.98E-10 |
| 62 | Down | *Fzd8* | NM_001044251 | 58.94 | 128.46 | -2.2 | 2.26E-12 | 9.98E-10 |
| 63 | Down | *Aoc3* | NM_031582 | 111.42 | 417.95 | -3.8 | 2.32E-12 | 1.01E-09 |
| 64 | Down | *Hpgd* | NM_024390 | 68.70 | 451.61 | -6.6 | 2.60E-12 | 1.12E-09 |
| 65 | Up | *Mmp14* | NM_031056 | 604.50 | 206.28 | 2.9 | 3.03E-12 | 1.28E-09 |
| 66 | Down | *Clec14a* | NM_001014077 | 252.35 | 1126.38 | -4.5 | 3.05E-12 | 1.28E-09 |
| 67 | Up | *Nupr1* | NM_053611 | 463.39 | 137.48 | 3.4 | 3.06E-12 | 1.28E-09 |
| 68 | Up | *Tubb6* | NM_001025675 | 281.87 | 82.34 | 3.4 | 3.16E-12 | 1.30E-09 |
| 69 | Up | *Myo5a* | NM_022178 | 355.82 | 137.51 | 2.6 | 3.20E-12 | 1.30E-09 |
| 70 | Down | *Epb4.1l5* | NM_001012023 | 159.48 | 374.92 | -2.4 | 3.41E-12 | 1.37E-09 |
| 71 | Down | *Cyp4b1* | NM_016999 | 105.90 | 763.10 | -7.2 | 3.64E-12 | 1.44E-09 |
| 72 | Up | *B4galt5* | NM_001108608 | 475.96 | 183.71 | 2.6 | 3.76E-12 | 1.47E-09 |
| 73 | Down | *Mme* | NM_012608 | 225.50 | 681.24 | -3.0 | 3.94E-12 | 1.52E-09 |
| 74 | Down | *Plekhh2* | NM_001191770 | 78.58 | 157.74 | -2.0 | 4.08E-12 | 1.56E-09 |
| 75 | Down | *Acoxl* | NM_001106508 | 32.25 | 201.93 | -6.3 | 4.49E-12 | 1.70E-09 |
| 76 | Down | *Nrgn* | NM_024140 | 44.63 | 90.20 | -2.0 | 5.08E-12 | 1.90E-09 |
| 77 | Up | *Tmsb10* | NM_021261 | 179.53 | 85.08 | 2.1 | 5.22E-12 | 1.92E-09 |
| 78 | Up | *Gsto1* | NM_001007602 | 158.86 | 75.14 | 2.1 | 5.24E-12 | 1.92E-09 |
| 79 | Down | *Fam122b* | NM_001166586 | 39.30 | 85.88 | -2.2 | 5.38E-12 | 1.95E-09 |
| 80 | Down | *Rarb* | NM_031529 | 53.54 | 123.35 | -2.3 | 5.42E-12 | 1.95E-09 |
| 81 | Down | *Npr3* | NM_012868 | 61.09 | 287.82 | -4.7 | 5.55E-12 | 1.98E-09 |
| 82 | Up | *Gars* | NM_001271139 | 836.77 | 404.92 | 2.1 | 5.69E-12 | 2.01E-09 |
| 83 | Down | *Lyve1* | NM_001106286 | 192.38 | 769.98 | -4.0 | 5.85E-12 | 2.04E-09 |
| 84 | Down | *Cav1* | NM_031556 | 979.06 | 2664.07 | -2.7 | 6.19E-12 | 2.10E-09 |
| 85 | Up | *Efemp2* | NM_001005907 | 216.79 | 100.51 | 2.2 | 6.27E-12 | 2.11E-09 |
| 86 | Up | *Col18a1* | NM_053489 | 95.48 | 41.68 | 2.3 | 6.38E-12 | 2.12E-09 |
| 87 | Up | *Wisp1* | NM_031716 | 93.30 | 19.22 | 4.9 | 6.43E-12 | 2.12E-09 |
| 88 | Up | *Capg* | NM_001013086 | 478.01 | 161.37 | 3.0 | 7.25E-12 | 2.31E-09 |
| 89 | Up | *Krt17* | NM_212545 | 1146.25 | 26.97 | 42.5 | 7.36E-12 | 2.32E-09 |
| 90 | Up | *Tcp11l1* | NM_001109202 | 157.63 | 77.96 | 2.0 | 8.31E-12 | 2.52E-09 |
| 91 | Down | *Rarb* | NM_031529 | 60.01 | 143.25 | -2.4 | 8.49E-12 | 2.55E-09 |
| 92 | Down | *Fam13a* | NM_001100862 | 37.87 | 101.05 | -2.7 | 9.41E-12 | 2.76E-09 |
| 93 | Up | *Asns* | NM_013079 | 182.37 | 86.52 | 2.1 | 9.63E-12 | 2.80E-09 |
| 94 | Up | *Micall2* | XM_006221279 | 53.24 | 23.39 | 2.3 | 9.72E-12 | 2.81E-09 |
| 95 | Up | *Tgfb3* | NM_013174 | 171.23 | 63.84 | 2.7 | 1.01E-11 | 2.89E-09 |
| 96 | Down | *Ldb2* | NM_001106009 | 153.54 | 422.84 | -2.8 | 1.08E-11 | 3.02E-09 |
| 97 | Up | *Osmr* | NM_001005384 | 422.11 | 113.45 | 3.7 | 1.08E-11 | 3.02E-09 |
| 98 | Up | *Tgfb2* | NM_031131 | 644.55 | 261.04 | 2.5 | 1.24E-11 | 3.35E-09 |
| 99 | Down | *Mettl7a* | NM_001037355 | 204.41 | 620.61 | -3.0 | 1.39E-11 | 3.63E-09 |
| 100 | Up | *Ctsk* | NM_031560 | 155.11 | 40.81 | 3.8 | 1.45E-11 | 3.73E-09 |
| 101 | Up | *Acta1* | NM_019212 | 269.83 | 39.19 | 6.9 | 1.56E-11 | 4.01E-09 |
| 102 | Up | *Ninj1* | NM_012867 | 153.99 | 68.53 | 2.2 | 1.79E-11 | 4.46E-09 |
| 103 | Down | *Thrb* | NM_001270854 | 56.86 | 117.31 | -2.1 | 1.82E-11 | 4.51E-09 |
| 104 | Down | *Raver2* | NM_001191867 | 143.81 | 352.77 | -2.5 | 1.96E-11 | 4.82E-09 |
| 105 | Up | *Slc7a1* | NM_013111 | 205.38 | 73.02 | 2.8 | 2.01E-11 | 4.88E-09 |
| 106 | Up | *Clec10a* | NM_022393 | 171.16 | 39.29 | 4.4 | 2.06E-11 | 4.88E-09 |
| 107 | Up | *Litaf* | NM_001105735 | 638.36 | 318.56 | 2.0 | 2.06E-11 | 4.88E-09 |
| 108 | Down | *LOC100361083* | NM_001291383 | 169.53 | 387.76 | -2.3 | 2.10E-11 | 4.92E-09 |
| 109 | Down | *She* | XM_001062249 | 104.75 | 225.07 | -2.1 | 2.11E-11 | 4.92E-09 |
| 110 | Up | *Fst* | NM_012561 | 94.72 | 35.10 | 2.7 | 2.14E-11 | 4.94E-09 |
| 111 | Down | *Gsta1* | NM_031509 | 106.28 | 217.84 | -2.0 | 2.30E-11 | 5.22E-09 |
| 112 | Up | *Trh* | NM_013046 | 82.62 | 16.53 | 5.0 | 2.31E-11 | 5.22E-09 |
| 113 | Up | *C3ar1* | NM_032060 | 140.18 | 34.65 | 4.0 | 2.35E-11 | 5.29E-09 |
| 114 | Up | *Bid* | NM_022684 | 147.87 | 69.99 | 2.1 | 2.37E-11 | 5.29E-09 |
| 115 | Down | *Sema3g* | NM_001100882 | 66.30 | 278.66 | -4.2 | 2.40E-11 | 5.30E-09 |
| 116 | Down | *G0s2* | NM_001009632 | 71.10 | 179.90 | -2.5 | 2.44E-11 | 5.36E-09 |
| 117 | Up | *Sh3pxd2b* | XM_006220627 | 185.75 | 71.76 | 2.6 | 2.76E-11 | 6.04E-09 |
| 118 | Up | *Csf2rb* | NM_133555 | 194.22 | 77.98 | 2.5 | 2.78E-11 | 6.04E-09 |
| 119 | Up | *Trem2* | NM_001106884 | 294.67 | 66.76 | 4.4 | 2.85E-11 | 6.15E-09 |
| 120 | Up | *Ifi27l2b* | NM_206846 | 316.59 | 87.31 | 3.6 | 3.00E-11 | 6.35E-09 |
| 121 | Down | *Thra* | NM_001017960 | 167.06 | 350.24 | -2.1 | 3.06E-11 | 6.45E-09 |
| 122 | Up | *Itgam* | NM_012711 | 172.53 | 35.67 | 4.8 | 3.11E-11 | 6.51E-09 |
| 123 | Up | *Fat1* | NM_031819 | 935.49 | 458.64 | 2.0 | 3.20E-11 | 6.64E-09 |
| 124 | Up | *Pld1* | NM_030992 | 166.52 | 77.06 | 2.2 | 3.29E-11 | 6.72E-09 |
| 125 | Up | *Fn1* | NM_019143 | 3572.71 | 962.67 | 3.7 | 3.30E-11 | 6.72E-09 |
| 126 | Up | *Pkm* | NM_053297 | 155.52 | 68.91 | 2.3 | 3.40E-11 | 6.84E-09 |
| 127 | Down | *Kank4* | NM_001107947 | 55.03 | 188.31 | -3.4 | 3.43E-11 | 6.89E-09 |
| 128 | Down | *Fam49a* | NM_001106718 | 117.30 | 239.05 | -2.0 | 3.61E-11 | 7.14E-09 |
| 129 | Up | *Aif1l* | NM_001108578 | 78.75 | 31.07 | 2.5 | 3.68E-11 | 7.14E-09 |
| 130 | Up | *Ppic* | NM_001004215 | 737.40 | 325.34 | 2.3 | 3.71E-11 | 7.16E-09 |
| 131 | Down | *Cct6b* | NM_001014228 | 11.13 | 31.89 | -2.9 | 4.05E-11 | 7.75E-09 |
| 132 | Up | *Tiam1* | NM_001100558 | 107.22 | 40.44 | 2.7 | 4.13E-11 | 7.78E-09 |
| 133 | Up | *Aen* | NM_001108487 | 95.51 | 33.22 | 2.9 | 4.32E-11 | 7.98E-09 |
| 134 | Down | *Fam174b* | XM_002728766 | 105.98 | 235.68 | -2.2 | 4.33E-11 | 7.98E-09 |
| 135 | Down | *Gfra2* | NM_012750 | 72.22 | 279.73 | -3.9 | 4.54E-11 | 8.25E-09 |
| 136 | Down | *Col4a4* | NM_001008332 | 118.93 | 248.30 | -2.1 | 4.71E-11 | 8.44E-09 |
| 137 | Down | *Synm* | NM_001134858 | 72.05 | 164.37 | -2.3 | 4.71E-11 | 8.44E-09 |
| 138 | Up | *Cldn4* | NM_001012022 | 167.87 | 35.17 | 4.8 | 4.75E-11 | 8.45E-09 |
| 139 | Up | *Fndc1* | NM_001038615 | 127.47 | 28.14 | 4.5 | 5.19E-11 | 9.11E-09 |
| 140 | Up | *Folr2* | NM_001106283 | 164.50 | 44.86 | 3.7 | 5.39E-11 | 9.37E-09 |
| 141 | Down | *Fam183b* | XM_006220684 | 53.85 | 247.12 | -4.6 | 5.43E-11 | 9.39E-09 |
| 142 | Up | *Tpm2* | NM_001024345 | 132.30 | 44.99 | 2.9 | 5.58E-11 | 9.61E-09 |
| 143 | Down | *Pla2g2d* | NM_001013428 | 186.33 | 620.92 | -3.3 | 5.71E-11 | 9.75E-09 |
| 144 | Up | *Clec4a3* | NM_001005891 | 265.45 | 52.80 | 5.0 | 5.87E-11 | 9.92E-09 |
| 145 | Up | *Myl9* | XM_006235401 | 330.72 | 145.61 | 2.3 | 6.06E-11 | 1.02E-08 |
| 146 | Down | *Tmem64* | XM_003749909 | 152.14 | 327.93 | -2.2 | 6.54E-11 | 1.08E-08 |
| 147 | Up | *Tnfrsf12a* | NM_181086 | 230.69 | 60.40 | 3.8 | 6.67E-11 | 1.09E-08 |
| 148 | Up | *Krt7* | XM_003750407 | 1174.19 | 456.55 | 2.6 | 6.77E-11 | 1.10E-08 |
| 149 | Down | *Cav2* | NM_131914 | 611.10 | 1290.09 | -2.1 | 6.79E-11 | 1.10E-08 |
| 150 | Up | *Clu* | NM_053021 | 2444.48 | 1068.37 | 2.3 | 6.85E-11 | 1.10E-08 |
| 151 | Down | *Zfp711* | XM_002727619 | 15.58 | 34.29 | -2.2 | 7.01E-11 | 1.12E-08 |
| 152 | Up | *Apoe* | NM_001270681 | 1559.41 | 485.24 | 3.2 | 7.43E-11 | 1.18E-08 |
| 153 | Up | *Mgmt* | NM_012861 | 162.53 | 72.56 | 2.2 | 7.44E-11 | 1.18E-08 |
| 154 | Down | *Cav2* | NM_131914 | 582.52 | 1227.07 | -2.1 | 7.52E-11 | 1.18E-08 |
| 155 | Up | *Mcm4* | NM_033651 | 172.30 | 85.79 | 2.0 | 7.84E-11 | 1.23E-08 |
| 156 | Up | *Arhgap11a* | NM_001168524 | 92.78 | 41.53 | 2.2 | 8.03E-11 | 1.25E-08 |
| 157 | Up | *Pdgfc* | NM_031317 | 87.45 | 35.27 | 2.5 | 8.06E-11 | 1.25E-08 |
| 158 | Up | *Ifi30* | NM_001030026 | 334.93 | 156.53 | 2.1 | 8.99E-11 | 1.35E-08 |
| 159 | Up | *Lama5* | NM_001191609 | 390.19 | 183.52 | 2.1 | 9.56E-11 | 1.42E-08 |
| 160 | Up | *Actn1* | NM_031005 | 1013.67 | 412.39 | 2.5 | 9.85E-11 | 1.46E-08 |
| 161 | Up | *Col1a1* | NM_053304 | 1255.71 | 303.02 | 4.1 | 1.01E-10 | 1.48E-08 |
| 162 | Up | *Ctss* | NM_017320 | 686.08 | 283.89 | 2.4 | 1.03E-10 | 1.50E-08 |
| 163 | Down | *Kcnj13* | NM_053608 | 19.76 | 51.31 | -2.6 | 1.14E-10 | 1.63E-08 |
| 164 | Down | *Scube2* | XM_003748970 | 77.49 | 411.65 | -5.3 | 1.17E-10 | 1.64E-08 |
| 165 | Down | *Bank1* | NM_001047918 | 32.09 | 192.72 | -6.0 | 1.19E-10 | 1.67E-08 |
| 166 | Down | *LOC291863* | NM_001013889 | 130.89 | 594.24 | -4.5 | 1.23E-10 | 1.72E-08 |
| 167 | Down | *Cldn5* | NM_031701 | 366.80 | 888.45 | -2.4 | 1.26E-10 | 1.74E-08 |
| 168 | Up | *Spp1* | NM_012881 | 1113.00 | 39.23 | 28.4 | 1.29E-10 | 1.78E-08 |
| 169 | Down | *Arhgap20* | NM_213629 | 53.73 | 115.55 | -2.2 | 1.29E-10 | 1.78E-08 |
| 170 | Down | *Mapt* | NM_017212 | 68.94 | 161.32 | -2.3 | 1.30E-10 | 1.78E-08 |
| 171 | Down | *Smad6* | NM_001109002 | 280.18 | 831.57 | -3.0 | 1.30E-10 | 1.78E-08 |
| 172 | Down | *Ptprb* | XM_003754286 | 462.40 | 1171.56 | -2.5 | 1.38E-10 | 1.87E-08 |
| 173 | Down | *Rnf125* | NM_001108424 | 135.95 | 299.18 | -2.2 | 1.44E-10 | 1.95E-08 |
| 174 | Down | *Col4a3* | NM_001135759 | 115.32 | 260.56 | -2.3 | 1.45E-10 | 1.95E-08 |
| 175 | Down | *Lifr* | NM_031048 | 393.35 | 1459.12 | -3.7 | 1.46E-10 | 1.95E-08 |
| 176 | Up | *Rrm2* | NM_001025740 | 80.52 | 23.11 | 3.5 | 1.48E-10 | 1.96E-08 |
| 177 | Up | *Cd86* | NM_020081 | 110.27 | 38.34 | 2.9 | 1.49E-10 | 1.97E-08 |
| 178 | Down | *Slc15a2* | NM_031672 | 33.83 | 117.12 | -3.5 | 1.70E-10 | 2.17E-08 |
| 179 | Up | *Pcolce* | NM_019237 | 193.99 | 78.57 | 2.5 | 1.75E-10 | 2.22E-08 |
| 180 | Up | *Odc1* | NM_001302083 | 446.12 | 208.97 | 2.1 | 1.76E-10 | 2.22E-08 |
| 181 | Down | *Tspan7* | NM_001108815 | 1032.80 | 2734.72 | -2.6 | 1.78E-10 | 2.22E-08 |
| 182 | Up | *Amd1* | NM_031011 | 373.28 | 178.29 | 2.1 | 1.83E-10 | 2.26E-08 |
| 183 | Down | *S1pr1* | NM_017301 | 301.81 | 662.54 | -2.2 | 1.89E-10 | 2.33E-08 |
| 184 | Up | *Rab32* | NM_001108902 | 155.14 | 74.07 | 2.1 | 1.95E-10 | 2.39E-08 |
| 185 | Down | *Esm1* | NM_022604 | 26.30 | 153.46 | -5.8 | 2.04E-10 | 2.47E-08 |
| 186 | Down | *Rasgef1a* | XM_001077186 | 34.92 | 90.11 | -2.6 | 2.06E-10 | 2.48E-08 |
| 187 | Up | *Slc26a4* | NM_019214 | 242.78 | 26.60 | 9.1 | 2.21E-10 | 2.66E-08 |
| 188 | Up | *Slc41a2* | NM_001108742 | 138.60 | 49.55 | 2.8 | 2.25E-10 | 2.69E-08 |
| 189 | Down | *Eml6* | XM_008767134 | 31.30 | 67.93 | -2.2 | 2.28E-10 | 2.70E-08 |
| 190 | Up | *Sel1l3* | XM_006251033 | 84.43 | 31.42 | 2.7 | 2.29E-10 | 2.70E-08 |
| 191 | Up | *Ctgf* | NM_022266 | 803.06 | 252.03 | 3.2 | 2.40E-10 | 2.80E-08 |
| 192 | Down | *Lpar3* | NM_023969 | 36.59 | 124.27 | -3.4 | 2.41E-10 | 2.81E-08 |
| 193 | Up | *Galc* | NM_001005888 | 137.74 | 65.88 | 2.1 | 2.47E-10 | 2.85E-08 |
| 194 | Down | *Kit* | NM_022264 | 146.68 | 402.10 | -2.7 | 2.51E-10 | 2.89E-08 |
| 195 | Up | *Glrx3* | NM_032614 | 377.10 | 185.01 | 2.0 | 2.73E-10 | 3.09E-08 |
| 196 | Down | *Tet1* | XM_006223880 | 16.30 | 38.25 | -2.3 | 2.73E-10 | 3.09E-08 |
| 197 | Up | *Lgals3bp* | NM_139096 | 817.68 | 358.54 | 2.3 | 2.83E-10 | 3.16E-08 |
| 198 | Down | *Erbb4* | NM_021687 | 15.62 | 43.29 | -2.8 | 2.84E-10 | 3.16E-08 |
| 199 | Up | *Clec4a1* | NM_001005890 | 221.73 | 56.75 | 3.9 | 2.84E-10 | 3.16E-08 |
| 200 | Down | *Rgs7* | NM_019343 | 19.70 | 61.67 | -3.1 | 2.84E-10 | 3.16E-08 |
| 201 | Down | *Adgrl1* | NM_022962 | 50.86 | 101.88 | -2.0 | 3.17E-10 | 3.44E-08 |
| 202 | Up | *Plk1* | NM_017100 | 122.50 | 53.76 | 2.3 | 3.48E-10 | 3.70E-08 |
| 203 | Down | *Slc39a2* | NM_001107260 | 48.79 | 166.84 | -3.4 | 3.49E-10 | 3.70E-08 |
| 204 | Up | *Rab11fip5* | XM_003753914 | 147.64 | 73.14 | 2.0 | 3.57E-10 | 3.76E-08 |
| 205 | Up | *Dlgap5* | NM_001135802 | 55.80 | 27.83 | 2.0 | 3.64E-10 | 3.82E-08 |
| 206 | Down | *Atp2a3* | NM_012914 | 51.63 | 159.18 | -3.1 | 3.70E-10 | 3.88E-08 |
| 207 | Down | *Klf8* | XM_008758380 | 12.42 | 28.26 | -2.3 | 3.80E-10 | 3.96E-08 |
| 208 | Up | *Krt14* | NM_001008751 | 1391.09 | 34.95 | 39.8 | 3.88E-10 | 4.00E-08 |
| 209 | Up | *Aif1* | NM_017196 | 154.29 | 61.14 | 2.5 | 3.91E-10 | 4.01E-08 |
| 210 | Up | *Rrm2* | ENSRNOT00000091551 | 198.85 | 55.38 | 3.6 | 3.91E-10 | 4.01E-08 |
| 211 | Down | *Mmp16* | NM_080776 | 37.11 | 94.21 | -2.5 | 3.99E-10 | 4.08E-08 |
| 212 | Up | *Lilrb4* | NM_001013894 | 462.25 | 100.96 | 4.6 | 4.08E-10 | 4.16E-08 |
| 213 | Up | *Cd200r1* | NM_023953 | 55.50 | 22.79 | 2.4 | 4.35E-10 | 4.37E-08 |
| 214 | Up | *Timp1* | NM_053819 | 788.11 | 190.38 | 4.1 | 4.46E-10 | 4.45E-08 |
| 215 | Up | *Mthfd2* | NM_001109398 | 100.98 | 36.76 | 2.7 | 4.52E-10 | 4.47E-08 |
| 216 | Down | *Ramp2* | NM_031646 | 183.45 | 483.17 | -2.6 | 4.59E-10 | 4.51E-08 |
| 217 | Down | *Abcd2* | NM_033352 | 20.76 | 83.54 | -4.0 | 4.78E-10 | 4.64E-08 |
| 218 | Down | *Sema3a* | NM_017310 | 61.87 | 137.09 | -2.2 | 4.82E-10 | 4.66E-08 |
| 219 | Up | *Depdc7* | NM_001029916 | 43.52 | 17.09 | 2.5 | 4.91E-10 | 4.72E-08 |
| 220 | Down | *Vipr1* | NM_012685 | 85.29 | 362.45 | -4.2 | 5.05E-10 | 4.83E-08 |
| 221 | Down | *Acsm5* | NM_001014162 | 42.20 | 103.82 | -2.5 | 5.12E-10 | 4.89E-08 |
| 222 | Up | *Fxyd5* | NM_001270688 | 417.99 | 191.17 | 2.2 | 5.49E-10 | 5.15E-08 |
| 223 | Down | *Col6a4* | NM_001271182 | 20.86 | 48.53 | -2.3 | 5.52E-10 | 5.15E-08 |
| 224 | Down | *Tspan12* | NM_001015026 | 486.20 | 979.40 | -2.0 | 5.73E-10 | 5.30E-08 |
| 225 | Up | *Igfbp4* | NM_001004274 | 401.84 | 156.58 | 2.6 | 5.74E-10 | 5.30E-08 |
| 226 | Down | *Dnali1* | NM_001031647 | 34.59 | 84.95 | -2.5 | 6.07E-10 | 5.59E-08 |
| 227 | Down | *Chic1* | XM_001055438 | 25.29 | 60.56 | -2.4 | 6.08E-10 | 5.59E-08 |
| 228 | Up | *Vcan* | NM_001170558 | 324.88 | 54.05 | 6.0 | 6.13E-10 | 5.61E-08 |
| 229 | Up | *Mmp2* | NM_031054 | 1134.02 | 398.26 | 2.8 | 6.16E-10 | 5.61E-08 |
| 230 | Up | *Serpine2* | NM_019197 | 214.22 | 62.25 | 3.4 | 6.18E-10 | 5.61E-08 |
| 231 | Up | *Ppp1r14b* | NM_172045 | 355.30 | 168.97 | 2.1 | 6.20E-10 | 5.61E-08 |
| 232 | Up | *Malt1* | XM_001060526 | 362.82 | 128.10 | 2.8 | 6.41E-10 | 5.76E-08 |
| 233 | Down | *Cfap43* | XM_006231588 | 25.29 | 120.92 | -4.8 | 6.59E-10 | 5.89E-08 |
| 234 | Down | *Kcne2* | NM_133603 | 56.00 | 141.14 | -2.5 | 7.06E-10 | 6.27E-08 |
| 235 | Up | *Mybl1* | NM_001106632 | 81.99 | 13.31 | 6.2 | 7.16E-10 | 6.33E-08 |
| 236 | Down | *Alox5* | NM_012822 | 102.89 | 223.08 | -2.2 | 7.19E-10 | 6.34E-08 |
| 237 | Up | *Ripk3* | NM_139342 | 66.29 | 30.66 | 2.2 | 7.22E-10 | 6.35E-08 |
| 238 | Up | *Metrnl* | NM_001014104 | 135.32 | 64.84 | 2.1 | 7.32E-10 | 6.42E-08 |
| 239 | Up | *Bcat1* | NM_017253 | 77.28 | 27.42 | 2.8 | 7.35E-10 | 6.44E-08 |
| 240 | Up | *Siglec1* | NM_001107777 | 177.30 | 45.50 | 3.9 | 7.49E-10 | 6.54E-08 |
| 241 | Down | *Omd* | NM_031817 | 10.36 | 55.81 | -5.4 | 7.95E-10 | 6.85E-08 |
| 242 | Up | *Hk2* | NM_012735 | 145.07 | 62.68 | 2.3 | 8.13E-10 | 6.94E-08 |
| 243 | Down | *Sdr9c7* | XM_006226054 | 33.58 | 73.03 | -2.2 | 8.98E-10 | 7.53E-08 |
| 244 | Up | *Ska3* | NM_001108379 | 52.91 | 23.76 | 2.2 | 9.01E-10 | 7.53E-08 |
| 245 | Down | *Snx19* | NM_001108131 | 167.14 | 391.94 | -2.3 | 9.13E-10 | 7.61E-08 |
| 246 | Up | *Prss22* | NM_001106984 | 107.64 | 52.39 | 2.1 | 9.28E-10 | 7.70E-08 |
| 247 | Down | *Nrxn1* | NM_021767 | 25.32 | 55.25 | -2.2 | 9.34E-10 | 7.72E-08 |
| 248 | Up | *Pld4* | NM_001126288 | 171.12 | 64.96 | 2.6 | 9.39E-10 | 7.74E-08 |
| 249 | Down | *Osbpl6* | NM_001107735 | 94.70 | 301.55 | -3.2 | 9.41E-10 | 7.74E-08 |
| 250 | Down | *Fam63b* | XM_001054973 | 195.83 | 392.15 | -2.0 | 9.74E-10 | 7.97E-08 |
| 251 | Down | *Sh3tc2* | XM_001059165 | 30.92 | 72.03 | -2.3 | 9.90E-10 | 8.07E-08 |
| 252 | Down | *Slc5a1* | NM_013033 | 39.20 | 149.53 | -3.8 | 1.03E-09 | 8.29E-08 |
| 253 | Down | *Plpp3* | NM_138905 | 206.72 | 646.55 | -3.1 | 1.05E-09 | 8.43E-08 |
| 254 | Down | *Magi1* | NM_001030045 | 110.16 | 230.71 | -2.1 | 1.06E-09 | 8.47E-08 |
| 255 | Up | *Sfrp1* | NM_001276712 | 106.49 | 41.25 | 2.6 | 1.08E-09 | 8.59E-08 |
| 256 | Up | *Thbs2* | NM_001169138 | 197.14 | 29.61 | 6.7 | 1.08E-09 | 8.59E-08 |
| 257 | Down | *Pacrg* | NM_001077677 | 41.27 | 86.51 | -2.1 | 1.09E-09 | 8.61E-08 |
| 258 | Down | *Dll4* | NM_001107760 | 89.14 | 433.01 | -4.9 | 1.09E-09 | 8.64E-08 |
| 259 | Up | *Fabp4* | NM_053365 | 215.87 | 52.61 | 4.1 | 1.11E-09 | 8.71E-08 |
| 260 | Up | *Irf7* | NM_001033691 | 309.29 | 101.55 | 3.0 | 1.11E-09 | 8.71E-08 |
| 261 | Up | *Des* | NM_022531 | 654.69 | 195.37 | 3.4 | 1.12E-09 | 8.71E-08 |
| 262 | Up | *Prdx5* | NM_053610 | 360.88 | 179.24 | 2.0 | 1.12E-09 | 8.71E-08 |
| 263 | Down | *Lrmp* | XM_006225121 | 72.33 | 155.21 | -2.1 | 1.13E-09 | 8.81E-08 |
| 264 | Up | *Blnk* | NM_001025767 | 63.15 | 27.24 | 2.3 | 1.14E-09 | 8.86E-08 |
| 265 | Up | *Phlda3* | NM_001012206 | 186.34 | 85.37 | 2.2 | 1.15E-09 | 8.90E-08 |
| 266 | Down | *Pde3a* | NM_017337 | 109.20 | 258.26 | -2.4 | 1.16E-09 | 8.94E-08 |
| 267 | Down | *Kcnmb2* | NM_176861 | 13.71 | 67.74 | -4.9 | 1.17E-09 | 8.97E-08 |
| 268 | Up | *Gss* | NM_012962 | 146.80 | 53.93 | 2.7 | 1.17E-09 | 8.97E-08 |
| 269 | Up | *Hexb* | NM_001011946 | 333.35 | 162.66 | 2.0 | 1.18E-09 | 8.99E-08 |
| 270 | Up | *Ptafr* | NM_053321 | 82.62 | 37.92 | 2.2 | 1.25E-09 | 9.45E-08 |
| 271 | Up | *Mcm6* | NM_017287 | 109.51 | 53.33 | 2.1 | 1.29E-09 | 9.70E-08 |
| 272 | Up | *Slc37a2* | NM_001191994 | 88.52 | 37.00 | 2.4 | 1.29E-09 | 9.70E-08 |
| 273 | Down | *Stard9* | XM_001080099 | 91.84 | 208.38 | -2.3 | 1.29E-09 | 9.70E-08 |
| 274 | Up | *Gpnmb* | NM_133298 | 2129.82 | 736.82 | 2.9 | 1.30E-09 | 9.74E-08 |
| 275 | Up | *Cpxm2* | NM_001106306 | 306.41 | 74.70 | 4.1 | 1.31E-09 | 9.78E-08 |
| 276 | Up | *Ccna2* | NM_053702 | 93.36 | 33.53 | 2.8 | 1.33E-09 | 9.88E-08 |
| 277 | Up | *Ccr5* | NM_053960 | 77.92 | 23.79 | 3.3 | 1.35E-09 | 1.01E-07 |
| 278 | Up | *Rarres1* | NM_001014790 | 158.04 | 36.75 | 4.3 | 1.38E-09 | 1.02E-07 |
| 279 | Down | *Galnt18* | NM_001079884 | 123.65 | 275.79 | -2.2 | 1.41E-09 | 1.04E-07 |
| 280 | Up | *Cdkn3* | NM_001106028 | 78.18 | 35.30 | 2.2 | 1.42E-09 | 1.04E-07 |
| 281 | Up | *Cp* | NM_001270961 | 726.44 | 350.15 | 2.1 | 1.42E-09 | 1.04E-07 |
| 282 | Up | *Mboat1* | NM_001109120 | 398.11 | 198.99 | 2.0 | 1.45E-09 | 1.06E-07 |
| 283 | Up | *Htatip2* | NM_001106263 | 157.77 | 57.77 | 2.7 | 1.47E-09 | 1.07E-07 |
| 284 | Up | *Rbp2* | NM_012640 | 133.79 | 21.47 | 6.2 | 1.50E-09 | 1.08E-07 |
| 285 | Up | *Parm1* | NM_173114 | 156.49 | 69.32 | 2.3 | 1.51E-09 | 1.09E-07 |
| 286 | Down | *Spns2* | NM_001144991 | 126.88 | 258.59 | -2.0 | 1.54E-09 | 1.10E-07 |
| 287 | Down | *Tet1* | XM_006223880 | 42.71 | 140.20 | -3.3 | 1.54E-09 | 1.10E-07 |
| 288 | Up | *Nipsnap3b* | NM_001009422 | 72.12 | 29.61 | 2.4 | 1.57E-09 | 1.12E-07 |
| 289 | Down | *Slc29a1* | NM_031684 | 98.47 | 335.00 | -3.4 | 1.63E-09 | 1.15E-07 |
| 290 | Down | *Gulp1* | NM_001013171 | 74.52 | 162.82 | -2.2 | 1.64E-09 | 1.16E-07 |
| 291 | Down | *RGD1561931* | NM_001313815 | 54.34 | 118.48 | -2.2 | 1.65E-09 | 1.17E-07 |
| 292 | Down | *Sult1a1* | NM_031834 | 335.96 | 800.04 | -2.4 | 1.67E-09 | 1.17E-07 |
| 293 | Up | *P2ry6* | NM_057124 | 84.63 | 33.85 | 2.5 | 1.68E-09 | 1.18E-07 |
| 294 | Up | *Fcgr1a* | NM_001100836 | 292.17 | 120.95 | 2.4 | 1.70E-09 | 1.18E-07 |
| 295 | Up | *Serinc2* | NM_001031656 | 302.04 | 86.77 | 3.5 | 1.87E-09 | 1.27E-07 |
| 296 | Down | *Cyth3* | NM_053912 | 344.48 | 755.43 | -2.2 | 1.91E-09 | 1.29E-07 |
| 297 | Down | *Limch1* | NM_001191678 | 518.26 | 1411.35 | -2.7 | 2.02E-09 | 1.35E-07 |
| 298 | Down | *Runx1t1* | NM_001108657 | 46.70 | 128.08 | -2.7 | 2.06E-09 | 1.37E-07 |
| 299 | Up | *Aoc1* | NM_022935 | 103.72 | 26.89 | 3.9 | 2.10E-09 | 1.39E-07 |
| 300 | Up | *Smpdl3a* | NM_001005539 | 824.12 | 339.18 | 2.4 | 2.15E-09 | 1.41E-07 |
| 301 | Up | *Trim16* | NM_001135033 | 111.18 | 44.92 | 2.5 | 2.16E-09 | 1.41E-07 |
| 302 | Up | *Dse* | NM_001108933 | 187.27 | 90.00 | 2.1 | 2.22E-09 | 1.45E-07 |
| 303 | Down | *Cers4* | NM_001107117 | 96.35 | 205.54 | -2.1 | 2.23E-09 | 1.45E-07 |
| 304 | Up | *Tm4sf1* | NM_001106434 | 168.87 | 70.83 | 2.4 | 2.24E-09 | 1.45E-07 |
| 305 | Up | *Apobec1* | NM_012907 | 181.39 | 55.90 | 3.2 | 2.24E-09 | 1.45E-07 |
| 306 | Down | *Arap3* | XM_003753045 | 105.26 | 294.14 | -2.8 | 2.25E-09 | 1.46E-07 |
| 307 | Up | *Nkain1* | XM_008764183 | 71.70 | 29.68 | 2.4 | 2.30E-09 | 1.48E-07 |
| 308 | Up | *Rhoq* | NM_053522 | 179.63 | 87.29 | 2.1 | 2.32E-09 | 1.48E-07 |
| 309 | Up | *Pla2g7* | NM_001009353 | 164.84 | 40.73 | 4.0 | 2.36E-09 | 1.50E-07 |
| 310 | Down | *Plscr2* | NM_001014094 | 119.91 | 253.24 | -2.1 | 2.37E-09 | 1.51E-07 |
| 311 | Down | *Efnb2* | NM_001107328 | 370.62 | 1080.95 | -2.9 | 2.59E-09 | 1.62E-07 |
| 312 | Up | *Itgb4* | NM_013180 | 261.65 | 63.05 | 4.2 | 2.61E-09 | 1.63E-07 |
| 313 | Up | *Slamf9* | NM_001105971 | 123.25 | 27.36 | 4.5 | 2.63E-09 | 1.64E-07 |
| 314 | Up | *Lum* | NM_031050 | 118.56 | 32.66 | 3.6 | 2.69E-09 | 1.67E-07 |
| 315 | Up | *Mpzl3* | NM_001108760 | 76.64 | 29.24 | 2.6 | 2.70E-09 | 1.67E-07 |
| 316 | Down | *Syne1* | XM_006227851 | 113.15 | 230.40 | -2.0 | 2.72E-09 | 1.68E-07 |
| 317 | Up | *Mt1m* | XM_002728917 | 1384.87 | 175.59 | 7.9 | 2.77E-09 | 1.70E-07 |
| 318 | Down | *Cnr1* | NM_012784 | 25.78 | 86.08 | -3.3 | 2.79E-09 | 1.70E-07 |
| 319 | Down | *Tmem255a* | NM_182822 | 22.28 | 80.12 | -3.6 | 2.82E-09 | 1.71E-07 |
| 320 | Up | *Tlr1* | NM_001172120 | 38.70 | 17.18 | 2.3 | 2.83E-09 | 1.72E-07 |
| 321 | Up | *LOC290595* | NM_001106063 | 607.25 | 20.12 | 30.2 | 2.97E-09 | 1.79E-07 |
| 322 | Up | *Pdlim7* | NM_173125 | 230.60 | 97.37 | 2.4 | 3.00E-09 | 1.80E-07 |
| 323 | Down | *Ciart* | XM_003753652 | 80.55 | 173.69 | -2.2 | 3.01E-09 | 1.80E-07 |
| 324 | Up | *Tagln* | NM_031549 | 1027.35 | 390.97 | 2.6 | 3.07E-09 | 1.83E-07 |
| 325 | Down | *Foxp2* | NM_001271104 | 37.95 | 78.68 | -2.1 | 3.08E-09 | 1.83E-07 |
| 326 | Down | *Calcrl* | NM_012717 | 857.30 | 1915.27 | -2.2 | 3.12E-09 | 1.85E-07 |
| 327 | Up | *Klra5* | XM_008763341 | 10.29 | 3.62 | 2.8 | 3.12E-09 | 1.85E-07 |
| 328 | Down | *Col6a5* | XM_008757899 | 81.90 | 245.25 | -3.0 | 3.19E-09 | 1.88E-07 |
| 329 | Down | *Fgfr2* | NM_001109892 | 276.11 | 664.01 | -2.4 | 3.20E-09 | 1.88E-07 |
| 330 | Up | *Adam12* | XM_008759968 | 218.66 | 53.85 | 4.1 | 3.20E-09 | 1.88E-07 |
| 331 | Down | *Hspb11* | NM_001131002 | 45.41 | 93.14 | -2.1 | 3.30E-09 | 1.93E-07 |
| 332 | Down | *Dnah9* | XM_002724507 | 27.17 | 102.76 | -3.8 | 3.31E-09 | 1.93E-07 |
| 333 | Down | *RGD1561147* | XM_008773023 | 20.57 | 63.22 | -3.1 | 3.36E-09 | 1.96E-07 |
| 334 | Down | *Sh2d6* | XM_008763005 | 39.78 | 167.60 | -4.2 | 3.44E-09 | 1.99E-07 |
| 335 | Down | *Nostrin* | NM_001024260 | 95.45 | 235.80 | -2.5 | 3.47E-09 | 2.01E-07 |
| 336 | Down | *Grem2* | NM_001105974 | 65.59 | 136.99 | -2.1 | 3.59E-09 | 2.06E-07 |
| 337 | Up | *Usp18* | NM_001014058 | 192.83 | 49.09 | 3.9 | 3.66E-09 | 2.10E-07 |
| 338 | Down | *Grap* | NM_001025749 | 39.61 | 95.10 | -2.4 | 3.79E-09 | 2.16E-07 |
| 339 | Up | *Iffo2* | NM_001134703 | 222.64 | 108.96 | 2.0 | 3.85E-09 | 2.18E-07 |
| 340 | Up | *Cd80* | NM_012926 | 128.01 | 35.04 | 3.7 | 3.89E-09 | 2.19E-07 |
| 341 | Down | *Ccdc187* | NM_001134617 | 32.22 | 80.87 | -2.5 | 3.96E-09 | 2.23E-07 |
| 342 | Down | *Enkur* | NM_001106126 | 49.84 | 111.14 | -2.2 | 4.14E-09 | 2.31E-07 |
| 343 | Down | *Fzd4* | NM_022623 | 131.45 | 317.05 | -2.4 | 4.18E-09 | 2.33E-07 |
| 344 | Up | *Arg1* | NM_017134 | 170.35 | 57.50 | 3.0 | 4.19E-09 | 2.33E-07 |
| 345 | Up | *Pkhd1* | XM_001070557 | 38.22 | 13.81 | 2.8 | 4.22E-09 | 2.33E-07 |
| 346 | Down | *Zbtb20* | NM_001105880 | 183.40 | 449.27 | -2.4 | 4.23E-09 | 2.33E-07 |
| 347 | Down | *Stxbp6* | NM_001191872 | 126.87 | 344.84 | -2.7 | 4.27E-09 | 2.34E-07 |
| 348 | Down | *Akr1c14* | NM_138547 | 89.92 | 230.88 | -2.6 | 4.29E-09 | 2.35E-07 |
| 349 | Up | *Basp1* | NM_022300 | 286.95 | 135.40 | 2.1 | 4.35E-09 | 2.37E-07 |
| 350 | Down | *Npr1* | NM_012613 | 102.64 | 227.50 | -2.2 | 4.58E-09 | 2.47E-07 |
| 351 | Up | *Pdzk1ip1* | NM_130401 | 68.07 | 31.96 | 2.1 | 4.63E-09 | 2.49E-07 |
| 352 | Up | *Gem* | NM_001106637 | 148.98 | 72.31 | 2.1 | 4.78E-09 | 2.56E-07 |
| 353 | Down | *Rap1gap* | NM_001100713 | 35.77 | 75.24 | -2.1 | 4.94E-09 | 2.62E-07 |
| 354 | Down | *Olfml2a* | NM_001106572 | 179.88 | 454.22 | -2.5 | 4.98E-09 | 2.64E-07 |
| 355 | Up | *B3gnt5* | NM_053932 | 67.95 | 29.13 | 2.3 | 5.03E-09 | 2.66E-07 |
| 356 | Up | *Cdh2* | NM_031333 | 63.74 | 26.70 | 2.4 | 5.27E-09 | 2.75E-07 |
| 357 | Up | *Dsc2* | NM_001033688 | 275.30 | 34.62 | 8.0 | 5.31E-09 | 2.77E-07 |
| 358 | Up | *Ifitm1* | NM_001106314 | 631.11 | 249.09 | 2.5 | 5.49E-09 | 2.85E-07 |
| 359 | Down | *Slc6a4* | NM_013034 | 81.75 | 193.51 | -2.4 | 5.63E-09 | 2.91E-07 |
| 360 | Up | *Actg2* | NM_012893 | 236.22 | 92.98 | 2.5 | 5.88E-09 | 3.04E-07 |
| 361 | Up | *Tuba8* | NM_001024339 | 67.00 | 19.80 | 3.4 | 6.10E-09 | 3.10E-07 |
| 362 | Up | *Cdh13* | NM_138889 | 72.31 | 29.86 | 2.4 | 6.55E-09 | 3.29E-07 |
| 363 | Up | *Pkib* | NM_001076553 | 206.68 | 83.61 | 2.5 | 6.70E-09 | 3.34E-07 |
| 364 | Down | *Exph5* | XM_001072028 | 103.28 | 212.23 | -2.1 | 6.75E-09 | 3.36E-07 |
| 365 | Up | *C1s* | NM_138900 | 530.40 | 176.07 | 3.0 | 6.80E-09 | 3.37E-07 |
| 366 | Down | *Lrrc34* | NM_001044696 | 23.48 | 77.46 | -3.3 | 7.01E-09 | 3.45E-07 |
| 367 | Up | *Clec4a2* | NM_001005880 | 47.10 | 19.63 | 2.4 | 7.08E-09 | 3.47E-07 |
| 368 | Down | *Ank2* | XM_008761478 | 62.85 | 132.38 | -2.1 | 7.08E-09 | 3.47E-07 |
| 369 | Down | *Gpt* | NM_031039 | 55.82 | 123.83 | -2.2 | 7.40E-09 | 3.58E-07 |
| 370 | Up | *Rhbdl2* | NM_001106684 | 44.89 | 14.63 | 3.1 | 7.52E-09 | 3.62E-07 |
| 371 | Up | *Rps27l* | NM_001276477 | 88.13 | 38.98 | 2.3 | 7.53E-09 | 3.62E-07 |
| 372 | Down | *Fgfr4* | NM_001109904 | 68.99 | 217.50 | -3.2 | 7.54E-09 | 3.62E-07 |
| 373 | Up | *Pmaip1* | NM_001008385 | 86.29 | 29.89 | 2.9 | 7.55E-09 | 3.62E-07 |
| 374 | Down | *Cacnb2* | NM_053851 | 69.39 | 157.96 | -2.3 | 7.56E-09 | 3.62E-07 |
| 375 | Up | *Mreg* | NM_001192002 | 50.69 | 20.01 | 2.5 | 7.70E-09 | 3.67E-07 |
| 376 | Down | *Spag6* | NM_001034960 | 43.23 | 179.44 | -4.2 | 7.76E-09 | 3.69E-07 |
| 377 | Down | *RGD1564149* | NM_001079708 | 57.04 | 154.73 | -2.7 | 8.00E-09 | 3.79E-07 |
| 378 | Up | *Serpinb5* | NM_057108 | 175.33 | 5.37 | 32.6 | 8.02E-09 | 3.79E-07 |
| 379 | Up | *Hist1h1b* | NM_001109417 | 157.75 | 64.95 | 2.4 | 8.10E-09 | 3.82E-07 |
| 380 | Up | *Cd68* | NM_001031638 | 706.51 | 253.48 | 2.8 | 8.10E-09 | 3.82E-07 |
| 381 | Down | *Wdr63* | NM_001134736 | 27.48 | 107.72 | -3.9 | 8.15E-09 | 3.83E-07 |
| 382 | Down | *Tppp* | NM_001108461 | 84.70 | 301.67 | -3.6 | 8.19E-09 | 3.84E-07 |
| 383 | Up | *Fcrl2* | NM_001107702 | 62.66 | 22.20 | 2.8 | 8.20E-09 | 3.84E-07 |
| 384 | Down | *Lrrn3* | NM_030856 | 29.65 | 108.46 | -3.7 | 8.54E-09 | 3.98E-07 |
| 385 | Down | *Gimap8* | NM_001033923 | 90.67 | 234.63 | -2.6 | 8.64E-09 | 4.02E-07 |
| 386 | Up | *Atp8b1* | NM_001106140 | 125.62 | 55.07 | 2.3 | 9.38E-09 | 4.31E-07 |
| 387 | Down | *Sec14l3* | NM_022608 | 338.30 | 2212.92 | -6.5 | 9.41E-09 | 4.32E-07 |
| 388 | Down | *Adrb2* | NM_012492 | 152.87 | 309.91 | -2.0 | 9.51E-09 | 4.36E-07 |
| 389 | Down | *Apln* | NM_031612 | 119.47 | 295.67 | -2.5 | 9.60E-09 | 4.38E-07 |
| 390 | Down | *Cyp2e1* | NM_031543 | 12.56 | 58.28 | -4.6 | 9.66E-09 | 4.40E-07 |
| 391 | Up | *Shmt2* | NM_001008322 | 132.30 | 65.77 | 2.0 | 1.03E-08 | 4.62E-07 |
| 392 | Down | *F11* | NM_001047848 | 16.76 | 39.36 | -2.3 | 1.05E-08 | 4.71E-07 |
| 393 | Up | *Col1a2* | NM_053356 | 1208.74 | 495.22 | 2.4 | 1.06E-08 | 4.71E-07 |
| 394 | Down | *Phex* | NM_013004 | 32.73 | 92.13 | -2.8 | 1.07E-08 | 4.77E-07 |
| 395 | Up | *Ereg* | NM_021689 | 128.95 | 29.50 | 4.4 | 1.08E-08 | 4.77E-07 |
| 396 | Down | *Ccdc108* | XM_008758069 | 31.61 | 108.95 | -3.4 | 1.11E-08 | 4.86E-07 |
| 397 | Down | *Epas1* | NM_023090 | 1907.96 | 3907.07 | -2.0 | 1.13E-08 | 4.94E-07 |
| 398 | Down | *Tnfsf10* | NM_145681 | 107.81 | 649.58 | -6.0 | 1.15E-08 | 5.02E-07 |
| 399 | Down | *Tfdp2* | NM_001106847 | 63.82 | 134.23 | -2.1 | 1.17E-08 | 5.08E-07 |
| 400 | Up | *Gas7* | NM_053484 | 188.11 | 91.67 | 2.1 | 1.20E-08 | 5.21E-07 |
| 401 | Down | *LOC102553195* | XM_006225183 | 25.95 | 113.37 | -4.4 | 1.24E-08 | 5.38E-07 |
| 402 | Up | *Tmprss11g* | NM_001008554 | 160.46 | 9.68 | 16.6 | 1.25E-08 | 5.38E-07 |
| 403 | Up | *Ccnb2* | NM_001009470 | 66.52 | 29.71 | 2.2 | 1.27E-08 | 5.46E-07 |
| 404 | Up | *Bcl3* | NM_001109422 | 116.82 | 49.89 | 2.3 | 1.29E-08 | 5.52E-07 |
| 405 | Down | *Cfap44* | XM_008768785 | 54.56 | 232.35 | -4.3 | 1.31E-08 | 5.59E-07 |
| 406 | Down | *Gpr155* | NM_001107811 | 185.49 | 462.71 | -2.5 | 1.37E-08 | 5.84E-07 |
| 407 | Up | *Tacr1* | NM_012667 | 45.71 | 16.92 | 2.7 | 1.45E-08 | 6.12E-07 |
| 408 | Up | *C6* | NM_176074 | 307.32 | 38.30 | 8.0 | 1.48E-08 | 6.18E-07 |
| 409 | Down | *Fmo1* | NM_012792 | 203.13 | 999.42 | -4.9 | 1.50E-08 | 6.23E-07 |
| 410 | Down | *Cmklr1* | NM_022218 | 170.32 | 422.76 | -2.5 | 1.53E-08 | 6.36E-07 |
| 411 | Up | *Dpp7* | NM_031973 | 352.07 | 142.51 | 2.5 | 1.57E-08 | 6.51E-07 |
| 412 | Down | *Fmo2* | NM_144737 | 409.31 | 1982.70 | -4.8 | 1.68E-08 | 6.78E-07 |
| 413 | Up | *Tpi1* | NM_022922 | 768.17 | 359.13 | 2.1 | 1.68E-08 | 6.79E-07 |
| 414 | Down | *Ackr4* | XM_006226547 | 78.31 | 477.06 | -6.1 | 1.72E-08 | 6.92E-07 |
| 415 | Down | *Gstm7* | NM_031154 | 34.84 | 88.70 | -2.5 | 1.73E-08 | 6.97E-07 |
| 416 | Up | *Uchl1* | NM_017237 | 47.33 | 23.21 | 2.0 | 1.74E-08 | 6.98E-07 |
| 417 | Down | *Hydin* | XM_008772592 | 33.95 | 117.70 | -3.5 | 1.79E-08 | 7.12E-07 |
| 418 | Down | *Npas2* | NM_001108214 | 157.48 | 321.89 | -2.0 | 1.82E-08 | 7.21E-07 |
| 419 | Up | *Plau* | ENSRNOT00000014273 | 318.93 | 148.20 | 2.2 | 1.82E-08 | 7.22E-07 |
| 420 | Up | *RGD1564688* | ENSRNOT00000058917 | 86.39 | 39.52 | 2.2 | 1.90E-08 | 7.44E-07 |
| 421 | Down | *Arhgap29* | NM_001009405 | 390.33 | 870.56 | -2.2 | 1.92E-08 | 7.50E-07 |
| 422 | Down | *Gpr158* | NM_001170326 | 32.34 | 89.27 | -2.8 | 1.94E-08 | 7.56E-07 |
| 423 | Up | *Olfml2b* | NM_001107195 | 104.19 | 44.24 | 2.4 | 1.94E-08 | 7.57E-07 |
| 424 | Up | *Rcc2* | XM_008764324 | 265.59 | 128.27 | 2.1 | 1.96E-08 | 7.62E-07 |
| 425 | Up | *LOC100359563* | ENSRNOT00000043008 | 651.33 | 306.68 | 2.1 | 1.97E-08 | 7.64E-07 |
| 426 | Up | *Fhl2* | NM_031677 | 65.50 | 28.30 | 2.3 | 1.99E-08 | 7.71E-07 |
| 427 | Up | *Plod2* | NM_001142915 | 435.26 | 111.13 | 3.9 | 2.00E-08 | 7.76E-07 |
| 428 | Up | *Cd80* | NM_012926 | 79.33 | 27.19 | 2.9 | 2.05E-08 | 7.92E-07 |
| 429 | Up | *Gpr176* | NM_001270986 | 71.40 | 34.49 | 2.1 | 2.06E-08 | 7.92E-07 |
| 430 | Up | *Rab7b* | NM_001109328 | 75.13 | 32.44 | 2.3 | 2.06E-08 | 7.92E-07 |
| 431 | Down | *Slc9a3r2* | NM_053811 | 197.52 | 425.21 | -2.2 | 2.06E-08 | 7.92E-07 |
| 432 | Down | *Spag16* | NM_001134728 | 17.28 | 47.55 | -2.8 | 2.06E-08 | 7.92E-07 |
| 433 | Up | *Gabrp* | NM_031029 | 345.03 | 33.43 | 10.3 | 2.10E-08 | 8.04E-07 |
| 434 | Down | *Rnf24* | NM_001108591 | 36.24 | 75.68 | -2.1 | 2.19E-08 | 8.34E-07 |
| 435 | Up | *Col12a1* | XM_001060689 | 1079.21 | 531.71 | 2.0 | 2.22E-08 | 8.44E-07 |
| 436 | Up | *Diaph3* | NM_001305172 | 115.17 | 37.46 | 3.1 | 2.24E-08 | 8.52E-07 |
| 437 | Down | *Rsb66* | NM_181694 | 46.89 | 95.22 | -2.0 | 2.26E-08 | 8.58E-07 |
| 438 | Down | *Hecw2* | NM_001108218 | 93.18 | 187.34 | -2.0 | 2.28E-08 | 8.58E-07 |
| 439 | Up | *Eln* | NM_012722 | 1223.36 | 511.28 | 2.4 | 2.29E-08 | 8.58E-07 |
| 440 | Down | *LOC102546798* | XR_590458 | 148.34 | 405.67 | -2.7 | 2.30E-08 | 8.58E-07 |
| 441 | Down | *LOC102546798* | XR_590458 | 148.34 | 405.67 | -2.7 | 2.30E-08 | 8.58E-07 |
| 442 | Down | *LOC103690686* | XR_589530 | 148.34 | 405.67 | -2.7 | 2.30E-08 | 8.58E-07 |
| 443 | Down | *LOC103690686* | XR_589530 | 148.34 | 405.67 | -2.7 | 2.30E-08 | 8.58E-07 |
| 444 | Down | *LOC103690686* | XR_589530 | 148.34 | 405.67 | -2.7 | 2.30E-08 | 8.58E-07 |
| 445 | Down | *LOC103690686* | XR_589530 | 148.34 | 405.67 | -2.7 | 2.30E-08 | 8.58E-07 |
| 446 | Down | *LOC103690686* | XR_589530 | 148.34 | 405.67 | -2.7 | 2.30E-08 | 8.58E-07 |
| 447 | Down | *LOC103690686* | XR_589530 | 148.34 | 405.67 | -2.7 | 2.30E-08 | 8.58E-07 |
| 448 | Down | *Akr1c19* | NM_001100576 | 39.78 | 92.85 | -2.3 | 2.30E-08 | 8.58E-07 |
| 449 | Up | *Glis3* | XM_008760357 | 91.40 | 43.28 | 2.1 | 2.39E-08 | 8.80E-07 |
| 450 | Down | *Sema6d* | NM_001107768 | 115.79 | 255.84 | -2.2 | 2.39E-08 | 8.82E-07 |
| 451 | Up | *Ccdc80* | NM_022543 | 497.06 | 192.11 | 2.6 | 2.42E-08 | 8.90E-07 |
| 452 | Down | *Dnah9* | XM_002724507 | 25.71 | 81.27 | -3.2 | 2.55E-08 | 9.27E-07 |
| 453 | Up | *Gpr88* | NM_031696 | 93.06 | 43.94 | 2.1 | 2.56E-08 | 9.27E-07 |
| 454 | Up | *C1r* | NM_001134555 | 750.71 | 338.98 | 2.2 | 2.60E-08 | 9.39E-07 |
| 455 | Down | *Kcnj15* | NM_133321 | 145.23 | 302.55 | -2.1 | 2.62E-08 | 9.43E-07 |
| 456 | Up | *Cxcl13* | NM_001017496 | 79.81 | 13.95 | 5.7 | 2.63E-08 | 9.45E-07 |
| 457 | Up | *Serpinb2* | NM_021696 | 329.14 | 10.18 | 32.3 | 2.63E-08 | 9.45E-07 |
| 458 | Up | *Aspm* | NM_001105955 | 49.07 | 22.41 | 2.2 | 2.67E-08 | 9.55E-07 |
| 459 | Down | *Slc7a10* | NM_053726 | 29.84 | 101.10 | -3.4 | 2.69E-08 | 9.61E-07 |
| 460 | Down | *Shank3* | NM_021676 | 58.96 | 148.50 | -2.5 | 2.69E-08 | 9.61E-07 |
| 461 | Up | *Flrt2* | NM_001106750 | 296.21 | 104.51 | 2.8 | 2.71E-08 | 9.65E-07 |
| 462 | Down | *Ak9* | XM_003753496 | 21.33 | 74.87 | -3.5 | 2.74E-08 | 9.75E-07 |
| 463 | Down | *Dcdc5* | NM_001114621 | 15.52 | 35.62 | -2.3 | 2.80E-08 | 9.93E-07 |
| 464 | Down | *Iqca1* | XM_001066515 | 18.59 | 44.15 | -2.4 | 2.81E-08 | 9.94E-07 |
| 465 | Down | *Prex2* | NM_001107899 | 229.28 | 684.12 | -3.0 | 2.81E-08 | 9.94E-07 |
| 466 | Down | *LOC100910790* | XM_008770410 | 104.02 | 403.17 | -3.9 | 2.83E-08 | 9.99E-07 |
| 467 | Down | *Dnah11* | XM_001061747 | 26.03 | 85.40 | -3.3 | 2.86E-08 | 1.01E-06 |
| 468 | Up | *Htr2b* | NM_017250 | 69.14 | 25.92 | 2.7 | 3.01E-08 | 1.05E-06 |
| 469 | Down | *Cfap54* | XM_006241270 | 32.09 | 98.13 | -3.1 | 3.10E-08 | 1.08E-06 |
| 470 | Up | *Serpina3n* | ENSRNOT00000014073 | 923.34 | 82.94 | 11.1 | 3.11E-08 | 1.08E-06 |
| 471 | Down | *Atp1a2* | NM_012505 | 38.96 | 114.12 | -2.9 | 3.11E-08 | 1.08E-06 |
| 472 | Up | *Loxl2* | ENSRNOT00000058663 | 137.87 | 57.51 | 2.4 | 3.13E-08 | 1.08E-06 |
| 473 | Down | *Hepacam2* | NM_001106580 | 17.49 | 63.52 | -3.6 | 3.15E-08 | 1.09E-06 |
| 474 | Down | *Asb14* | NM_001170749 | 20.17 | 102.69 | -5.1 | 3.20E-08 | 1.10E-06 |
| 475 | Up | *Sertad4* | NM_001108351 | 145.93 | 67.67 | 2.2 | 3.21E-08 | 1.10E-06 |
| 476 | Down | *Ager* | NM_053336 | 1655.83 | 3774.34 | -2.3 | 3.21E-08 | 1.10E-06 |
| 477 | Down | *Fam47e* | XM_006221696 | 28.11 | 61.50 | -2.2 | 3.22E-08 | 1.10E-06 |
| 478 | Down | *Ppp1r32* | NM_145786 | 13.25 | 29.06 | -2.2 | 3.28E-08 | 1.12E-06 |
| 479 | Up | *Ankrd1* | NM_013220 | 518.06 | 117.04 | 4.4 | 3.30E-08 | 1.12E-06 |
| 480 | Up | *Hist1h2ac* | XM_001062714 | 18.53 | 7.82 | 2.4 | 3.46E-08 | 1.17E-06 |
| 481 | Down | *Neb* | XM_006224416 | 44.66 | 124.35 | -2.8 | 3.52E-08 | 1.18E-06 |
| 482 | Down | *Akap12* | NM_001033653 | 87.28 | 210.05 | -2.4 | 3.52E-08 | 1.18E-06 |
| 483 | Up | *Lox* | NM_017061 | 741.26 | 345.23 | 2.1 | 3.52E-08 | 1.18E-06 |
| 484 | Up | *Fam20c* | NM_001012238 | 201.31 | 90.02 | 2.2 | 3.59E-08 | 1.20E-06 |
| 485 | Down | *Amigo2* | NM_182816 | 290.02 | 862.48 | -3.0 | 3.62E-08 | 1.21E-06 |
| 486 | Down | *RGD1565611* | NM_001110155 | 53.84 | 117.26 | -2.2 | 3.62E-08 | 1.21E-06 |
| 487 | Up | *Nuf2* | NM_001012028 | 49.49 | 19.56 | 2.5 | 3.72E-08 | 1.23E-06 |
| 488 | Up | *Retnlg* | XM_006248282 | 63.62 | 14.16 | 4.5 | 3.78E-08 | 1.25E-06 |
| 489 | Up | *Eif4ebp1* | NM_053857 | 225.61 | 109.08 | 2.1 | 3.91E-08 | 1.28E-06 |
| 490 | Down | *Maats1* | XM_008768768 | 32.74 | 84.00 | -2.6 | 4.00E-08 | 1.30E-06 |
| 491 | Up | *Asgr2* | NM_017189 | 57.86 | 26.41 | 2.2 | 4.09E-08 | 1.33E-06 |
| 492 | Up | *Pappa* | NM_001107939 | 98.06 | 40.92 | 2.4 | 4.10E-08 | 1.33E-06 |
| 493 | Up | *Ttk* | NM_001108172 | 52.45 | 18.07 | 2.9 | 4.12E-08 | 1.33E-06 |
| 494 | Down | *LOC102548248* | XM_008764471 | 217.86 | 571.36 | -2.6 | 4.23E-08 | 1.36E-06 |
| 495 | Down | *Ednrb* | NM_017333 | 804.22 | 1822.69 | -2.3 | 4.23E-08 | 1.36E-06 |
| 496 | Up | *Ccl7* | NM_001007612 | 139.26 | 19.88 | 7.0 | 4.25E-08 | 1.36E-06 |
| 497 | Down | *Pdzd2* | NM_022940 | 205.12 | 556.33 | -2.7 | 4.29E-08 | 1.37E-06 |
| 498 | Down | *Kctd8* | NM_001100172 | 14.65 | 30.38 | -2.1 | 4.31E-08 | 1.37E-06 |
| 499 | Down | *Rgs6* | NM_019342 | 49.37 | 128.31 | -2.6 | 4.32E-08 | 1.37E-06 |
| 500 | Down | *Lrrc9* | NM_001191613 | 9.78 | 25.00 | -2.6 | 4.33E-08 | 1.38E-06 |
| 501 | Down | *Bmp6* | NM_013107 | 351.62 | 873.86 | -2.5 | 4.41E-08 | 1.40E-06 |
| 502 | Down | *RGD1310587* | NM_001100857 | 87.86 | 275.42 | -3.1 | 4.50E-08 | 1.42E-06 |
| 503 | Up | *Mall* | NM_001014182 | 155.66 | 17.24 | 9.0 | 4.50E-08 | 1.42E-06 |
| 504 | Down | *Nsun7* | NM_001017452 | 48.41 | 153.69 | -3.2 | 4.65E-08 | 1.45E-06 |
| 505 | Down | *Lrrc6* | NM_001025659 | 27.44 | 67.50 | -2.5 | 4.71E-08 | 1.47E-06 |
| 506 | Up | *Gpr39* | NM_001100943 | 61.07 | 28.78 | 2.1 | 4.72E-08 | 1.47E-06 |
| 507 | Down | *Dnah9* | XM_002724507 | 40.51 | 113.22 | -2.8 | 4.72E-08 | 1.47E-06 |
| 508 | Up | *Hk3* | NM_022179 | 76.66 | 35.46 | 2.2 | 4.72E-08 | 1.47E-06 |
| 509 | Down | *RGD1561147* | OTTRNOT00000001670 | 21.23 | 51.25 | -2.4 | 4.76E-08 | 1.48E-06 |
| 510 | Up | *RGD1562618* | NM_001113791 | 152.82 | 64.99 | 2.4 | 4.81E-08 | 1.49E-06 |
| 511 | Down | *Kcnrg* | NM_001191687 | 27.71 | 77.26 | -2.8 | 4.85E-08 | 1.50E-06 |
| 512 | Up | *Rgs10* | NM_019337 | 113.41 | 56.01 | 2.0 | 4.89E-08 | 1.51E-06 |
| 513 | Down | *Gucy1a2* | NM_023956 | 50.77 | 102.97 | -2.0 | 4.95E-08 | 1.52E-06 |
| 514 | Down | *Smad9* | NM_138872 | 56.66 | 142.78 | -2.5 | 5.13E-08 | 1.57E-06 |
| 515 | Down | *Mamdc2* | XM_006223679 | 83.26 | 250.11 | -3.0 | 5.16E-08 | 1.57E-06 |
| 516 | Down | *Phactr1* | NM_214457 | 113.88 | 235.46 | -2.1 | 5.23E-08 | 1.59E-06 |
| 517 | Up | *Ms4a12* | XM_006223643 | 56.35 | 10.29 | 5.5 | 5.26E-08 | 1.60E-06 |
| 518 | Down | *Efhc2* | NM_001106952 | 16.42 | 37.52 | -2.3 | 5.28E-08 | 1.60E-06 |
| 519 | Down | *Tox3* | NM_001106171 | 47.66 | 148.53 | -3.1 | 5.31E-08 | 1.61E-06 |
| 520 | Down | *Adgre5* | NM_001012164 | 420.22 | 1243.47 | -3.0 | 5.43E-08 | 1.63E-06 |
| 521 | Up | *Slc4a7* | NM_001270860 | 193.71 | 92.57 | 2.1 | 5.51E-08 | 1.65E-06 |
| 522 | Down | *LOC100361866* | XM_002728192 | 101.81 | 215.84 | -2.1 | 5.55E-08 | 1.66E-06 |
| 523 | Down | *Dnah9* | XM_002724507 | 19.67 | 71.39 | -3.6 | 5.57E-08 | 1.66E-06 |
| 524 | Down | *Ak7* | NM_001108055 | 31.28 | 86.99 | -2.8 | 5.57E-08 | 1.66E-06 |
| 525 | Down | *Vwa3a* | NM_001079885 | 23.36 | 78.72 | -3.4 | 5.95E-08 | 1.75E-06 |
| 526 | Up | *Ptprn* | NM_053881 | 64.52 | 27.20 | 2.4 | 6.17E-08 | 1.80E-06 |
| 527 | Up | *Kif2c* | NM_001085369 | 35.73 | 17.83 | 2.0 | 6.24E-08 | 1.82E-06 |
| 528 | Up | *Steap3* | NM_133314 | 80.92 | 38.40 | 2.1 | 6.32E-08 | 1.83E-06 |
| 529 | Up | *LOC100364062* | XM_002727444 | 499.15 | 211.84 | 2.4 | 6.50E-08 | 1.87E-06 |
| 530 | Up | *Uhrf1* | NM_001008882 | 78.11 | 35.83 | 2.2 | 6.79E-08 | 1.93E-06 |
| 531 | Up | *Vtcn1* | NM_001024244 | 96.46 | 28.05 | 3.4 | 6.94E-08 | 1.96E-06 |
| 532 | Up | *Psca* | NM_001172106 | 35.01 | 16.21 | 2.2 | 6.95E-08 | 1.97E-06 |
| 533 | Down | *Cacna1d* | NM_017298 | 26.32 | 62.76 | -2.4 | 6.96E-08 | 1.97E-06 |
| 534 | Down | *Ttll9* | NM_001014051 | 32.10 | 68.66 | -2.1 | 6.98E-08 | 1.97E-06 |
| 535 | Up | *Gas2l3* | XM_001056437 | 80.38 | 33.79 | 2.4 | 7.11E-08 | 2.00E-06 |
| 536 | Down | *LOC685158* | XM_008764589 | 25.20 | 76.62 | -3.0 | 7.21E-08 | 2.02E-06 |
| 537 | Up | *Grem1* | NM_019282 | 129.77 | 18.23 | 7.1 | 7.50E-08 | 2.09E-06 |
| 538 | Down | *Gal3st3* | NM_001024290 | 38.96 | 80.04 | -2.1 | 7.53E-08 | 2.09E-06 |
| 539 | Down | *LOC102548248* | XM_008764471 | 71.45 | 189.91 | -2.7 | 7.61E-08 | 2.11E-06 |
| 540 | Up | *Ccl2* | NM_031530 | 406.73 | 51.85 | 7.8 | 7.77E-08 | 2.15E-06 |
| 541 | Up | *Dsg3* | NM_001191079 | 184.38 | 17.58 | 10.5 | 7.81E-08 | 2.16E-06 |
| 542 | Down | *Acvrl1* | NM_022441 | 400.03 | 877.91 | -2.2 | 7.84E-08 | 2.16E-06 |
| 543 | Down | *Trpm5* | NM_001191896 | 25.40 | 52.76 | -2.1 | 7.85E-08 | 2.17E-06 |
| 544 | Down | *LOC686753* | XM_001075423 | 348.48 | 738.28 | -2.1 | 7.87E-08 | 2.17E-06 |
| 545 | Up | *Rgs16* | NM_001077589 | 63.48 | 25.79 | 2.5 | 7.89E-08 | 2.17E-06 |
| 546 | Up | *Ubd* | NM_053299 | 13.23 | 4.94 | 2.7 | 8.14E-08 | 2.22E-06 |
| 547 | Down | *Nr3c2* | NM_013131 | 45.09 | 92.42 | -2.0 | 8.44E-08 | 2.29E-06 |
| 548 | Up | *Hist1h3a* | XM_008771684 | 303.42 | 120.45 | 2.5 | 8.52E-08 | 2.31E-06 |
| 549 | Down | *Dnah7* | XM_006244944 | 16.02 | 45.97 | -2.9 | 8.95E-08 | 2.41E-06 |
| 550 | Up | *Aldh18a1* | NM_001108524 | 144.38 | 72.05 | 2.0 | 8.98E-08 | 2.42E-06 |
| 551 | Up | *Kif20a* | NM_001108426 | 72.16 | 35.10 | 2.1 | 9.13E-08 | 2.45E-06 |
| 552 | Down | *Ppp1r1b* | NM_138521 | 41.06 | 93.97 | -2.3 | 9.19E-08 | 2.46E-06 |
| 553 | Down | *Plvap* | NM_020086 | 383.20 | 2257.70 | -5.9 | 9.22E-08 | 2.46E-06 |
| 554 | Down | *Sh2d7* | XM_008766361 | 12.90 | 47.86 | -3.7 | 9.27E-08 | 2.47E-06 |
| 555 | Down | *Gimap6* | NM_001011968 | 76.49 | 173.65 | -2.3 | 9.28E-08 | 2.47E-06 |
| 556 | Down | *Ak9* | XM_008773020 | 26.07 | 75.27 | -2.9 | 9.39E-08 | 2.50E-06 |
| 557 | Up | *Lif* | NM_022196 | 79.38 | 31.65 | 2.5 | 9.57E-08 | 2.53E-06 |
| 558 | Up | *Cxcl10* | NM_139089 | 83.57 | 31.32 | 2.7 | 9.66E-08 | 2.55E-06 |
| 559 | Up | *MGC105649* | NM_001008518 | 359.81 | 124.33 | 2.9 | 9.92E-08 | 2.60E-06 |
| 560 | Down | *Tekt4* | NM_001013965 | 32.58 | 65.24 | -2.0 | 9.92E-08 | 2.60E-06 |
| 561 | Down | *Sdpr* | NM_001007712 | 302.71 | 805.72 | -2.7 | 9.93E-08 | 2.60E-06 |
| 562 | Up | *Emb* | NM_053719 | 286.77 | 118.10 | 2.4 | 9.96E-08 | 2.60E-06 |
| 563 | Down | *RGD1309139* | NM_001134578 | 13.72 | 29.45 | -2.1 | 9.97E-08 | 2.60E-06 |
| 564 | Up | *Dsg2* | XM_006222509 | 235.51 | 111.72 | 2.1 | 1.01E-07 | 2.62E-06 |
| 565 | Up | *Lad1* | NM_001107942 | 333.34 | 79.26 | 4.2 | 1.02E-07 | 2.64E-06 |
| 566 | Down | *Far2* | XM_001074438 | 44.10 | 96.10 | -2.2 | 1.02E-07 | 2.64E-06 |
| 567 | Down | *Gpr182* | NM_053302 | 200.89 | 475.39 | -2.4 | 1.05E-07 | 2.70E-06 |
| 568 | Down | *LOC100360330* | XM_006253210 | 46.38 | 96.30 | -2.1 | 1.05E-07 | 2.70E-06 |
| 569 | Down | *Ecm2* | XM_006222357 | 45.96 | 94.78 | -2.1 | 1.06E-07 | 2.72E-06 |
| 570 | Up | *Mt2A* | ENSRNOT00000067391 | 114.18 | 9.37 | 12.2 | 1.06E-07 | 2.72E-06 |
| 571 | Down | *Spef2* | NM_022620 | 12.58 | 36.21 | -2.9 | 1.06E-07 | 2.73E-06 |
| 572 | Up | *Gfpt2* | NM_001002819 | 65.55 | 30.73 | 2.1 | 1.07E-07 | 2.73E-06 |
| 573 | Down | *Klrb1a* | NM_001010964 | 22.30 | 46.12 | -2.1 | 1.07E-07 | 2.74E-06 |
| 574 | Up | *Pkp1* | NM_001107181 | 345.68 | 35.82 | 9.6 | 1.09E-07 | 2.78E-06 |
| 575 | Up | *Cotl1* | NM_001108452 | 149.52 | 72.52 | 2.1 | 1.09E-07 | 2.79E-06 |
| 576 | Down | *RGD1560020_predicted* | XM_003753133 | 37.26 | 90.68 | -2.4 | 1.13E-07 | 2.87E-06 |
| 577 | Up | *Tac4* | NM_172328 | 46.15 | 22.15 | 2.1 | 1.13E-07 | 2.88E-06 |
| 578 | Up | *Slc39a6* | NM_001024745 | 273.14 | 102.85 | 2.7 | 1.14E-07 | 2.88E-06 |
| 579 | Up | *Aldh1l2* | NM_001191778 | 34.67 | 13.64 | 2.5 | 1.14E-07 | 2.88E-06 |
| 580 | Up | *Ns5atp9* | NM_201418 | 112.29 | 50.50 | 2.2 | 1.18E-07 | 2.95E-06 |
| 581 | Down | *LOC103690686* | XR_589530 | 119.70 | 308.07 | -2.6 | 1.18E-07 | 2.96E-06 |
| 582 | Down | *Shh* | NM_017221 | 44.61 | 91.52 | -2.1 | 1.19E-07 | 2.97E-06 |
| 583 | Up | *Enpp3* | NM_019370 | 656.20 | 287.10 | 2.3 | 1.19E-07 | 2.98E-06 |
| 584 | Down | *RGD1560207* | XM_008758470 | 27.25 | 61.59 | -2.3 | 1.19E-07 | 2.98E-06 |
| 585 | Down | *Plxna2* | NM_001105988 | 261.17 | 556.28 | -2.1 | 1.23E-07 | 3.04E-06 |
| 586 | Up | *Ncapg* | XM_006221774 | 40.20 | 17.82 | 2.3 | 1.24E-07 | 3.07E-06 |
| 587 | Down | *Tmcc2* | NM_001305128 | 50.48 | 108.52 | -2.1 | 1.25E-07 | 3.08E-06 |
| 588 | Up | *Ddr1* | NM_001166022 | 134.41 | 66.45 | 2.0 | 1.26E-07 | 3.09E-06 |
| 589 | Up | *Slc39a14* | NM_001107275 | 104.43 | 42.18 | 2.5 | 1.27E-07 | 3.12E-06 |
| 590 | Up | *Aspn* | NM_001014008 | 64.27 | 15.79 | 4.1 | 1.29E-07 | 3.14E-06 |
| 591 | Up | *Mki67* | NM_001271366 | 335.80 | 125.85 | 2.7 | 1.30E-07 | 3.17E-06 |
| 592 | Down | *RGD1310209* | XR_085774 | 135.92 | 310.58 | -2.3 | 1.31E-07 | 3.19E-06 |
| 593 | Down | *LOC102549148* | XR_598844 | 58.02 | 135.48 | -2.3 | 1.35E-07 | 3.26E-06 |
| 594 | Down | *Cav1* | NM_031556 | 233.68 | 507.28 | -2.2 | 1.36E-07 | 3.29E-06 |
| 595 | Up | *Hmga1* | XM_006256156 | 416.01 | 193.27 | 2.2 | 1.36E-07 | 3.29E-06 |
| 596 | Up | *Ndc80* | NM_001126270 | 63.48 | 28.12 | 2.3 | 1.37E-07 | 3.30E-06 |
| 597 | Up | *Hist1h2ai* | XM_001061350 | 458.43 | 194.47 | 2.4 | 1.40E-07 | 3.35E-06 |
| 598 | Down | *Thsd1* | NM_001108878 | 59.53 | 120.73 | -2.0 | 1.43E-07 | 3.40E-06 |
| 599 | Down | *RGD1565862* | XM_006227295 | 13.11 | 33.84 | -2.6 | 1.44E-07 | 3.44E-06 |
| 600 | Down | *Dnah5* | XM_008760824 | 28.00 | 63.90 | -2.3 | 1.46E-07 | 3.47E-06 |
| 601 | Down | *Gucy1a3* | NM_017090 | 127.38 | 300.82 | -2.4 | 1.48E-07 | 3.50E-06 |
| 602 | Up | *Cthrc1* | NM_001271300 | 56.37 | 24.82 | 2.3 | 1.49E-07 | 3.52E-06 |
| 603 | Down | *Cfap206* | NM_001079701 | 25.90 | 73.03 | -2.8 | 1.51E-07 | 3.55E-06 |
| 604 | Up | *Depdc1* | NM_001305205 | 36.89 | 13.70 | 2.7 | 1.52E-07 | 3.57E-06 |
| 605 | Down | *Osbpl6* | XM_008761900 | 74.86 | 305.91 | -4.1 | 1.55E-07 | 3.63E-06 |
| 606 | Up | *S100a14* | XM_002725995 | 191.38 | 38.98 | 4.9 | 1.56E-07 | 3.64E-06 |
| 607 | Down | *Pon1* | NM_032077 | 17.69 | 67.69 | -3.8 | 1.56E-07 | 3.65E-06 |
| 608 | Down | *T2* | NM_001161835 | 16.38 | 55.16 | -3.4 | 1.56E-07 | 3.65E-06 |
| 609 | Up | *Clec2d2* | NM_001085402 | 70.54 | 19.33 | 3.6 | 1.57E-07 | 3.67E-06 |
| 610 | Down | *Prss23* | NM_001007691 | 459.34 | 922.24 | -2.0 | 1.58E-07 | 3.67E-06 |
| 611 | Down | *Dlc1* | NM_001127446 | 317.04 | 690.15 | -2.2 | 1.58E-07 | 3.68E-06 |
| 612 | Down | *Icam2* | NM_001007725 | 98.18 | 206.25 | -2.1 | 1.61E-07 | 3.74E-06 |
| 613 | Down | *Wdr49* | XM_008761118 | 15.67 | 33.56 | -2.1 | 1.62E-07 | 3.76E-06 |
| 614 | Up | *Ccnb1* | NM_171991 | 54.01 | 19.07 | 2.8 | 1.66E-07 | 3.83E-06 |
| 615 | Up | *Trib3* | NM_144755 | 69.85 | 28.11 | 2.5 | 1.67E-07 | 3.84E-06 |
| 616 | Up | *Rsad2* | NM_138881 | 119.27 | 53.15 | 2.2 | 1.75E-07 | 4.00E-06 |
| 617 | Down | *Cfap61* | XM_008762285 | 16.89 | 44.26 | -2.6 | 1.76E-07 | 4.01E-06 |
| 618 | Up | *Gpr34* | NM_001024925 | 74.22 | 21.97 | 3.4 | 1.77E-07 | 4.03E-06 |
| 619 | Down | *Dnah6* | XM_008763013 | 26.96 | 89.39 | -3.3 | 1.79E-07 | 4.06E-06 |
| 620 | Up | *Mnda* | NM_001012029 | 122.12 | 42.82 | 2.9 | 1.80E-07 | 4.08E-06 |
| 621 | Down | *Fras1* | NM_001191595 | 46.38 | 132.62 | -2.9 | 1.81E-07 | 4.10E-06 |
| 622 | Down | *Efcab1* | NM_001106930 | 22.79 | 66.01 | -2.9 | 1.82E-07 | 4.11E-06 |
| 623 | Down | *Tnxb* | BC168181 | 66.36 | 143.24 | -2.2 | 1.85E-07 | 4.17E-06 |
| 624 | Down | *Gnat3* | NM_173139 | 6.90 | 16.36 | -2.4 | 1.85E-07 | 4.18E-06 |
| 625 | Up | *Cdc20* | NM_171993 | 73.63 | 36.69 | 2.0 | 1.86E-07 | 4.18E-06 |
| 626 | Up | *Tnip3* | XM_006224951 | 54.54 | 19.16 | 2.8 | 1.89E-07 | 4.25E-06 |
| 627 | Up | *LOC100912024* | ENSRNOT00000046475 | 59.98 | 25.73 | 2.3 | 1.97E-07 | 4.38E-06 |
| 628 | Down | *Spag17* | XM_006224237 | 20.19 | 56.26 | -2.8 | 1.99E-07 | 4.42E-06 |
| 629 | Down | *Ppp1r14a* | NM_130403 | 93.17 | 197.40 | -2.1 | 2.08E-07 | 4.58E-06 |
| 630 | Up | *Glrx* | NM_022278 | 453.99 | 115.39 | 3.9 | 2.09E-07 | 4.60E-06 |
| 631 | Up | *Tpx2* | NM_001107790 | 66.55 | 26.82 | 2.5 | 2.12E-07 | 4.64E-06 |
| 632 | Up | *LOC500959* | NM_001033072 | 406.46 | 195.88 | 2.1 | 2.12E-07 | 4.64E-06 |
| 633 | Up | *Msln* | NM_031658 | 541.34 | 176.48 | 3.1 | 2.12E-07 | 4.64E-06 |
| 634 | Down | *Bmp3* | NM_017105 | 80.10 | 198.66 | -2.5 | 2.14E-07 | 4.66E-06 |
| 635 | Down | *Rsph4a* | XM_003751961 | 32.41 | 91.74 | -2.8 | 2.18E-07 | 4.74E-06 |
| 636 | Down | *LOC103689922* | XM_003749749 | 107.43 | 289.68 | -2.7 | 2.18E-07 | 4.74E-06 |
| 637 | Down | *Spata18* | NM_199374 | 52.28 | 158.01 | -3.0 | 2.20E-07 | 4.78E-06 |
| 638 | Down | *Rmdn2* | XM_006239633 | 53.94 | 168.92 | -3.1 | 2.22E-07 | 4.80E-06 |
| 639 | Down | *Gzmk* | NM_017119 | 42.66 | 110.53 | -2.6 | 2.22E-07 | 4.81E-06 |
| 640 | Up | *Pi15* | NM_001106917 | 103.46 | 29.81 | 3.5 | 2.28E-07 | 4.92E-06 |
| 641 | Up | *Ms4a7* | NM_001106338 | 31.60 | 15.11 | 2.1 | 2.30E-07 | 4.95E-06 |
| 642 | Down | *Spock2* | NM_001108533 | 129.23 | 494.16 | -3.8 | 2.31E-07 | 4.96E-06 |
| 643 | Down | *Faxdc2* | XM_001077260 | 181.13 | 378.57 | -2.1 | 2.31E-07 | 4.96E-06 |
| 644 | Up | *Hist1h2ak* | NM_001109423 | 134.78 | 58.34 | 2.3 | 2.32E-07 | 4.97E-06 |
| 645 | Up | *Fgf23* | NM_130754 | 57.21 | 11.85 | 4.8 | 2.33E-07 | 4.99E-06 |
| 646 | Down | *Phyhipl* | NM_001012076 | 55.06 | 247.52 | -4.5 | 2.35E-07 | 5.01E-06 |
| 647 | Up | *Cdca3* | NM_001007648 | 34.29 | 14.88 | 2.3 | 2.43E-07 | 5.15E-06 |
| 648 | Up | *Il6* | NM_012589 | 37.35 | 14.22 | 2.6 | 2.43E-07 | 5.15E-06 |
| 649 | Up | *Tmem176a* | NM_001039008 | 662.06 | 320.51 | 2.1 | 2.64E-07 | 5.52E-06 |
| 650 | Down | *Cysltr2* | NM_133413 | 15.06 | 31.20 | -2.1 | 2.66E-07 | 5.55E-06 |
| 651 | Up | *Cpxm1* | NM_001106511 | 104.45 | 39.85 | 2.6 | 2.67E-07 | 5.57E-06 |
| 652 | Up | *Hmox1* | NM_012580 | 399.43 | 83.15 | 4.8 | 2.75E-07 | 5.70E-06 |
| 653 | Down | *Fbxl13* | NM_001103355 | 20.26 | 54.83 | -2.7 | 2.83E-07 | 5.81E-06 |
| 654 | Down | *Colca2* | XM_006226396 | 15.72 | 38.21 | -2.4 | 2.85E-07 | 5.84E-06 |
| 655 | Up | *Top2a* | NM_022183 | 158.01 | 64.36 | 2.5 | 2.87E-07 | 5.88E-06 |
| 656 | Down | *Acer2* | NM_001107943 | 519.52 | 1215.94 | -2.3 | 2.97E-07 | 6.03E-06 |
| 657 | Up | *Muc4* | XM_006221167 | 162.22 | 19.10 | 8.5 | 3.06E-07 | 6.18E-06 |
| 658 | Up | *Mcpt1l1* | NM_001277668 | 261.66 | 18.03 | 14.5 | 3.15E-07 | 6.32E-06 |
| 659 | Down | *Sntn* | NM_001109098 | 26.97 | 64.49 | -2.4 | 3.16E-07 | 6.34E-06 |
| 660 | Down | *Cytl1* | NM_001134604 | 25.31 | 57.72 | -2.3 | 3.17E-07 | 6.35E-06 |
| 661 | Down | *Casc1* | NM_001106627 | 36.01 | 72.99 | -2.0 | 3.21E-07 | 6.39E-06 |
| 662 | Up | *Casc5* | NM_001170594 | 66.31 | 24.94 | 2.7 | 3.21E-07 | 6.39E-06 |
| 663 | Down | *LOC680885* | NM_001109431 | 35.53 | 110.43 | -3.1 | 3.21E-07 | 6.40E-06 |
| 664 | Down | *Zdhhc2* | NM_145096 | 133.47 | 299.08 | -2.2 | 3.23E-07 | 6.42E-06 |
| 665 | Up | *Sgcg* | NM_001006993 | 108.80 | 32.65 | 3.3 | 3.34E-07 | 6.59E-06 |
| 666 | Down | *Cyyr1* | NM_001013980 | 429.28 | 1176.94 | -2.7 | 3.39E-07 | 6.65E-06 |
| 667 | Down | *Rtkn2* | NM_001107625 | 148.58 | 908.56 | -6.1 | 3.51E-07 | 6.86E-06 |
| 668 | Up | *Ptn* | NM_017066 | 49.71 | 17.66 | 2.8 | 3.55E-07 | 6.93E-06 |
| 669 | Up | *Ccnd2* | NM_022267 | 716.42 | 347.17 | 2.1 | 3.63E-07 | 7.04E-06 |
| 670 | Down | *Akap2* | NM_001011974 | 493.46 | 1005.15 | -2.0 | 3.64E-07 | 7.05E-06 |
| 671 | Down | *Fam216b* | XM_008770086 | 20.39 | 46.96 | -2.3 | 3.64E-07 | 7.05E-06 |
| 672 | Up | *Trim29* | NM_001106815 | 188.08 | 16.38 | 11.5 | 3.67E-07 | 7.10E-06 |
| 673 | Up | *Slc16a6* | NM_198760 | 180.30 | 72.70 | 2.5 | 3.68E-07 | 7.11E-06 |
| 674 | Up | *Fblim1* | NM_001007554 | 259.68 | 118.49 | 2.2 | 3.72E-07 | 7.17E-06 |
| 675 | Down | *Sema6a* | XM_003751782 | 158.56 | 410.71 | -2.6 | 3.74E-07 | 7.20E-06 |
| 676 | Down | *LOC100361866* | XM_002728192 | 31.32 | 98.22 | -3.1 | 3.81E-07 | 7.30E-06 |
| 677 | Down | *Lrrc23* | NM_001013165 | 42.11 | 103.36 | -2.5 | 4.04E-07 | 7.67E-06 |
| 678 | Down | *Rgs13* | XM_008762407 | 18.42 | 70.38 | -3.8 | 4.09E-07 | 7.76E-06 |
| 679 | Down | *Cep126* | XM_006226292 | 30.93 | 63.76 | -2.1 | 4.16E-07 | 7.85E-06 |
| 680 | Up | *Cxcl11* | NM_182952 | 18.85 | 9.04 | 2.1 | 4.23E-07 | 7.97E-06 |
| 681 | Down | *Rp1* | NM_001195676 | 17.87 | 61.53 | -3.4 | 4.27E-07 | 8.02E-06 |
| 682 | Down | *Clic5* | NM_053603 | 1367.97 | 3397.48 | -2.5 | 4.27E-07 | 8.02E-06 |
| 683 | Down | *Sstr4* | NM_013036 | 37.35 | 93.22 | -2.5 | 4.35E-07 | 8.14E-06 |
| 684 | Down | *Reck* | NM_001107954 | 105.87 | 238.11 | -2.2 | 4.42E-07 | 8.25E-06 |
| 685 | Down | *Tnik* | NM_001106422 | 82.38 | 211.73 | -2.6 | 4.48E-07 | 8.35E-06 |
| 686 | Up | *Crabp2* | NM_017244 | 68.88 | 25.02 | 2.8 | 4.48E-07 | 8.35E-06 |
| 687 | Up | *Msr1* | NM_001191939 | 416.11 | 171.30 | 2.4 | 4.49E-07 | 8.36E-06 |
| 688 | Up | *Rcan1* | NM_153724 | 421.18 | 146.92 | 2.9 | 4.51E-07 | 8.39E-06 |
| 689 | Down | *Hba-a1* | NM_001013853 | 310.97 | 925.99 | -3.0 | 4.53E-07 | 8.41E-06 |
| 690 | Down | *Cfap99* | XM_008766340 | 39.51 | 94.94 | -2.4 | 4.53E-07 | 8.41E-06 |
| 691 | Up | *Calcb* | NM_138513 | 83.90 | 39.14 | 2.1 | 4.60E-07 | 8.52E-06 |
| 692 | Up | *Oasl* | NM_001009681 | 60.24 | 28.70 | 2.1 | 4.73E-07 | 8.72E-06 |
| 693 | Up | *Lcn2* | NM_130741 | 2399.75 | 664.48 | 3.6 | 4.80E-07 | 8.82E-06 |
| 694 | Down | *Klrd1* | NM_012745 | 36.13 | 77.85 | -2.2 | 4.82E-07 | 8.85E-06 |
| 695 | Up | *Cdk1* | NM_019296 | 66.30 | 32.53 | 2.0 | 4.91E-07 | 9.00E-06 |
| 696 | Down | *Rab6b* | NM_001108775 | 85.70 | 228.43 | -2.7 | 5.04E-07 | 9.20E-06 |
| 697 | Up | *Ect2* | NM_001108547 | 67.26 | 25.90 | 2.6 | 5.16E-07 | 9.38E-06 |
| 698 | Down | *Erg* | NM_133397 | 210.43 | 461.30 | -2.2 | 5.22E-07 | 9.47E-06 |
| 699 | Down | *Cdo1* | NM_052809 | 130.12 | 328.09 | -2.5 | 5.24E-07 | 9.51E-06 |
| 700 | Down | *Car4* | NM_019174 | 173.02 | 383.07 | -2.2 | 5.49E-07 | 9.88E-06 |
| 701 | Up | *Ugt1a5* | NM_001039549 | 140.94 | 64.09 | 2.2 | 5.62E-07 | 1.01E-05 |
| 702 | Up | *Tubb2b* | NM_001013886 | 70.88 | 21.63 | 3.3 | 5.67E-07 | 1.01E-05 |
| 703 | Down | *Tcf21* | NM_001032397 | 151.87 | 364.07 | -2.4 | 5.70E-07 | 1.02E-05 |
| 704 | Up | *Lmo1* | NM_139112 | 38.68 | 18.29 | 2.1 | 5.81E-07 | 1.04E-05 |
| 705 | Up | *Mmp12* | NM_053963 | 288.38 | 22.46 | 12.8 | 5.91E-07 | 1.05E-05 |
| 706 | Down | *Cfap74* | XM_008764399 | 20.86 | 45.17 | -2.2 | 6.00E-07 | 1.06E-05 |
| 707 | Down | *Akr1cl* | NM_001109900 | 462.41 | 1209.96 | -2.6 | 6.18E-07 | 1.09E-05 |
| 708 | Down | *Ppp1r16b* | NM_001191072 | 128.68 | 286.38 | -2.2 | 6.26E-07 | 1.10E-05 |
| 709 | Up | *Frzb* | NM_001100527 | 125.02 | 52.04 | 2.4 | 6.28E-07 | 1.10E-05 |
| 710 | Up | *Pln* | NM_022707 | 84.28 | 28.06 | 3.0 | 6.49E-07 | 1.13E-05 |
| 711 | Up | *Col5a1* | NM_134452 | 164.63 | 82.00 | 2.0 | 6.49E-07 | 1.13E-05 |
| 712 | Down | *Rsph1* | NM_001012176 | 23.20 | 47.31 | -2.0 | 6.62E-07 | 1.15E-05 |
| 713 | Down | *Rgs22* | XM_006241548 | 29.09 | 83.47 | -2.9 | 6.64E-07 | 1.16E-05 |
| 714 | Down | *Zmynd10* | NM_001004284 | 39.97 | 100.30 | -2.5 | 6.68E-07 | 1.16E-05 |
| 715 | Down | *Itpkb* | NM_019312 | 214.46 | 433.50 | -2.0 | 6.80E-07 | 1.18E-05 |
| 716 | Down | *Tnik* | XM_008760893 | 80.76 | 242.67 | -3.0 | 6.94E-07 | 1.20E-05 |
| 717 | Down | *Kcnn3* | NM_019315 | 52.88 | 116.72 | -2.2 | 6.96E-07 | 1.20E-05 |
| 718 | Down | *Ogn* | NM_001106103 | 552.28 | 1351.33 | -2.4 | 6.99E-07 | 1.20E-05 |
| 719 | Up | *Cfd* | NM_001077642 | 83.50 | 38.58 | 2.2 | 7.02E-07 | 1.21E-05 |
| 720 | Down | *Mir224* | NR_032111 | 8.69 | 20.00 | -2.3 | 7.04E-07 | 1.21E-05 |
| 721 | Down | *Dnah9* | XM_002724507 | 31.78 | 91.78 | -2.9 | 7.11E-07 | 1.22E-05 |
| 722 | Down | *Mlf1* | NM_001107680 | 55.18 | 122.53 | -2.2 | 7.36E-07 | 1.26E-05 |
| 723 | Down | *Sphkap* | NM_001127492 | 12.01 | 26.28 | -2.2 | 7.43E-07 | 1.26E-05 |
| 724 | Up | *Cks1l* | ENSRNOT00000052379 | 129.15 | 53.65 | 2.4 | 7.53E-07 | 1.28E-05 |
| 725 | Down | *Tmem204* | NM_001009620 | 235.84 | 493.07 | -2.1 | 7.54E-07 | 1.28E-05 |
| 726 | Down | *Ccnjl* | NM_001037773 | 41.17 | 85.59 | -2.1 | 8.03E-07 | 1.35E-05 |
| 727 | Down | *Cdkl5* | XM_006227325 | 141.16 | 344.88 | -2.4 | 8.07E-07 | 1.36E-05 |
| 728 | Down | *Nedd9* | NM_001011922 | 345.40 | 725.74 | -2.1 | 8.24E-07 | 1.37E-05 |
| 729 | Up | *Krt15* | NM_001004022 | 488.05 | 36.45 | 13.4 | 8.31E-07 | 1.38E-05 |
| 730 | Down | *Atp1a2* | AJ007485 | 17.33 | 38.25 | -2.2 | 8.32E-07 | 1.38E-05 |
| 731 | Up | *LOC683295* | ENSRNOT00000039417 | 103.92 | 36.70 | 2.8 | 8.42E-07 | 1.40E-05 |
| 732 | Down | *Ccna1* | NM_001011949 | 29.69 | 62.60 | -2.1 | 8.58E-07 | 1.42E-05 |
| 733 | Down | *Abcb1b* | NM_012623 | 312.81 | 735.47 | -2.4 | 8.73E-07 | 1.44E-05 |
| 734 | Up | *Mx1* | NM_001271058 | 207.45 | 67.97 | 3.1 | 8.81E-07 | 1.45E-05 |
| 735 | Down | *Cpn1* | NM_053526 | 46.26 | 142.81 | -3.1 | 8.88E-07 | 1.46E-05 |
| 736 | Down | *Gimap7* | NM_001024328 | 63.83 | 175.76 | -2.8 | 9.04E-07 | 1.47E-05 |
| 737 | Down | *Wdr78* | NM_001024786 | 40.06 | 86.09 | -2.1 | 9.12E-07 | 1.49E-05 |
| 738 | Up | *Isg15* | NM_001106700 | 95.70 | 40.52 | 2.4 | 9.19E-07 | 1.49E-05 |
| 739 | Down | *Pde8b* | NM_199268 | 45.06 | 91.51 | -2.0 | 9.20E-07 | 1.50E-05 |
| 740 | Down | *Ccdc30* | XM_001073839 | 41.06 | 83.34 | -2.0 | 9.41E-07 | 1.52E-05 |
| 741 | Down | *Klf2* | NM_001007684 | 195.61 | 410.86 | -2.1 | 9.51E-07 | 1.53E-05 |
| 742 | Up | *Stra6* | NM_001029924 | 62.71 | 30.43 | 2.1 | 9.70E-07 | 1.56E-05 |
| 743 | Down | *RGD1306739* | NM_001134576 | 30.32 | 71.02 | -2.3 | 9.84E-07 | 1.58E-05 |
| 744 | Down | *Ttc21a* | XM_003750604 | 17.29 | 35.74 | -2.1 | 1.01E-06 | 1.61E-05 |
| 745 | Up | *Bub1* | NM_001106507 | 39.29 | 18.61 | 2.1 | 1.01E-06 | 1.62E-05 |
| 746 | Down | *Frem2* | NM_001245978 | 65.92 | 154.11 | -2.3 | 1.01E-06 | 1.62E-05 |
| 747 | Down | *Cfap46* | XM_006223555 | 29.91 | 61.07 | -2.0 | 1.01E-06 | 1.62E-05 |
| 748 | Up | *A2m* | NM_012488 | 88.89 | 16.29 | 5.5 | 1.07E-06 | 1.69E-05 |
| 749 | Up | *Car13* | NM_001134993 | 315.47 | 50.89 | 6.2 | 1.08E-06 | 1.70E-05 |
| 750 | Down | *Lrguk* | NM_001106589 | 15.37 | 32.66 | -2.1 | 1.16E-06 | 1.81E-05 |
| 751 | Down | *Slc34a2* | NM_053380 | 1470.62 | 3013.37 | -2.0 | 1.17E-06 | 1.81E-05 |
| 752 | Down | *Plch1* | NM_001191707 | 34.12 | 70.05 | -2.1 | 1.18E-06 | 1.83E-05 |
| 753 | Down | *Cfap47* | XM_006227385 | 15.60 | 38.61 | -2.5 | 1.19E-06 | 1.85E-05 |
| 754 | Down | *Wdr66* | XM_008760397 | 23.59 | 58.52 | -2.5 | 1.22E-06 | 1.88E-05 |
| 755 | Up | *Ly86* | NM_001106128 | 122.47 | 53.37 | 2.3 | 1.22E-06 | 1.89E-05 |
| 756 | Up | *Cpz* | NM_031766 | 114.30 | 39.83 | 2.9 | 1.23E-06 | 1.90E-05 |
| 757 | Up | *Ncam1* | NM_031521 | 54.45 | 22.16 | 2.5 | 1.30E-06 | 1.99E-05 |
| 758 | Down | *Spib* | NM_001024286 | 17.03 | 37.20 | -2.2 | 1.34E-06 | 2.03E-05 |
| 759 | Up | *Ckmt1b* | NM_001012738 | 349.87 | 92.14 | 3.8 | 1.39E-06 | 2.09E-05 |
| 760 | Down | *Dnah3* | XM_006223470 | 15.05 | 32.68 | -2.2 | 1.42E-06 | 2.13E-05 |
| 761 | Up | *Padi4* | NM_017227 | 61.05 | 29.70 | 2.1 | 1.44E-06 | 2.16E-05 |
| 762 | Down | *Flrt3* | NM_001126291 | 249.29 | 637.11 | -2.6 | 1.45E-06 | 2.16E-05 |
| 763 | Down | *Samd12* | NM_001130562 | 44.81 | 89.83 | -2.0 | 1.47E-06 | 2.19E-05 |
| 764 | Up | *Mt1a* | NM_138826 | 565.41 | 161.75 | 3.5 | 1.48E-06 | 2.20E-05 |
| 765 | Down | *Cpn1* | NM_053526 | 32.05 | 70.76 | -2.2 | 1.49E-06 | 2.20E-05 |
| 766 | Down | *Cfap46* | XM_006223555 | 25.45 | 59.31 | -2.3 | 1.56E-06 | 2.30E-05 |
| 767 | Down | *Ect2l* | XM_006227648 | 19.80 | 49.53 | -2.5 | 1.58E-06 | 2.33E-05 |
| 768 | Down | *Gimap9* | NM_001008398 | 110.52 | 321.49 | -2.9 | 1.59E-06 | 2.33E-05 |
| 769 | Down | *Slc38a5* | NM_138854 | 29.34 | 71.89 | -2.5 | 1.62E-06 | 2.36E-05 |
| 770 | Up | *Dsg1* | XM_002725320 | 34.82 | 15.94 | 2.2 | 1.64E-06 | 2.39E-05 |
| 771 | Down | *Iqub* | NM_001034130 | 18.26 | 46.57 | -2.6 | 1.66E-06 | 2.42E-05 |
| 772 | Up | *Usp18* | NM_001014058 | 193.98 | 86.25 | 2.2 | 1.77E-06 | 2.56E-05 |
| 773 | Up | *Cep55* | NM_001025646 | 58.23 | 28.81 | 2.0 | 1.78E-06 | 2.57E-05 |
| 774 | Up | *Tfap2a* | XM_008771570 | 45.28 | 13.21 | 3.4 | 1.82E-06 | 2.62E-05 |
| 775 | Up | *Rgs18* | NM_001047084 | 157.69 | 76.05 | 2.1 | 1.88E-06 | 2.69E-05 |
| 776 | Up | *Ms4a4a* | XM_001075321 | 307.43 | 82.96 | 3.7 | 1.89E-06 | 2.70E-05 |
| 777 | Up | *Ptprz1* | NM_001170685 | 137.09 | 28.18 | 4.9 | 1.95E-06 | 2.76E-05 |
| 778 | Up | *Akr1b8* | NM_173136 | 83.16 | 23.88 | 3.5 | 1.95E-06 | 2.76E-05 |
| 779 | Up | *Ly49si1* | NM_001009497 | 83.68 | 26.01 | 3.2 | 1.96E-06 | 2.77E-05 |
| 780 | Down | *Plpp4* | NM_001191631 | 24.40 | 63.63 | -2.6 | 1.99E-06 | 2.81E-05 |
| 781 | Down | *Cdh5* | NM_001107407 | 676.35 | 1638.03 | -2.4 | 2.06E-06 | 2.89E-05 |
| 782 | Down | *Pde7b* | NM_080894 | 87.34 | 215.44 | -2.5 | 2.12E-06 | 2.96E-05 |
| 783 | Down | *Dpp4* | NM_012789 | 265.39 | 616.55 | -2.3 | 2.16E-06 | 3.00E-05 |
| 784 | Up | *Prim1* | NM_001008768 | 184.08 | 89.44 | 2.1 | 2.18E-06 | 3.03E-05 |
| 785 | Up | *Ankrd22* | NM_001191638 | 49.51 | 9.82 | 5.0 | 2.28E-06 | 3.12E-05 |
| 786 | Down | *Cyp2b1* | NM_001134844 | 500.50 | 2089.00 | -4.2 | 2.45E-06 | 3.32E-05 |
| 787 | Up | *Rspo3* | NM_001100990 | 32.10 | 9.55 | 3.4 | 2.46E-06 | 3.33E-05 |
| 788 | Up | *Gdf15* | NM_019216 | 101.93 | 41.49 | 2.5 | 2.52E-06 | 3.40E-05 |
| 789 | Down | *Sstr1* | NM_012719 | 16.48 | 33.97 | -2.1 | 2.55E-06 | 3.44E-05 |
| 790 | Down | *Susd2* | NM_001106381 | 148.63 | 411.63 | -2.8 | 2.56E-06 | 3.45E-05 |
| 791 | Up | *Nabp1* | NM_001014216 | 130.42 | 62.94 | 2.1 | 2.57E-06 | 3.46E-05 |
| 792 | Up | *Tmem59l* | NM_001271055 | 63.54 | 31.44 | 2.0 | 2.61E-06 | 3.50E-05 |
| 793 | Up | *Lilrb3* | XM_001070618 | 23.91 | 11.63 | 2.1 | 2.61E-06 | 3.50E-05 |
| 794 | Up | *Glb1l* | NM_001127529 | 179.25 | 83.05 | 2.2 | 2.63E-06 | 3.53E-05 |
| 795 | Up | *Cks2* | NM_001126083 | 28.75 | 14.03 | 2.0 | 2.68E-06 | 3.57E-05 |
| 796 | Down | *Ccdc17* | NM_001014067 | 75.98 | 196.20 | -2.6 | 2.75E-06 | 3.65E-05 |
| 797 | Up | *Dsc3* | NM_001107402 | 177.87 | 27.55 | 6.5 | 2.76E-06 | 3.67E-05 |
| 798 | Down | *Xpnpep2* | NM_057155 | 121.01 | 364.78 | -3.0 | 2.87E-06 | 3.79E-05 |
| 799 | Up | *Apoc1* | NM_001109996 | 36.30 | 16.64 | 2.2 | 2.89E-06 | 3.81E-05 |
| 800 | Down | *Btbd3* | NM_001107782 | 156.87 | 342.69 | -2.2 | 2.92E-06 | 3.83E-05 |
| 801 | Up | *Rbm3* | NM_053696 | 1374.68 | 525.22 | 2.6 | 2.92E-06 | 3.84E-05 |
| 802 | Down | *LOC100912942* | XR_589462 | 43.64 | 90.28 | -2.1 | 3.02E-06 | 3.95E-05 |
| 803 | Down | *Hmcn1* | NM_001271292 | 120.21 | 241.45 | -2.0 | 3.02E-06 | 3.95E-05 |
| 804 | Up | *Slfn4* | NM_053687 | 293.27 | 128.51 | 2.3 | 3.03E-06 | 3.97E-05 |
| 805 | Down | *Nrp1* | NM_145098 | 485.64 | 1119.15 | -2.3 | 3.05E-06 | 3.99E-05 |
| 806 | Up | *Fermt1* | NM_001106515 | 51.94 | 14.47 | 3.6 | 3.22E-06 | 4.18E-05 |
| 807 | Down | *Azgp1* | NM_012826 | 22.83 | 53.39 | -2.3 | 3.55E-06 | 4.53E-05 |
| 808 | Down | *Fam227a* | NM_001130581 | 24.23 | 69.39 | -2.9 | 3.66E-06 | 4.64E-05 |
| 809 | Up | *Nr1d1* | NM_001113422 | 144.21 | 56.34 | 2.6 | 3.83E-06 | 4.84E-05 |
| 810 | Down | *Riiad1* | NM_001144957 | 41.47 | 100.97 | -2.4 | 3.83E-06 | 4.84E-05 |
| 811 | Down | *Mir126* | NR_031871 | 9.03 | 22.53 | -2.5 | 3.93E-06 | 4.93E-05 |
| 812 | Down | *Pou2f3* | NM_001105745 | 18.17 | 38.34 | -2.1 | 4.01E-06 | 5.01E-05 |
| 813 | Up | *Aqp3* | NM_031703 | 228.63 | 33.01 | 6.9 | 4.02E-06 | 5.02E-05 |
| 814 | Up | *Retnla* | NM_053333 | 2315.07 | 632.28 | 3.7 | 4.03E-06 | 5.02E-05 |
| 815 | Down | *LOC501467* | XM_006227100 | 16.53 | 36.56 | -2.2 | 4.03E-06 | 5.02E-05 |
| 816 | Down | *LOC685989* | XM_001066079 | 16.53 | 36.56 | -2.2 | 4.03E-06 | 5.02E-05 |
| 817 | Up | *Cd163* | NM_001107887 | 133.45 | 26.05 | 5.1 | 4.27E-06 | 5.28E-05 |
| 818 | Down | *Aqp5* | NM_012779 | 275.98 | 785.88 | -2.8 | 4.28E-06 | 5.28E-05 |
| 819 | Down | *Cntn1* | NM_057118 | 44.87 | 110.98 | -2.5 | 4.32E-06 | 5.32E-05 |
| 820 | Down | *Cpvl* | NM_001029927 | 17.08 | 36.61 | -2.1 | 4.55E-06 | 5.55E-05 |
| 821 | Down | *Akap5* | NM_133515 | 650.05 | 1617.52 | -2.5 | 4.58E-06 | 5.58E-05 |
| 822 | Down | *Etv5* | NM_001107082 | 96.31 | 224.78 | -2.3 | 4.60E-06 | 5.60E-05 |
| 823 | Up | *Efna3* | XM_001072657 | 40.11 | 16.34 | 2.5 | 4.71E-06 | 5.72E-05 |
| 824 | Down | *Cadm1* | NM_001012201 | 202.75 | 508.16 | -2.5 | 4.80E-06 | 5.80E-05 |
| 825 | Down | *Aqp4* | NM_001142366 | 86.62 | 206.48 | -2.4 | 4.89E-06 | 5.91E-05 |
| 826 | Up | *Itga6* | NM_053725 | 478.23 | 231.49 | 2.1 | 4.99E-06 | 6.01E-05 |
| 827 | Up | *Tnn* | NM_001107189 | 32.18 | 10.74 | 3.0 | 5.12E-06 | 6.13E-05 |
| 828 | Down | *Csprs* | XM_006226911 | 15.61 | 31.79 | -2.0 | 5.14E-06 | 6.15E-05 |
| 829 | Up | *Fap* | NM_138850 | 19.39 | 7.93 | 2.4 | 5.14E-06 | 6.15E-05 |
| 830 | Down | *Arhgef15* | NM_001105789 | 99.73 | 219.99 | -2.2 | 5.20E-06 | 6.20E-05 |
| 831 | Down | *Ubxn10* | NM_001013093 | 44.01 | 115.59 | -2.6 | 5.38E-06 | 6.37E-05 |
| 832 | Down | *Dynlrb2* | NM_001108451 | 26.82 | 67.06 | -2.5 | 5.44E-06 | 6.43E-05 |
| 833 | Down | *Mir568* | NR_032745 | 52.32 | 105.56 | -2.0 | 5.65E-06 | 6.63E-05 |
| 834 | Down | *Dnai1* | NM_001024342 | 24.92 | 49.86 | -2.0 | 5.66E-06 | 6.64E-05 |
| 835 | Down | *Efhc1* | NM_001122947 | 48.96 | 99.21 | -2.0 | 5.90E-06 | 6.89E-05 |
| 836 | Down | *Vtn* | NM_019156 | 83.82 | 213.55 | -2.5 | 6.01E-06 | 6.99E-05 |
| 837 | Up | *Tmprss11b* | NM_001004020 | 67.06 | 11.42 | 5.9 | 6.10E-06 | 7.08E-05 |
| 838 | Down | *Vegfa* | NM_001110333 | 733.40 | 1560.97 | -2.1 | 6.15E-06 | 7.12E-05 |
| 839 | Up | *Clec2dl1* | NM_001085404 | 83.57 | 29.58 | 2.8 | 6.17E-06 | 7.14E-05 |
| 840 | Down | *Zfp945* | XM_008758834 | 11.30 | 23.31 | -2.1 | 6.24E-06 | 7.21E-05 |
| 841 | Down | *Lrriq1* | XM_008765345 | 30.13 | 69.76 | -2.3 | 6.27E-06 | 7.24E-05 |
| 842 | Down | *Ccdc146* | NM_001025044 | 14.38 | 31.83 | -2.2 | 6.35E-06 | 7.34E-05 |
| 843 | Up | *Fam83h* | NM_001130565 | 170.72 | 84.84 | 2.0 | 6.41E-06 | 7.39E-05 |
| 844 | Down | *Gabra1* | NM_183326 | 26.60 | 54.77 | -2.1 | 6.90E-06 | 7.84E-05 |
| 845 | Up | *Adgrg2* | NM_001270871 | 21.71 | 9.82 | 2.2 | 7.16E-06 | 8.10E-05 |
| 846 | Up | *Mt1a* | ENSRNOT00000057898 | 66.52 | 13.48 | 4.9 | 7.43E-06 | 8.35E-05 |
| 847 | Up | *Pbk* | NM_001079937 | 36.19 | 18.00 | 2.0 | 7.56E-06 | 8.48E-05 |
| 848 | Up | *Igfbp3* | NM_012588 | 548.74 | 218.02 | 2.5 | 7.59E-06 | 8.51E-05 |
| 849 | Up | *Serpinb11* | NM_001107167 | 56.88 | 11.86 | 4.8 | 7.66E-06 | 8.56E-05 |
| 850 | Up | *Cma1* | NM_013092 | 44.26 | 15.01 | 2.9 | 7.80E-06 | 8.67E-05 |
| 851 | Up | *Col11a1* | NM_013117 | 39.66 | 19.45 | 2.0 | 7.82E-06 | 8.68E-05 |
| 852 | Up | *Ephb6* | NM_001107857 | 67.18 | 25.88 | 2.6 | 7.93E-06 | 8.78E-05 |
| 853 | Down | *Maob* | NM_013198 | 105.55 | 293.60 | -2.8 | 8.00E-06 | 8.85E-05 |
| 854 | Up | *Sgol2* | NM_001271191 | 24.84 | 11.63 | 2.1 | 8.24E-06 | 9.03E-05 |
| 855 | Down | *Coro2b* | XM_001074062 | 58.72 | 124.39 | -2.1 | 8.42E-06 | 9.19E-05 |
| 856 | Down | *Pygo1* | NM_001191117 | 53.80 | 111.72 | -2.1 | 8.42E-06 | 9.19E-05 |
| 857 | Down | *Rmdn2* | NM_001037200 | 148.67 | 445.27 | -3.0 | 8.57E-06 | 9.33E-05 |
| 858 | Up | *Fat2* | NM_022954 | 63.94 | 17.77 | 3.6 | 8.75E-06 | 9.50E-05 |
| 859 | Down | *Prx* | NM_023976 | 62.78 | 134.86 | -2.1 | 8.86E-06 | 9.59E-05 |
| 860 | Up | *LOC682330* | XM_001061048 | 66.96 | 31.47 | 2.1 | 9.08E-06 | 9.78E-05 |
| 861 | Down | *Pcdh12* | NM_053944 | 59.72 | 146.88 | -2.5 | 9.14E-06 | 9.85E-05 |
| 862 | Up | *Ckap2* | NM_001169139 | 43.20 | 15.81 | 2.7 | 9.29E-06 | 9.98E-05 |
| 863 | Up | *Ccl20* | NM_019233 | 75.20 | 31.00 | 2.4 | 9.97E-06 | 0.0001 |
| 864 | Down | *Ccdc129* | NM_001191973 | 10.89 | 24.91 | -2.3 | 1.02E-05 | 0.0001 |
| 865 | Up | *Lypd3* | NM_021759 | 68.38 | 29.68 | 2.3 | 1.08E-05 | 0.0001 |
| 866 | Up | *RGD1565166* | NM_001105762 | 204.64 | 90.42 | 2.3 | 1.10E-05 | 0.0001 |
| 867 | Up | *Sema4g* | NM_001108526 | 111.43 | 42.97 | 2.6 | 1.10E-05 | 0.0001 |
| 868 | Down | *Gzma* | NM_153468 | 29.57 | 92.30 | -3.1 | 1.11E-05 | 0.0001 |
| 869 | Up | *Ltbp2* | NM_021586 | 373.64 | 136.20 | 2.7 | 1.12E-05 | 0.0001 |
| 870 | Down | *Tmem47* | NM_001109317 | 357.42 | 1006.27 | -2.8 | 1.12E-05 | 0.0001 |
| 871 | Down | *Slco4c1* | NM_001002024 | 127.16 | 360.74 | -2.8 | 1.21E-05 | 0.0001 |
| 872 | Up | *Elf3* | NM_001024768 | 160.40 | 54.48 | 2.9 | 1.31E-05 | 0.0001 |
| 873 | Down | *Dmc1* | NM_001130567 | 10.65 | 23.62 | -2.2 | 1.32E-05 | 0.0001 |
| 874 | Up | *Epha7* | NM_134331 | 103.49 | 42.96 | 2.4 | 1.36E-05 | 0.0001 |
| 875 | Down | *Plet1* | NM_001014209 | 350.12 | 816.27 | -2.3 | 1.36E-05 | 0.0001 |
| 876 | Up | *Zfp185* | XM_006229520 | 58.19 | 21.55 | 2.7 | 1.37E-05 | 0.0001 |
| 877 | Up | *Car3* | NM_019292 | 142.03 | 70.21 | 2.0 | 1.38E-05 | 0.0001 |
| 878 | Up | *Ccl12* | NM_001105822 | 80.63 | 16.72 | 4.8 | 1.42E-05 | 0.0001 |
| 879 | Up | *Galnt5* | NM_031796 | 66.64 | 23.75 | 2.8 | 1.47E-05 | 0.0001 |
| 880 | Up | *Gpr87* | NM_001107677 | 40.84 | 16.06 | 2.5 | 1.49E-05 | 0.0001 |
| 881 | Down | *Dnah3* | XM_006223470 | 25.83 | 52.41 | -2.0 | 1.51E-05 | 0.0002 |
| 882 | Down | *Efemp1* | NM_001012039 | 308.49 | 621.74 | -2.0 | 1.55E-05 | 0.0002 |
| 883 | Up | *Pax9* | NM_001039539 | 56.29 | 24.24 | 2.3 | 1.56E-05 | 0.0002 |
| 884 | Up | *Hist1h2ah* | NM_001315492 | 53.48 | 23.25 | 2.3 | 1.58E-05 | 0.0002 |
| 885 | Down | *LOC501346* | XR_597211 | 17.94 | 37.32 | -2.1 | 1.58E-05 | 0.0002 |
| 886 | Up | *Adam28* | NM_181693 | 57.89 | 21.44 | 2.7 | 1.64E-05 | 0.0002 |
| 887 | Down | *Enpp6* | NM_001107311 | 15.61 | 31.71 | -2.0 | 1.66E-05 | 0.0002 |
| 888 | Down | *LOC681325* | NM_001109439 | 54.84 | 114.99 | -2.1 | 1.66E-05 | 0.0002 |
| 889 | Up | *Ephb2* | NM_001127319 | 67.04 | 32.20 | 2.1 | 1.68E-05 | 0.0002 |
| 890 | Down | *Aldh1a1* | NM_022407 | 255.53 | 699.27 | -2.7 | 1.70E-05 | 0.0002 |
| 891 | Up | *Tns4* | NM_001024881 | 66.75 | 20.79 | 3.2 | 1.72E-05 | 0.0002 |
| 892 | Down | *Ar* | NM_012502 | 24.08 | 60.28 | -2.5 | 1.79E-05 | 0.0002 |
| 893 | Down | *Ooep* | XM_008757824 | 89.45 | 277.25 | -3.1 | 1.83E-05 | 0.0002 |
| 894 | Down | *Sox18* | NM_001024781 | 68.04 | 136.09 | -2.0 | 1.83E-05 | 0.0002 |
| 895 | Up | *Ms4a8* | NM_001108519 | 56.90 | 25.84 | 2.2 | 1.87E-05 | 0.0002 |
| 896 | Down | *Ccdc39* | NM_001107667 | 24.81 | 51.42 | -2.1 | 1.97E-05 | 0.0002 |
| 897 | Up | *Cox6a2* | NM_012812 | 132.00 | 65.17 | 2.0 | 2.03E-05 | 0.0002 |
| 898 | Down | *Thbd* | NM_031771 | 361.85 | 805.01 | -2.2 | 2.06E-05 | 0.0002 |
| 899 | Up | *Cdkn2a* | NM_031550 | 48.04 | 21.62 | 2.2 | 2.12E-05 | 0.0002 |
| 900 | Up | *Chi3l1* | NM_001309820 | 678.24 | 318.79 | 2.1 | 2.16E-05 | 0.0002 |
| 901 | Down | *LOC103691276* | XR_590567 | 47.07 | 114.27 | -2.4 | 2.20E-05 | 0.0002 |
| 902 | Up | *Slc2a1* | NM_138827 | 378.01 | 152.81 | 2.5 | 2.30E-05 | 0.0002 |
| 903 | Down | *LOC685989* | XM_001066079 | 28.95 | 69.69 | -2.4 | 2.33E-05 | 0.0002 |
| 904 | Down | *LOC685989* | XM_008758250 | 32.40 | 66.76 | -2.1 | 2.34E-05 | 0.0002 |
| 905 | Up | *Sprr1a* | NM_021864 | 137.19 | 17.95 | 7.6 | 2.37E-05 | 0.0002 |
| 906 | Up | *Capn8* | NM_133309 | 43.14 | 21.28 | 2.0 | 2.58E-05 | 0.0002 |
| 907 | Up | *Gjb2* | NM_001004099 | 103.20 | 42.47 | 2.4 | 2.59E-05 | 0.0002 |
| 908 | Down | *Fank1* | NM_001008347 | 21.49 | 43.11 | -2.0 | 2.88E-05 | 0.0003 |
| 909 | Down | *Capsl* | NM_001106417 | 36.79 | 77.44 | -2.1 | 3.00E-05 | 0.0003 |
| 910 | Up | *Col17a1* | NM_001106366 | 104.70 | 31.78 | 3.3 | 3.05E-05 | 0.0003 |
| 911 | Down | *Nkg7* | NM_133540 | 58.71 | 118.78 | -2.0 | 3.05E-05 | 0.0003 |
| 912 | Down | *Veph1* | NM_001014171 | 89.65 | 229.82 | -2.6 | 3.19E-05 | 0.0003 |
| 913 | Up | *Tfpi2* | NM_173141 | 50.80 | 23.56 | 2.2 | 3.24E-05 | 0.0003 |
| 914 | Up | *Bpifa1* | NM_172031 | 481.48 | 26.62 | 18.1 | 3.26E-05 | 0.0003 |
| 915 | Up | *Lgals7* | NM_022582 | 66.55 | 21.70 | 3.1 | 3.27E-05 | 0.0003 |
| 916 | Down | *LOC689453* | XM_006255043 | 32.22 | 65.66 | -2.0 | 3.27E-05 | 0.0003 |
| 917 | Up | *Slpi* | ENSRNOT00000076191 | 134.12 | 29.20 | 4.6 | 3.30E-05 | 0.0003 |
| 918 | Down | *Ccl5* | NM_031116 | 211.24 | 461.35 | -2.2 | 3.34E-05 | 0.0003 |
| 919 | Up | *Galnt12* | XM_001066416 | 62.11 | 25.59 | 2.4 | 3.35E-05 | 0.0003 |
| 920 | Up | *Angptl4* | NM_199115 | 91.59 | 30.33 | 3.0 | 3.50E-05 | 0.0003 |
| 921 | Down | *RGD1566226* | NM_001134515 | 9.86 | 30.00 | -3.0 | 3.58E-05 | 0.0003 |
| 922 | Down | *Bmp4* | NM_012827 | 289.30 | 651.60 | -2.3 | 3.75E-05 | 0.0003 |
| 923 | Down | *LOC685989* | XM_001066079 | 28.80 | 71.83 | -2.5 | 3.79E-05 | 0.0003 |
| 924 | Up | *Plek2* | NM_001114180 | 92.68 | 35.41 | 2.6 | 3.96E-05 | 0.0003 |
| 925 | Up | *Tmem154* | NM_001108553 | 190.44 | 94.27 | 2.0 | 4.16E-05 | 0.0004 |
| 926 | Up | *Tnnt2* | NM_012676 | 94.64 | 34.63 | 2.7 | 4.23E-05 | 0.0004 |
| 927 | Down | *Hhip* | NM_001191817 | 149.76 | 521.27 | -3.5 | 4.54E-05 | 0.0004 |
| 928 | Up | *Wfdc21* | NM_001003706 | 236.27 | 55.96 | 4.2 | 5.00E-05 | 0.0004 |
| 929 | Up | *Calml3* | NM_001012054 | 316.30 | 77.90 | 4.1 | 5.17E-05 | 0.0004 |
| 930 | Down | *Stard8* | NM_001107849 | 94.94 | 213.50 | -2.2 | 5.32E-05 | 0.0004 |
| 931 | Up | *Wnt7b* | NM_001009695 | 30.90 | 15.21 | 2.0 | 5.39E-05 | 0.0004 |
| 932 | Down | *LOC685989* | XM_001066079 | 28.88 | 70.82 | -2.5 | 5.47E-05 | 0.0004 |
| 933 | Up | *Tp63* | NM_001127339 | 47.40 | 16.38 | 2.9 | 5.74E-05 | 0.0005 |
| 934 | Down | *LOC685989* | XM_001066079 | 29.62 | 69.68 | -2.4 | 5.80E-05 | 0.0005 |
| 935 | Down | *Gimap4* | NM_173153 | 198.81 | 489.30 | -2.5 | 5.84E-05 | 0.0005 |
| 936 | Down | *Npw* | NM_153294 | 33.93 | 73.12 | -2.2 | 5.98E-05 | 0.0005 |
| 937 | Up | *Htr1b* | NM_022225 | 127.73 | 47.96 | 2.7 | 5.99E-05 | 0.0005 |
| 938 | Down | *LOC689453* | XM_006255043 | 34.14 | 77.51 | -2.3 | 6.19E-05 | 0.0005 |
| 939 | Down | *LOC685989* | XM_001066079 | 33.23 | 71.20 | -2.1 | 6.32E-05 | 0.0005 |
| 940 | Down | *LOC685989* | XM_001066079 | 28.91 | 66.75 | -2.3 | 6.36E-05 | 0.0005 |
| 941 | Down | *Gna14* | NM_001013151 | 45.32 | 120.30 | -2.7 | 6.41E-05 | 0.0005 |
| 942 | Down | *T* | NM_001106209 | 12.61 | 26.52 | -2.1 | 6.46E-05 | 0.0005 |
| 943 | Down | *LOC689453* | XM_006255043 | 26.99 | 71.39 | -2.6 | 6.50E-05 | 0.0005 |
| 944 | Down | *LOC685989* | XM_001066079 | 25.33 | 59.29 | -2.3 | 6.74E-05 | 0.0005 |
| 945 | Down | *LOC103690327* | XM_006227158 | 28.87 | 72.64 | -2.5 | 6.75E-05 | 0.0005 |
| 946 | Up | *S100a9* | NM_053587 | 308.50 | 104.73 | 2.9 | 6.86E-05 | 0.0005 |
| 947 | Down | *Baat* | NM_017300 | 9.54 | 19.14 | -2.0 | 6.88E-05 | 0.0005 |
| 948 | Down | *Nckap5* | XM_008761430 | 261.48 | 527.89 | -2.0 | 6.89E-05 | 0.0005 |
| 949 | Down | *LOC501349* | XM_008772459 | 26.59 | 63.56 | -2.4 | 7.35E-05 | 0.0006 |
| 950 | Up | *Spink5* | NM_001170606 | 106.13 | 25.78 | 4.1 | 7.45E-05 | 0.0006 |
| 951 | Up | *Clec4e* | NM_001005897 | 35.40 | 16.69 | 2.1 | 7.47E-05 | 0.0006 |
| 952 | Down | *Prf1* | NM_017330 | 82.85 | 237.49 | -2.9 | 7.89E-05 | 0.0006 |
| 953 | Down | *LOC689453* | XM_006255043 | 31.53 | 64.77 | -2.1 | 8.51E-05 | 0.0006 |
| 954 | Down | *Cfap100* | XM_006224986 | 13.66 | 35.26 | -2.6 | 8.67E-05 | 0.0006 |
| 955 | Up | *Cers3* | NM_001127561 | 21.42 | 10.27 | 2.1 | 8.76E-05 | 0.0007 |
| 956 | Up | *Fam110c* | NM_001025051 | 91.39 | 40.53 | 2.3 | 9.46E-05 | 0.0007 |
| 957 | Down | *Egfl6* | NM_001108254 | 88.33 | 269.15 | -3.0 | 9.54E-05 | 0.0007 |
| 958 | Up | *Fam83b* | NM_001108169 | 66.60 | 28.14 | 2.4 | 9.66E-05 | 0.0007 |
| 959 | Up | *Rgs4* | NM_017214 | 90.79 | 43.22 | 2.1 | 0.0001 | 0.0007 |
| 960 | Up | *Serpinb1a* | NM_001031642 | 439.93 | 168.95 | 2.6 | 0.0001 | 0.0008 |
| 961 | Down | *LOC685989* | XM_008758250 | 30.49 | 65.57 | -2.2 | 0.0001 | 0.0008 |
| 962 | Up | *Adgrd1* | XM_001070157 | 78.17 | 37.50 | 2.1 | 0.0001 | 0.0008 |
| 963 | Down | *LOC103691127* | XR_590209 | 17.55 | 42.61 | -2.4 | 0.0001 | 0.0008 |
| 964 | Down | *LOC501346* | XR_597211 | 30.98 | 63.81 | -2.1 | 0.0001 | 0.0009 |
| 965 | Down | *LOC501346* | XR_597211 | 30.98 | 63.81 | -2.1 | 0.0001 | 0.0009 |
| 966 | Up | *Cpa3* | NM_019300 | 280.38 | 63.09 | 4.4 | 0.0001 | 0.0009 |
| 967 | Down | *Myct1* | NM_001106207 | 96.00 | 192.49 | -2.0 | 0.0001 | 0.0009 |
| 968 | Up | *Krt23* | NM_001008753 | 65.93 | 31.54 | 2.1 | 0.0001 | 0.0009 |
| 969 | Up | *Tpsab1* | NM_019322 | 86.43 | 21.74 | 4.0 | 0.0001 | 0.0009 |
| 970 | Down | *LOC102551957* | XR_589430 | 17.94 | 42.04 | -2.3 | 0.0001 | 0.001 |
| 971 | Down | *LOC102551957* | XR_589430 | 17.94 | 42.04 | -2.3 | 0.0001 | 0.001 |
| 972 | Down | *LOC102551957* | XR_589430 | 17.94 | 42.04 | -2.3 | 0.0001 | 0.001 |
| 973 | Down | *LOC102551957* | XR_589430 | 17.94 | 42.04 | -2.3 | 0.0001 | 0.001 |
| 974 | Down | *LOC102551957* | XR_589430 | 17.94 | 42.04 | -2.3 | 0.0001 | 0.001 |
| 975 | Down | *LOC102551957* | XR_589430 | 17.94 | 42.04 | -2.3 | 0.0001 | 0.001 |
| 976 | Down | *LOC102551957* | XR_589430 | 17.94 | 42.04 | -2.3 | 0.0001 | 0.001 |
| 977 | Down | *LOC102551957* | XR_589430 | 17.94 | 42.04 | -2.3 | 0.0001 | 0.001 |
| 978 | Down | *LOC102551957* | XR_589430 | 17.94 | 42.04 | -2.3 | 0.0001 | 0.001 |
| 979 | Up | *Lgi2* | NM_001107219 | 55.82 | 17.54 | 3.2 | 0.0001 | 0.001 |
| 980 | Up | *Steap1* | NM_001106629 | 24.86 | 11.71 | 2.1 | 0.0001 | 0.001 |
| 981 | Up | *Krt16* | NM_001008752 | 38.24 | 11.33 | 3.4 | 0.0001 | 0.001 |
| 982 | Down | *LOC679711* | XM_006227121 | 23.91 | 50.32 | -2.1 | 0.0002 | 0.001 |
| 983 | Up | *Cyp4f39* | XM_006225942 | 63.49 | 30.66 | 2.1 | 0.0002 | 0.001 |
| 984 | Up | *Bpifb1* | NM_001077680 | 169.12 | 49.90 | 3.4 | 0.0002 | 0.001 |
| 985 | Down | *LOC501349* | XM_008772459 | 29.60 | 62.01 | -2.1 | 0.0002 | 0.001 |
| 986 | Up | *Ehf* | NM_001106493 | 271.11 | 132.24 | 2.1 | 0.0002 | 0.001 |
| 987 | Down | *Cyp2a3* | NM_012542 | 14.71 | 50.83 | -3.5 | 0.0002 | 0.001 |
| 988 | Up | *Dsel* | NM_001305281 | 177.71 | 68.42 | 2.6 | 0.0002 | 0.001 |
| 989 | Up | *Cx3cr1* | NM_133534 | 169.19 | 81.98 | 2.1 | 0.0002 | 0.001 |
| 990 | Up | *Fkbp5* | NM_001012174 | 162.31 | 78.51 | 2.1 | 0.0002 | 0.001 |
| 991 | Up | *Sele* | NM_138879 | 46.47 | 19.27 | 2.4 | 0.0002 | 0.002 |
| 992 | Up | *Pinlyp* | NM_001107488 | 110.19 | 46.77 | 2.4 | 0.0003 | 0.002 |
| 993 | Up | *LOC691352* | NM_001109638 | 37.25 | 14.35 | 2.6 | 0.0003 | 0.002 |
| 994 | Up | *Rnase2* | NM_001007015 | 43.66 | 12.63 | 3.5 | 0.0003 | 0.002 |
| 995 | Down | *LOC685989* | XM_001066079 | 23.28 | 46.90 | -2.0 | 0.0004 | 0.002 |
| 996 | Up | *Ocm2* | NM_012995 | 62.46 | 26.92 | 2.3 | 0.0004 | 0.002 |
| 997 | Down | *LOC681069* | XM_006249044 | 120.44 | 246.00 | -2.0 | 0.0004 | 0.002 |
| 998 | Up | *Il1r2* | NM_053953 | 62.11 | 22.96 | 2.7 | 0.0004 | 0.002 |
| 999 | Up | *Krt4* | NM_001008806 | 135.46 | 31.53 | 4.3 | 0.0004 | 0.002 |
| 1000 | Up | *Ms4a2* | NM_012845 | 28.72 | 12.74 | 2.3 | 0.0004 | 0.002 |
| 1001 | Up | *Cldn1* | NM_031699 | 73.84 | 29.49 | 2.5 | 0.0005 | 0.003 |
| 1002 | Up | *Reg3b* | NM_053289 | 18.05 | 6.31 | 2.9 | 0.0005 | 0.003 |
| 1003 | Up | *Clec4d* | NM_001003707 | 73.01 | 36.35 | 2.0 | 0.0005 | 0.003 |
| 1004 | Up | *S100a8* | NM_053822 | 97.36 | 35.47 | 2.7 | 0.0006 | 0.003 |
| 1005 | Down | *Lrp2* | NM_030827 | 93.83 | 232.56 | -2.5 | 0.0006 | 0.003 |
| 1006 | Up | *Pla2g2a* | NM_031598 | 340.64 | 92.53 | 3.7 | 0.0007 | 0.004 |
| 1007 | Up | *Mcpt1l1* | NM_001277668 | 97.02 | 36.47 | 2.7 | 0.0008 | 0.004 |
| 1008 | Down | *Dlk1* | NM_053744 | 43.66 | 101.14 | -2.3 | 0.0008 | 0.004 |
| 1009 | Down | *Scd1* | NM_139192 | 264.22 | 701.58 | -2.7 | 0.0009 | 0.005 |
| 1010 | Up | *Ighg* | BC088271 | 170.12 | 52.87 | 3.2 | 0.001 | 0.005 |
| 1011 | Down | *Aplnr* | NM_031349 | 213.25 | 449.94 | -2.1 | 0.001 | 0.006 |
| 1012 | Up | *Amtn* | NM_001044296 | 62.35 | 31.17 | 2.0 | 0.001 | 0.006 |
| 1013 | Down | *S100g* | NM_012521 | 224.95 | 474.75 | -2.1 | 0.002 | 0.008 |
| 1014 | Up | *Sprr3* | NM_001107686 | 66.28 | 23.20 | 2.9 | 0.002 | 0.008 |
| 1015 | Down | *LOC685767* | XM_008768748 | 84.11 | 207.27 | -2.5 | 0.002 | 0.008 |
| 1016 | Up | *Scel* | NM_001108388 | 86.18 | 40.44 | 2.1 | 0.003 | 0.013 |
| 1017 | Up | *Clip4* | NM_001013942 | 32.83 | 15.02 | 2.2 | 0.003 | 0.013 |
| 1018 | Up | *Krt13* | NM_001004021 | 103.08 | 45.07 | 2.3 | 0.003 | 0.014 |
| 1019 | Down | *Gpm6a* | NM_178105 | 147.55 | 342.87 | -2.3 | 0.003 | 0.014 |
| 1020 | Up | *Reg3g* | NM_173097 | 42.09 | 17.25 | 2.4 | 0.004 | 0.015 |
| 1021 | Down | *LOC689064* | NM_001111269 | 304.12 | 610.70 | -2.0 | 0.005 | 0.019 |
| 1022 | Up | *Ces2g* | NM_001106175 | 45.77 | 21.35 | 2.1 | 0.005 | 0.020 |
| 1023 | Up | *Tpsb2* | NM_019180 | 100.54 | 46.34 | 2.2 | 0.005 | 0.020 |
| 1024 | Down | *LOC685767* | XM_001065185 | 7.13 | 16.50 | -2.3 | 0.005 | 0.021 |

**Supplementary table S6.** Overlapping genes (*P_FDR_* < 0.05) of humidifier disinfectant-associated lung injury in human and PHMG-induced lung fibrosis in rat

| No | Gene | Human | | | | | |  | | Rat | | | | | |
| --- | --- | --- | --- | --- | --- | --- | --- | --- | --- | --- | --- | --- | --- | --- | --- |
|  |  | mRNA  accession ID | Expression level (average) | | Fold change  (case/control) | *P_FDR_* |  | | mRNA  accession ID | | Expression level  (average) | | Fold change  (case/control) | *P_FDR_* |  |
|  |  |  | Cases  (n = 9) | Unaffected controls  (n = 10) |  |  |  | |  |  | Cases  (n = 10) | Controls  (n = 10) |  |  |  |
| 1 | *COL1A2* | NM_000089 | 25.31 | 9.82 | 2.6 | 0.04 |  | | NM_053356 | | 1208.74 | 495.22 | 2.4 | 4.71×10^-7^ |  |
| 2 | *BASP1* | NM_001271606 | 26.35 | 10.14 | 2.6 | 0.01 |  | | NM_022300 | | 286.95 | 135.40 | 2.1 | 2.37×10^-7^ |  |
| 3 | *MMP2* | NM_001127891 | 20.34 | 6.14 | 3.3 | 0.01 |  | | NM_031054 | | 1134.02 | 398.26 | 2.8 | 5.61×10^-8^ |  |
| 4 | *SERPINF1* | NM_002615 | 10.62 | 4.40 | 2.4 | 0.01 |  | | NM_177927 | | 455.48 | 61.55 | 7.4 | 1.06×10^-10^ |  |
| 5 | *LUM* | NM_002345 | 10.71 | 5.31 | 2.0 | 0.01 |  | | NM_031050 | | 118.56 | 32.66 | 3.6 | 1.67×10^-7^ |  |
| 6 | *TMSB10* | NM_021103 | 615.43 | 194.90 | 3.2 | 0.03 |  | | NM_021261 | | 179.53 | 85.08 | 2.1 | 1.92×10^-9^ |  |
| 7 | *A2M* | NM_000014 | 53.56 | 16.66 | 3.2 | 0.03 |  | | NM_012488 | | 88.89 | 16.29 | 5.5 | 1.69×10^-5^ |  |
| 8 | *MT2A* | NM_005953 | 75.07 | 23.28 | 3.2 | 0.04 |  | | ENSRNOT00000067391 | | 114.18 | 9.37 | 12.2 | 2.72×10^-6^ |  |

**Supplementary table S7.** List of top networks and their respective scores of human and rat gene expressions using IPA program

| Human/Rat | Top networks | Score | Number of focus molecules |
| --- | --- | --- | --- |
| Human | Developmental Disorder, Hereditary Disorder, Metabolic Disease (I) | 33 | 18 |
|  | Developmental Disorder, Hereditary Disorder, Metabolic Disease (II) | 28 | 16 |
|  | Cellular Development, Cellular Growth and Proliferation, Hematological System Development and Function | 26 | 15 |
|  | Dermatological Diseases and Conditions, Inflammatory Disease, Organismal Injury and Abnormalities | 24 | 14 |
|  | Cancer, Hereditary Disorder, Organismal Injury and Abnormalities | 21 | 13 |
|  | Cancer, Organismal Injury and Abnormalities, Respiratory Disease | 17 | 11 |
|  | Cell Morphology, Cellular Assembly and Organization, Cellular Compromise | 15 | 10 |
|  |  |  |  |
| Rat | Cell Signaling, Cell-To-Cell Signaling and Interaction, Nucleic Acid Metabolism | 37 | 30 |
|  | Tissue Development, Cancer, Organismal Injury and Abnormalities | 31 | 27 |
|  | Dermatological Diseases and Conditions, Organismal Injury and Abnormalities, Developmental Disorder | 31 | 27 |
|  | Cell Cycle, Cellular Assembly and Organization, DNA Replication, Recombination, and Repair | 31 | 27 |
|  | Cell Cycle, Tissue Morphology, Cardiovascular System Development and Function | 27 | 25 |
|  | Cancer, Connective Tissue Disorders, Organismal Injury and Abnormalities | 27 | 25 |
|  | Connective Tissue Disorders, Hematological Disease, Organismal Injury and Abnormalities | 27 | 25 |
|  | Amino Acid Metabolism, Small Molecule Biochemistry, Cancer | 27 | 25 |
|  | Cardiovascular Disease, Heart Failure, Organismal Injury and Abnormalities | 25 | 24 |
|  | Cell Morphology, Reproductive System Development and Function, Molecular Transport | 25 | 24 |
|  | Cell-To-Cell Signaling and Interaction, Cellular Movement, Immune Cell Trafficking | 25 | 24 |
|  | Connective Tissue Development and Function, Tissue Development, Organismal Functions | 25 | 24 |
|  | Developmental Disorder, Hereditary Disorder, Ophthalmic Disease | 22 | 22 |
|  | Ophthalmic Disease, Organismal Injury and Abnormalities, Developmental Disorder | 22 | 22 |
|  | Cancer, Endocrine System Disorders, Organismal Injury and Abnormalities | 22 | 22 |
|  | Drug Metabolism, Vitamin and Mineral Metabolism, Lipid Metabolism | 21 | 21 |
|  | Immunological Disease, Developmental Disorder, Neurological Disease | 21 | 21 |
|  | Embryonic Development, Hematological System Development and Function, Lymphoid Tissue Structure and Development | 21 | 21 |
|  | DNA Replication, Recombination, and Repair, Cancer, Gastrointestinal Disease | 21 | 21 |
|  | Cellular Movement, Reproductive System Development and Function, Tissue Morphology | 21 | 21 |
|  | Developmental Disorder, Hereditary Disorder, Immunological Disease | 19 | 20 |
|  | Drug Metabolism, Small Molecule Biochemistry, Lipid Metabolism | 19 | 20 |
|  | Cancer, Cellular Development, Cellular Growth and Proliferation | 17 | 19 |
|  | Cardiac Arrythmia, Cardiovascular Disease, Organismal Injury and Abnormalities | 17 | 19 |
|  | Carbohydrate Metabolism, Small Molecule Biochemistry, Connective Tissue Development and Function | 17 | 19 |

**Supplementary table S8.** List of top canonical pathways of human and rat gene expressions using IPA program

| Human/Rat | Top canonical pathway | *P*-value |
| --- | --- | --- |
| Human | Oxidative Phosphorylation | 5.89E-08 |
|  | Mitochondrial Dysfunction | 1.51E-07 |
|  | Hepatic Fibrosis / Hepatic Stellate Cell Activation | 0.0003 |
|  | Sirtuin Signaling Pathway | 0.0006 |
|  | Hematopoiesis from Pluripotent Stem Cells | 0.002 |
|  | Primary Immunodeficiency Signaling | 0.002 |
|  | Acute Phase Response Signaling | 0.01 |
|  | B Cell Development | 0.01 |
|  | Diphthamide Biosynthesis | 0.01 |
|  | Inhibition of Matrix Metalloproteases | 0.02 |
|  | Intrinsic Prothrombin Activation Pathway | 0.02 |
|  | Spermine and Spermidine Degradation I | 0.02 |
|  | Actin Cytoskeleton Signaling | 0.03 |
|  | GP6 Signaling Pathway | 0.03 |
|  | Cancer Drug Resistance By Drug Efflux | 0.03 |
|  | Actin Nucleation by ARP-WASP Complex | 0.04 |
|  | Airway Pathology in Chronic Obstructive Pulmonary Disease | 0.04 |
|  | Inositol Pyrophosphates Biosynthesis | 0.04 |
|  | Remodeling of Epithelial Adherens Junctions | 0.04 |
|  |  |  |
| Rat | Hepatic Fibrosis / Hepatic Stellate Cell Activation | 3.16E-12 |
|  | Agranulocyte Adhesion and Diapedesis | 6.03E-08 |
|  | Complement System | 1.29E-07 |
|  | Atherosclerosis Signaling | 1.55E-07 |
|  | Granulocyte Adhesion and Diapedesis | 9.77E-07 |
|  | Inhibition of Matrix Metalloproteases | 2.19E-06 |
|  | Axonal Guidance Signaling | 4.07E-05 |
|  | Neuroprotective Role of THOP1 in Alzheimer's Disease | 8.91E-05 |
|  | Human Embryonic Stem Cell Pluripotency | 0.0002 |
|  | Phagosome Formation | 0.0002 |
|  | Pathogenesis of Multiple Sclerosis | 0.0003 |
|  | Glucocorticoid Receptor Signaling | 0.0003 |
|  | cAMP-mediated signaling | 0.0003 |
|  | Osteoarthritis Pathway | 0.0003 |
|  | Iron homeostasis signaling pathway | 0.0004 |
|  | Aryl Hydrocarbon Receptor Signaling | 0.0005 |
|  | Cell Cycle: G2/M DNA Damage Checkpoint Regulation | 0.0008 |
|  | GP6 Signaling Pathway | 0.001 |
|  | G-Protein Coupled Receptor Signaling | 0.001 |
|  | Role of Osteoblasts, Osteoclasts and Chondrocytes in Rheumatoid Arthritis | 0.001 |
|  | Antioxidant Action of Vitamin C | 0.001 |
|  | Role of IL-17A in Psoriasis | 0.001 |
|  | Cyclins and Cell Cycle Regulation | 0.001 |
|  | Leukocyte Extravasation Signaling | 0.002 |
|  | Germ Cell-Sertoli Cell Junction Signaling | 0.002 |
|  | Coagulation System | 0.002 |
|  | Gap Junction Signaling | 0.003 |
|  | Pancreatic Adenocarcinoma Signaling | 0.003 |
|  | Phospholipases | 0.003 |
|  | Antiproliferative Role of TOB in T Cell Signaling | 0.003 |
|  | Role of Pattern Recognition Receptors in Recognition of Bacteria and Viruses | 0.003 |
|  | Neuroinflammation Signaling Pathway | 0.004 |
|  | VDR/RXR Activation | 0.004 |
|  | eNOS Signaling | 0.004 |
|  | Wnt/β-catenin Signaling | 0.004 |
|  | Cardiac β-adrenergic Signaling | 0.004 |
|  | Role of NANOG in Mammalian Embryonic Stem Cell Pluripotency | 0.005 |
|  | Gαq Signaling | 0.005 |
|  | GADD45 Signaling | 0.006 |
|  | GDP-glucose Biosynthesis | 0.006 |
|  | Adrenomedullin signaling pathway | 0.007 |
|  | Basal Cell Carcinoma Signaling | 0.008 |
|  | MSP-RON Signaling Pathway | 0.008 |
|  | STAT3 Pathway | 0.008 |
|  | Glucose and Glucose-1-phosphate Degradation | 0.008 |
|  | Ascorbate Recycling (Cytosolic) | 0.009 |
|  | Crosstalk between Dendritic Cells and Natural Killer Cells | 0.009 |
|  | HMGB1 Signaling | 0.01 |
|  | Glioma Invasiveness Signaling | 0.01 |
|  | Relaxin Signaling | 0.01 |
|  | Factors Promoting Cardiogenesis in Vertebrates | 0.01 |
|  | TGF-β Signaling | 0.01 |
|  | Endothelin-1 Signaling | 0.01 |
|  | Bladder Cancer Signaling | 0.01 |
|  | ILK Signaling | 0.01 |
|  | p53 Signaling | 0.01 |
|  | Choline Biosynthesis III | 0.01 |
|  | Trehalose Degradation II (Trehalase) | 0.01 |
|  | Nicotine Degradation II | 0.01 |
|  | Integrin Signaling | 0.02 |
|  | Nitric Oxide Signaling in the Cardiovascular System | 0.02 |
|  | Cellular Effects of Sildenafil (Viagra) | 0.02 |
|  | IL-17A Signaling in Gastric Cells | 0.02 |
|  | Bupropion Degradation | 0.02 |
|  | IL-8 Signaling | 0.02 |
|  | BMP signaling pathway | 0.02 |
|  | Estrogen-mediated S-phase Entry | 0.02 |
|  | Superpathway of Melatonin Degradation | 0.02 |
|  | LXR/RXR Activation | 0.02 |
|  | Vitamin-C Transport | 0.02 |
|  | Intrinsic Prothrombin Activation Pathway | 0.03 |
|  | tRNA Splicing | 0.03 |
|  | Gαi Signaling | 0.03 |
|  | Colorectal Cancer Metastasis Signaling | 0.03 |
|  | Molecular Mechanisms of Cancer | 0.03 |
|  | Role of Hypercytokinemia/hyperchemokinemia in the Pathogenesis of Influenza | 0.03 |
|  | Superpathway of Inositol Phosphate Compounds | 0.03 |
|  | Acetone Degradation I (to Methylglyoxal) | 0.03 |
|  | Sonic Hedgehog Signaling | 0.03 |
|  | Differential Regulation of Cytokine Production in Macrophages and T Helper Cells by IL-17A and IL-17F | 0.03 |
|  | LPS/IL-1 Mediated Inhibition of RXR Function | 0.03 |
|  | Sertoli Cell-Sertoli Cell Junction Signaling | 0.03 |
|  | Mouse Embryonic Stem Cell Pluripotency | 0.04 |
|  | Glioblastoma Multiforme Signaling | 0.04 |
|  | Histamine Degradation | 0.04 |
|  | DNA damage-induced 14-3-3σ Signaling | 0.04 |
|  | Epithelial Adherens Junction Signaling | 0.04 |
|  | Clathrin-mediated Endocytosis Signaling | 0.04 |
|  | Asparagine Biosynthesis I | 0.04 |
|  | Adipogenesis pathway | 0.04 |
|  | Antiproliferative Role of Somatostatin Receptor 2 | 0.04 |
|  | Cardiomyocyte Differentiation via BMP Receptors | 0.04 |
|  | Melatonin Degradation I | 0.04 |

**Supplementary table S9.** List of top diseases or bio-functions of human and rat gene expressions using IPA program

| Human/Rat | Categories | Diseases or Functions Annotation | *P*-value |
| --- | --- | --- | --- |
| Human | Cancer, Endocrine System Disorders, Organismal Injury and Abnormalities | Thyroid gland tumor | 1.04.E-07 |
|  | Endocrine System Disorders, Organismal Injury and Abnormalities | Hyperplastic thyroid nodule | 1.11.E-07 |
|  | Cancer, Organismal Injury and Abnormalities | Solid tumor | 1.18.E-07 |
|  | Cancer, Organismal Injury and Abnormalities | Non-melanoma solid tumor | 1.56.E-07 |
|  | Cancer, Endocrine System Disorders, Organismal Injury and Abnormalities | Thyroid gland nonmedullary carcinoma | 2.71.E-07 |
|  | Cancer, Organismal Injury and Abnormalities | Epithelial neoplasm | 2.89.E-07 |
|  | Cancer, Organismal Injury and Abnormalities | Nonhematologic malignant neoplasm | 3.22.E-07 |
|  | Cancer, Endocrine System Disorders, Organismal Injury and Abnormalities | Differentiated thyroid cancer | 3.25.E-07 |
|  | Cancer, Organismal Injury and Abnormalities | Carcinoma | 3.84.E-07 |
|  | Cancer, Organismal Injury and Abnormalities | Tumorigenesis of tissue | 3.85.E-07 |
|  | Cancer, Organismal Injury and Abnormalities, Respiratory Disease | Stage I metastatic non-small cell lung carcinoma | 4.42.E-07 |
|  | Cancer, Organismal Injury and Abnormalities | Extracranial solid tumor | 5.25.E-07 |
|  | Cancer, Organismal Injury and Abnormalities | Non-hematological solid tumor | 6.66.E-07 |
|  | Cancer, Organismal Injury and Abnormalities | Malignant solid tumor | 8.05.E-07 |
|  | Cancer, Organismal Injury and Abnormalities | Cancer | 8.13.E-07 |
|  | Developmental Disorder, Organismal Injury and Abnormalities | Dystrophy | 8.97.E-07 |
|  | Cancer, Organismal Injury and Abnormalities | Malignant genitourinary solid tumor | 1.19.E-06 |
|  | Endocrine System Disorders, Organismal Injury and Abnormalities | Thyroid nodule | 1.80.E-06 |
|  | Cancer, Endocrine System Disorders, Organismal Injury and Abnormalities | Papillary thyroid carcinoma | 2.22.E-06 |
|  | Cancer, Endocrine System Disorders, Organismal Injury and Abnormalities | Thyroid cancer | 2.96.E-06 |
|  | Cancer, Organismal Injury and Abnormalities | Genitourinary tumor | 3.08.E-06 |
|  | Hereditary Disorder, Organismal Injury and Abnormalities, Skeletal and Muscular Disorders | Hereditary myopathy | 3.24.E-06 |
|  | Cancer, Endocrine System Disorders, Organismal Injury and Abnormalities | Primary thyroid gland neoplasm | 3.82.E-06 |
|  | Developmental Disorder, Hereditary Disorder, Organismal Injury and Abnormalities, Skeletal and Muscular Disorders | Dystrophy of muscle | 3.86.E-06 |
|  | Developmental Disorder, Hereditary Disorder, Organismal Injury and Abnormalities, Skeletal and Muscular Disorders | Duchenne muscular dystrophy | 3.89.E-06 |
|  | Connective Tissue Disorders, Inflammatory Disease, Organismal Injury and Abnormalities, Skeletal and Muscular Disorders | Rheumatic Disease | 4.81.E-06 |
|  | Cancer, Organismal Injury and Abnormalities | Abdominal carcinoma | 5.00.E-06 |
|  | Cancer, Organismal Injury and Abnormalities | Head and neck adenocarcinoma | 5.38.E-06 |
|  | Cancer, Organismal Injury and Abnormalities | Breast or pancreatic cancer | 6.21.E-06 |
|  | Organismal Injury and Abnormalities | Nodule | 6.49.E-06 |
|  | Cancer, Organismal Injury and Abnormalities | Adenocarcinoma | 6.54.E-06 |
|  | Cancer, Organismal Injury and Abnormalities | Neoplasia of melanoma cell lines | 8.26.E-06 |
|  | Developmental Disorder, Hereditary Disorder, Organismal Injury and Abnormalities, Skeletal and Muscular Disorders | Autosomal dominant Emery-Dreifuss muscular dystrophy | 8.35.E-06 |
|  | Dermatological Diseases and Conditions, Inflammatory Disease, Organismal Injury and Abnormalities, Skeletal and Muscular Disorders | Juvenile dermatomyositis | 8.35.E-06 |
|  | Cancer, Organismal Injury and Abnormalities | Breast or colorectal cancer | 8.56.E-06 |
|  | Cancer, Organismal Injury and Abnormalities, Reproductive System Disease | Breast or ovarian cancer | 1.54.E-05 |
|  | Cancer, Organismal Injury and Abnormalities | Secondary tumor | 1.85.E-05 |
|  | Cancer, Organismal Injury and Abnormalities | Metastasis of melanoma cell lines | 2.14.E-05 |
|  | Cancer, Organismal Injury and Abnormalities | Papillary adenocarcinoma | 2.16.E-05 |
|  | Cardiovascular Disease, Organismal Injury and Abnormalities | Rupture of heart ventricle | 2.33.E-05 |
|  | Cancer, Organismal Injury and Abnormalities | Metastatic solid tumor | 2.73.E-05 |
|  | Cancer, Organismal Injury and Abnormalities, Respiratory Disease | Advanced lung tumor | 2.80.E-05 |
|  | Cancer, Organismal Injury and Abnormalities | Advanced malignant tumor | 2.85.E-05 |
|  | Cancer, Endocrine System Disorders, Organismal Injury and Abnormalities | Follicular thyroid tumor | 3.01.E-05 |
|  | Cancer, Organismal Injury and Abnormalities | Head and neck carcinoma | 3.39.E-05 |
|  | Dermatological Diseases and Conditions, Inflammatory Disease, Organismal Injury and Abnormalities, Skeletal and Muscular Disorders | Dermatomyositis | 3.46.E-05 |
|  | Cancer, Organismal Injury and Abnormalities | Advanced malignant solid tumor | 3.74.E-05 |
|  | Cancer, Organismal Injury and Abnormalities | Abdominal neoplasm | 3.84.E-05 |
|  | Immunological Disease | Systemic autoimmune syndrome | 4.41.E-05 |
|  | Cancer, Organismal Injury and Abnormalities | Ductal carcinoma | 4.73.E-05 |
|  | Cancer, Organismal Injury and Abnormalities, Reproductive System Disease | Breast cancer | 5.76.E-05 |
|  | Cellular Development, Cellular Growth and Proliferation | Cell proliferation of tumor cell lines | 6.20.E-05 |
|  | Cancer, Organismal Injury and Abnormalities | Abdominal cancer | 6.49.E-05 |
|  | Cellular Assembly and Organization, Cellular Function and Maintenance, Protein Trafficking | Organization of F-actin | 6.97.E-05 |
|  | Cancer, Organismal Injury and Abnormalities | Early stage solid tumor | 7.56.E-05 |
|  | Cancer, Organismal Injury and Abnormalities, Reproductive System Disease | Ductal breast carcinoma | 7.74.E-05 |
|  | Cellular Movement | Migration of sarcoma cell lines | 9.99.E-05 |
|  |  |  |  |
| Rat | Cancer, Organismal Injury and Abnormalities | Non-hematological solid tumor | 7.42E-52 |
|  | Cancer, Organismal Injury and Abnormalities | Tumorigenesis of tissue | 5.47E-51 |
|  | Cancer, Organismal Injury and Abnormalities | Cancer | 1.31E-50 |
|  | Cancer, Organismal Injury and Abnormalities | Solid tumor | 3.64E-50 |
|  | Cancer, Organismal Injury and Abnormalities | Malignant solid tumor | 1.19E-49 |
|  | Cancer, Organismal Injury and Abnormalities | Epithelial neoplasm | 3.19E-49 |
|  | Cancer, Organismal Injury and Abnormalities | Abdominal cancer | 5.59E-47 |
|  | Cancer, Organismal Injury and Abnormalities | Non-melanoma solid tumor | 7.19E-47 |
|  | Cancer, Organismal Injury and Abnormalities | Abdominal neoplasm | 4.08E-46 |
|  | Cancer, Organismal Injury and Abnormalities | Extracranial solid tumor | 1.78E-45 |
|  | Cellular Movement | Cell movement | 7.16E-44 |
|  | Cellular Movement | Migration of cells | 2.59E-42 |
|  | Cancer, Organismal Injury and Abnormalities | Nonhematologic malignant neoplasm | 4.06E-42 |
|  | Cancer, Organismal Injury and Abnormalities | Carcinoma | 1.06E-41 |
|  | Cancer, Organismal Injury and Abnormalities | Malignant genitourinary solid tumor | 2.06E-41 |
|  | Cancer, Organismal Injury and Abnormalities | Genitourinary tumor | 2.39E-38 |
|  | Cancer, Gastrointestinal Disease, Organismal Injury and Abnormalities | Digestive organ tumor | 5.12E-38 |
|  | Cancer, Gastrointestinal Disease, Organismal Injury and Abnormalities | Digestive system cancer | 1.23E-36 |
|  | Cancer, Organismal Injury and Abnormalities | Neoplasia of cells | 1.04E-35 |
|  | Cancer, Organismal Injury and Abnormalities, Reproductive System Disease | Genital tract cancer | 2.24E-34 |
|  | Cancer, Organismal Injury and Abnormalities | Cancer of cells | 6.04E-34 |
|  | Cancer, Organismal Injury and Abnormalities | Pelvic cancer | 1.93E-33 |
|  | Cancer, Organismal Injury and Abnormalities, Reproductive System Disease | Genital tumor | 4.13E-33 |
|  | Cancer, Organismal Injury and Abnormalities, Reproductive System Disease | Female genital tract cancer | 8.06E-33 |
|  | Cancer, Organismal Injury and Abnormalities | Abdominal carcinoma | 1.22E-32 |
|  | Cancer, Organismal Injury and Abnormalities, Reproductive System Disease | Tumorigenesis of reproductive tract | 4.58E-32 |
|  | Cancer, Organismal Injury and Abnormalities, Reproductive System Disease | Female genital neoplasm | 7.55E-32 |
|  | Cancer, Organismal Injury and Abnormalities | Pelvic tumor | 1.95E-31 |
|  | Organismal Injury and Abnormalities | Benign lesion | 6.92E-31 |
|  | Cancer, Organismal Injury and Abnormalities | Advanced stage tumor | 8.25E-31 |
|  | Cellular Movement | Invasion of cells | 9.08E-31 |
|  | Cancer, Organismal Injury and Abnormalities | Advanced malignant tumor | 1.71E-30 |
|  | Cancer, Organismal Injury and Abnormalities | Benign Tumors | 2.61E-30 |
|  | Cancer, Organismal Injury and Abnormalities | Breast or pancreatic cancer | 4.51E-30 |
|  | Cancer, Organismal Injury and Abnormalities | Breast or colorectal cancer | 5.74E-30 |
|  | Cancer, Organismal Injury and Abnormalities | Secondary tumor | 6.33E-30 |
|  | Cancer, Organismal Injury and Abnormalities | Serous adenocarcinoma | 1.84E-29 |
|  | Cell Death and Survival | Cell death | 3.67E-29 |
|  | Cancer, Organismal Injury and Abnormalities, Reproductive System Disease | Female genital tract serous carcinoma | 3.83E-29 |
|  | Cancer, Organismal Injury and Abnormalities | Abdominal adenocarcinoma | 4.89E-29 |
|  | Cancer, Gastrointestinal Disease, Organismal Injury and Abnormalities | Gastrointestinal tumor | 6.78E-29 |
|  | Cancer, Gastrointestinal Disease, Organismal Injury and Abnormalities | Gastrointestinal tract cancer | 1.79E-28 |
|  | Cancer, Organismal Injury and Abnormalities, Reproductive System Disease | Breast or ovarian cancer | 1.29E-27 |
|  | Cancer, Organismal Injury and Abnormalities | Adenocarcinoma | 1.38E-27 |
|  | Cancer, Organismal Injury and Abnormalities, Reproductive System Disease | Female genital tract adenocarcinoma | 3.47E-27 |
|  | Organismal Injury and Abnormalities | Growth of lesion | 5.38E-27 |
|  | Cell Death and Survival | Apoptosis | 5.67E-27 |
|  | Cardiovascular Disease, Organismal Injury and Abnormalities | Vaso-occlusion | 5.91E-27 |
|  | Cardiovascular Disease, Organismal Injury and Abnormalities | Atherosclerosis | 7.7E-27 |
|  | Cellular Movement | Cell movement of tumor cell lines | 1.09E-26 |
|  | Cancer, Organismal Injury and Abnormalities | Incidence of tumor | 1.2E-26 |
|  | Cardiovascular System Development and Function, Organismal Development | Angiogenesis | 1.27E-26 |
|  | Cardiovascular System Development and Function | Development of vasculature | 1.3E-26 |
|  | Cancer, Organismal Injury and Abnormalities | Growth of tumor | 1.51E-26 |
|  | Cancer, Organismal Injury and Abnormalities, Reproductive System Disease | Uterine serous papillary cancer | 1.64E-26 |
|  | Cancer, Organismal Injury and Abnormalities | Frequency of tumor | 1.94E-26 |
|  | Cardiovascular System Development and Function, Organismal Development | Vasculogenesis | 2.23E-26 |
|  | Cancer, Organismal Injury and Abnormalities | Genitourinary adenocarcinoma | 4.46E-26 |
|  | Cancer, Organismal Injury and Abnormalities | Genitourinary carcinoma | 5.27E-26 |
|  | Cellular Movement | Invasion of tumor cell lines | 1.47E-25 |
|  | Cardiovascular Disease, Organismal Injury and Abnormalities | Occlusion of artery | 1.89E-25 |
|  | Cellular Movement | Migration of tumor cell lines | 6.05E-25 |
|  | Cellular Movement | Cell movement of blood cells | 2.26E-24 |
|  | Cellular Movement | Migration of blood cells | 2.51E-24 |
|  | Inflammatory Response, Organismal Injury and Abnormalities | Inflammation of organ | 2.65E-24 |
|  | Metabolic Disease | Glucose metabolism disorder | 2.93E-24 |
|  | Cancer, Gastrointestinal Disease, Organismal Injury and Abnormalities | Colorectal tumor | 3.18E-24 |
|  | Cell Death and Survival | Necrosis | 5.12E-24 |
|  | Cancer, Organismal Injury and Abnormalities, Reproductive System Disease | Endometrial cancer | 6.43E-24 |
|  | Cellular Movement, Immune Cell Trafficking | Leukocyte migration | 6.83E-24 |
|  | Cancer, Organismal Injury and Abnormalities, Reproductive System Disease | Endometrium tumor | 1.14E-23 |
|  | Cancer, Gastrointestinal Disease, Organismal Injury and Abnormalities | Intestinal tumor | 1.79E-23 |
|  | Cancer, Organismal Injury and Abnormalities, Reproductive System Disease | Mammary tumor | 1.97E-23 |
|  | Cancer, Gastrointestinal Disease, Organismal Injury and Abnormalities | Colorectal cancer | 3.81E-23 |
|  | Dermatological Diseases and Conditions, Organismal Injury and Abnormalities | Psoriasis | 4.13E-23 |
|  | Cancer, Organismal Injury and Abnormalities, Reproductive System Disease | Uterine cancer | 1.21E-22 |
|  | Cancer, Organismal Injury and Abnormalities | Advanced malignant solid tumor | 2.78E-22 |
|  | Cancer, Hematological Disease, Organismal Injury and Abnormalities | Lymphohematopoietic neoplasia | 3.18E-22 |
|  | Tissue Morphology | Quantity of cells | 7.94E-22 |
|  | Cancer, Organismal Injury and Abnormalities, Reproductive System Disease | Uterine tumor | 8.35E-22 |
|  | Cancer, Organismal Injury and Abnormalities | Tumorigenesis of epithelial neoplasm | 1.94E-21 |
|  | Cellular Development, Cellular Growth and Proliferation | Cell proliferation of tumor cell lines | 2.06E-21 |
|  | Inflammatory Disease | Chronic inflammatory disorder | 2.15E-21 |
|  | Cancer, Organismal Injury and Abnormalities, Reproductive System Disease | Breast cancer | 3.17E-21 |
|  | Endocrine System Disorders, Gastrointestinal Disease, Metabolic Disease, Organismal Injury and Abnormalities | Diabetes mellitus | 3.44E-21 |
|  | Cancer, Organismal Injury and Abnormalities | Metastatic solid tumor | 7.14E-21 |
|  | Organismal Injury and Abnormalities | Fibrosis | 7.84E-21 |
|  | Cellular Movement | Cell movement of myeloid cells | 8E-21 |
|  | Cancer, Hematological Disease, Organismal Injury and Abnormalities | Lymphohematopoietic cancer | 1.13E-20 |
|  | Cancer, Organismal Injury and Abnormalities | Head and neck tumor | 1.51E-20 |
|  | Cancer, Organismal Injury and Abnormalities | Squamous-cell carcinoma | 1.82E-20 |
|  | Organismal Survival | Survival of organism | 2.11E-20 |
|  | Cancer, Hematological Disease, Organismal Injury and Abnormalities | Hematopoietic neoplasm | 2.41E-20 |
|  | Cellular Movement, Hematological System Development and Function, Immune Cell Trafficking | Cell movement of leukocytes | 7.62E-20 |
|  | Cancer, Organismal Injury and Abnormalities | Connective tissue tumor | 1.07E-19 |
|  | Connective Tissue Disorders, Inflammatory Disease, Inflammatory Response, Organismal Injury and Abnormalities, Skeletal and Muscular Disorders | Inflammation of joint | 1.31E-19 |
|  | Connective Tissue Disorders, Inflammatory Disease, Organismal Injury and Abnormalities, Skeletal and Muscular Disorders | Rheumatic Disease | 1.47E-19 |
|  | Cell Death and Survival | Apoptosis of tumor cell lines | 1.58E-19 |
|  | Cancer, Organismal Injury and Abnormalities | Head and neck carcinoma | 1.89E-19 |
|  | Gastrointestinal Disease, Hepatic System Disease, Organismal Injury and Abnormalities | Liver lesion | 2.07E-19 |
|  | Cancer, Organismal Injury and Abnormalities | Development of malignant tumor | 2.43E-19 |
|  | Cancer, Organismal Injury and Abnormalities | Connective or soft tissue tumor | 4.98E-19 |
|  | Cancer, Endocrine System Disorders, Organismal Injury and Abnormalities, Reproductive System Disease | Ovarian cancer | 5.03E-19 |
|  | Cell Death and Survival | Cell death of tumor cell lines | 6.19E-19 |
|  | Cancer, Organismal Injury and Abnormalities | Lymphatic system tumor | 6.55E-19 |
|  | Cancer, Gastrointestinal Disease, Organismal Injury and Abnormalities | Upper gastrointestinal carcinoma | 8.04E-19 |
|  | Cancer, Hematological Disease, Organismal Injury and Abnormalities | Lymphoid cancer | 8.64E-19 |
|  | Cell-To-Cell Signaling and Interaction | Interaction of tumor cell lines | 1.06E-18 |
|  | Cancer, Hematological Disease, Organismal Injury and Abnormalities | Hematologic cancer | 1.7E-18 |
|  | Cancer, Gastrointestinal Disease, Organismal Injury and Abnormalities | Gastrointestinal carcinoma | 2.08E-18 |
|  | Cardiovascular Disease | Hypertension | 2.08E-18 |
|  | Cancer, Organismal Injury and Abnormalities | Cancer of secretory structure | 2.89E-18 |
|  | Cardiovascular System Development and Function | Morphology of cardiovascular system | 2.98E-18 |
|  | Cancer, Organismal Injury and Abnormalities | Lymphoreticular neoplasm | 3.02E-18 |
|  | Cardiovascular Disease | Disorder of blood pressure | 3.63E-18 |
|  | Cellular Movement, Hematological System Development and Function, Immune Cell Trafficking, Inflammatory Response | Cell movement of phagocytes | 4.51E-18 |
|  | Cancer, Endocrine System Disorders, Organismal Injury and Abnormalities, Reproductive System Disease | Gonadal tumor | 4.9E-18 |
|  | Cancer, Endocrine System Disorders, Organismal Injury and Abnormalities, Reproductive System Disease | Ovarian tumor | 5.57E-18 |
|  | Cancer, Organismal Injury and Abnormalities | Visceral metastasis | 7.24E-18 |
|  | Cell-To-Cell Signaling and Interaction | Activation of cells | 7.62E-18 |
|  | Cell-To-Cell Signaling and Interaction | Binding of tumor cell lines | 7.97E-18 |
|  | Cancer, Hematological Disease, Immunological Disease, Organismal Injury and Abnormalities | Neoplasia of leukocytes | 8E-18 |
|  | Cancer, Gastrointestinal Disease, Organismal Injury and Abnormalities | Colon tumor | 1.42E-17 |
|  | Cancer, Organismal Injury and Abnormalities | Head and neck cancer | 1.73E-17 |
|  | Tissue Development | Growth of epithelial tissue | 1.73E-17 |
|  | Hematological Disease, Immunological Disease | Lymphoproliferative disorder | 2.11E-17 |
|  | Inflammatory Response | Inflammation of absolute anatomical region | 2.85E-17 |
|  | Cancer, Organismal Injury and Abnormalities | Thoracic neoplasm | 3.04E-17 |
|  | Cancer, Gastrointestinal Disease, Organismal Injury and Abnormalities | Upper gastrointestinal tract tumor | 3.1E-17 |
|  | Cancer, Organismal Injury and Abnormalities | Thoracic cancer | 3.59E-17 |
|  | Cancer, Gastrointestinal Disease, Organismal Injury and Abnormalities | Upper gastrointestinal tract cancer | 4.18E-17 |
|  | Cellular Movement | Cellular infiltration | 5.19E-17 |
|  | Cell-To-Cell Signaling and Interaction | Attachment of cells | 6.4E-17 |
|  | Cancer, Organismal Injury and Abnormalities, Respiratory Disease | Lung cancer | 6.56E-17 |
|  | Cancer, Organismal Injury and Abnormalities, Respiratory Disease | Lung tumor | 7.84E-17 |
|  | Cancer, Hematological Disease, Organismal Injury and Abnormalities | Lymphocytic neoplasm | 8.58E-17 |
|  | Cancer, Organismal Injury and Abnormalities, Respiratory Disease | Respiratory system tumor | 8.94E-17 |
|  | Cellular Development, Cellular Growth and Proliferation, Organ Development, Skeletal and Muscular System Development and Function, Tissue Development | Proliferation of muscle cells | 9.83E-17 |
|  | Cellular Movement | Homing of cells | 1.03E-16 |
|  | Hematological System Development and Function, Inflammatory Response, Tissue Morphology | Quantity of phagocytes | 1.37E-16 |
|  | Cardiovascular System Development and Function, Cellular Movement | Cell movement of endothelial cells | 1.71E-16 |
|  | Cancer, Gastrointestinal Disease, Organismal Injury and Abnormalities | Colon cancer | 1.73E-16 |
|  | Organismal Development | Morphology of body cavity | 2.07E-16 |
|  | Connective Tissue Disorders, Organismal Injury and Abnormalities, Skeletal and Muscular Disorders | Non-traumatic arthropathy | 2.16E-16 |
|  | Inflammatory Response | Inflammatory response | 2.33E-16 |
|  | Cancer, Hematological Disease, Immunological Disease, Organismal Injury and Abnormalities | Lymphocytic cancer | 2.49E-16 |
|  | Cardiovascular System Development and Function, Cellular Movement | Migration of endothelial cells | 2.55E-16 |
|  | Cellular Movement | Migration of tumor cells | 2.74E-16 |
|  | Cancer, Organismal Injury and Abnormalities | Malignant neoplasm of aerodigestive tract | 2.92E-16 |
|  | Cancer, Gastrointestinal Disease, Hepatic System Disease, Organismal Injury and Abnormalities | Hepatobiliary system cancer | 3.15E-16 |
|  | Cellular Movement | Cell movement of tumor cells | 3.33E-16 |
|  | Organismal Injury and Abnormalities, Reproductive System Disease | Benign pelvic disease | 3.7E-16 |
|  | Cellular Movement | Migration of cancer cells | 4.04E-16 |
|  | Cellular Movement | Chemotaxis | 4.15E-16 |
|  | Organismal Injury and Abnormalities, Reproductive System Disease | Endometriosis | 4.35E-16 |
|  | Cancer, Organismal Injury and Abnormalities, Tumor Morphology | Progression of tumor | 4.41E-16 |
|  | Cancer, Organismal Injury and Abnormalities | Malignant connective or soft tissue neoplasm | 5.44E-16 |
|  | Hematological System Development and Function, Tissue Morphology | Quantity of myeloid cells | 6.68E-16 |
|  | Cancer, Gastrointestinal Disease, Hepatic System Disease, Organismal Injury and Abnormalities | Liver tumor | 7.21E-16 |
|  | Dermatological Diseases and Conditions, Organismal Injury and Abnormalities | Skin lesion | 9.32E-16 |
|  | Cancer, Gastrointestinal Disease, Hepatic System Disease, Organismal Injury and Abnormalities | Liver cancer | 1.03E-15 |
|  | Immunological Disease | Systemic autoimmune syndrome | 1.04E-15 |
|  | Cancer, Organismal Injury and Abnormalities | Development of carcinoma | 1.1E-15 |
|  | Cell Death and Survival | Cell survival | 1.14E-15 |
|  | Organismal Survival | Organismal death | 1.4E-15 |
|  | Endocrine System Disorders, Gastrointestinal Disease, Metabolic Disease, Organismal Injury and Abnormalities | Diabetic complication | 1.66E-15 |
|  | Cancer, Gastrointestinal Disease, Organismal Injury and Abnormalities | Metastatic malignant neoplasm of digestive system | 1.79E-15 |
|  | Cancer, Gastrointestinal Disease, Organismal Injury and Abnormalities | Oral cancer | 1.85E-15 |
|  | Cancer, Organismal Injury and Abnormalities | Sarcoma | 2.03E-15 |
|  | Cancer, Organismal Injury and Abnormalities | Upper aerodigestive tract carcinoma | 2.07E-15 |
|  | Cellular Movement, Hematological System Development and Function, Immune Cell Trafficking, Inflammatory Response | Migration of phagocytes | 2.18E-15 |
|  | Cellular Movement | Cell movement of cancer cells | 2.27E-15 |
|  | Cellular Assembly and Organization, Cellular Function and Maintenance | Organization of cytoskeleton | 2.3E-15 |
|  | Infectious Diseases | Bacterial Infections | 2.74E-15 |
|  | Connective Tissue Development and Function, Tissue Development | Growth of connective tissue | 4.86E-15 |
|  | Connective Tissue Disorders, Immunological Disease, Inflammatory Disease, Inflammatory Response, Organismal Injury and Abnormalities, Skeletal and Muscular Disorders | Rheumatoid arthritis | 5.29E-15 |
|  | Cancer, Organismal Injury and Abnormalities | Breast or ovarian carcinoma | 6.48E-15 |
|  | Cancer, Organismal Injury and Abnormalities | Head and neck squamous cell carcinoma | 7.69E-15 |
|  | Cancer, Organismal Injury and Abnormalities | Metastasis of cells | 8.66E-15 |
|  | Dermatological Diseases and Conditions, Organismal Injury and Abnormalities | Plaque psoriasis | 8.78E-15 |
|  | Cardiovascular Disease, Organismal Injury and Abnormalities | Congestive heart failure | 1.13E-14 |
|  | Cancer, Cellular Development, Cellular Growth and Proliferation, Organismal Injury and Abnormalities, Tumor Morphology | Proliferation of tumor cells | 1.37E-14 |
|  | Cancer, Organismal Injury and Abnormalities | Extraadrenal retroperitoneal tumor | 1.46E-14 |
|  | Hematological System Development and Function, Tissue Morphology | Quantity of leukocytes | 1.47E-14 |
|  | Cancer, Hematological Disease, Immunological Disease, Organismal Injury and Abnormalities | Lymphoma | 1.56E-14 |
|  | Cellular Movement | Cell movement of muscle cells | 1.56E-14 |
|  | Cancer, Gastrointestinal Disease, Hepatic System Disease, Organismal Injury and Abnormalities | Liver carcinoma | 1.69E-14 |
|  | Cancer, Gastrointestinal Disease, Organismal Injury and Abnormalities | Metastatic gastrointestinal tract cancer | 1.81E-14 |
|  | Cancer, Gastrointestinal Disease, Organismal Injury and Abnormalities | Oral cavity carcinoma | 1.81E-14 |
|  | Cellular Assembly and Organization, Tissue Development | Fibrogenesis | 1.86E-14 |
|  | Cellular Movement, Skeletal and Muscular System Development and Function | Cell movement of smooth muscle cells | 2.79E-14 |
|  | Cell Death and Survival | Cell viability | 3.12E-14 |
|  | Cancer, Gastrointestinal Disease, Organismal Injury and Abnormalities | Advanced gastrointestinal tumor | 3.17E-14 |
|  | Cancer, Organismal Injury and Abnormalities | Malignant neoplasm of retroperitoneum | 3.23E-14 |
|  | Inflammatory Response | Inflammation of body cavity | 3.85E-14 |
|  | Organismal Injury and Abnormalities | Edema | 3.92E-14 |
|  | Cellular Assembly and Organization, Tissue Development | Formation of filaments | 4.25E-14 |
|  | Cellular Movement, Hematological System Development and Function, Immune Cell Trafficking | Cellular infiltration by leukocytes | 4.37E-14 |
|  | Cancer, Gastrointestinal Disease, Organismal Injury and Abnormalities | Secondary neoplasm of digestive system | 4.6E-14 |
|  | Tissue Development | Development of epithelial tissue | 5.1E-14 |
|  | Cancer, Organismal Injury and Abnormalities | Growth of malignant tumor | 5.72E-14 |
|  | Cancer, Gastrointestinal Disease, Hepatic System Disease, Organismal Injury and Abnormalities | Hepatocellular carcinoma | 5.77E-14 |
|  | Cancer, Gastrointestinal Disease, Organismal Injury and Abnormalities | Metastatic intestinal tumor | 5.91E-14 |
|  | Cancer, Organismal Injury and Abnormalities | Development of adenocarcinoma | 7.14E-14 |
|  | Cellular Development, Cellular Growth and Proliferation, Organ Development, Skeletal and Muscular System Development and Function, Tissue Development | Proliferation of smooth muscle cells | 7.35E-14 |
|  | Cellular Movement, Hematological System Development and Function, Immune Cell Trafficking | Cell movement of mononuclear leukocytes | 8.62E-14 |
|  | Cellular Development, Cellular Function and Maintenance, Cellular Growth and Proliferation | Assembly of cells | 8.86E-14 |
|  | Cell-To-Cell Signaling and Interaction, Cellular Assembly and Organization | Cell-cell contact | 1.05E-13 |
|  | Cardiovascular Disease | Vascular lesion | 1.07E-13 |
|  | Organismal Injury and Abnormalities, Renal and Urological Disease | Cystic kidney disease | 1.22E-13 |
|  | Cancer, Hematological Disease, Organismal Injury and Abnormalities | Hematologic cancer of cells | 1.23E-13 |
|  | Cancer, Organismal Injury and Abnormalities | Development of benign tumor | 1.32E-13 |
|  | Cardiovascular System Development and Function, Tissue Development | Development of endothelial tissue | 1.33E-13 |
|  | Cellular Growth and Proliferation, Connective Tissue Development and Function, Tissue Development | Proliferation of connective tissue cells | 1.45E-13 |
|  | Cancer, Hematological Disease, Immunological Disease, Organismal Injury and Abnormalities | Non-Hodgkin lymphoma | 1.61E-13 |
|  | Cell-To-Cell Signaling and Interaction, Cellular Movement | Recruitment of cells | 1.92E-13 |
|  | Developmental Disorder, Hereditary Disorder, Organismal Injury and Abnormalities, Renal and Urological Disease | Polycystic Kidney Disease | 2.01E-13 |
|  | Cardiovascular System Development and Function | Morphology of vessel | 2.07E-13 |
|  | Cardiovascular System Development and Function, Cellular Development, Cellular Function and Maintenance, Cellular Growth and Proliferation, Organismal Development, Tissue Development | Endothelial cell development | 2.26E-13 |
|  | Cardiovascular System Development and Function, Organ Morphology, Organismal Development | Morphology of heart | 2.51E-13 |
|  | Organismal Injury and Abnormalities, Renal and Urological Disease | Nephrotoxicity | 2.87E-13 |
|  | Immunological Disease | Allergy | 3.35E-13 |
|  | Cancer, Gastrointestinal Disease, Organismal Injury and Abnormalities | Advanced colorectal tumor | 3.83E-13 |
|  | Cancer, Gastrointestinal Disease, Organismal Injury and Abnormalities | Metastatic colorectal cancer | 3.91E-13 |
|  | Developmental Disorder, Organismal Injury and Abnormalities | Congenital malformation of genitourinary system | 3.94E-13 |
|  | Cellular Movement, Hematological System Development and Function, Immune Cell Trafficking | Cell movement of antigen presenting cells | 4.28E-13 |
|  | Endocrine System Disorders, Gastrointestinal Disease, Metabolic Disease, Organismal Injury and Abnormalities, Renal and Urological Disease | Diabetic nephropathy | 4.35E-13 |
|  | Embryonic Development, Organismal Development | Development of body trunk | 4.37E-13 |
|  | Cancer, Organismal Injury and Abnormalities | Cancer of head | 4.55E-13 |
|  | Cellular Growth and Proliferation | Colony formation | 4.56E-13 |
|  | Organismal Injury and Abnormalities, Renal and Urological Disease | Renal impairment | 4.8E-13 |
|  | Cancer, Organismal Injury and Abnormalities, Reproductive System Disease | Prostate cancer | 5.34E-13 |
|  | Cardiovascular Disease, Organismal Injury and Abnormalities | Coronary disease | 5.39E-13 |
|  | Hematological System Development and Function, Tissue Morphology | Quantity of blood cells | 5.46E-13 |
|  | Cancer, Organismal Injury and Abnormalities, Reproductive System Disease | Malignant neoplasm of male genital organ | 5.84E-13 |
|  | Cardiovascular System Development and Function, Cell-To-Cell Signaling and Interaction | Interaction of endothelial cells | 6.18E-13 |
|  | Cellular Development, Cellular Growth and Proliferation | Cell proliferation of carcinoma cell lines | 6.45E-13 |
|  | Cellular Movement, Hematological System Development and Function, Immune Cell Trafficking, Inflammatory Response | Cell movement of macrophages | 6.62E-13 |
|  | Cardiovascular Disease, Organismal Injury and Abnormalities | Infarction | 6.65E-13 |
|  | Cellular Assembly and Organization, Cellular Function and Maintenance | Microtubule dynamics | 7.14E-13 |
|  | Cancer, Organismal Injury and Abnormalities | Upper aero-digestive squamous cell carcinoma | 7.19E-13 |
|  | Hematological System Development and Function, Tissue Development | Accumulation of myeloid cells | 7.34E-13 |
|  | Cancer, Gastrointestinal Disease, Organismal Injury and Abnormalities | Advanced malignant gastrointestinal neoplasm | 7.84E-13 |
|  | Cellular Movement, Skeletal and Muscular System Development and Function | Migration of smooth muscle cells | 7.88E-13 |
|  | Cell-To-Cell Signaling and Interaction | Adhesion of blood cells | 8.4E-13 |
|  | Developmental Disorder | Syndromic ciliopathy | 8.72E-13 |
|  | Cardiovascular System Development and Function, Tissue Morphology | Morphology of blood vessel | 8.98E-13 |
|  | Cancer, Gastrointestinal Disease, Organismal Injury and Abnormalities | Oral squamous cell carcinoma | 9.12E-13 |
|  | Developmental Disorder, Organismal Injury and Abnormalities, Renal and Urological Disease | Congenital anomalies of kidney and urinary tract | 9.32E-13 |
|  | Tissue Development | Accumulation of cells | 9.54E-13 |
|  | Cancer, Hematological Disease, Immunological Disease, Organismal Injury and Abnormalities | T-cell non-Hodgkin lymphoma | 9.98E-13 |
|  | Cancer, Hematological Disease, Immunological Disease, Organismal Injury and Abnormalities | Mature T-cell neoplasm | 1E-12 |
|  | Dermatological Diseases and Conditions, Inflammatory Disease, Inflammatory Response, Organismal Injury and Abnormalities | Dermatitis | 1.12E-12 |
|  | Cardiovascular System Development and Function, Cell-To-Cell Signaling and Interaction | Binding of endothelial cells | 1.16E-12 |
|  | Cancer, Organismal Injury and Abnormalities, Reproductive System Disease | Male genital neoplasm | 1.22E-12 |
|  | Cancer, Organismal Injury and Abnormalities, Reproductive System Disease | Prostatic tumor | 1.33E-12 |
|  | Cancer, Organismal Injury and Abnormalities | Angiogenesis of tumor | 1.4E-12 |
|  | Cell-To-Cell Signaling and Interaction, Hematological System Development and Function | Activation of myeloid cells | 1.66E-12 |
|  | Cell Morphology, Tissue Development | Tubulation of cells | 1.72E-12 |
|  | Cancer, Endocrine System Disorders, Organismal Injury and Abnormalities | Endocrine gland tumor | 1.94E-12 |
|  | Embryonic Development, Organismal Development | Development of head | 1.99E-12 |
|  | Gastrointestinal Disease, Organismal Injury and Abnormalities | Benign oral disorder | 2.11E-12 |
|  | Cellular Development, Connective Tissue Development and Function, Tissue Development | Differentiation of connective tissue cells | 2.15E-12 |
|  | Cancer, Organismal Injury and Abnormalities, Respiratory Disease | Non-small cell lung carcinoma | 2.2E-12 |
|  | Cancer, Dermatological Diseases and Conditions, Organismal Injury and Abnormalities | Skin tumor | 2.26E-12 |
|  | Cancer, Organismal Injury and Abnormalities, Reproductive System Disease | Breast carcinoma | 2.33E-12 |
|  | Cell-To-Cell Signaling and Interaction, Cellular Growth and Proliferation | Stimulation of cells | 2.44E-12 |
|  | Cellular Movement, Hematological System Development and Function, Immune Cell Trafficking | Migration of antigen presenting cells | 2.54E-12 |
|  | Cell-To-Cell Signaling and Interaction | Binding of blood cells | 2.65E-12 |
|  | Cellular Growth and Proliferation | Outgrowth of cells | 2.84E-12 |
|  | Connective Tissue Development and Function, Skeletal and Muscular System Development and Function, Tissue Development | Morphology of bone | 2.85E-12 |
|  | Hematological System Development and Function, Tissue Development | Accumulation of blood cells | 2.96E-12 |
|  | Respiratory Disease | Lower respiratory tract disorder | 3.09E-12 |
|  | Cancer, Endocrine System Disorders, Gastrointestinal Disease, Organismal Injury and Abnormalities | Pancreatic cancer | 3.25E-12 |
|  | Endocrine System Disorders, Gastrointestinal Disease, Organismal Injury and Abnormalities | Pancreatic mass | 3.26E-12 |
|  | Cardiovascular Disease, Organismal Injury and Abnormalities | Peripheral vascular disease | 3.32E-12 |
|  | Cellular Movement | Transmigration of cells | 3.35E-12 |
|  | Hematological System Development and Function, Immune Cell Trafficking, Inflammatory Response, Tissue Development | Accumulation of leukocytes | 3.5E-12 |
|  | Cardiovascular System Development and Function | Neovascularization | 3.56E-12 |
|  | Cancer, Organismal Injury and Abnormalities | Neoplasia of tumor cell lines | 4.04E-12 |
|  | Cell Death and Survival | Cell death of blood cells | 4.08E-12 |
|  | Cardiovascular System Development and Function, Organismal Development | Vascularization of absolute anatomical region | 4.32E-12 |
|  | Cell-To-Cell Signaling and Interaction | Adhesion of tumor cell lines | 4.61E-12 |
|  | Cancer, Cardiovascular Disease, Organismal Injury and Abnormalities | Vascular tumor | 4.99E-12 |
|  | Embryonic Development, Organismal Development | Development of body axis | 4.99E-12 |
|  | Cell-To-Cell Signaling and Interaction, Hematological System Development and Function, Immune Cell Trafficking, Inflammatory Response | Activation of phagocytes | 5.1E-12 |
|  | Cancer, Gastrointestinal Disease, Organismal Injury and Abnormalities | Pancreatobiliary tumor | 5.52E-12 |
|  | Cancer, Organismal Injury and Abnormalities, Respiratory Disease | Development of lung tumor | 6.01E-12 |
|  | Immunological Disease | Hypersensitive reaction | 6.44E-12 |
|  | Cardiovascular System Development and Function | Neovascularization of organ | 6.55E-12 |
|  | Organismal Injury and Abnormalities, Reproductive System Disease | Disorder of pregnancy | 6.6E-12 |
|  | Cancer, Organismal Injury and Abnormalities, Respiratory Disease | Lung carcinoma | 6.97E-12 |
|  | Cancer, Organismal Injury and Abnormalities, Tumor Morphology | Invasion of tumor | 7.25E-12 |
|  | Connective Tissue Disorders, Inflammatory Disease, Inflammatory Response, Organismal Injury and Abnormalities, Skeletal and Muscular Disorders | Osteoarthritis | 7.34E-12 |
|  | Cardiovascular System Development and Function | Vascularization | 7.62E-12 |
|  | Cell-To-Cell Signaling and Interaction, Cellular Movement | Recruitment of blood cells | 7.92E-12 |
|  | Cellular Growth and Proliferation | Colony formation of tumor cell lines | 7.93E-12 |
|  | Cancer, Endocrine System Disorders, Gastrointestinal Disease, Organismal Injury and Abnormalities | Pancreatic tumor | 8.04E-12 |
|  | Lipid Metabolism, Small Molecule Biochemistry | Synthesis of lipid | 8.11E-12 |
|  | Cancer, Organismal Injury and Abnormalities | Development of neuroepithelial tumor | 8.15E-12 |
|  | Cardiovascular Disease, Organismal Injury and Abnormalities | Coronary artery disease | 9.68E-12 |
|  | Cancer, Organismal Injury and Abnormalities | Invasion of tissue | 9.82E-12 |
|  | Hematological System Development and Function, Tissue Morphology | Quantity of antigen presenting cells | 1.02E-11 |
|  | Cell-To-Cell Signaling and Interaction, Cellular Movement, Hematological System Development and Function, Immune Cell Trafficking | Recruitment of leukocytes | 1.03E-11 |
|  | Cell-To-Cell Signaling and Interaction, Hematological System Development and Function, Immune Cell Trafficking | Adhesion of immune cells | 1.08E-11 |
|  | Organismal Injury and Abnormalities, Renal and Urological Disease | Proximal tubular toxicity | 1.08E-11 |
|  | Organismal Development | Growth of vessel | 1.16E-11 |
|  | Cancer, Organismal Injury and Abnormalities, Respiratory Disease | Lung adenocarcinoma | 1.35E-11 |
|  | Cell-To-Cell Signaling and Interaction, Hematological System Development and Function | Binding of leukocytes | 1.42E-11 |
|  | Cancer, Neurological Disease, Organismal Injury and Abnormalities | Glioma | 1.43E-11 |
|  | Cancer, Endocrine System Disorders, Organismal Injury and Abnormalities, Reproductive System Disease | Primary ovarian cancer | 1.48E-11 |
|  | Cancer, Organismal Injury and Abnormalities | Malignant squamous-cell carcinoma | 1.48E-11 |
|  | Cell Death and Survival | Cell death of immune cells | 1.58E-11 |
|  | Cellular Growth and Proliferation | Colony formation of cells | 1.62E-11 |
|  | Cellular Movement, Hematological System Development and Function, Immune Cell Trafficking, Inflammatory Response | Migration of macrophages | 1.63E-11 |
|  | Cancer, Organismal Injury and Abnormalities, Respiratory Disease | Advanced lung tumor | 1.7E-11 |
|  | Cardiovascular System Development and Function, Organismal Development, Visual System Development and Function | Vascularization of eye | 2.02E-11 |
|  | Cancer, Organismal Injury and Abnormalities | Adenoma | 2.39E-11 |
|  | Cellular Development, Cellular Growth and Proliferation, Nervous System Development and Function, Tissue Development | Outgrowth of neurons | 2.82E-11 |
|  | Hematological System Development and Function, Inflammatory Response, Tissue Morphology | Quantity of macrophages | 3.08E-11 |
|  | Cellular Assembly and Organization | Formation of cytoskeleton | 3.18E-11 |
|  | Cancer, Organismal Injury and Abnormalities | Tumor in nervous system | 3.25E-11 |
|  | Cellular Movement, Hematological System Development and Function, Immune Cell Trafficking | Migration of mononuclear leukocytes | 3.54E-11 |
|  | Nutritional Disease | Obesity | 3.69E-11 |
|  | Organismal Injury and Abnormalities | Hypertrophy | 3.79E-11 |
|  | Cellular Development, Cellular Growth and Proliferation, Nervous System Development and Function, Tissue Development | Proliferation of neuronal cells | 3.85E-11 |
|  | Cardiovascular Disease, Cardiovascular System Development and Function, Organ Morphology, Organismal Development, Organismal Injury and Abnormalities | Hypertrophy of heart | 3.86E-11 |
|  | Lipid Metabolism, Small Molecule Biochemistry | Fatty acid metabolism | 4.63E-11 |
|  | Cancer, Hematological Disease, Immunological Disease, Organismal Injury and Abnormalities | T-cell malignant neoplasm | 4.66E-11 |
|  | Cancer, Organismal Injury and Abnormalities, Tumor Morphology | Invasion of malignant tumor | 4.77E-11 |
|  | Cell-To-Cell Signaling and Interaction, Hematological System Development and Function | Activation of blood cells | 5.18E-11 |
|  | Embryonic Development, Organ Development, Organismal Development, Tissue Development | Development of sensory organ | 5.39E-11 |
|  | Organismal Injury and Abnormalities, Tissue Morphology | Size of lesion | 5.65E-11 |
|  | Cellular Movement, Hematological System Development and Function, Immune Cell Trafficking, Inflammatory Response | Migration of monocytes | 6.19E-11 |
|  | DNA Replication, Recombination, and Repair | Synthesis of DNA | 6.39E-11 |
|  | Cardiovascular Disease, Hematological Disease, Organismal Injury and Abnormalities | Thrombus | 6.41E-11 |
|  | Cardiovascular System Development and Function, Cellular Movement | Migration of vascular endothelial cells | 6.56E-11 |
|  | Cardiovascular System Development and Function, Cellular Movement | Movement of vascular endothelial cells | 7.21E-11 |
|  | Organismal Injury and Abnormalities | Blood clot | 7.73E-11 |
|  | Cancer, Neurological Disease, Organismal Injury and Abnormalities | Central nervous system tumor | 8.32E-11 |
|  | Cellular Movement, Hematological System Development and Function, Immune Cell Trafficking | Cell movement of granulocytes | 9.01E-11 |
|  | Cellular Movement, Hematological System Development and Function, Immune Cell Trafficking, Inflammatory Response | Cell movement of monocytes | 9.11E-11 |
|  | Cancer, Organismal Injury and Abnormalities | Primary neoplasm | 9.63E-11 |
|  | Cellular Movement | Cell movement of connective tissue cells | 1.01E-10 |
|  | Organismal Injury and Abnormalities | Visceromegaly | 1.02E-10 |
|  | Neurological Disease, Organismal Injury and Abnormalities, Psychological Disorders | Dementia | 1.16E-10 |
|  | Hematological System Development and Function, Immune Cell Trafficking, Inflammatory Response, Tissue Development | Accumulation of phagocytes | 1.29E-10 |
|  | Cell-To-Cell Signaling and Interaction, Hematological System Development and Function, Immune Cell Trafficking, Inflammatory Response | Activation of leukocytes | 1.3E-10 |
|  | Cellular Movement | Invasion of carcinoma cell lines | 1.36E-10 |
|  | Cardiovascular System Development and Function, Cellular Development, Cellular Function and Maintenance, Cellular Growth and Proliferation, Organismal Development, Tissue Development | Proliferation of endothelial cells | 1.37E-10 |
|  | Cellular Movement | Migration of myeloid cells | 1.52E-10 |
|  | Cellular Movement, Hematological System Development and Function, Immune Cell Trafficking, Inflammatory Response | Cellular infiltration by phagocytes | 1.57E-10 |
|  | Cardiovascular Disease, Organismal Injury and Abnormalities, Respiratory Disease | Pulmonary vascular disease | 1.62E-10 |
|  | Organismal Injury and Abnormalities, Renal and Urological Disease | Failure of kidney | 1.69E-10 |
|  | Cellular Development, Cellular Growth and Proliferation, Nervous System Development and Function, Tissue Development | Outgrowth of neurites | 1.79E-10 |
|  | Cardiovascular System Development and Function, Organismal Development, Visual System Development and Function | Neovascularization of eye | 1.9E-10 |
|  | Cellular Assembly and Organization | Development of cytoplasm | 2.02E-10 |
|  | Organ Morphology, Skeletal and Muscular System Development and Function, Tissue Morphology | Quantity of muscle cells | 2.04E-10 |
|  | Cancer, Dermatological Diseases and Conditions, Organismal Injury and Abnormalities | Papilloma | 2.33E-10 |
|  | Cancer, Cardiovascular Disease, Organismal Injury and Abnormalities | Hemangioma | 2.33E-10 |
|  | Cardiovascular Disease, Organismal Injury and Abnormalities | Abnormality of heart ventricle | 2.35E-10 |
|  | Cancer, Organismal Injury and Abnormalities | Papillary adenocarcinoma | 2.37E-10 |
|  | Cancer, Organismal Injury and Abnormalities | Metastasis of tumor cell lines | 2.39E-10 |
|  | Cellular Movement | Cellular infiltration by myeloid cells | 2.41E-10 |
|  | Cellular Movement | Cell movement of melanoma cell lines | 2.51E-10 |
|  | Cardiovascular Disease, Organismal Injury and Abnormalities | Atherosclerotic lesion | 2.54E-10 |
|  | Cell Death and Survival, Organismal Injury and Abnormalities | Necrosis of epithelial tissue | 2.61E-10 |
|  | Cellular Movement | Migration of melanoma cell lines | 2.75E-10 |
|  | Cellular Growth and Proliferation, Tissue Development | Proliferation of epithelial cells | 2.94E-10 |
|  | Cancer, Organismal Injury and Abnormalities, Skeletal and Muscular Disorders | Myosarcoma | 2.97E-10 |
|  | Organismal Injury and Abnormalities, Tissue Morphology | Morphology of lesion | 2.99E-10 |
|  | Organismal Injury and Abnormalities, Renal and Urological Disease | Renal lesion | 3.01E-10 |
|  | Cell-To-Cell Signaling and Interaction | Binding of connective tissue cells | 3.32E-10 |
|  | Skeletal and Muscular System Development and Function | Quantity of muscle | 3.49E-10 |
|  | Embryonic Development, Nervous System Development and Function, Organ Development, Organismal Development, Tissue Development, Visual System Development and Function | Formation of eye | 3.54E-10 |
|  | Molecular Transport | Quantity of metal | 3.79E-10 |
|  | Cell Cycle | Cell cycle progression | 3.99E-10 |
|  | Cellular Development, Connective Tissue Development and Function, Tissue Development | Differentiation of bone | 4.13E-10 |
|  | Embryonic Development, Hair and Skin Development and Function, Organ Development, Organismal Development, Tissue Development | Formation of skin | 4.28E-10 |
|  | Tissue Development | Tubulation of epithelial tissue | 4.66E-10 |
|  | Cardiovascular Disease, Organismal Injury and Abnormalities, Reproductive System Disease | Preeclampsia | 4.8E-10 |
|  | Cardiovascular Disease | Primary hypertension | 5.11E-10 |
|  | Cell Death and Survival | Cell death of leukemia cell lines | 5.35E-10 |
|  | Cardiovascular Disease, Organismal Injury and Abnormalities | Hypertension of blood vessel | 5.35E-10 |
|  | Hematological System Development and Function, Tissue Morphology | Quantity of granulocytes | 5.45E-10 |
|  | Dermatological Diseases and Conditions, Organismal Injury and Abnormalities | Chronic skin disorder | 5.54E-10 |
|  | Lipid Metabolism, Small Molecule Biochemistry | Synthesis of eicosanoid | 6.06E-10 |
|  | Cellular Movement | Cell movement of neuroglia | 6.18E-10 |
|  | Cancer, Dermatological Diseases and Conditions, Organismal Injury and Abnormalities | Skin cancer | 6.22E-10 |
|  | Cardiovascular System Development and Function, Tissue Morphology | Morphology of artery | 6.33E-10 |
|  | Lipid Metabolism, Small Molecule Biochemistry | Synthesis of fatty acid | 6.35E-10 |
|  | Cell Death and Survival | Cell viability of tumor cell lines | 6.35E-10 |
|  | Cancer, Neurological Disease, Organismal Injury and Abnormalities | Central nervous system solid tumor | 6.74E-10 |
|  | Organismal Injury and Abnormalities, Tissue Morphology | Volume of lesion | 7.34E-10 |
|  | Cellular Development, Connective Tissue Development and Function, Tissue Development | Differentiation of osteoblastic-lineage cells | 7.7E-10 |
|  | Connective Tissue Disorders, Organismal Injury and Abnormalities, Skeletal and Muscular Disorders | Abnormal bone density | 7.93E-10 |
|  | Cardiovascular Disease, Organismal Injury and Abnormalities | Failure of heart | 8.2E-10 |
|  | Cell-To-Cell Signaling and Interaction, Cellular Movement | Recruitment of myeloid cells | 8.2E-10 |
|  | Immunological Disease | Atopy | 9.02E-10 |
|  | Cancer, Organismal Injury and Abnormalities | Primary solid tumor | 9.14E-10 |
|  | Cellular Development, Connective Tissue Development and Function, Tissue Development | Differentiation of bone cells | 9.3E-10 |
|  | Cancer, Organismal Injury and Abnormalities, Reproductive System Disease | Invasive breast cancer | 9.36E-10 |
|  | Cellular Development | Differentiation of tumor cell lines | 9.37E-10 |
|  | Organismal Injury and Abnormalities, Renal and Urological Disease | Proteinuria | 9.46E-10 |
|  | Cellular Movement | Cell movement of carcinoma cell lines | 9.63E-10 |
|  | Cardiovascular Disease, Organismal Injury and Abnormalities, Respiratory Disease | Pulmonary Hypertension | 9.93E-10 |
|  | Cardiovascular System Development and Function, Cell Morphology, Cellular Development, Organismal Development, Tissue Development | Tubulation of endothelial cells | 9.93E-10 |
|  | Cardiovascular Disease, Organismal Injury and Abnormalities, Renal and Urological Disease | Cardiorenal syndrome | 1.04E-09 |
|  | Cellular Movement, Immune Cell Trafficking | Cell movement of lymphatic system cells | 1.08E-09 |
|  | Cancer, Endocrine System Disorders, Gastrointestinal Disease, Organismal Injury and Abnormalities | Pancreatic carcinoma | 1.18E-09 |
|  | Cancer, Organismal Injury and Abnormalities, Reproductive System Disease | Progesterone receptor negative breast tumor | 1.2E-09 |
|  | Cardiovascular Disease, Organismal Injury and Abnormalities | Infarction of heart | 1.25E-09 |
|  | Cancer, Hematological Disease, Immunological Disease, Organismal Injury and Abnormalities | Tumorigenesis of lymphocytes | 1.39E-09 |
|  | Nervous System Development and Function | Sensory system development | 1.48E-09 |
|  | Cancer, Endocrine System Disorders, Organismal Injury and Abnormalities | Thyroid carcinoma | 1.54E-09 |
|  | Cancer, Organismal Injury and Abnormalities, Reproductive System Disease | HER2 negative hormone receptor negative breast cancer | 1.6E-09 |
|  | Organismal Injury and Abnormalities, Respiratory Disease | Obstructive pulmonary disease | 1.6E-09 |
|  | Cancer, Endocrine System Disorders, Organismal Injury and Abnormalities | Neuroendocrine tumor | 1.64E-09 |
|  | Metabolic Disease, Neurological Disease, Organismal Injury and Abnormalities, Psychological Disorders | Alzheimer disease | 1.67E-09 |
|  | Cellular Development, Cellular Growth and Proliferation, Nervous System Development and Function, Tissue Development | Growth of neurites | 1.67E-09 |
|  | Cardiovascular System Development and Function, Hematological System Development and Function | Blood pressure | 1.72E-09 |
|  | Skeletal and Muscular System Development and Function | Quantity of smooth muscle | 1.73E-09 |
|  | Cancer, Gastrointestinal Disease, Organismal Injury and Abnormalities | Development of digestive organ tumor | 1.74E-09 |
|  | Metabolic Disease | Amyloidosis | 1.75E-09 |
|  | Cancer, Organismal Injury and Abnormalities, Skeletal and Muscular Disorders | Muscle tumor | 1.91E-09 |
|  | Cancer, Endocrine System Disorders, Organismal Injury and Abnormalities | Endocrine cancer | 2.01E-09 |
|  | Cancer, Gastrointestinal Disease, Organismal Injury and Abnormalities | Growth of digestive organ tumor | 2.03E-09 |
|  | Cancer, Endocrine System Disorders, Gastrointestinal Disease, Organismal Injury and Abnormalities | Pancreatic adenocarcinoma | 2.34E-09 |
|  | Immunological Disease | Immediate hypersensitivity | 2.36E-09 |
|  | Cellular Movement | Migration of breast cancer cell lines | 2.36E-09 |
|  | Cellular Movement | Invasion of pancreatic cancer cell lines | 2.38E-09 |
|  | Cell-To-Cell Signaling and Interaction, Cellular Movement, Hematological System Development and Function, Immune Cell Trafficking, Inflammatory Response | Recruitment of neutrophils | 2.39E-09 |
|  | Cellular Movement | Migration of connective tissue cells | 2.47E-09 |
|  | Cell Signaling, Molecular Transport, Vitamin and Mineral Metabolism | Quantity of Ca2+ | 2.48E-09 |
|  | Organismal Injury and Abnormalities | Bleeding | 2.56E-09 |
|  | Cell Death and Survival | Apoptosis of leukemia cell lines | 2.65E-09 |
|  | Cellular Movement, Skeletal and Muscular System Development and Function | Migration of vascular smooth muscle cells | 2.81E-09 |
|  | Cancer, Hematological Disease, Immunological Disease, Organismal Injury and Abnormalities | B-cell neoplasm | 2.93E-09 |
|  | Cellular Function and Maintenance | Cellular homeostasis | 3.19E-09 |
|  | Cancer, Organismal Injury and Abnormalities, Renal and Urological Disease | Renal tumor | 3.26E-09 |
|  | Cellular Movement, Hematological System Development and Function, Immune Cell Trafficking, Inflammatory Response | Cell movement of neutrophils | 3.38E-09 |
|  | Organismal Development | Development of genitourinary system | 3.4E-09 |
|  | Cellular Development, Cellular Growth and Proliferation | Proliferation of blood cells | 3.47E-09 |
|  | Cellular Movement, Immune Cell Trafficking | Cell movement of lymphoid cells | 3.47E-09 |
|  | Neurological Disease, Organismal Injury and Abnormalities, Psychological Disorders | Tauopathy | 3.75E-09 |
|  | Cellular Movement | Cell movement of central nervous system cells | 3.85E-09 |
|  | Cellular Movement, Skeletal and Muscular System Development and Function | Cell movement of vascular smooth muscle cells | 3.87E-09 |
|  | Cancer, Organismal Injury and Abnormalities, Reproductive System Disease | Development of genital tumor | 4.47E-09 |
|  | Cardiovascular System Development and Function, Cell-To-Cell Signaling and Interaction | Adhesion of endothelial cells | 4.67E-09 |
|  | Cancer, Organismal Injury and Abnormalities, Reproductive System Disease | HER2-negative breast cancer | 4.84E-09 |
|  | Cancer, Gastrointestinal Disease, Organismal Injury and Abnormalities | Gastro-esophageal carcinoma | 5.12E-09 |
|  | Cancer, Organismal Injury and Abnormalities | Primary tumor | 5.35E-09 |
|  | Organismal Injury and Abnormalities, Renal and Urological Disease | Urination disorder | 5.42E-09 |
|  | Cancer, Organismal Injury and Abnormalities, Renal and Urological Disease | Renal cancer | 5.74E-09 |
|  | Molecular Transport | Transport of molecule | 5.84E-09 |
|  | Cell-To-Cell Signaling and Interaction, Hematological System Development and Function, Immune Cell Trafficking, Inflammatory Response | Activation of antigen presenting cells | 5.89E-09 |
|  | Cell Death and Survival | Neuronal cell death | 5.97E-09 |
|  | Endocrine System Disorders, Metabolic Disease | Metabolic syndrome X | 6E-09 |
|  | Cancer, Organismal Injury and Abnormalities | Benign connective or soft tissue neoplasm | 6.84E-09 |
|  | Cardiovascular System Development and Function, Organ Development, Organ Morphology | Contraction of heart | 7E-09 |
|  | Cardiovascular Disease, Organismal Injury and Abnormalities, Skeletal and Muscular Disorders | Myocardial infarction | 7.2E-09 |
|  | Cancer, Cellular Development, Cellular Growth and Proliferation, Organismal Injury and Abnormalities, Tumor Morphology | Proliferation of cancer cells | 7.31E-09 |
|  | Cell-To-Cell Signaling and Interaction, Cellular Movement, Hematological System Development and Function, Immune Cell Trafficking | Recruitment of granulocytes | 7.39E-09 |
|  | Cellular Growth and Proliferation, Lymphoid Tissue Structure and Development | Proliferation of lymphatic system cells | 7.68E-09 |
|  | Cancer, Organismal Injury and Abnormalities | Growth of carcinoma | 7.68E-09 |
|  | Cellular Movement | Cell movement of prostate cancer cell lines | 7.69E-09 |
|  | Cellular Movement, Hematological System Development and Function, Immune Cell Trafficking | Cell movement of lymphocytes | 7.76E-09 |
|  | Cancer, Organismal Injury and Abnormalities, Renal and Urological Disease | Urinary tract cancer | 7.93E-09 |
|  | Cellular Development, Cellular Growth and Proliferation, Lymphoid Tissue Structure and Development, Tissue Development | Proliferation of bone marrow cells | 7.98E-09 |
|  | Cardiovascular System Development and Function, Organ Development | Heart rate | 8.68E-09 |
|  | Cell-To-Cell Signaling and Interaction, Cellular Movement, Hematological System Development and Function, Immune Cell Trafficking, Inflammatory Response | Recruitment of phagocytes | 8.74E-09 |
|  | Hematological Disease | Blood protein disorder | 9.15E-09 |
|  | Cell-To-Cell Signaling and Interaction | Adhesion of connective tissue cells | 9.42E-09 |
|  | Organismal Injury and Abnormalities | Fibrosis of tissue | 9.73E-09 |
|  | Cancer, Hematological Disease, Immunological Disease, Organismal Injury and Abnormalities | Angioimmunoblastic T-cell lymphoma | 9.79E-09 |
|  | Cellular Development, Connective Tissue Development and Function, Skeletal and Muscular System Development and Function, Tissue Development | Differentiation of osteoblasts | 9.91E-09 |
|  | Cellular Assembly and Organization, Cellular Function and Maintenance, Organ Morphology, Skeletal and Muscular System Development and Function, Tissue Morphology | Quantity of smooth muscle cells | 9.94E-09 |
|  | Cancer, Organismal Injury and Abnormalities | Invasive cancer | 1.05E-08 |
|  | Cancer, Organismal Injury and Abnormalities | Subcutaneous tumor | 1.06E-08 |
|  | Embryonic Development, Organ Development, Organismal Development, Respiratory System Development and Function, Tissue Development | Formation of lung | 1.12E-08 |
|  | Cancer, Endocrine System Disorders, Organismal Injury and Abnormalities | Thyroid gland adenocarcinoma | 1.18E-08 |
|  | Cancer, Endocrine System Disorders, Organismal Injury and Abnormalities, Reproductive System Disease | Ovarian adenocarcinoma | 1.23E-08 |
|  | Hair and Skin Development and Function, Organ Development | Growth of skin | 1.24E-08 |
|  | Cancer, Organismal Injury and Abnormalities | Chondrosarcoma | 1.35E-08 |
|  | Organismal Injury and Abnormalities, Respiratory Disease | Chronic obstructive pulmonary disease | 1.38E-08 |
|  | Hematological System Development and Function, Immune Cell Trafficking, Inflammatory Response, Tissue Development | Accumulation of antigen presenting cells | 1.38E-08 |
|  | Cell Death and Survival | Apoptosis of leukocytes | 1.43E-08 |
|  | Dermatological Diseases and Conditions, Organismal Injury and Abnormalities | Chronic psoriasis | 1.44E-08 |
|  | Cancer, Organismal Injury and Abnormalities, Respiratory Disease | Pulmonary metastasis | 1.49E-08 |
|  | Cellular Movement, Nervous System Development and Function | Migration of neuroglia | 1.5E-08 |
|  | Cardiovascular Disease, Organismal Injury and Abnormalities | Aortic dilatation | 1.5E-08 |
|  | Organismal Development, Renal and Urological System Development and Function | Development of urinary tract | 1.57E-08 |
|  | Cellular Movement | Cell movement of breast cancer cell lines | 1.69E-08 |
|  | Cancer, Organismal Injury and Abnormalities | Head and neck adenocarcinoma | 1.71E-08 |
|  | Gastrointestinal Disease, Immunological Disease, Ophthalmic Disease, Organismal Injury and Abnormalities | Sjögren syndrome | 1.76E-08 |
|  | Cancer, Endocrine System Disorders, Organismal Injury and Abnormalities, Reproductive System Disease | Malignant epithelial ovarian tumor | 1.77E-08 |
|  | Cardiovascular Disease, Organismal Injury and Abnormalities | Acute coronary event | 1.84E-08 |
|  | Cancer, Hematological Disease, Immunological Disease, Organismal Injury and Abnormalities | B cell cancer | 1.88E-08 |
|  | Cardiovascular System Development and Function, Cellular Movement | Cell movement of endothelial cell lines | 1.96E-08 |
|  | Cellular Movement | Cell movement of epithelial cells | 1.98E-08 |

**Supplementary table S10.** Differentially expressed proteins (*P_FDR_* < 0.05 and fold change ≥ 2) in the patients with HDLI compared to unaffected control, adjusted by age

| Protein | Accession number | log2(Patient/Control) | *P_FDR_* |
| --- | --- | --- | --- |
| LSP1 | P33241-3 | 1.50435 | 0.032 |
| GMPPA | Q96IJ6-2 | 1.38002 | 0.032 |
| ALDH1B1 | P30837 | 1.24173 | 0.032 |
| HLA-E | P13747 | -1.05277 | 0.032 |
| ARAP1 | Q96P48 | 1.42779 | 0.032 |
| ABI3BP | Q7Z7G0 | -1.28817 | 0.032 |
| MAP4 | P27816 | 2.14847 | 0.032 |
| OGFR | Q9NZT2 | 1.2265 | 0.032 |
| CRKL | P46109 | 1.48084 | 0.032 |
| VTN | P04004 | -1.25665 | 0.032 |
| GMPPB | Q9Y5P6-2 | 1.46584 | 0.032 |
| PPP2R2A | P63151-2 | 1.36842 | 0.032 |
| SAE1 | Q9UBE0 | 1.29224 | 0.032 |
| EIF2AK2 | P19525 | 1.65349 | 0.032 |
| FKBP5 | Q13451 | 1.65972 | 0.032 |
| YBX1 | P67809 | 1.3046 | 0.032 |
| IGKV4-1 | P06312 | 2.01561 | 0.032 |
| ITGAV | P06756 | 1.3727 | 0.032 |
| EPB42 | P16452-2 | -1.4004 | 0.033 |
| DNPH1 | O43598 | 1.25266 | 0.033 |
| OVCA2 | Q8WZ82 | 1.9717 | 0.033 |
| LAMA3 | Q16787 | -1.18938 | 0.033 |
| THEMIS2 | Q5TEJ8 | 1.27533 | 0.033 |
| CAPRIN1 | Q14444 | 2.05674 | 0.033 |
| POLDIP2 | Q9Y2S7 | 1.68648 | 0.033 |
| CFHR1 | Q03591 | -1.13105 | 0.033 |
| TMOD1 | P28289 | -1.60482 | 0.033 |
| DRG2 | P55039 | 1.1919 | 0.033 |
| DPYD | Q12882 | 2.33397 | 0.033 |
| GUSB | P08236 | 1.7385 | 0.033 |
| PODN | Q7Z5L7-3 | -1.92753 | 0.033 |
| PRPF4 | O43172 | 1.03999 | 0.033 |
| LAMA5 | O15230 | -1.0683 | 0.033 |
| CDH13 | P55290-4 | -1.6758 | 0.033 |
| MMP2 | P08253 | 1.08637 | 0.033 |
| ABHD11 | Q8NFV4 | 1.00862 | 0.033 |
| GYG1 | P46976 | 1.2597 | 0.033 |
| RAB34 | Q9BZG1 | 1.85728 | 0.033 |
| OAS3 | Q9Y6K5 | 1.33351 | 0.033 |
| ABCE1 | P61221 | 1.41278 | 0.033 |
| GGT5 | P36269-3 | 1.77026 | 0.033 |
| ALDH18A1 | P54886 | 1.14476 | 0.033 |
| CPSF3 | Q9UKF6 | 1.28704 | 0.033 |
| YKT6 | O15498 | 1.07867 | 0.033 |
| THY1 | P04216 | 2.34721 | 0.033 |
| GYS1 | P13807 | 1.09769 | 0.033 |
| SRRM2 | Q9UQ35 | 1.26889 | 0.033 |
| RETN | Q9HD89 | 2.11359 | 0.033 |
| RIC8A | Q9NPQ8-3 | 1.75061 | 0.033 |
| UTRN | P46939-2 | -1.41133 | 0.033 |
| CAV2 | P51636 | -2.83182 | 0.033 |
| PDLIM4 | P50479 | 2.36498 | 0.033 |
| COPZ1 | P61923-4 | 1.19246 | 0.033 |
| SRSF3 | P84103 | 2.50704 | 0.033 |
| UPF1 | Q92900 | 1.25017 | 0.033 |
| ATP6V1D | Q9Y5K8 | 1.96211 | 0.033 |
| VCP | P55072 | 1.10988 | 0.033 |
| TIMP3 | P35625 | -1.95407 | 0.033 |
| LYPLA2 | O95372 | 2.44819 | 0.033 |
| SDF2L1 | Q9HCN8 | 1.89295 | 0.033 |
| PLAA | Q9Y263 | 1.32268 | 0.033 |
| DAZAP1 | Q96EP5 | 1.85524 | 0.033 |
| ADPRHL2 | Q9NX46 | 1.02453 | 0.034 |
| S100A9 | P06702 | 1.69962 | 0.034 |
| NCK1 | P16333 | 1.79376 | 0.034 |
| MRC2 | Q9UBG0 | 1.02421 | 0.034 |
| ACAT2 | Q9BWD1-2 | 2.57541 | 0.034 |
| SRP19 | P09132 | 1.40062 | 0.034 |
| CTSE | P14091 | 2.15255 | 0.034 |
| CRK | P46108 | 1.04034 | 0.034 |
| C12orf10 | Q9HB07 | 1.10733 | 0.034 |
| ARMT1 | Q9H993 | 1.89389 | 0.034 |
| DDX58 | O95786 | 1.55655 | 0.034 |
| PTBP1 | P26599-3 | 1.64612 | 0.034 |
| NIF3L1 | Q9GZT8 | 1.75547 | 0.034 |
| CSE1L | P55060 | 1.01051 | 0.034 |
| CFH | P08603 | -1.4329 | 0.034 |
| ELANE | P08246 | 1.74602 | 0.034 |
| IMMT | Q16891 | -1.23802 | 0.034 |
| SORBS1 | Q9BX66 | -1.26123 | 0.034 |
| NT5DC2 | Q9H857-2 | 1.95902 | 0.034 |
| PTPA | Q15257 | 1.59616 | 0.034 |
| RPL31 | P62899-2 | 1.46827 | 0.034 |
| FIP1L1 | Q6UN15 | 1.67915 | 0.034 |
| RNASE2 | P10153 | 1.34959 | 0.034 |
| MAVS | Q7Z434 | 1.4535 | 0.034 |
| S100A16 | Q96FQ6 | 2.04078 | 0.034 |
| TWF1 | Q12792 | 1.60085 | 0.034 |
| HSPB8 | Q9UJY1 | 1.55264 | 0.034 |
| HTATSF1 | O43719 | 1.95225 | 0.034 |
| RPS25 | P62851 | 1.33483 | 0.034 |
| FBLN1 | P23142-4 | 1.56046 | 0.034 |
| ARCN1 | P48444 | 1.09741 | 0.034 |
| ABCF1 | Q8NE71 | 1.20824 | 0.034 |
| CAD | P27708 | 1.5484 | 0.034 |
| MAGED2 | Q9UNF1 | 1.48175 | 0.034 |
| DCTD | P32321-2 | 2.06646 | 0.034 |
| SNX2 | O60749 | 1.05318 | 0.034 |
| PPCS | Q9HAB8 | 1.27273 | 0.034 |
| PFDN1 | O60925 | 2.02353 | 0.034 |
| HP | P00738 | -2.28703 | 0.034 |
| SPRYD4 | Q8WW59 | 2.3414 | 0.034 |
| PSMD5 | Q16401 | 1.25114 | 0.034 |
| COL15A1 | P39059 | 1.08331 | 0.035 |
| SUPT4H1 | P63272 | 1.95177 | 0.035 |
| AOC3 | Q16853 | -1.02622 | 0.035 |
| NAMPT | P43490 | 1.05022 | 0.035 |
| TRMT112 | Q9UI30 | 1.40932 | 0.035 |
| TTLL12 | Q14166 | 1.05862 | 0.035 |
| PEPD | P12955 | 1.65087 | 0.035 |
| MAP1LC3B2 | A6NCE7 | 1.66981 | 0.035 |
| MOXD1 | Q6UVY6 | 1.57358 | 0.035 |
| RANBP1 | P43487 | 1.3783 | 0.035 |
| EIF3B | P55884-2 | 1.14881 | 0.035 |
| AKAP12 | Q02952 | 1.21461 | 0.035 |
| CTSL | P07711 | 1.8056 | 0.035 |
| DDB1 | Q16531 | 1.11072 | 0.035 |
| NCBP2 | P52298 | 1.4867 | 0.035 |
| EML4 | Q9HC35 | 1.38739 | 0.035 |
| ATRIP | Q8WXE1 | 1.2136 | 0.036 |
| ATP6V1H | Q9UI12 | 1.31377 | 0.036 |
| ENPP4 | Q9Y6X5 | 1.07239 | 0.036 |
| OAT | P04181 | 1.6049 | 0.036 |
| PTGFRN | Q9P2B2 | 1.99812 | 0.036 |
| RHEB | Q15382 | 1.15687 | 0.036 |
| KRT5 | P13647 | 1.53404 | 0.036 |
| METAP2 | P50579 | 1.21398 | 0.036 |
| DAB2 | P98082 | 1.90823 | 0.036 |
| GSTO1 | P78417 | 1.30272 | 0.036 |
| MCU | Q8NE86 | 1.45376 | 0.036 |
| FKBP9 | O95302 | 1.29275 | 0.036 |
| GBP1 | P32455 | 1.22933 | 0.036 |
| CACNA2D2 | Q9NY47-5 | -1.41074 | 0.036 |
| UAP1L1 | Q3KQV9 | 2.17735 | 0.036 |
| HDGF | P51858 | 1.00489 | 0.036 |
| ORM1 | P02763 | 1.01419 | 0.036 |
| PPM1F | P49593 | 1.14723 | 0.036 |
| DCPS | Q96C86 | 1.42987 | 0.036 |
| AP1G2 | O75843 | 1.56143 | 0.036 |
| SH3D19 | Q5HYK7 | 1.18869 | 0.036 |
| NAE1 | Q13564-4 | 1.47323 | 0.037 |
| NTPCR | Q9BSD7 | 1.54775 | 0.037 |
| HIST1H1E | P10412 | -1.01234 | 0.037 |
| IL4I1 | Q96RQ9-2 | 1.97294 | 0.037 |
| GAA | P10253 | 1.65604 | 0.037 |
| SMC3 | Q9UQE7 | -1.48548 | 0.037 |
| FUBP3 | Q96I24 | 1.63206 | 0.037 |
| IGBP1 | P78318 | 1.30021 | 0.038 |
| LGALS7 | P47929 | 2.38183 | 0.038 |
| FLOT1 | O75955 | -1.16093 | 0.038 |
| PPIC | P45877 | 1.41976 | 0.038 |
| PIR | O00625 | 1.93227 | 0.038 |
| AMDHD2 | Q9Y303-3 | 1.79611 | 0.038 |
| SETD3 | Q86TU7 | 1.80667 | 0.038 |
| LAMB3 | Q13751 | -1.38865 | 0.038 |
| SAFB | Q15424-3 | 1.1323 | 0.038 |
| NMT1 | P30419 | 1.78437 | 0.039 |
| CD84 | Q9UIB8 | 1.20946 | 0.039 |
| PMPCB | O75439 | 1.8282 | 0.039 |
| TRADD | Q15628 | 1.32129 | 0.039 |
| TARDBP | Q13148 | 1.51496 | 0.039 |
| CA3 | P07451 | 1.40842 | 0.039 |
| SIAE | Q9HAT2 | 1.73205 | 0.039 |
| UQCRH | P07919 | 1.96284 | 0.039 |
| HDHD2 | Q9H0R4 | 1.67748 | 0.039 |
| HEATR5A | Q86XA9 | 1.74861 | 0.039 |
| S100A11 | P31949 | 1.35948 | 0.039 |
| CALU | O43852-3 | 1.77373 | 0.039 |
| LSM4 | Q9Y4Z0 | 1.88234 | 0.039 |
| STX11 | O75558 | -1.26505 | 0.039 |
| PNPO | Q9NVS9 | 1.04017 | 0.040 |
| PC | P11498 | 1.08399 | 0.040 |
| UBXN1 | Q04323-2 | 1.86872 | 0.040 |
| PKM | P14618-2 | 1.30726 | 0.040 |
| RFC4 | P35249 | 1.60872 | 0.040 |
| NDUFV3 | P56181-2 | 1.47174 | 0.041 |
| MCM2 | P49736 | 3.1072 | 0.041 |
| EHD3 | Q9NZN3 | -1.06318 | 0.041 |
| SAR1A | Q9NR31 | 1.07419 | 0.041 |
| NUP35 | Q8NFH5 | 1.14628 | 0.041 |
| BCKDK | O14874 | 1.28417 | 0.041 |
| RPS20 | P60866-2 | 1.27289 | 0.041 |
| KRT19 | P08727 | 1.10864 | 0.041 |
| GBP2 | P32456 | 1.00778 | 0.041 |
| EVPL | Q92817 | -1.06019 | 0.041 |
| F12 | P00748 | 1.72438 | 0.041 |
| NPC2 | P61916 | 1.20148 | 0.041 |
| ITGA3 | P26006-1 | -1.3723 | 0.041 |
| COL12A1 | Q99715 | -1.26924 | 0.041 |
| APOB | P04114 | -1.43381 | 0.041 |
| EIF2B1 | Q14232 | 2.07617 | 0.041 |
| VCAN | P13611-2 | 1.0527 | 0.041 |
| LARP1 | Q6PKG0 | 1.27371 | 0.041 |
| CNRIP1 | Q96F85 | 1.03182 | 0.041 |
| SLC44A2 | Q8IWA5-2 | -1.31973 | 0.042 |
| MECP2 | P51608-2 | -1.26956 | 0.042 |
| DIABLO | Q9NR28 | -1.06739 | 0.042 |
| AP1S1 | P61966 | 1.08656 | 0.042 |
| DIS3 | Q9Y2L1 | 3.53699 | 0.042 |
| IGFBP7 | Q16270 | 1.13596 | 0.042 |
| SRPRA | P08240 | 1.63124 | 0.042 |
| ITGA2B | P08514 | -2.45588 | 0.042 |
| CCDC124 | Q96CT7 | 2.08074 | 0.042 |
| ITGB5 | P18084 | 1.04275 | 0.042 |
| CREG1 | O75629 | 1.81817 | 0.042 |
| ARL1 | P40616 | 1.77656 | 0.042 |
| PDCD10 | Q9BUL8 | 1.36858 | 0.042 |
| PSMB2 | P49721 | 1.52775 | 0.043 |
| TIAL1 | Q01085-2 | 1.37442 | 0.043 |
| AASS | Q9UDR5 | -2.5776 | 0.043 |
| SF1 | Q15637 | 1.70382 | 0.043 |
| RAB18 | Q9NP72 | 1.539 | 0.043 |
| CPSF6 | Q16630-2 | 1.26875 | 0.044 |
| GNAI3 | P08754 | 1.10992 | 0.044 |
| ADRM1 | Q16186 | 1.33235 | 0.044 |
| PAFAH1B2 | P68402 | 2.50829 | 0.045 |
| EWSR1 | Q01844-5 | 1.583 | 0.045 |
| RPS2 | P15880 | 1.32077 | 0.045 |
| TP53I3 | Q53FA7 | 1.47095 | 0.045 |
| ATP6V1G1 | O75348 | 1.10925 | 0.046 |
| KPNB1 | Q14974 | 1.06131 | 0.046 |
| TRNT1 | Q96Q11 | 1.27418 | 0.046 |
| KPNA3 | O00505 | 1.40123 | 0.046 |
| SELENOF | O60613 | 1.03429 | 0.046 |
| SPTLC1 | O15269 | 1.15929 | 0.046 |
| ABHD12 | Q8N2K0-2 | 1.69616 | 0.046 |
| BPIFB1 | Q8TDL5 | 2.77833 | 0.046 |
| RBP1 | P09455 | 1.30233 | 0.046 |
| DDX42 | Q86XP3 | 1.71097 | 0.047 |
| AKR1C3 | P42330 | 1.59264 | 0.047 |
| UBE2I | P63279 | 1.20213 | 0.047 |
| SGSH | P51688 | 1.08088 | 0.047 |
| G3BP2 | Q9UN86 | 1.81408 | 0.047 |
| MURC | Q5BKX8 | -1.0478 | 0.047 |
| PRPF40A | O75400 | 1.04734 | 0.047 |
| SLC30A7 | Q8NEW0 | 2.38103 | 0.047 |
| DOCK9 | Q9BZ29-5 | -1.49817 | 0.047 |
| CYGB | Q8WWM9 | 1.74463 | 0.047 |
| CD38 | P28907 | 1.54552 | 0.047 |
| HNRNPAB | Q99729-2 | 1.61995 | 0.047 |
| ATXN2L | Q8WWM7-3 | 2.36328 | 0.048 |
| DFFA | O00273 | 1.3283 | 0.048 |
| GMPR2 | Q9P2T1-2 | 1.57833 | 0.048 |
| SIRT1 | Q96EB6 | 1.24417 | 0.048 |
| MRPS16 | Q9Y3D3 | 1.42193 | 0.048 |
| ARL2 | P36404 | 1.37551 | 0.048 |
| EMC3 | Q9P0I2 | 1.23275 | 0.048 |
| XPNPEP1 | Q9NQW7-3 | 1.52555 | 0.048 |
| CDC42 | P60953 | 2.10391 | 0.049 |
| PXDN | Q92626 | 1.31448 | 0.049 |
| CARHSP1 | Q9Y2V2 | 1.28566 | 0.049 |
| CTBS | Q01459 | 1.0656 | 0.049 |
| IMPDH2 | P12268 | 1.54494 | 0.049 |
| NT5C2 | P49902 | 2.33661 | 0.049 |
| LIPA | P38571 | 1.28119 | 0.049 |
| SNX17 | Q15036 | 1.36423 | 0.049 |
| TAP2 | Q03519 | 1.17181 | 0.049 |
| PRELP | P51888 | -1.34734 | 0.049 |
| ENOPH1 | Q9UHY7 | 1.00533 | 0.049 |
| RPL14 | P50914 | 1.24797 | 0.049 |

**Supplementary table S12.** Up-/down-regulated genes (fold change ≥ 2, *P* < 0.05) in the HD-exposed children FFPE lung tissue (Case, n = 5 vs. Control, n = 5)

| No | Up-/Down-regulation | Gene | Fold change (Case/Control) | *P*-value |
| --- | --- | --- | --- | --- |
| 1 | Up | *OR6C4* | 59.23 | 7.11E-07 |
| 2 | Up | *MIR637* | 12.95 | 7.18E-06 |
| 3 | Down | *LOC101927414* | -2.96 | 3.32E-05 |
| 4 | Up | *OR2L1P* | 5.54 | 4.30E-05 |
| 5 | Down | *TRBV20OR9-2* | -4.53 | 4.61E-05 |
| 6 | Up | *HIST1H2AE* | 2.49 | 8.00E-05 |
| 7 | Up | *VTRNA1-3* | 11.60 | 0.0002 |
| 8 | Up | *HIST1H2BK* | 15.83 | 0.0002 |
| 9 | Down | *MIR3689D1* | -2.03 | 0.0002 |
| 10 | Up | *MDM2* | 2.01 | 0.0002 |
| 11 | Up | *LOC105369360* | 4.10 | 0.0002 |
| 12 | Down | *HBG2* | -2.69 | 0.0003 |
| 13 | Down | *TRDJ2* | -3.01 | 0.0003 |
| 14 | Up | *MFSD12* | 2.46 | 0.0004 |
| 15 | Up | *SCARNA21* | 2.85 | 0.0004 |
| 16 | Up | *IGHV3-33* | 2.35 | 0.0004 |
| 17 | Up | *MIR130B* | 6.83 | 0.0004 |
| 18 | Up | *HIST1H2BI* | 4.18 | 0.0004 |
| 19 | Down | *SNORA80E* | -4.29 | 0.0005 |
| 20 | Down | *SNORD17* | -2.48 | 0.0005 |
| 21 | Up | *SNORA10* | 4.22 | 0.0006 |
| 22 | Up | *PLEKHM1P* | 2.49 | 0.0006 |
| 23 | Down | *LOC101927329* | -2.29 | 0.0007 |
| 24 | Down | *SNORD8* | -2.41 | 0.0007 |
| 25 | Down | *RFX3-AS1* | -2.01 | 0.0007 |
| 26 | Up | *MIR1910* | 3.19 | 0.0008 |
| 27 | Up | *CD248* | 2.03 | 0.0009 |
| 28 | Down | *MIR4478* | -2.78 | 0.0009 |
| 29 | Down | *LOC105376863* | -2.73 | 0.0009 |
| 30 | Up | *PPIAL4E* | 2.25 | 0.001 |
| 31 | Down | *FAM74A3* | -3.59 | 0.001 |
| 32 | Up | *IGLV2-11* | 1.95 | 0.001 |
| 33 | Up | *TMSB4XP4* | 4.07 | 0.001 |
| 34 | Up | *MMAB* | 8.91 | 0.001 |
| 35 | Up | *MIRLET7BHG* | 4.19 | 0.001 |
| 36 | Up | *HIST1H2BM* | 2.17 | 0.001 |
| 37 | Up | *WASH2P* | 2.05 | 0.002 |
| 38 | Down | *SNORA36B* | -2.34 | 0.002 |
| 39 | Up | *BGN* | 3.32 | 0.002 |
| 40 | Down | *PMS2P4* | -1.75 | 0.002 |
| 41 | Up | *NBPF12* | 3.21 | 0.002 |
| 42 | Up | *MRPL41* | 2.50 | 0.002 |
| 43 | Up | *ARL17A* | 2.16 | 0.002 |
| 44 | Up | *RPL10A* | 2.15 | 0.002 |
| 45 | Up | *IGHV1-18* | 13.59 | 0.002 |
| 46 | Down | *MIR3617* | -2.16 | 0.002 |
| 47 | Up | *H1F0* | 1.77 | 0.002 |
| 48 | Down | *SPDYE18* | -2.03 | 0.003 |
| 49 | Up | *LOXL1* | 1.49 | 0.003 |
| 50 | Up | *METRN* | 1.87 | 0.003 |
| 51 | Up | *SNRPN* | 3.14 | 0.003 |
| 52 | Down | *TRAJ59* | -2.11 | 0.003 |
| 53 | Up | *IGLV2-18* | 6.90 | 0.003 |
| 54 | Up | *CTSK* | 1.39 | 0.003 |
| 55 | Down | *SNORA68* | -2.60 | 0.003 |
| 56 | Up | *SULT1A2* | 2.11 | 0.003 |
| 57 | Up | *COL6A3* | 2.70 | 0.003 |
| 58 | Up | *IGF1* | 1.64 | 0.004 |
| 59 | Down | *RBMY2EP* | -2.06 | 0.004 |
| 60 | Up | *LUC7L3* | 2.40 | 0.004 |
| 61 | Down | *LOC105372587* | -2.29 | 0.004 |
| 62 | Up | *TEX35* | 2.11 | 0.004 |
| 63 | Down | *IGHV4-39* | -2.73 | 0.004 |
| 64 | Up | *SAT1* | 2.77 | 0.004 |
| 65 | Up | *IGKV1-5* | 2.03 | 0.004 |
| 66 | Down | *LINC00508* | -1.63 | 0.005 |
| 67 | Up | *USP18* | 2.28 | 0.005 |
| 68 | Down | *MAGEA1* | -2.11 | 0.005 |
| 69 | Down | *MIR4518* | -2.05 | 0.005 |
| 70 | Down | *MIR3689D2* | -2.02 | 0.005 |
| 71 | Up | *COL3A1* | 2.26 | 0.005 |
| 72 | Down | *XAGE-4* | -2.23 | 0.005 |
| 73 | Down | *MIR1321* | -2.91 | 0.005 |
| 74 | Down | *LOC105377042* | -3.18 | 0.006 |
| 75 | Down | *MIR548AD* | -2.07 | 0.006 |
| 76 | Up | *ZFP36L1* | 1.90 | 0.006 |
| 77 | Up | *MMP2* | 2.30 | 0.006 |
| 78 | Up | *IGHG4* | 2.27 | 0.006 |
| 79 | Up | *MIR4454* | 3.46 | 0.006 |
| 80 | Up | *CST3* | 2.59 | 0.006 |
| 81 | Down | *TRAJ7* | -2.52 | 0.006 |
| 82 | Down | *CCDC30* | -2.10 | 0.007 |
| 83 | Down | *MIR3918* | -2.06 | 0.007 |
| 84 | Up | *LOC100288069* | 2.37 | 0.007 |
| 85 | Up | *MIR4263* | 4.92 | 0.007 |
| 86 | Up | *VTRNA1-1* | 2.31 | 0.007 |
| 87 | Down | *KRT17* | -2.17 | 0.007 |
| 88 | Up | *NPIPA7* | 3.69 | 0.007 |
| 89 | Down | *MIR4486* | -2.01 | 0.007 |
| 90 | Up | *NPIPB11* | 4.17 | 0.008 |
| 91 | Up | *SRSF6* | 2.07 | 0.008 |
| 92 | Down | *MIR4266* | -2.09 | 0.008 |
| 93 | Down | *SNORD123* | -2.37 | 0.008 |
| 94 | Down | *TRAJ46* | -2.47 | 0.008 |
| 95 | Up | *IGLV3-25* | 2.34 | 0.008 |
| 96 | Up | *NPIPB5* | 2.96 | 0.008 |
| 97 | Up | *MIR623* | 6.65 | 0.008 |
| 98 | Up | *COL1A2* | 1.90 | 0.008 |
| 99 | Up | *ZNF417* | 2.49 | 0.008 |
| 100 | Up | *IGHV3-48* | 2.48 | 0.008 |
| 101 | Up | *KRT18P54* | 2.12 | 0.008 |
| 102 | Up | *NPIP* | 2.41 | 0.008 |
| 103 | Up | *GALNT1* | 1.97 | 0.009 |
| 104 | Down | *ZNF474* | -2.10 | 0.009 |
| 105 | Up | *NPIPA5* | 3.03 | 0.009 |
| 106 | Down | *IGKV1D-27* | -2.81 | 0.009 |
| 107 | Up | *SNORD14E* | 2.60 | 0.009 |
| 108 | Down | *LOC105370111* | -2.03 | 0.009 |
| 109 | Up | *SNORD35B* | 2.21 | 0.010 |
| 110 | Up | *HILPDA* | 1.59 | 0.010 |
| 111 | Up | *NPIPB3* | 3.16 | 0.010 |
| 112 | Down | *MIR520C* | -2.66 | 0.010 |
| 113 | Up | *SNORD41* | 6.03 | 0.010 |
| 114 | Down | *MIR520E* | -2.03 | 0.011 |
| 115 | Up | *EEF2* | 2.51 | 0.011 |
| 116 | Down | *ARGFX* | -2.01 | 0.011 |
| 117 | Up | *THBS2* | 1.51 | 0.011 |
| 118 | Up | *CCL13* | 2.10 | 0.011 |
| 119 | Up | *GLUL* | 1.58 | 0.011 |
| 120 | Up | *DUSP23* | 2.42 | 0.011 |
| 121 | Up | *NPIPB4* | 3.67 | 0.011 |
| 122 | Down | *MIR3914-1* | -3.66 | 0.011 |
| 123 | Up | *LOC105379690* | 2.00 | 0.011 |
| 124 | Up | *ARPC2* | 2.27 | 0.011 |
| 125 | Down | *MIRLET7A2* | -2.10 | 0.012 |
| 126 | Up | *MALAT1* | 3.79 | 0.012 |
| 127 | Up | *RASA4B* | 2.04 | 0.012 |
| 128 | Up | *ANKRD36B* | 2.09 | 0.012 |
| 129 | Up | *IGKV2D-28* | 1.60 | 0.012 |
| 130 | Up | *ND6* | 6.18 | 0.013 |
| 131 | Up | *LOC100505570* | 2.26 | 0.013 |
| 132 | Up | *MIR4442* | 1.92 | 0.013 |
| 133 | Down | *MIR3920* | -2.10 | 0.013 |
| 134 | Up | *GUSBP9* | 2.55 | 0.013 |
| 135 | Up | *SDHAP1* | 3.81 | 0.013 |
| 136 | Up | *SNORD113-1* | 7.86 | 0.013 |
| 137 | Up | *LOC647264* | 2.73 | 0.013 |
| 138 | Down | *OR1S1* | -2.27 | 0.013 |
| 139 | Up | *MPLKIP* | 5.20 | 0.013 |
| 140 | Up | *COX8A* | 2.17 | 0.013 |
| 141 | Down | *OR1L4* | -2.22 | 0.013 |
| 142 | Up | *TPT1* | 3.53 | 0.013 |
| 143 | Up | *SMG1P2* | 2.12 | 0.013 |
| 144 | Down | *CTAGE11P* | -2.02 | 0.014 |
| 145 | Up | *PPIP5K1* | 2.10 | 0.014 |
| 146 | Down | *SNORD115-45* | -2.03 | 0.014 |
| 147 | Up | *IGKV3D-20* | 1.94 | 0.014 |
| 148 | Up | *RAD51D* | 3.76 | 0.014 |
| 149 | Up | *LOC100506123* | 2.49 | 0.014 |
| 150 | Up | *HIST2H2BE* | 1.95 | 0.014 |
| 151 | Down | *LOC105373498* | -1.74 | 0.015 |
| 152 | Up | *YBX1* | 2.05 | 0.015 |
| 153 | Up | *SNORA71A* | 3.74 | 0.015 |
| 154 | Up | *GOLGA8H* | 2.33 | 0.016 |
| 155 | Up | *SMG1P5* | 2.17 | 0.016 |
| 156 | Up | *BASP1* | 2.46 | 0.016 |
| 157 | Up | *BMP5* | 2.73 | 0.016 |
| 158 | Up | *IGLC1* | 3.59 | 0.016 |
| 159 | Down | *LOC101928600* | -2.06 | 0.016 |
| 160 | Up | *CALR* | 3.16 | 0.017 |
| 161 | Up | *HIST2H2AC* | 2.18 | 0.017 |
| 162 | Down | *ATP8* | -3.49 | 0.017 |
| 163 | Up | *KRTAP4-16P* | 2.58 | 0.017 |
| 164 | Up | *TEAD2* | 2.36 | 0.017 |
| 165 | Up | *IGLC2* | 3.77 | 0.018 |
| 166 | Down | *LOC102724148* | -2.37 | 0.018 |
| 167 | Up | *DAB2* | 2.26 | 0.018 |
| 168 | Up | *MIR548L* | 3.33 | 0.018 |
| 169 | Up | *VTRNA1-2* | 4.47 | 0.019 |
| 170 | Down | *COX1* | -3.78 | 0.019 |
| 171 | Down | *TRAJ16* | -2.91 | 0.019 |
| 172 | Down | *TRDJ4* | -2.15 | 0.019 |
| 173 | Up | *MIR4533* | 2.17 | 0.019 |
| 174 | Down | *MIR520A* | -2.17 | 0.020 |
| 175 | Up | *MIR4525* | 7.55 | 0.020 |
| 176 | Up | *HTRA1* | 1.23 | 0.020 |
| 177 | Down | *IGHV2OR16-5* | -2.41 | 0.020 |
| 178 | Down | *IGHV3-53* | -2.52 | 0.021 |
| 179 | Down | *TRAJ61* | -2.01 | 0.021 |
| 180 | Up | *HSPB1* | 1.76 | 0.021 |
| 181 | Up | *IGKV3D-7* | 1.92 | 0.021 |
| 182 | Up | *HIST1H3F* | 3.34 | 0.021 |
| 183 | Up | *SNORD114-28* | 1.69 | 0.022 |
| 184 | Down | *MIR520D* | -2.17 | 0.022 |
| 185 | Up | *F8A1* | 5.79 | 0.022 |
| 186 | Up | *CPM* | 2.17 | 0.023 |
| 187 | Down | *C1QTNF9B-AS1* | -2.96 | 0.023 |
| 188 | Up | *BAGE2* | 4.56 | 0.023 |
| 189 | Up | *HMGN3* | 2.09 | 0.023 |
| 190 | Up | *IGHEP1* | 2.58 | 0.023 |
| 191 | Up | *MT4* | 2.05 | 0.023 |
| 192 | Down | *MIR524* | -2.06 | 0.023 |
| 193 | Up | *LCE3A* | 2.22 | 0.024 |
| 194 | Up | *SEC61B* | 1.84 | 0.024 |
| 195 | Down | *SNORA12* | -5.57 | 0.024 |
| 196 | Up | *ZCWPW2* | 3.96 | 0.024 |
| 197 | Down | *SNORA74B* | -2.09 | 0.024 |
| 198 | Up | *HIST2H2BC* | 6.20 | 0.024 |
| 199 | Up | *IGK* | 2.18 | 0.024 |
| 200 | Down | *MIR3975* | -2.43 | 0.025 |
| 201 | Up | *IGFBP5* | 1.56 | 0.025 |
| 202 | Up | *IGKV3D-15* | 3.07 | 0.026 |
| 203 | Down | *PP13* | -2.03 | 0.026 |
| 204 | Up | *GANC* | 5.62 | 0.026 |
| 205 | Up | *RPL29P2* | 2.26 | 0.027 |
| 206 | Up | *ZEB2* | 3.80 | 0.027 |
| 207 | Up | *HIST1H2BD* | 3.78 | 0.027 |
| 208 | Up | *JAK1* | 1.80 | 0.027 |
| 209 | Up | *GXYLT2* | 1.69 | 0.028 |
| 210 | Up | *SCARNA10* | 2.01 | 0.028 |
| 211 | Up | *IGHG2* | 2.16 | 0.028 |
| 212 | Up | *SNAR-F* | 2.29 | 0.028 |
| 213 | Up | *HIST1H4A* | 2.01 | 0.029 |
| 214 | Up | *MIR1224* | 2.01 | 0.030 |
| 215 | Up | *MAP3K8* | 5.61 | 0.030 |
| 216 | Up | *LOC100132062* | 2.15 | 0.031 |
| 217 | Up | *SNORD13P2* | 1.79 | 0.031 |
| 218 | Up | *IGLV7-46* | 2.03 | 0.031 |
| 219 | Down | *ND4L* | -2.99 | 0.033 |
| 220 | Up | *MIR93* | 2.13 | 0.033 |
| 221 | Up | *MIR1254-1* | 3.30 | 0.033 |
| 222 | Down | *LOC105375068* | -2.06 | 0.033 |
| 223 | Down | *MIR4717* | -2.13 | 0.033 |
| 224 | Up | *IGHD* | 4.58 | 0.034 |
| 225 | Up | *MIR539* | 2.12 | 0.034 |
| 226 | Up | *IGLV3-27* | 2.13 | 0.034 |
| 227 | Up | *IGKC* | 1.78 | 0.035 |
| 228 | Up | *ETV6* | 1.53 | 0.035 |
| 229 | Down | *ND5* | -3.01 | 0.035 |
| 230 | Down | *MIR373* | -2.02 | 0.035 |
| 231 | Up | *COL1A1* | 1.45 | 0.036 |
| 232 | Up | *ZNF250* | 2.32 | 0.036 |
| 233 | Up | *RPS2* | 2.54 | 0.036 |
| 234 | Up | *RPL4* | 2.18 | 0.036 |
| 235 | Up | *LOC100133091* | 3.24 | 0.036 |
| 236 | Up | *UBALD2* | 1.80 | 0.037 |
| 237 | Down | *SCARNA4* | -2.13 | 0.038 |
| 238 | Up | *MIR647* | 2.12 | 0.038 |
| 239 | Up | *PARM1* | 2.31 | 0.039 |
| 240 | Up | *PCBP1* | 2.02 | 0.039 |
| 241 | Up | *MIR4664* | 2.68 | 0.039 |
| 242 | Up | *SNORA60* | 3.14 | 0.039 |
| 243 | Up | *C1S* | 1.57 | 0.040 |
| 244 | Up | *MIR4780* | 2.04 | 0.040 |
| 245 | Down | *PPP2R2B* | -2.06 | 0.040 |
| 246 | Up | *NOMO3* | 2.11 | 0.041 |
| 247 | Up | *FTL* | 3.29 | 0.041 |
| 248 | Up | *DDIT4* | 1.35 | 0.041 |
| 249 | Up | *IGKV1-37* | 2.57 | 0.041 |
| 250 | Down | *LOC105369669* | -2.20 | 0.042 |
| 251 | Up | *LOC105376050* | 2.24 | 0.042 |
| 252 | Up | *LOC105371220* | 3.47 | 0.043 |
| 253 | Up | *RPL21* | 4.69 | 0.043 |
| 254 | Up | *TMEM128* | 2.23 | 0.043 |
| 255 | Up | *SNORD117* | 3.52 | 0.044 |
| 256 | Up | *SNORD11B* | 3.67 | 0.044 |
| 257 | Up | *IGLC7* | 2.50 | 0.044 |
| 258 | Up | *MIR27A* | 3.02 | 0.044 |
| 259 | Down | *MIR2115* | -2.02 | 0.045 |
| 260 | Up | *POSTN* | 1.67 | 0.046 |
| 261 | Up | *ZBTB39* | 2.17 | 0.047 |
| 262 | Down | *MIR513B* | -2.09 | 0.048 |
| 263 | Down | *CYTB* | -2.79 | 0.049 |

“-” in the fold change indicates down regulation.

**Supplementary table S13.** Up-/down-regulated genes (fold change ≥ 2, *P* < 0.05) in the HD-exposed adult FFPE lung tissue (Case, n = 4 vs. Control, n = 5)

| No | Up-/Down-regulation | Gene | Fold change (Case/Control) | *P*-value |
| --- | --- | --- | --- | --- |
| 1 | Up | *HIST1H2BK* | 14.00 | 7.49E-06 |
| 2 | Down | *LINC00508* | -2.33 | 4.32E-05 |
| 3 | Down | *LOC101927414* | -2.74 | 6.31E-05 |
| 4 | Up | *HTRA1* | 2.39 | 0.0001 |
| 5 | Down | *TRBV20OR9-2* | -4.28 | 0.0001 |
| 6 | Up | *IGHG4* | 9.01 | 0.0001 |
| 7 | Up | *OR2L1P* | 3.68 | 0.0001 |
| 8 | Up | *CTSK* | 2.74 | 0.0001 |
| 9 | Up | *SNORD113-1* | 24.21 | 0.0003 |
| 10 | Down | *LOC105373498* | -2.03 | 0.0003 |
| 11 | Up | *C1S* | 2.17 | 0.0004 |
| 12 | Down | *MIR4478* | -3.11 | 0.0005 |
| 13 | Up | *LOXL1* | 2.21 | 0.0007 |
| 14 | Up | *MRPL41* | 2.44 | 0.0007 |
| 15 | Up | *MIR637* | 10.42 | 0.0008 |
| 16 | Up | *MIR130B* | 2.43 | 0.0008 |
| 17 | Up | *THBS2* | 2.71 | 0.001 |
| 18 | Down | *MIR3689D1* | -2.22 | 0.001 |
| 19 | Up | *IGK* | 7.08 | 0.001 |
| 20 | Down | *PMS2P4* | -2.90 | 0.001 |
| 21 | Up | *METRN* | 2.02 | 0.001 |
| 22 | Up | *CST3* | 2.31 | 0.002 |
| 23 | Down | *SNORA80B* | -3.12 | 0.002 |
| 24 | Up | *IGKV3D-7* | 7.75 | 0.002 |
| 25 | Up | *XIST* | 5.55 | 0.002 |
| 26 | Up | *MIR1910* | 2.67 | 0.002 |
| 27 | Up | *VTRNA1-2* | 10.71 | 0.002 |
| 28 | Down | *ND3* | -3.15 | 0.002 |
| 29 | Up | *IGLC2* | 9.98 | 0.002 |
| 30 | Up | *SNORA10* | 3.23 | 0.002 |
| 31 | Up | *MMP2* | 5.17 | 0.002 |
| 32 | Up | *TRAJ21* | 16.77 | 0.002 |
| 33 | Down | *MAGEA1* | -1.77 | 0.003 |
| 34 | Up | *COL1A2* | 3.71 | 0.003 |
| 35 | Up | *GXYLT2* | 3.13 | 0.003 |
| 36 | Up | *LUC7L* | 2.21 | 0.003 |
| 37 | Down | *COX1* | -4.08 | 0.003 |
| 38 | Down | *C1QTNF9B-AS1* | -2.30 | 0.003 |
| 39 | Up | *IGKV1D-33* | 2.21 | 0.003 |
| 40 | Up | *HNRNPA0* | 2.14 | 0.004 |
| 41 | Down | *MCEMP1* | -2.40 | 0.004 |
| 42 | Up | *H1F0* | 2.11 | 0.004 |
| 43 | Up | *ZBTB39* | 1.82 | 0.004 |
| 44 | Down | *MTRNR2L8* | -2.53 | 0.005 |
| 45 | Up | *IGLC1* | 5.86 | 0.005 |
| 46 | Down | *ND5* | -2.25 | 0.005 |
| 47 | Up | *RPS18* | 2.22 | 0.005 |
| 48 | Up | *OR6C4* | 31.91 | 0.005 |
| 49 | Down | *TRAJ59* | -1.76 | 0.005 |
| 50 | Up | *IGKV1-37* | 3.92 | 0.005 |
| 51 | Up | *BGN* | 4.05 | 0.005 |
| 52 | Up | *TIMP1* | 2.97 | 0.005 |
| 53 | Up | *SOX4* | 2.06 | 0.006 |
| 54 | Up | *RPL10A* | 1.61 | 0.006 |
| 55 | Up | *NAT8B* | 2.35 | 0.006 |
| 56 | Up | *PTP4A3* | 2.36 | 0.006 |
| 57 | Up | *SNORD14E* | 3.18 | 0.007 |
| 58 | Up | *IGKV3D-20* | 3.82 | 0.007 |
| 59 | Up | *INTS5* | 3.74 | 0.007 |
| 60 | Down | *ATP8* | -2.38 | 0.007 |
| 61 | Down | *TRAJ16* | -1.71 | 0.008 |
| 62 | Up | *IGHV1-18* | 12.54 | 0.008 |
| 63 | Up | *LUM* | 3.50 | 0.008 |
| 64 | Up | *IGKV1-39* | 4.75 | 0.008 |
| 65 | Up | *H1FX* | 2.17 | 0.008 |
| 66 | Down | *ND1* | -2.97 | 0.008 |
| 67 | Down | *LOC105372587* | -2.31 | 0.008 |
| 68 | Up | *VTRNA1-3* | 6.34 | 0.008 |
| 69 | Up | *MT1X* | 6.27 | 0.009 |
| 70 | Up | *MIRLET7BHG* | 2.81 | 0.009 |
| 71 | Down | *LOC101927329* | -1.58 | 0.009 |
| 72 | Down | *SNORD113-8* | -2.22 | 0.009 |
| 73 | Up | *RNU6ATAC* | 2.22 | 0.009 |
| 74 | Down | *ND4L* | -3.01 | 0.009 |
| 75 | Up | *SNORA71A* | 4.64 | 0.009 |
| 76 | Down | *LOC105376863* | -1.90 | 0.009 |
| 77 | Up | *SAT1* | 2.94 | 0.010 |
| 78 | Down | *MIR4290* | -2.02 | 0.010 |
| 79 | Up | *GALNT1* | 2.07 | 0.010 |
| 80 | Up | *SNORD4B* | 3.05 | 0.010 |
| 81 | Up | *KRTAP4-16P* | 1.66 | 0.010 |
| 82 | Up | *IGHD* | 5.65 | 0.010 |
| 83 | Up | *HIF1A* | 2.71 | 0.010 |
| 84 | Up | *IGHEP1* | 2.47 | 0.010 |
| 85 | Up | *TRAJ36* | 3.13 | 0.011 |
| 86 | Up | *SEC61B* | 2.06 | 0.011 |
| 87 | Up | *CMIP* | 2.32 | 0.011 |
| 88 | Down | *RFX3-AS1* | -1.64 | 0.011 |
| 89 | Down | *COX2* | -2.19 | 0.011 |
| 90 | Up | *IGKV1-16* | 6.88 | 0.011 |
| 91 | Down | *IGHV2OR16-5* | -1.70 | 0.012 |
| 92 | Up | *HSP90AB4P* | 2.32 | 0.012 |
| 93 | Up | *CCNL1* | 2.05 | 0.012 |
| 94 | Up | *RASA4B* | 1.74 | 0.013 |
| 95 | Up | *HIST2H2BE* | 3.03 | 0.013 |
| 96 | Down | *CACNA2D2* | -2.30 | 0.013 |
| 97 | Up | *IGLC7* | 4.15 | 0.013 |
| 98 | Up | *IGKV2-24* | 7.31 | 0.013 |
| 99 | Up | *NIFK* | 2.20 | 0.013 |
| 100 | Up | *POSTN* | 2.95 | 0.013 |
| 101 | Up | *TRAV20* | 2.40 | 0.013 |
| 102 | Up | *TRAJ35* | 6.90 | 0.014 |
| 103 | Up | *CCDC80* | 2.73 | 0.014 |
| 104 | Up | *MIR3650* | 2.03 | 0.014 |
| 105 | Up | *HIST1H2BD* | 3.80 | 0.014 |
| 106 | Up | *DUSP23* | 1.67 | 0.014 |
| 107 | Up | *BASP1* | 3.04 | 0.014 |
| 108 | Up | *IGKV3D-15* | 5.38 | 0.014 |
| 109 | Down | *ND2* | -2.46 | 0.015 |
| 110 | Up | *LUC7L3* | 2.14 | 0.015 |
| 111 | Up | *SNORD13P2* | 2.83 | 0.015 |
| 112 | Down | *KRT17* | -1.82 | 0.015 |
| 113 | Up | *COL6A3* | 3.30 | 0.015 |
| 114 | Up | *IGHV3-48* | 2.74 | 0.016 |
| 115 | Up | *SCARNA21* | 1.74 | 0.016 |
| 116 | Up | *COL3A1* | 3.20 | 0.016 |
| 117 | Up | *MT2A* | 3.33 | 0.016 |
| 118 | Up | *SSH1* | 2.64 | 0.017 |
| 119 | Up | *IGHA1* | 2.09 | 0.017 |
| 120 | Up | *IGLV2-11* | 5.32 | 0.017 |
| 121 | Up | *NAA38* | 2.33 | 0.017 |
| 122 | Up | *STAT1* | 2.64 | 0.017 |
| 123 | Up | *IGKV1-12* | 2.46 | 0.018 |
| 124 | Down | *MIR3689D2* | -1.55 | 0.018 |
| 125 | Up | *SNORD114-2* | 4.14 | 0.019 |
| 126 | Up | *MIR548L* | 2.63 | 0.019 |
| 127 | Up | *SCARNA17* | 3.31 | 0.019 |
| 128 | Up | *MAP3K8* | 7.30 | 0.019 |
| 129 | Up | *SNORD93* | 2.74 | 0.019 |
| 130 | Up | *ETV6* | 2.01 | 0.020 |
| 131 | Up | *ARPC2* | 2.41 | 0.020 |
| 132 | Up | *MIR4442* | 2.23 | 0.021 |
| 133 | Up | *SFRP2* | 3.28 | 0.021 |
| 134 | Up | *HSPB1* | 2.14 | 0.021 |
| 135 | Up | *NSA2* | 2.06 | 0.022 |
| 136 | Up | *DAB2* | 2.00 | 0.022 |
| 137 | Down | *CYTB* | -2.48 | 0.022 |
| 138 | Down | *MIR524* | -1.59 | 0.023 |
| 139 | Up | *MIR4454* | 3.31 | 0.023 |
| 140 | Up | *UBALD2* | 2.18 | 0.023 |
| 141 | Up | *IGHV3-33* | 6.62 | 0.023 |
| 142 | Up | *MIR4730* | 2.01 | 0.024 |
| 143 | Up | *DDIT4* | 2.50 | 0.024 |
| 144 | Down | *MIR3918* | -1.62 | 0.024 |
| 145 | Up | *MPLKIP* | 2.38 | 0.024 |
| 146 | Down | *IGKV1D-27* | -2.23 | 0.024 |
| 147 | Down | *PP13* | -2.08 | 0.024 |
| 148 | Up | *THBS1* | 4.02 | 0.025 |
| 149 | Up | *COPG1* | 2.28 | 0.025 |
| 150 | Up | *C2orf81* | 2.27 | 0.025 |
| 151 | Up | *SNORD41* | 5.30 | 0.025 |
| 152 | Up | *SNORD3C* | 4.83 | 0.025 |
| 153 | Up | *RPL4* | 2.19 | 0.026 |
| 154 | Up | *ZCWPW2* | 3.58 | 0.026 |
| 155 | Up | *ASAP1-IT2* | 2.12 | 0.027 |
| 156 | Up | *GLTSCR2* | 2.07 | 0.027 |
| 157 | Up | *GLUL* | 2.58 | 0.028 |
| 158 | Up | *RPL29P2* | 1.94 | 0.028 |
| 159 | Up | *MMAB* | 4.85 | 0.028 |
| 160 | Down | *AQP4* | -2.07 | 0.028 |
| 161 | Up | *IGFBP5* | 2.01 | 0.028 |
| 162 | Up | *IGKC* | 2.27 | 0.028 |
| 163 | Up | *LOC100133091* | 2.99 | 0.029 |
| 164 | Down | *MIR1321* | -1.74 | 0.029 |
| 165 | Up | *IGLV3-25* | 5.02 | 0.030 |
| 166 | Up | *LOC100131131* | 2.22 | 0.030 |
| 167 | Up | *ZFP36L1* | 2.54 | 0.030 |
| 168 | Up | *SCARNA10* | 1.81 | 0.030 |
| 169 | Down | *MIR200C* | -2.11 | 0.030 |
| 170 | Up | *ZEB2* | 1.96 | 0.031 |
| 171 | Up | *HELB* | 2.35 | 0.031 |
| 172 | Down | *LOC105377042* | -2.07 | 0.033 |
| 173 | Up | *PCBP1* | 2.32 | 0.034 |
| 174 | Up | *HIST2H2BC* | 6.58 | 0.034 |
| 175 | Up | *SNORD11B* | 3.50 | 0.034 |
| 176 | Up | *SNORD3A* | 4.57 | 0.034 |
| 177 | Up | *SNORD3B-1* | 4.56 | 0.034 |
| 178 | Up | *SNORD35B* | 1.65 | 0.034 |
| 179 | Up | *SNORD114-28* | 5.76 | 0.034 |
| 180 | Down | *LOC105375068* | -1.55 | 0.035 |
| 181 | Up | *NEAT1* | 8.19 | 0.035 |
| 182 | Up | *IGKV1-5* | 5.14 | 0.035 |
| 183 | Up | *IGF1* | 2.51 | 0.035 |
| 184 | Up | *IGHV1-69* | 4.06 | 0.036 |
| 185 | Up | *CD248* | 3.18 | 0.037 |
| 186 | Up | *HILPDA* | 2.15 | 0.039 |
| 187 | Up | *IGKV2D-28* | 3.81 | 0.039 |
| 188 | Down | *RTKN2* | -2.10 | 0.039 |
| 189 | Up | *NBPF12* | 2.05 | 0.039 |
| 190 | Up | *TPT1* | 1.64 | 0.040 |
| 191 | Up | *RAD51D* | 3.10 | 0.040 |
| 192 | Up | *SNORA60* | 6.50 | 0.040 |
| 193 | Up | *MIR4780* | 1.44 | 0.040 |
| 194 | Down | *SLC6A4* | -3.26 | 0.040 |
| 195 | Down | *IGHV3-53* | -2.02 | 0.041 |
| 196 | Up | *IGKV2D-24* | 5.72 | 0.041 |
| 197 | Down | *OR1L4* | -1.56 | 0.042 |
| 198 | Up | *SNORD113-9* | 2.67 | 0.043 |
| 199 | Up | *EEF2* | 2.10 | 0.043 |
| 200 | Up | *JAK1* | 2.04 | 0.043 |
| 201 | Up | *SERPINF1* | 3.33 | 0.044 |
| 202 | Down | *MIR23A* | -2.46 | 0.046 |
| 203 | Up | *IGLV2-18* | 5.53 | 0.047 |
| 204 | Up | *IGHM* | 5.74 | 0.047 |
| 205 | Up | *SNORD114-25* | 5.97 | 0.047 |
| 206 | Up | *TEAD2* | 1.67 | 0.047 |
| 207 | Up | *COL1A1* | 2.09 | 0.050 |

“-” in the fold change indicates down regulation.

**Supplementary table S14.** Panther pathway analysis based on significantly interacting genes with differentially expressed genes in children and adults with HDLI

| Adults |  |  |  |
| --- | --- | --- | --- |
| Term | P-value | Adjusted P-value | Genes |
| Apoptosis signaling pathway_Homo sapiens_P00006 | 9.07E-07 | 3.90E-05 | JUN;PRKCE;CYCS;BAX;ATF7;TP53;ATF3;BCL2L1 |
| CCKR signaling map ST_Homo sapiens_P06959 | 0.0002 | 0.005 | JUN;PRKCE;BAX;PRKD2;TACR1;MMP9;BCL2L1 |
| EGF receptor signaling pathway_Homo sapiens_P00018 | 0.001 | 0.019 | ERBB4;PRKCE;PRKD2;YWHAZ;YWHAG |
| Alzheimer disease-presenilin pathway_Homo sapiens_P00004 | 0.006 | 0.068 | APP;ERBB4;MMP2;MMP9 |
| Huntington disease_Homo sapiens_P00029 | 0.01 | 0.117 | JUN;NCOR1;BAX;TP53 |
| Wnt signaling pathway_Homo sapiens_P00057 | 0.02 | 0.120 | SMARCC1;HDAC3;PRKCE;CSNK2A2;SMAD5;TP53 |
| Angiogenesis_Homo sapiens_P00005 | 0.02 | 0.120 | JUN;PRKCE;PRKD2;JAK1 |
| Parkinson disease_Homo sapiens_P00049 | 0.02 | 0.120 | CSNK2A2;YWHAZ;YWHAG |
| TGF-beta signaling pathway_Homo sapiens_P00052 | 0.03 | 0.132 | JUN;SMAD3;SMAD5 |
| FGF signaling pathway_Homo sapiens_P00021 | 0.04 | 0.133 | PRKCE;YWHAZ;YWHAG |
|  |  |  |  |
| Children |  |  |  |
| Term | P-value | Adjusted P-value | Genes |
| EGF receptor signaling pathway_Homo sapiens_P00018 | 3.80E-05 | 0.002 | STAT5B;ERBB3;SHC1;GAB1;AKT1;RRAS2;PLCG1;CBL |
| CCKR signaling map ST_Homo sapiens_P06959 | 0.0007 | 0.02 | SRC;SHC1;RHEB;AKT1;PTPN11;PLCG1;CRK;BCAR1 |
| p53 pathway_Homo sapiens_P00059 | 0.001 | 0.02 | CDKN1A;SIN3A;AKT1;EP300;TRAF2 |
| Integrin signalling pathway_Homo sapiens_P00034 | 0.002 | 0.02 | SRC;SHC1;ITGA3;FN1;FLNB;CRK;BCAR1 |
| Ubiquitin proteasome pathway_Homo sapiens_P00060 | 0.001 | 0.02 | UBE2E3;UBE2E1;UBE2D1;UBE2G2 |
| Interleukin signaling pathway_Homo sapiens_P00036 | 0.003 | 0.03 | STAT5B;CDKN1A;SHC1;AKT1;IRS2 |
| FGF signaling pathway_Homo sapiens_P00021 | 0.006 | 0.04 | SHC1;AKT1;FRS2;PLCG1;FGFR3 |
| Angiogenesis_Homo sapiens_P00005 | 0.006 | 0.04 | SRC;SHC1;AKT1;FRS2;PLCG1;CRK |
| Insulin/IGF pathway-protein kinase B signaling cascade_Homo sapiens_P00033 | 0.007 | 0.04 | INSR;AKT1;IRS2 |
| Apoptosis signaling pathway_Homo sapiens_P00006 | 0.03 | 0.14 | HSPA8;REL;AKT1;TRAF2 |
| PI3 kinase pathway_Homo sapiens_P00048 | 0.01 | 0.07 | INSR;GNAQ;AKT1 |

**Supplementary table S15.** Reactome pathway analysis based on significantly interacting genes with differentially expressed genes in adults with HDLI (*P* < 0.05)

| **Term** | **Overlap** | **P-value** | **Adjusted P-value** | **Genes** |
| --- | --- | --- | --- | --- |
| Gene Expression_Homo sapiens_R-HSA-74160 | 41/1631 | 1.58E-12 | 7.97E-10 | MEAF6;THRA;DHX9;RPLP0;SRSF1;ZC3H8;HNRNPR;AURKA;EFTUD2;MTA1;SNRNP70;E2F1;RPS3;IGF2BP3;HNRNPA1;UTP14A;YWHAG;UPF1;JUN;NOP14;G6PD;SMAD3;SF3A2;CSNK2A2;YWHAZ;CDK9;EEF1A1;EEF1G;LSM7;NCOR1;HNRNPUL1;HNRNPH1;EIF3I;CYCS;BAX;SNRPA1;RNPS1;SNRPF;EIF3D;TP53;RPL26L1 |
| mRNA Splicing - Major Pathway_Homo sapiens_R-HSA-72163 | 13/134 | 2.15E-11 | 5.40E-09 | SF3A2;DHX9;SRSF1;HNRNPR;EFTUD2;LSM7;HNRNPUL1;SNRNP70;HNRNPH1;SNRPA1;RNPS1;SNRPF;HNRNPA1 |
| mRNA Splicing_Homo sapiens_R-HSA-72172 | 13/144 | 5.37E-11 | 9.00E-09 | SF3A2;DHX9;SRSF1;HNRNPR;EFTUD2;LSM7;HNRNPUL1;SNRNP70;HNRNPH1;SNRPA1;RNPS1;SNRPF;HNRNPA1 |
| Processing of Capped Intron-Containing Pre-mRNA_Homo sapiens_R-HSA-72203 | 13/193 | 2.05E-09 | 2.58E-07 | SF3A2;DHX9;SRSF1;HNRNPR;EFTUD2;LSM7;HNRNPUL1;SNRNP70;HNRNPH1;SNRPA1;RNPS1;SNRPF;HNRNPA1 |
| Intrinsic Pathway for Apoptosis_Homo sapiens_R-HSA-109606 | 7/42 | 2.29E-08 | 2.30E-06 | E2F1;CYCS;BAX;YWHAZ;TP53;YWHAG;BCL2L1 |
| Organelle biogenesis and maintenance_Homo sapiens_R-HSA-1852241 | 14/326 | 1.46E-07 | 1.22E-05 | CCT2;HDAC3;MRPS33;MRPS11;MRPS12;MRPS31;MRPS10;MRPS5;MRPL4;NCOR1;TCP1;CYCS;YWHAG;CCT4 |
| Mitochondrial translation elongation_Homo sapiens_R-HSA-5389840 | 7/84 | 2.96E-06 | 0.00017 | MRPL4;MRPS33;MRPS11;MRPS12;MRPS31;MRPS10;MRPS5 |
| Mitochondrial translation termination_Homo sapiens_R-HSA-5419276 | 7/84 | 2.96E-06 | 0.00017 | MRPL4;MRPS33;MRPS11;MRPS12;MRPS31;MRPS10;MRPS5 |
| Mitochondrial translation initiation_Homo sapiens_R-HSA-5368286 | 7/84 | 2.96E-06 | 0.00017 | MRPL4;MRPS33;MRPS11;MRPS12;MRPS31;MRPS10;MRPS5 |
| Mitochondrial translation_Homo sapiens_R-HSA-5368287 | 7/90 | 4.71E-06 | 0.00024 | MRPL4;MRPS33;MRPS11;MRPS12;MRPS31;MRPS10;MRPS5 |
| Association of TriC/CCT with target proteins during biosynthesis_Homo sapiens_R-HSA-390471 | 5/39 | 9.75E-06 | 0.00045 | CCT2;HDAC3;TCP1;TP53;CCT4 |
| Transcriptional Regulation by TP53_Homo sapiens_R-HSA-3700989 | 12/348 | 1.12E-05 | 0.00047 | CDK9;JUN;G6PD;MEAF6;CSNK2A2;E2F1;CYCS;BAX;YWHAZ;TP53;YWHAG;AURKA |
| Apoptosis_Homo sapiens_R-HSA-109581 | 8/163 | 2.94E-05 | 0.0011 | E2F1;CYCS;BAX;HIST1H1A;YWHAZ;TP53;YWHAG;BCL2L1 |
| Programmed Cell Death_Homo sapiens_R-HSA-5357801 | 8/166 | 3.35E-05 | 0.0012 | E2F1;CYCS;BAX;HIST1H1A;YWHAZ;TP53;YWHAG;BCL2L1 |
| Folding of actin by CCT/TriC_Homo sapiens_R-HSA-390450 | 3/10 | 4.59E-05 | 0.0015 | CCT2;TCP1;CCT4 |
| Activation of BH3-only proteins_Homo sapiens_R-HSA-114452 | 4/29 | 5.92E-05 | 0.0019 | E2F1;YWHAZ;TP53;YWHAG |
| Chaperonin-mediated protein folding_Homo sapiens_R-HSA-390466 | 6/95 | 7.50E-05 | 0.0022 | CCT2;HDAC3;CSNK2A2;TCP1;TP53;CCT4 |
| Protein folding_Homo sapiens_R-HSA-391251 | 6/101 | 0.00011 | 0.0029 | CCT2;HDAC3;CSNK2A2;TCP1;TP53;CCT4 |
| Metabolism of proteins_Homo sapiens_R-HSA-392499 | 20/1074 | 0.00013 | 0.0034 | CCT2;APP;HDAC3;MMP2;RPLP0;CSNK2A2;AURKA;EEF1A1;EEF1G;MTA1;OS9;EIF3I;TCP1;RPS3;TOP1;EIF3D;TP53;ATF3;RPL26L1;CCT4 |
| Translation_Homo sapiens_R-HSA-72766 | 7/151 | 0.00013 | 0.0034 | EEF1A1;EEF1G;EIF3I;RPLP0;RPS3;EIF3D;RPL26L1 |
| Respiratory electron transport, ATP synthesis by chemiosmotic coupling, and heat production by uncoupling proteins._Homo sapiens_R-HSA-163200 | 6/109 | 0.00016 | 0.0038 | UQCRB;NDUFB11;UQCRQ;NDUFS3;CYCS;ATP5F1 |
| Cooperation of PDCL (PhLP1) and TRiC/CCT in G-protein beta folding_Homo sapiens_R-HSA-6814122 | 4/42 | 0.00026 | 0.0059 | CCT2;CSNK2A2;TCP1;CCT4 |
| TP53 Regulates Transcription of Genes Involved in G2 Cell Cycle Arrest_Homo sapiens_R-HSA-6804114 | 3/18 | 0.00030 | 0.0065 | BAX;TP53;AURKA |
| TP53 Regulates Metabolic Genes_Homo sapiens_R-HSA-5628897 | 5/84 | 0.00040 | 0.0084 | G6PD;CYCS;YWHAZ;TP53;YWHAG |
| TP53 Regulates Transcription of Cell Cycle Genes_Homo sapiens_R-HSA-6791312 | 4/48 | 0.00043 | 0.0088 | E2F1;BAX;TP53;AURKA |
| Respiratory electron transport_Homo sapiens_R-HSA-611105 | 5/88 | 0.00050 | 0.0096 | UQCRB;NDUFB11;UQCRQ;NDUFS3;CYCS |
| Eukaryotic Translation Elongation_Homo sapiens_R-HSA-156842 | 5/89 | 0.00052 | 0.0097 | EEF1A1;EEF1G;RPLP0;RPS3;RPL26L1 |
| BBSome-mediated cargo-targeting to cilium_Homo sapiens_R-HSA-5620922 | 3/23 | 0.00063 | 0.011 | CCT2;TCP1;CCT4 |
| Formation of a pool of free 40S subunits_Homo sapiens_R-HSA-72689 | 5/96 | 0.00074 | 0.013 | EIF3I;RPLP0;RPS3;EIF3D;RPL26L1 |
| SMAD2/3 Phosphorylation Motif Mutants in Cancer_Homo sapiens_R-HSA-3304356 | 2/6 | 0.00080 | 0.013 | TGFB1;SMAD3 |
| Formation of tubulin folding intermediates by CCT/TriC_Homo sapiens_R-HSA-389960 | 3/25 | 0.00081 | 0.013 | CCT2;TCP1;CCT4 |
| The citric acid (TCA) cycle and respiratory electron transport_Homo sapiens_R-HSA-1428517 | 6/153 | 0.00098 | 0.014 | UQCRB;NDUFB11;UQCRQ;NDUFS3;CYCS;ATP5F1 |
| Prefoldin mediated transfer of substrate to CCT/TriC_Homo sapiens_R-HSA-389957 | 3/27 | 0.0010 | 0.014 | CCT2;TCP1;CCT4 |
| Generic Transcription Pathway_Homo sapiens_R-HSA-212436 | 15/812 | 0.0010 | 0.014 | JUN;G6PD;SMAD3;MEAF6;THRA;CSNK2A2;YWHAZ;AURKA;CDK9;NCOR1;E2F1;CYCS;BAX;TP53;YWHAG |
| Loss of Function of SMAD2/3 in Cancer_Homo sapiens_R-HSA-3304349 | 2/7 | 0.0011 | 0.014 | TGFB1;SMAD3 |
| Loss of Function of TGFBR1 in Cancer_Homo sapiens_R-HSA-3656534 | 2/7 | 0.0011 | 0.014 | TGFB1;SMAD3 |
| SMAD2/3 MH2 Domain Mutants in Cancer_Homo sapiens_R-HSA-3315487 | 2/7 | 0.0011 | 0.014 | TGFB1;SMAD3 |
| TGFBR1 KD Mutants in Cancer_Homo sapiens_R-HSA-3656532 | 2/7 | 0.0011 | 0.014 | TGFB1;SMAD3 |
| 3' -UTR-mediated translational regulation_Homo sapiens_R-HSA-157279 | 5/106 | 0.0012 | 0.014 | EIF3I;RPLP0;RPS3;EIF3D;RPL26L1 |
| L13a-mediated translational silencing of Ceruloplasmin expression_Homo sapiens_R-HSA-156827 | 5/106 | 0.0012 | 0.014 | EIF3I;RPLP0;RPS3;EIF3D;RPL26L1 |
| Nonsense Mediated Decay (NMD) enhanced by the Exon Junction Complex (EJC)_Homo sapiens_R-HSA-975957 | 5/106 | 0.0012 | 0.014 | UPF1;RPLP0;RPS3;RNPS1;RPL26L1 |
| Nonsense-Mediated Decay (NMD)_Homo sapiens_R-HSA-927802 | 5/106 | 0.0012 | 0.014 | UPF1;RPLP0;RPS3;RNPS1;RPL26L1 |
| GTP hydrolysis and joining of the 60S ribosomal subunit_Homo sapiens_R-HSA-72706 | 5/107 | 0.0012 | 0.014 | EIF3I;RPLP0;RPS3;EIF3D;RPL26L1 |
| Extracellular matrix organization_Homo sapiens_R-HSA-1474244 | 8/283 | 0.0012 | 0.014 | COL1A1;TGFB1;COL14A1;ELN;MMP2;ITGB3;COL6A1;MMP9 |
| Signaling by TGF-beta Receptor Complex in Cancer_Homo sapiens_R-HSA-3304351 | 2/8 | 0.0015 | 0.015 | TGFB1;SMAD3 |
| Activation of PUMA and translocation to mitochondria_Homo sapiens_R-HSA-139915 | 2/8 | 0.0015 | 0.015 | E2F1;TP53 |
| NR1D1 (REV-ERBA) represses gene expression_Homo sapiens_R-HSA-1368071 | 2/8 | 0.0015 | 0.015 | NCOR1;HDAC3 |
| Chromatin modifying enzymes_Homo sapiens_R-HSA-3247509 | 7/226 | 0.0015 | 0.015 | PRMT6;SMARCC1;ING3;NCOR1;MTA1;HDAC3;MEAF6 |
| Chromatin organization_Homo sapiens_R-HSA-4839726 | 7/226 | 0.0015 | 0.015 | PRMT6;SMARCC1;ING3;NCOR1;MTA1;HDAC3;MEAF6 |
| Cap-dependent Translation Initiation_Homo sapiens_R-HSA-72737 | 5/114 | 0.0016 | 0.016 | EIF3I;RPLP0;RPS3;EIF3D;RPL26L1 |
| Eukaryotic Translation Initiation_Homo sapiens_R-HSA-72613 | 5/114 | 0.0016 | 0.016 | EIF3I;RPLP0;RPS3;EIF3D;RPL26L1 |
| Cooperation of Prefoldin and TriC/CCT in actin and tubulin folding_Homo sapiens_R-HSA-389958 | 3/32 | 0.0017 | 0.016 | CCT2;TCP1;CCT4 |
| Signaling by TGF-beta Receptor Complex_Homo sapiens_R-HSA-170834 | 4/73 | 0.0021 | 0.020 | CDK9;TGFB1;SMAD3;NCOR1 |
| Signaling by NOTCH_Homo sapiens_R-HSA-157118 | 5/122 | 0.0021 | 0.020 | JUN;NCOR1;HDAC3;E2F1;TP53 |
| Collagen degradation_Homo sapiens_R-HSA-1442490 | 3/39 | 0.0030 | 0.027 | COL14A1;MMP2;MMP9 |
| Transcriptional activation of mitochondrial biogenesis_Homo sapiens_R-HSA-2151201 | 3/40 | 0.0032 | 0.029 | NCOR1;HDAC3;CYCS |
| Chk1/Chk2(Cds1) mediated inactivation of Cyclin B:Cdk1 complex_Homo sapiens_R-HSA-75035 | 2/12 | 0.0034 | 0.029 | YWHAZ;YWHAG |
| Pre-NOTCH Transcription and Translation_Homo sapiens_R-HSA-1912408 | 3/41 | 0.0034 | 0.029 | JUN;E2F1;TP53 |
| Elastic fibre formation_Homo sapiens_R-HSA-1566948 | 3/41 | 0.0034 | 0.029 | TGFB1;ELN;ITGB3 |
| Peptide chain elongation_Homo sapiens_R-HSA-156902 | 4/84 | 0.0035 | 0.029 | EEF1A1;RPLP0;RPS3;RPL26L1 |
| Collagen formation_Homo sapiens_R-HSA-1474290 | 4/85 | 0.0037 | 0.030 | COL1A1;COL14A1;COL6A1;MMP9 |
| TP53 Regulates Transcription of Genes Involved in G1 Cell Cycle Arrest_Homo sapiens_R-HSA-6804116 | 2/13 | 0.0040 | 0.032 | E2F1;TP53 |
| DEx/H-box helicases activate type I IFN and inflammatory cytokines production_Homo sapiens_R-HSA-3134963 | 2/13 | 0.0040 | 0.032 | APP;DHX9 |
| Advanced glycosylation endproduct receptor signaling_Homo sapiens_R-HSA-879415 | 2/13 | 0.0040 | 0.032 | LGALS3;APP |
| Transcriptional activity of SMAD2/SMAD3:SMAD4 heterotrimer_Homo sapiens_R-HSA-2173793 | 3/44 | 0.0042 | 0.033 | CDK9;SMAD3;NCOR1 |
| Nonsense Mediated Decay (NMD) independent of the Exon Junction Complex (EJC)_Homo sapiens_R-HSA-975956 | 4/89 | 0.0043 | 0.033 | UPF1;RPLP0;RPS3;RPL26L1 |
| SUMOylation of transcription factors_Homo sapiens_R-HSA-3232118 | 2/14 | 0.0047 | 0.035 | MTA1;TP53 |
| Activation of BAD and translocation to mitochondria_Homo sapiens_R-HSA-111447 | 2/15 | 0.0054 | 0.040 | YWHAZ;YWHAG |
| SUMO E3 ligases SUMOylate target proteins_Homo sapiens_R-HSA-3108232 | 4/96 | 0.0057 | 0.040 | MTA1;TOP1;TP53;AURKA |
| Developmental Biology_Homo sapiens_R-HSA-1266738 | 13/786 | 0.0057 | 0.040 | JUN;TGFB1;SMAD3;HDAC3;EPAS1;MMP2;ITGB3;CSNK2A2;MMP9;NCOR1;ERBB4;COL6A1;JAK1 |
| Diseases of signal transduction_Homo sapiens_R-HSA-5663202 | 7/288 | 0.0057 | 0.040 | TGFB1;SMAD3;CPSF6;NCOR1;HDAC3;OS9;ERBB4 |
| Mitochondrial biogenesis_Homo sapiens_R-HSA-1592230 | 3/49 | 0.0057 | 0.040 | NCOR1;HDAC3;CYCS |
| Formation of the ternary complex, and subsequently, the 43S complex_Homo sapiens_R-HSA-72695 | 3/50 | 0.0060 | 0.042 | EIF3I;RPS3;EIF3D |
| Cargo trafficking to the periciliary membrane_Homo sapiens_R-HSA-5620920 | 3/51 | 0.0064 | 0.043 | CCT2;TCP1;CCT4 |
| mRNA Splicing - Minor Pathway_Homo sapiens_R-HSA-72165 | 3/52 | 0.0067 | 0.044 | EFTUD2;SRSF1;SNRPF |
| SUMOylation_Homo sapiens_R-HSA-2990846 | 4/101 | 0.0068 | 0.044 | MTA1;TOP1;TP53;AURKA |
| G1/S-Specific Transcription_Homo sapiens_R-HSA-69205 | 2/17 | 0.0069 | 0.044 | DHFR;E2F1 |
| Formation of Senescence-Associated Heterochromatin Foci (SAHF)_Homo sapiens_R-HSA-2559584 | 2/17 | 0.0069 | 0.044 | HIST1H1A;TP53 |
| Inflammasomes_Homo sapiens_R-HSA-622312 | 2/17 | 0.0069 | 0.044 | APP;BCL2L1 |
| Signal Transduction_Homo sapiens_R-HSA-162582 | 29/2465 | 0.0074 | 0.046 | APP;HDAC3;EPAS1;ITGB3;LPAR1;STK4;RTN4;STK3;OS9;ERBB4;E2F1;HNRNPA1;YWHAG;JAK1;JUN;TGFB1;SMAD3;MMP2;PRKCE;CSNK2A2;TACR1;MMP9;SMAD5;YWHAZ;CDK9;NCOR1;HNRNPH1;COL6A1;TP53 |
| Assembly of collagen fibrils and other multimeric structures_Homo sapiens_R-HSA-2022090 | 3/54 | 0.0075 | 0.046 | COL1A1;COL6A1;MMP9 |
| Major pathway of rRNA processing in the nucleolus_Homo sapiens_R-HSA-6791226 | 5/166 | 0.0079 | 0.049 | NOP14;RPLP0;RPS3;UTP14A;RPL26L1 |
| TP53 Regulates Transcription of Genes Involved in Cytochrome C Release_Homo sapiens_R-HSA-6803204 | 2/19 | 0.0086 | 0.051 | BAX;TP53 |
| Pre-NOTCH Expression and Processing_Homo sapiens_R-HSA-1912422 | 3/57 | 0.0087 | 0.051 | JUN;E2F1;TP53 |
| Ribosomal scanning and start codon recognition_Homo sapiens_R-HSA-72702 | 3/57 | 0.0087 | 0.051 | EIF3I;RPS3;EIF3D |
| Translation initiation complex formation_Homo sapiens_R-HSA-72649 | 3/57 | 0.0087 | 0.051 | EIF3I;RPS3;EIF3D |
| Activation of the mRNA upon binding of the cap-binding complex and eIFs, and subsequent binding to 43S_Homo sapiens_R-HSA-72662 | 3/58 | 0.0091 | 0.053 | EIF3I;RPS3;EIF3D |
| Syndecan interactions_Homo sapiens_R-HSA-3000170 | 2/20 | 0.0095 | 0.054 | TGFB1;ITGB3 |
| Signaling by Hippo_Homo sapiens_R-HSA-2028269 | 2/20 | 0.0095 | 0.054 | STK4;STK3 |
| HDACs deacetylate histones_Homo sapiens_R-HSA-3214815 | 3/60 | 0.010 | 0.054 | NCOR1;MTA1;HDAC3 |
| Cleavage of Growing Transcript in the Termination Region_Homo sapiens_R-HSA-109688 | 3/60 | 0.010 | 0.054 | SRSF1;RNPS1;SNRPF |
| Post-Elongation Processing of the Transcript_Homo sapiens_R-HSA-76044 | 3/60 | 0.010 | 0.054 | SRSF1;RNPS1;SNRPF |
| RNA Polymerase II Transcription Termination_Homo sapiens_R-HSA-73856 | 3/60 | 0.010 | 0.054 | SRSF1;RNPS1;SNRPF |
| RIP-mediated NFkB activation via ZBP1_Homo sapiens_R-HSA-1810476 | 2/21 | 0.010 | 0.055 | APP;DHX9 |
| Signal transduction by L1_Homo sapiens_R-HSA-445144 | 2/21 | 0.010 | 0.055 | ITGB3;CSNK2A2 |
| TP53 Regulates Transcription of DNA Repair Genes_Homo sapiens_R-HSA-6796648 | 3/61 | 0.010 | 0.055 | CDK9;JUN;TP53 |
| rRNA processing_Homo sapiens_R-HSA-72312 | 5/180 | 0.011 | 0.057 | NOP14;RPLP0;RPS3;UTP14A;RPL26L1 |
| Collagen biosynthesis and modifying enzymes_Homo sapiens_R-HSA-1650814 | 3/63 | 0.011 | 0.058 | COL1A1;COL14A1;COL6A1 |
| Platelet activation, signaling and aggregation_Homo sapiens_R-HSA-76002 | 6/253 | 0.011 | 0.058 | APP;TGFB1;ITGB3;PRKCE;YWHAZ;JAK1 |
| Downregulation of SMAD2/3:SMAD4 transcriptional activity_Homo sapiens_R-HSA-2173795 | 2/23 | 0.012 | 0.062 | SMAD3;NCOR1 |
| Regulation of TP53 Activity through Acetylation_Homo sapiens_R-HSA-6804758 | 2/23 | 0.012 | 0.062 | MEAF6;TP53 |
| TRAF6 mediated NF-kB activation_Homo sapiens_R-HSA-933542 | 2/24 | 0.013 | 0.067 | APP;DHX9 |
| RNA Polymerase II Transcription_Homo sapiens_R-HSA-73857 | 4/124 | 0.014 | 0.067 | CDK9;SRSF1;RNPS1;SNRPF |
| TAK1 activates NFkB by phosphorylation and activation of IKKs complex_Homo sapiens_R-HSA-445989 | 2/26 | 0.016 | 0.074 | APP;DHX9 |
| Downregulation of TGF-beta receptor signaling_Homo sapiens_R-HSA-2173788 | 2/26 | 0.016 | 0.074 | TGFB1;SMAD3 |
| ZBP1(DAI) mediated induction of type I IFNs_Homo sapiens_R-HSA-1606322 | 2/26 | 0.016 | 0.074 | APP;DHX9 |
| FGFR2 alternative splicing_Homo sapiens_R-HSA-6803529 | 2/26 | 0.016 | 0.074 | HNRNPH1;HNRNPA1 |
| TRAF6 Mediated Induction of proinflammatory cytokines_Homo sapiens_R-HSA-168180 | 3/72 | 0.016 | 0.076 | APP;JUN;DHX9 |
| RORA activates gene expression_Homo sapiens_R-HSA-1368082 | 2/27 | 0.017 | 0.078 | NCOR1;HDAC3 |
| Disease_Homo sapiens_R-HSA-1643685 | 11/725 | 0.019 | 0.087 | CDK9;TGFB1;SMAD3;CPSF6;NCOR1;HDAC3;OS9;ERBB4;RPLP0;RPS3;RPL26L1 |
| Cellular responses to stress_Homo sapiens_R-HSA-2262752 | 7/367 | 0.020 | 0.089 | EEF1A1;JUN;EPAS1;E2F1;CYCS;HIST1H1A;TP53 |
| G2/M DNA damage checkpoint_Homo sapiens_R-HSA-69473 | 3/78 | 0.020 | 0.091 | YWHAZ;TP53;YWHAG |
| Molecules associated with elastic fibres_Homo sapiens_R-HSA-2129379 | 2/30 | 0.021 | 0.092 | TGFB1;ITGB3 |
| Transcriptional regulation of white adipocyte differentiation_Homo sapiens_R-HSA-381340 | 3/79 | 0.021 | 0.092 | TGFB1;NCOR1;HDAC3 |
| MyD88 cascade initiated on plasma membrane_Homo sapiens_R-HSA-975871 | 3/82 | 0.023 | 0.097 | APP;JUN;DHX9 |
| Toll Like Receptor 10 (TLR10) Cascade_Homo sapiens_R-HSA-168142 | 3/82 | 0.023 | 0.097 | APP;JUN;DHX9 |
| Toll Like Receptor 5 (TLR5) Cascade_Homo sapiens_R-HSA-168176 | 3/82 | 0.023 | 0.097 | APP;JUN;DHX9 |
| Oncogene Induced Senescence_Homo sapiens_R-HSA-2559585 | 2/32 | 0.023 | 0.097 | E2F1;TP53 |
| SMAD2/SMAD3:SMAD4 heterotrimer regulates transcription_Homo sapiens_R-HSA-2173796 | 2/32 | 0.023 | 0.097 | CDK9;SMAD3 |
| TGF-beta receptor signaling activates SMADs_Homo sapiens_R-HSA-2173789 | 2/32 | 0.023 | 0.097 | TGFB1;SMAD3 |
| Activation of Matrix Metalloproteinases_Homo sapiens_R-HSA-1592389 | 2/32 | 0.023 | 0.097 | MMP2;MMP9 |
| TRAF6 mediated induction of NFkB and MAP kinases upon TLR7/8 or 9 activation_Homo sapiens_R-HSA-975138 | 3/83 | 0.024 | 0.097 | APP;JUN;DHX9 |
| Influenza Infection_Homo sapiens_R-HSA-168254 | 4/147 | 0.024 | 0.097 | TGFB1;RPLP0;RPS3;RPL26L1 |
| Viral mRNA Translation_Homo sapiens_R-HSA-192823 | 3/84 | 0.024 | 0.099 | RPLP0;RPS3;RPL26L1 |
| E2F mediated regulation of DNA replication_Homo sapiens_R-HSA-113510 | 2/33 | 0.025 | 0.099 | DHFR;E2F1 |
| MyD88 dependent cascade initiated on endosome_Homo sapiens_R-HSA-975155 | 3/85 | 0.025 | 0.10 | APP;JUN;DHX9 |
| Toll Like Receptor 7/8 (TLR7/8) Cascade_Homo sapiens_R-HSA-168181 | 3/85 | 0.025 | 0.10 | APP;JUN;DHX9 |
| Regulation of TP53 Activity_Homo sapiens_R-HSA-5633007 | 4/151 | 0.026 | 0.10 | MEAF6;CSNK2A2;TP53;AURKA |
| Striated Muscle Contraction_Homo sapiens_R-HSA-390522 | 2/34 | 0.026 | 0.10 | TNNT1;TPM1 |
| Selenocysteine synthesis_Homo sapiens_R-HSA-2408557 | 3/87 | 0.027 | 0.10 | RPLP0;RPS3;RPL26L1 |
| Eukaryotic Translation Termination_Homo sapiens_R-HSA-72764 | 3/87 | 0.027 | 0.10 | RPLP0;RPS3;RPL26L1 |
| Toll Like Receptor 9 (TLR9) Cascade_Homo sapiens_R-HSA-168138 | 3/88 | 0.028 | 0.11 | APP;JUN;DHX9 |
| Activation of anterior HOX genes in hindbrain development during early embryogenesis_Homo sapiens_R-HSA-5617472 | 3/89 | 0.028 | 0.11 | JUN;NCOR1;HDAC3 |
| Activation of HOX genes during differentiation_Homo sapiens_R-HSA-5619507 | 3/89 | 0.028 | 0.11 | JUN;NCOR1;HDAC3 |
| Regulation of TP53 Activity through Phosphorylation_Homo sapiens_R-HSA-6804756 | 3/89 | 0.028 | 0.11 | CSNK2A2;TP53;AURKA |
| Oxidative Stress Induced Senescence_Homo sapiens_R-HSA-2559580 | 3/91 | 0.030 | 0.11 | JUN;E2F1;TP53 |
| MyD88:Mal cascade initiated on plasma membrane_Homo sapiens_R-HSA-166058 | 3/92 | 0.031 | 0.11 | APP;JUN;DHX9 |
| Toll Like Receptor 2 (TLR2) Cascade_Homo sapiens_R-HSA-181438 | 3/92 | 0.031 | 0.11 | APP;JUN;DHX9 |
| Toll Like Receptor TLR1:TLR2 Cascade_Homo sapiens_R-HSA-168179 | 3/92 | 0.031 | 0.11 | APP;JUN;DHX9 |
| Toll Like Receptor TLR6:TLR2 Cascade_Homo sapiens_R-HSA-168188 | 3/92 | 0.031 | 0.11 | APP;JUN;DHX9 |
| Cellular Senescence_Homo sapiens_R-HSA-2559583 | 4/161 | 0.032 | 0.11 | JUN;E2F1;HIST1H1A;TP53 |
| Nuclear signaling by ERBB4_Homo sapiens_R-HSA-1251985 | 2/39 | 0.034 | 0.12 | NCOR1;ERBB4 |
| MyD88-independent TLR3/TLR4 cascade_Homo sapiens_R-HSA-166166 | 3/97 | 0.035 | 0.12 | APP;JUN;DHX9 |
| Toll Like Receptor 3 (TLR3) Cascade_Homo sapiens_R-HSA-168164 | 3/97 | 0.035 | 0.12 | APP;JUN;DHX9 |
| TRIF-mediated TLR3/TLR4 signaling_Homo sapiens_R-HSA-937061 | 3/97 | 0.035 | 0.12 | APP;JUN;DHX9 |
| Activation of caspases through apoptosome-mediated cleavage_Homo sapiens_R-HSA-111459 | 1/5 | 0.036 | 0.12 | CYCS |
| G2 Phase_Homo sapiens_R-HSA-68911 | 1/5 | 0.036 | 0.12 | E2F1 |
| Cytochrome c-mediated apoptotic response_Homo sapiens_R-HSA-111461 | 1/5 | 0.036 | 0.12 | CYCS |
| PTK6 Expression_Homo sapiens_R-HSA-8849473 | 1/5 | 0.036 | 0.12 | EPAS1 |
| Tachykinin receptors bind tachykinins_Homo sapiens_R-HSA-380095 | 1/5 | 0.036 | 0.12 | TACR1 |
| BMAL1:CLOCK,NPAS2 activates circadian gene expression_Homo sapiens_R-HSA-1368108 | 2/42 | 0.039 | 0.13 | NCOR1;HDAC3 |
| Non-integrin membrane-ECM interactions_Homo sapiens_R-HSA-3000171 | 2/42 | 0.039 | 0.13 | TGFB1;ITGB3 |
| TP53 Regulates Transcription of Cell Death Genes_Homo sapiens_R-HSA-5633008 | 2/43 | 0.040 | 0.13 | BAX;TP53 |
| Mitotic G2-G2/M phases_Homo sapiens_R-HSA-453274 | 4/175 | 0.041 | 0.13 | E2F1;TP53;YWHAG;AURKA |
| Gastrin-CREB signalling pathway via PKC and MAPK_Homo sapiens_R-HSA-881907 | 7/432 | 0.042 | 0.14 | APP;ERBB4;ITGB3;PRKCE;LPAR1;TACR1;JAK1 |
| SUMOylation of DNA replication proteins_Homo sapiens_R-HSA-4615885 | 2/44 | 0.042 | 0.14 | TOP1;AURKA |
| Platelet degranulation_Homo sapiens_R-HSA-114608 | 3/105 | 0.043 | 0.14 | APP;TGFB1;ITGB3 |
| p75NTR negatively regulates cell cycle via SC1_Homo sapiens_R-HSA-193670 | 1/6 | 0.044 | 0.14 | HDAC3 |
| RMTs methylate histone arginines_Homo sapiens_R-HSA-3214858 | 2/45 | 0.044 | 0.14 | PRMT6;SMARCC1 |
| Degradation of the extracellular matrix_Homo sapiens_R-HSA-1474228 | 3/106 | 0.044 | 0.14 | COL14A1;MMP2;MMP9 |
| SRP-dependent cotranslational protein targeting to membrane_Homo sapiens_R-HSA-1799339 | 3/107 | 0.045 | 0.14 | RPLP0;RPS3;RPL26L1 |
| NOTCH1 Intracellular Domain Regulates Transcription_Homo sapiens_R-HSA-2122947 | 2/47 | 0.047 | 0.15 | NCOR1;HDAC3 |
| Nucleotide-binding domain, leucine rich repeat containing receptor (NLR) signaling pathways_Homo sapiens_R-HSA-168643 | 2/47 | 0.047 | 0.15 | APP;BCL2L1 |
| Response to elevated platelet cytosolic Ca2+_Homo sapiens_R-HSA-76005 | 3/110 | 0.048 | 0.15 | APP;TGFB1;ITGB3 |
| EPH-ephrin mediated repulsion of cells_Homo sapiens_R-HSA-3928665 | 2/48 | 0.049 | 0.15 | MMP2;MMP9 |
| Selenoamino acid metabolism_Homo sapiens_R-HSA-2408522 | 3/111 | 0.049 | 0.15 | RPLP0;RPS3;RPL26L1 |

**Supplementary table S16.** Reactome pathway analysis based on significantly interacting genes with differentially expressed genes in children with HDLI (*P* < 0.05)

| **Term** | **Overlap** | **P-value** | **Adjusted P-value** | **Genes** |
| --- | --- | --- | --- | --- |
| Signaling by the B Cell Receptor (BCR)_Homo sapiens_R-HSA-983705 | 19/233 | 2.84E-11 | 1.96E-08 | CDKN1A;SRC;SHC1;SH3KBP1;INSR;GAB1;PTPN11;IRS2;FRS2;CBL;PSMC6;PSMC3;ERBB3;PSMC1;REL;AKT1;PLCG1;FGFR3;CARD11 |
| Downstream signal transduction_Homo sapiens_R-HSA-186763 | 21/341 | 4.75E-10 | 1.64E-07 | FGB;STAT5B;CDKN1A;SRC;SHC1;INSR;FN1;GAB1;PTPN11;IRS2;WDR83;FRS2;PSMC6;PSMC3;ERBB3;PSMC1;AKT1;PLCG1;FGFR3;CRK;BCAR1 |
| Diseases of signal transduction_Homo sapiens_R-HSA-5663202 | 19/288 | 1.06E-09 | 2.13E-07 | STAT5B;CDKN1A;HDAC2;SHC1;GAB1;PTPN11;IRS2;FRS2;CBL;TGFBR1;PSMC6;TTR;PSMC3;ERBB3;PSMC1;AKT1;EP300;PLCG1;FGFR3 |
| Signaling by SCF-KIT_Homo sapiens_R-HSA-1433557 | 20/325 | 1.27E-09 | 2.13E-07 | FGB;STAT5B;CDKN1A;SRC;SHC1;INSR;FN1;GAB1;PTPN11;IRS2;WDR83;FRS2;CBL;PSMC6;PSMC3;ERBB3;PSMC1;AKT1;FGFR3;SH2B2 |
| Signaling by PDGF_Homo sapiens_R-HSA-186797 | 21/364 | 1.54E-09 | 2.13E-07 | FGB;STAT5B;CDKN1A;SRC;SHC1;INSR;FN1;GAB1;PTPN11;IRS2;WDR83;FRS2;PSMC6;PSMC3;ERBB3;PSMC1;AKT1;PLCG1;FGFR3;CRK;BCAR1 |
| Signalling by NGF_Homo sapiens_R-HSA-166520 | 23/450 | 2.56E-09 | 2.95E-07 | FGB;CDKN1A;HDAC2;SRC;SHC1;MAGED1;ARHGEF17;INSR;ITSN1;FN1;GAB1;PTPN11;IRS2;WDR83;FRS2;PSMC6;PSMC3;ERBB3;PSMC1;AKT1;PLCG1;FGFR3;CRK |
| Signaling by EGFR_Homo sapiens_R-HSA-177929 | 20/355 | 5.77E-09 | 5.70E-07 | FGB;CDKN1A;SRC;SHC1;SH3KBP1;INSR;FN1;GAB1;PTPN11;IRS2;WDR83;FRS2;CBL;PSMC6;PSMC3;ERBB3;PSMC1;AKT1;PLCG1;FGFR3 |
| Downstream signaling events of B Cell Receptor (BCR)_Homo sapiens_R-HSA-1168372 | 15/192 | 6.61E-09 | 5.71E-07 | CDKN1A;SRC;INSR;GAB1;PTPN11;IRS2;FRS2;PSMC6;PSMC3;ERBB3;PSMC1;REL;AKT1;FGFR3;CARD11 |
| Signaling by ERBB4_Homo sapiens_R-HSA-1236394 | 19/330 | 9.92E-09 | 6.61E-07 | FGB;STAT5B;CDKN1A;SRC;SHC1;INSR;FN1;GAB1;PTPN11;IRS2;WDR83;FRS2;ESR1;PSMC6;PSMC3;ERBB3;PSMC1;AKT1;FGFR3 |
| Interleukin-3, 5 and GM-CSF signaling_Homo sapiens_R-HSA-512988 | 17/261 | 9.96E-09 | 6.61E-07 | FGB;STAT5B;SRC;SHC1;FN1;PTPN11;IRS2;WDR83;FRS2;CBL;PSMC6;PSMC3;ERBB3;INPP5D;PSMC1;FGFR3;CRK |
| Signaling by FGFR4_Homo sapiens_R-HSA-5654743 | 19/332 | 1.09E-08 | 6.61E-07 | FGB;CDKN1A;SRC;SHC1;INSR;FN1;GAB1;PTPN11;IRS2;WDR83;FRS2;CBL;PSMC6;PSMC3;ERBB3;PSMC1;AKT1;PLCG1;FGFR3 |
| Signaling by FGFR3_Homo sapiens_R-HSA-5654741 | 19/333 | 1.15E-08 | 6.61E-07 | FGB;CDKN1A;SRC;SHC1;INSR;FN1;GAB1;PTPN11;IRS2;WDR83;FRS2;CBL;PSMC6;PSMC3;ERBB3;PSMC1;AKT1;PLCG1;FGFR3 |
| Signaling by FGFR1_Homo sapiens_R-HSA-5654736 | 19/336 | 1.33E-08 | 7.05E-07 | FGB;CDKN1A;SRC;SHC1;INSR;FN1;GAB1;PTPN11;IRS2;WDR83;FRS2;CBL;PSMC6;PSMC3;ERBB3;PSMC1;AKT1;PLCG1;FGFR3 |
| Integrin alphaIIb beta3 signaling_Homo sapiens_R-HSA-354192 | 7/27 | 1.77E-08 | 8.75E-07 | FGB;SRC;SHC1;FN1;AKT1;CRK;BCAR1 |
| Disease_Homo sapiens_R-HSA-1643685 | 28/725 | 2.15E-08 | 9.92E-07 | CDKN1A;HDAC2;SHC1;IRS2;CBL;RPL6;TTR;ERBB3;AKT1;RCC1;EP300;BANF1;PLCG1;STAT5B;NPM1;XRCC6;GAB1;PTPN11;RPSA;FRS2;TGFBR1;CD4;PSMC6;PSMC3;PSMC1;RPL37A;FGFR3;RPL28 |
| Signaling by Interleukins_Homo sapiens_R-HSA-449147 | 20/392 | 3.04E-08 | 1.31E-06 | FGB;STAT5B;SRC;IL1R1;SHC1;FN1;PTPN11;IRS2;WDR83;FRS2;CBL;IL1RL2;CD4;PSMC6;PSMC3;ERBB3;INPP5D;PSMC1;FGFR3;CRK |
| Fc epsilon receptor (FCERI) signaling_Homo sapiens_R-HSA-2454202 | 20/395 | 3.44E-08 | 1.40E-06 | FGB;CDKN1A;SRC;SHC1;INSR;FN1;GAB1;UBE2D1;PTPN11;IRS2;WDR83;FRS2;PSMC6;PSMC3;ERBB3;PSMC1;AKT1;PLCG1;FGFR3;CARD11 |
| Insulin receptor signalling cascade_Homo sapiens_R-HSA-74751 | 17/287 | 4.04E-08 | 1.52E-06 | FGB;SRC;SHC1;INSR;FN1;GAB1;PTPN11;IRS2;WDR83;FRS2;PSMC6;PSMC3;ERBB3;RHEB;PSMC1;AKT1;FGFR3 |
| Signaling by FGFR2_Homo sapiens_R-HSA-5654738 | 19/361 | 4.18E-08 | 1.52E-06 | FGB;CDKN1A;SRC;SHC1;INSR;FN1;GAB1;PTPN11;IRS2;WDR83;FRS2;CBL;PSMC6;PSMC3;ERBB3;PSMC1;AKT1;PLCG1;FGFR3 |
| Immune System_Homo sapiens_R-HSA-168256 | 43/1547 | 5.12E-08 | 1.52E-06 | CDKN1A;SRC;SHC1;SH3KBP1;UBE2D1;IRS2;DEFB1;WDR83;CBL;IL1RL2;ERBB3;INPP5D;ABL1;AKT1;EP300;FLNB;PLCG1;MBL2;FGB;STAT5B;XRCC6;TRIM41;TNFSF14;IL1R1;UBE2E3;INSR;UBE2E1;FN1;GAB1;TRAF2;PTPN11;FRS2;UBE2G2;ATG12;CD4;PSMC6;PSMC3;PSMC1;UBE2U;REL;FGFR3;CRK;CARD11 |
| Signaling by FGFR_Homo sapiens_R-HSA-190236 | 19/366 | 5.19E-08 | 1.52E-06 | FGB;CDKN1A;SRC;SHC1;INSR;FN1;GAB1;PTPN11;IRS2;WDR83;FRS2;CBL;PSMC6;PSMC3;ERBB3;PSMC1;AKT1;PLCG1;FGFR3 |
| Downstream signaling of activated FGFR2_Homo sapiens_R-HSA-5654696 | 18/329 | 5.36E-08 | 1.52E-06 | FGB;CDKN1A;SRC;SHC1;INSR;FN1;GAB1;PTPN11;IRS2;WDR83;FRS2;PSMC6;PSMC3;ERBB3;PSMC1;AKT1;PLCG1;FGFR3 |
| Downstream signaling of activated FGFR3_Homo sapiens_R-HSA-5654708 | 18/329 | 5.36E-08 | 1.52E-06 | FGB;CDKN1A;SRC;SHC1;INSR;FN1;GAB1;PTPN11;IRS2;WDR83;FRS2;PSMC6;PSMC3;ERBB3;PSMC1;AKT1;PLCG1;FGFR3 |
| Downstream signaling of activated FGFR4_Homo sapiens_R-HSA-5654716 | 18/329 | 5.36E-08 | 1.52E-06 | FGB;CDKN1A;SRC;SHC1;INSR;FN1;GAB1;PTPN11;IRS2;WDR83;FRS2;PSMC6;PSMC3;ERBB3;PSMC1;AKT1;PLCG1;FGFR3 |
| Innate Immune System_Homo sapiens_R-HSA-168249 | 29/807 | 5.64E-08 | 1.52E-06 | CDKN1A;SRC;SHC1;UBE2D1;IRS2;DEFB1;WDR83;ERBB3;ABL1;AKT1;EP300;PLCG1;MBL2;FGB;XRCC6;INSR;FN1;GAB1;TRAF2;PTPN11;FRS2;ATG12;CD4;PSMC6;PSMC3;PSMC1;FGFR3;CRK;CARD11 |
| Cytokine Signaling in Immune system_Homo sapiens_R-HSA-1280215 | 25/620 | 5.70E-08 | 1.52E-06 | SRC;SHC1;IRS2;WDR83;CBL;IL1RL2;ERBB3;INPP5D;FLNB;PLCG1;FGB;STAT5B;TNFSF14;IL1R1;UBE2E1;FN1;TRAF2;PTPN11;FRS2;CD4;PSMC6;PSMC3;PSMC1;FGFR3;CRK |
| Downstream signaling of activated FGFR1_Homo sapiens_R-HSA-5654687 | 18/332 | 6.15E-08 | 1.57E-06 | FGB;CDKN1A;SRC;SHC1;INSR;FN1;GAB1;PTPN11;IRS2;WDR83;FRS2;PSMC6;PSMC3;ERBB3;PSMC1;AKT1;PLCG1;FGFR3 |
| NGF signalling via TRKA from the plasma membrane_Homo sapiens_R-HSA-187037 | 19/374 | 7.29E-08 | 1.80E-06 | FGB;CDKN1A;SRC;SHC1;INSR;FN1;GAB1;PTPN11;IRS2;WDR83;FRS2;PSMC6;PSMC3;ERBB3;PSMC1;AKT1;PLCG1;FGFR3;CRK |
| DAP12 signaling_Homo sapiens_R-HSA-2424491 | 18/344 | 1.05E-07 | 2.50E-06 | FGB;CDKN1A;SRC;SHC1;INSR;FN1;GAB1;PTPN11;IRS2;WDR83;FRS2;PSMC6;PSMC3;ERBB3;PSMC1;AKT1;PLCG1;FGFR3 |
| Signaling by Insulin receptor_Homo sapiens_R-HSA-74752 | 17/311 | 1.29E-07 | 2.97E-06 | FGB;SRC;SHC1;INSR;FN1;GAB1;PTPN11;IRS2;WDR83;FRS2;PSMC6;PSMC3;ERBB3;RHEB;PSMC1;AKT1;FGFR3 |
| Platelet Aggregation (Plug Formation)_Homo sapiens_R-HSA-76009 | 7/37 | 1.86E-07 | 4.15E-06 | FGB;SRC;SHC1;FN1;AKT1;CRK;BCAR1 |
| DAP12 interactions_Homo sapiens_R-HSA-2172127 | 18/359 | 1.98E-07 | 4.25E-06 | FGB;CDKN1A;SRC;SHC1;INSR;FN1;GAB1;PTPN11;IRS2;WDR83;FRS2;PSMC6;PSMC3;ERBB3;PSMC1;AKT1;PLCG1;FGFR3 |
| IRS-mediated signalling_Homo sapiens_R-HSA-112399 | 16/284 | 2.03E-07 | 4.25E-06 | FGB;SRC;SHC1;FN1;GAB1;PTPN11;IRS2;WDR83;FRS2;PSMC6;PSMC3;ERBB3;RHEB;PSMC1;AKT1;FGFR3 |
| Adaptive Immune System_Homo sapiens_R-HSA-1280218 | 27/762 | 2.23E-07 | 4.52E-06 | CDKN1A;SRC;SHC1;SH3KBP1;UBE2D1;IRS2;CBL;ERBB3;INPP5D;AKT1;PLCG1;TRIM41;UBE2E3;INSR;UBE2E1;GAB1;PTPN11;FRS2;UBE2G2;CD4;PSMC6;PSMC3;PSMC1;UBE2U;REL;FGFR3;CARD11 |
| IGF1R signaling cascade_Homo sapiens_R-HSA-2428924 | 16/288 | 2.45E-07 | 4.58E-06 | FGB;SRC;SHC1;FN1;GAB1;PTPN11;IRS2;WDR83;FRS2;PSMC6;PSMC3;ERBB3;RHEB;PSMC1;AKT1;FGFR3 |
| IRS-related events triggered by IGF1R_Homo sapiens_R-HSA-2428928 | 16/288 | 2.45E-07 | 4.58E-06 | FGB;SRC;SHC1;FN1;GAB1;PTPN11;IRS2;WDR83;FRS2;PSMC6;PSMC3;ERBB3;RHEB;PSMC1;AKT1;FGFR3 |
| Signaling by Type 1 Insulin-like Growth Factor 1 Receptor (IGF1R)_Homo sapiens_R-HSA-2404192 | 16/288 | 2.45E-07 | 4.58E-06 | FGB;SRC;SHC1;FN1;GAB1;PTPN11;IRS2;WDR83;FRS2;PSMC6;PSMC3;ERBB3;RHEB;PSMC1;AKT1;FGFR3 |
| Signaling by VEGF_Homo sapiens_R-HSA-194138 | 17/328 | 2.74E-07 | 4.99E-06 | FGB;FLT1;SRC;SHC1;FN1;IRS2;WDR83;FRS2;PSMC6;PSMC3;ERBB3;PSMC1;AKT1;PLCG1;FGFR3;CRK;BCAR1 |
| PI5P, PP2A and IER3 Regulate PI3K/AKT Signaling_Homo sapiens_R-HSA-6811558 | 9/83 | 4.72E-07 | 8.37E-06 | ERBB3;SRC;INSR;GAB1;AKT1;PTPN11;IRS2;FRS2;FGFR3 |
| p130Cas linkage to MAPK signaling for integrins_Homo sapiens_R-HSA-372708 | 5/15 | 5.26E-07 | 9.09E-06 | FGB;SRC;FN1;CRK;BCAR1 |
| Prolonged ERK activation events_Homo sapiens_R-HSA-169893 | 14/242 | 8.70E-07 | 1.47E-05 | FGB;SRC;SHC1;FN1;IRS2;WDR83;FRS2;PSMC6;PSMC3;ERBB3;PSMC1;PLCG1;FGFR3;CRK |
| mRNA Splicing_Homo sapiens_R-HSA-72172 | 11/144 | 8.96E-07 | 1.47E-05 | SF3B4;SF3A3;HSPA8;SF3B5;SF3A1;HNRNPU;U2AF1;SNRNP35;ELAVL2;RBM5;SUGP1 |
| Signaling by Leptin_Homo sapiens_R-HSA-2586552 | 14/243 | 9.14E-07 | 1.47E-05 | FGB;STAT5B;SRC;SHC1;FN1;PTPN11;IRS2;WDR83;FRS2;PSMC6;PSMC3;ERBB3;PSMC1;FGFR3 |
| Negative regulation of the PI3K/AKT network_Homo sapiens_R-HSA-199418 | 9/90 | 9.47E-07 | 1.49E-05 | ERBB3;SRC;INSR;GAB1;AKT1;PTPN11;IRS2;FRS2;FGFR3 |
| VEGFA-VEGFR2 Pathway_Homo sapiens_R-HSA-4420097 | 16/320 | 9.98E-07 | 1.53E-05 | FGB;SRC;SHC1;FN1;IRS2;WDR83;FRS2;PSMC6;PSMC3;ERBB3;PSMC1;AKT1;PLCG1;FGFR3;CRK;BCAR1 |
| Interleukin-2 signaling_Homo sapiens_R-HSA-451927 | 14/252 | 1.41E-06 | 1.94E-05 | FGB;STAT5B;SRC;SHC1;FN1;IRS2;WDR83;FRS2;PSMC6;PSMC3;ERBB3;INPP5D;PSMC1;FGFR3 |
| Signalling to ERKs_Homo sapiens_R-HSA-187687 | 14/253 | 1.48E-06 | 1.94E-05 | FGB;SRC;SHC1;FN1;IRS2;WDR83;FRS2;PSMC6;PSMC3;ERBB3;PSMC1;PLCG1;FGFR3;CRK |
| PI-3K cascade:FGFR1_Homo sapiens_R-HSA-5654689 | 10/122 | 1.49E-06 | 1.94E-05 | CDKN1A;ERBB3;SRC;INSR;GAB1;AKT1;PTPN11;IRS2;FRS2;FGFR3 |
| PI-3K cascade:FGFR2_Homo sapiens_R-HSA-5654695 | 10/122 | 1.49E-06 | 1.94E-05 | CDKN1A;ERBB3;SRC;INSR;GAB1;AKT1;PTPN11;IRS2;FRS2;FGFR3 |
| PI-3K cascade:FGFR3_Homo sapiens_R-HSA-5654710 | 10/122 | 1.49E-06 | 1.94E-05 | CDKN1A;ERBB3;SRC;INSR;GAB1;AKT1;PTPN11;IRS2;FRS2;FGFR3 |
| PI-3K cascade:FGFR4_Homo sapiens_R-HSA-5654720 | 10/122 | 1.49E-06 | 1.94E-05 | CDKN1A;ERBB3;SRC;INSR;GAB1;AKT1;PTPN11;IRS2;FRS2;FGFR3 |
| PI3K events in ERBB4 signaling_Homo sapiens_R-HSA-1250342 | 10/122 | 1.49E-06 | 1.94E-05 | CDKN1A;ERBB3;SRC;INSR;GAB1;AKT1;PTPN11;IRS2;FRS2;FGFR3 |
| PIP3 activates AKT signaling_Homo sapiens_R-HSA-1257604 | 10/122 | 1.49E-06 | 1.94E-05 | CDKN1A;ERBB3;SRC;INSR;GAB1;AKT1;PTPN11;IRS2;FRS2;FGFR3 |
| Hemostasis_Homo sapiens_R-HSA-109582 | 21/552 | 1.77E-06 | 2.26E-05 | FGB;HDAC2;SRC;SHC1;ITGA3;FN1;GNAI3;PTPN11;SERPINA4;SIN3A;INPP5D;GNAQ;ABL1;AKT1;EP300;PLCG1;TIMP1;A2M;CRK;SH2B2;BCAR1 |
| GAB1 signalosome_Homo sapiens_R-HSA-180292 | 10/125 | 1.86E-06 | 2.30E-05 | CDKN1A;ERBB3;SRC;INSR;GAB1;AKT1;PTPN11;IRS2;FRS2;FGFR3 |
| PI3K/AKT activation_Homo sapiens_R-HSA-198203 | 10/125 | 1.86E-06 | 2.30E-05 | CDKN1A;ERBB3;SRC;INSR;GAB1;AKT1;PTPN11;IRS2;FRS2;FGFR3 |
| Role of LAT2/NTAL/LAB on calcium mobilization_Homo sapiens_R-HSA-2730905 | 11/162 | 2.86E-06 | 3.46E-05 | CDKN1A;ERBB3;SRC;SHC1;INSR;GAB1;AKT1;PTPN11;IRS2;FRS2;FGFR3 |
| mRNA Splicing - Major Pathway_Homo sapiens_R-HSA-72163 | 10/134 | 3.50E-06 | 4.12E-05 | SF3B4;SF3A3;HSPA8;SF3B5;SF3A1;HNRNPU;U2AF1;ELAVL2;RBM5;SUGP1 |
| FRS-mediated FGFR1 signaling_Homo sapiens_R-HSA-5654693 | 13/236 | 3.70E-06 | 4.12E-05 | FGB;SRC;SHC1;FN1;PTPN11;IRS2;WDR83;FRS2;PSMC6;PSMC3;ERBB3;PSMC1;FGFR3 |
| FRS-mediated FGFR2 signaling_Homo sapiens_R-HSA-5654700 | 13/236 | 3.70E-06 | 4.12E-05 | FGB;SRC;SHC1;FN1;PTPN11;IRS2;WDR83;FRS2;PSMC6;PSMC3;ERBB3;PSMC1;FGFR3 |
| FRS-mediated FGFR3 signaling_Homo sapiens_R-HSA-5654706 | 13/236 | 3.70E-06 | 4.12E-05 | FGB;SRC;SHC1;FN1;PTPN11;IRS2;WDR83;FRS2;PSMC6;PSMC3;ERBB3;PSMC1;FGFR3 |
| FRS-mediated FGFR4 signaling_Homo sapiens_R-HSA-5654712 | 13/236 | 3.70E-06 | 4.12E-05 | FGB;SRC;SHC1;FN1;PTPN11;IRS2;WDR83;FRS2;PSMC6;PSMC3;ERBB3;PSMC1;FGFR3 |
| Gene Expression_Homo sapiens_R-HSA-74160 | 40/1631 | 3.88E-06 | 4.26E-05 | SF3B4;SF3B5;CDKN1A;HDAC2;ZNF274;ZNF324B;SUV39H1;HNRNPU;UBE2D1;U2AF1;NOC2L;ELAVL2;RPL6;TRIM28;PDCD11;SIN3A;AKT1;EP300;ZNF468;SNRNP35;SKIL;RBM5;SF3A3;HSPA8;BNIP3L;SF3A1;NPM1;H2AFX;RPSA;ESR1;SAP30;AR;PSMC6;PSMC3;MYBBP1A;RHEB;PSMC1;RPL37A;RPL28;SUGP1 |
| ARMS-mediated activation_Homo sapiens_R-HSA-170984 | 13/239 | 4.25E-06 | 4.59E-05 | FGB;SRC;SHC1;FN1;IRS2;WDR83;FRS2;PSMC6;PSMC3;ERBB3;PSMC1;FGFR3;CRK |
| Frs2-mediated activation_Homo sapiens_R-HSA-170968 | 13/240 | 4.45E-06 | 4.73E-05 | FGB;SRC;SHC1;FN1;IRS2;WDR83;FRS2;PSMC6;PSMC3;ERBB3;PSMC1;PLCG1;FGFR3 |
| MAPK1/MAPK3 signaling_Homo sapiens_R-HSA-5684996 | 13/241 | 4.65E-06 | 4.87E-05 | FGB;SRC;SHC1;FN1;PTPN11;IRS2;WDR83;FRS2;PSMC6;PSMC3;ERBB3;PSMC1;FGFR3 |
| Interleukin receptor SHC signaling_Homo sapiens_R-HSA-912526 | 13/245 | 5.56E-06 | 5.74E-05 | FGB;SRC;SHC1;FN1;IRS2;WDR83;FRS2;PSMC6;PSMC3;ERBB3;INPP5D;PSMC1;FGFR3 |
| VEGFR2 mediated cell proliferation_Homo sapiens_R-HSA-5218921 | 13/248 | 6.35E-06 | 6.45E-05 | FGB;SRC;SHC1;FN1;IRS2;WDR83;FRS2;PSMC6;PSMC3;ERBB3;PSMC1;PLCG1;FGFR3 |
| PI3K/AKT Signaling in Cancer_Homo sapiens_R-HSA-2219528 | 8/86 | 6.65E-06 | 6.66E-05 | CDKN1A;ERBB3;GAB1;AKT1;PTPN11;IRS2;FRS2;FGFR3 |
| PECAM1 interactions_Homo sapiens_R-HSA-210990 | 4/12 | 7.85E-06 | 7.66E-05 | SRC;INPP5D;PTPN11;PLCG1 |
| Platelet activation, signaling and aggregation_Homo sapiens_R-HSA-76002 | 13/253 | 7.87E-06 | 7.66E-05 | FGB;SRC;SHC1;FN1;GNAI3;PTPN11;SERPINA4;GNAQ;AKT1;TIMP1;A2M;CRK;BCAR1 |
| Signaling by ERBB2_Homo sapiens_R-HSA-1227986 | 6/45 | 1.21E-05 | 0.00012 | ERBB3;SRC;SHC1;GAB1;AKT1;PLCG1 |
| Developmental Biology_Homo sapiens_R-HSA-1266738 | 24/786 | 1.37E-05 | 0.00013 | FGB;HSPA8;TSC22D1;SRC;SHC1;H2AFX;ITSN1;FN1;AGAP2;PTPN11;IRS2;WDR83;FRS2;POU5F1;PSMC6;PSMC3;ERBB3;PSMC1;ABL1;AKT1;BNIP2;EP300;PLCG1;FGFR3 |
| Processing of Capped Intron-Containing Pre-mRNA_Homo sapiens_R-HSA-72203 | 11/193 | 1.52E-05 | 0.00014 | SF3B4;SF3A3;HSPA8;SF3B5;SF3A1;HNRNPU;U2AF1;SNRNP35;ELAVL2;RBM5;SUGP1 |
| Negative regulation of FGFR3 signaling_Homo sapiens_R-HSA-5654732 | 5/29 | 1.83E-05 | 0.00016 | SRC;PTPN11;FRS2;CBL;FGFR3 |
| GRB2 events in EGFR signaling_Homo sapiens_R-HSA-179812 | 12/235 | 1.88E-05 | 0.00016 | FGB;PSMC6;PSMC3;ERBB3;SRC;SHC1;PSMC1;FN1;IRS2;WDR83;FRS2;FGFR3 |
| RAF/MAP kinase cascade_Homo sapiens_R-HSA-5673001 | 12/235 | 1.88E-05 | 0.00016 | FGB;PSMC6;PSMC3;ERBB3;SRC;SHC1;PSMC1;FN1;IRS2;WDR83;FRS2;FGFR3 |
| SHC1 events in EGFR signaling_Homo sapiens_R-HSA-180336 | 12/235 | 1.88E-05 | 0.00016 | FGB;PSMC6;PSMC3;ERBB3;SRC;SHC1;PSMC1;FN1;IRS2;WDR83;FRS2;FGFR3 |
| SHC1 events in ERBB4 signaling_Homo sapiens_R-HSA-1250347 | 12/235 | 1.88E-05 | 0.00016 | FGB;PSMC6;PSMC3;ERBB3;SRC;SHC1;PSMC1;FN1;IRS2;WDR83;FRS2;FGFR3 |
| SOS-mediated signalling_Homo sapiens_R-HSA-112412 | 12/235 | 1.88E-05 | 0.00016 | FGB;PSMC6;PSMC3;ERBB3;SRC;SHC1;PSMC1;FN1;IRS2;WDR83;FRS2;FGFR3 |
| Constitutive Signaling by EGFRvIII_Homo sapiens_R-HSA-5637810 | 4/15 | 2.11E-05 | 0.00018 | SHC1;GAB1;PLCG1;CBL |
| Signaling by EGFRvIII in Cancer_Homo sapiens_R-HSA-5637812 | 4/15 | 2.11E-05 | 0.00018 | SHC1;GAB1;PLCG1;CBL |
| Signalling to p38 via RIT and RIN_Homo sapiens_R-HSA-187706 | 12/239 | 2.22E-05 | 0.00018 | FGB;PSMC6;PSMC3;ERBB3;SRC;SHC1;PSMC1;FN1;IRS2;WDR83;FRS2;FGFR3 |
| MAPK family signaling cascades_Homo sapiens_R-HSA-5683057 | 13/284 | 2.68E-05 | 0.00022 | FGB;SRC;SHC1;FN1;PTPN11;IRS2;WDR83;FRS2;PSMC6;PSMC3;ERBB3;PSMC1;FGFR3 |
| Signalling to RAS_Homo sapiens_R-HSA-167044 | 12/246 | 2.95E-05 | 0.00024 | FGB;PSMC6;PSMC3;ERBB3;SRC;SHC1;PSMC1;FN1;IRS2;WDR83;FRS2;FGFR3 |
| Axon guidance_Homo sapiens_R-HSA-422475 | 18/515 | 3.07E-05 | 0.00025 | FGB;HSPA8;SRC;SHC1;ITSN1;FN1;AGAP2;PTPN11;IRS2;WDR83;FRS2;PSMC6;PSMC3;ERBB3;PSMC1;ABL1;PLCG1;FGFR3 |
| FCERI mediated MAPK activation_Homo sapiens_R-HSA-2871796 | 13/289 | 3.21E-05 | 0.00026 | FGB;SRC;SHC1;FN1;IRS2;WDR83;FRS2;PSMC6;PSMC3;ERBB3;PSMC1;PLCG1;FGFR3 |
| PI3K Cascade_Homo sapiens_R-HSA-109704 | 7/79 | 3.48E-05 | 0.00027 | RHEB;GAB1;AKT1;PTPN11;IRS2;FRS2;FGFR3 |
| Constitutive Signaling by Ligand-Responsive EGFR Cancer Variants_Homo sapiens_R-HSA-1236382 | 4/19 | 5.77E-05 | 0.00044 | SHC1;GAB1;PLCG1;CBL |
| Signaling by EGFR in Cancer_Homo sapiens_R-HSA-1643713 | 4/19 | 5.77E-05 | 0.00044 | SHC1;GAB1;PLCG1;CBL |
| Signaling by Ligand-Responsive EGFR Variants in Cancer_Homo sapiens_R-HSA-5637815 | 4/19 | 5.77E-05 | 0.00044 | SHC1;GAB1;PLCG1;CBL |
| NCAM signaling for neurite out-growth_Homo sapiens_R-HSA-375165 | 12/266 | 6.29E-05 | 0.00047 | FGB;PSMC6;PSMC3;ERBB3;SRC;SHC1;PSMC1;FN1;IRS2;WDR83;FRS2;FGFR3 |
| TCR signaling_Homo sapiens_R-HSA-202403 | 8/118 | 6.69E-05 | 0.00050 | CD4;PSMC6;PSMC3;INPP5D;PSMC1;UBE2D1;PLCG1;CARD11 |
| Constitutive Signaling by Aberrant PI3K in Cancer_Homo sapiens_R-HSA-2219530 | 6/61 | 7.10E-05 | 0.00052 | ERBB3;GAB1;PTPN11;IRS2;FRS2;FGFR3 |
| Signal Transduction_Homo sapiens_R-HSA-162582 | 49/2465 | 8.11E-05 | 0.00059 | CDKN1A;FLT1;SH3KBP1;ITSN1;UBE2D1;IRS2;WDR83;RSPO2;AKT1;EP300;FGB;ARHGEF17;H2AFX;GAB1;TRAF2;FRS2;TRAF1;UCHL5;TGFBR1;AR;CRK;HDAC2;SRC;SHC1;MAGED1;GNAI3;LRP2;CBL;TTR;ERBB3;ABL1;PLCG1;A2M;SKIL;SH2B2;STAT5B;INSR;FN1;PTPN11;ESR1;GNB2L1;ARHGAP32;PSMC6;PSMC3;RHEB;PSMC1;GNAQ;FGFR3;BCAR1 |
| Cellular responses to stress_Homo sapiens_R-HSA-2262752 | 14/367 | 9.55E-05 | 0.00069 | HSPA8;CDKN1A;PHC2;CBX6;H2AFX;TINF2;UBE2E1;UBE2D1;TERF1;PHC3;ATG12;RHEB;BAG1;EP300 |
| Cellular Senescence_Homo sapiens_R-HSA-2559583 | 9/161 | 0.00011 | 0.00074 | CDKN1A;PHC2;CBX6;H2AFX;TINF2;UBE2E1;UBE2D1;TERF1;PHC3 |
| Signaling by FGFR3 in disease_Homo sapiens_R-HSA-5655332 | 4/22 | 0.00011 | 0.00074 | GAB1;FRS2;PLCG1;FGFR3 |
| Signaling by FGFR3 point mutants in cancer_Homo sapiens_R-HSA-8853338 | 4/22 | 0.00011 | 0.00074 | GAB1;FRS2;PLCG1;FGFR3 |
| Downstream TCR signaling_Homo sapiens_R-HSA-202424 | 7/99 | 0.00015 | 0.0010 | CD4;PSMC6;PSMC3;INPP5D;PSMC1;UBE2D1;CARD11 |
| Cell surface interactions at the vascular wall_Homo sapiens_R-HSA-202733 | 7/101 | 0.00017 | 0.0011 | SRC;SHC1;ITGA3;INPP5D;FN1;PTPN11;PLCG1 |
| Signaling by FGFR3 fusions in cancer_Homo sapiens_R-HSA-8853334 | 3/10 | 0.00017 | 0.0011 | GAB1;FRS2;FGFR3 |
| Signal attenuation_Homo sapiens_R-HSA-74749 | 3/10 | 0.00017 | 0.0011 | SHC1;INSR;IRS2 |
| Signaling by FGFR4 in disease_Homo sapiens_R-HSA-5655291 | 3/11 | 0.00023 | 0.0015 | GAB1;FRS2;PLCG1 |
| Molecules associated with elastic fibres_Homo sapiens_R-HSA-2129379 | 4/30 | 0.00037 | 0.0024 | EFEMP2;MFAP2;FBLN1;FBLN2 |
| Epigenetic regulation of gene expression_Homo sapiens_R-HSA-212165 | 7/115 | 0.00037 | 0.0024 | HDAC2;MYBBP1A;SIN3A;SUV39H1;H2AFX;EP300;SAP30 |
| Negative regulation of FGFR4 signaling_Homo sapiens_R-HSA-5654733 | 4/31 | 0.00042 | 0.0027 | SRC;PTPN11;FRS2;CBL |
| Negative regulation of FGFR1 signaling_Homo sapiens_R-HSA-5654726 | 4/32 | 0.00048 | 0.0030 | SRC;PTPN11;FRS2;CBL |
| Early Phase of HIV Life Cycle_Homo sapiens_R-HSA-162594 | 3/14 | 0.00050 | 0.0032 | CD4;XRCC6;BANF1 |
| C-type lectin receptors (CLRs)_Homo sapiens_R-HSA-5621481 | 7/123 | 0.00056 | 0.0035 | PSMC6;PSMC3;SRC;PSMC1;UBE2D1;EP300;CARD11 |
| Negative regulation of FGFR2 signaling_Homo sapiens_R-HSA-5654727 | 4/34 | 0.00060 | 0.0038 | SRC;PTPN11;FRS2;CBL |
| GRB2:SOS provides linkage to MAPK signaling for Integrins_Homo sapiens_R-HSA-354194 | 3/15 | 0.00062 | 0.0038 | FGB;SRC;FN1 |
| Autodegradation of Cdh1 by Cdh1:APC/C_Homo sapiens_R-HSA-174084 | 5/62 | 0.00073 | 0.0044 | PSMC6;PSMC3;PSMC1;UBE2E1;UBE2D1 |
| Infectious disease_Homo sapiens_R-HSA-5663205 | 12/348 | 0.00074 | 0.0044 | CD4;NPM1;XRCC6;PSMC6;PSMC3;PSMC1;RPL37A;RCC1;RPSA;BANF1;RPL28;RPL6 |
| Host Interactions of HIV factors_Homo sapiens_R-HSA-162909 | 7/129 | 0.00074 | 0.0044 | CD4;NPM1;PSMC6;PSMC3;PSMC1;RCC1;BANF1 |
| Spry regulation of FGF signaling_Homo sapiens_R-HSA-1295596 | 3/16 | 0.00075 | 0.0044 | SRC;PTPN11;CBL |
| Regulation of KIT signaling_Homo sapiens_R-HSA-1433559 | 3/16 | 0.00075 | 0.0044 | SRC;CBL;SH2B2 |
| Signaling by FGFR in disease_Homo sapiens_R-HSA-1226099 | 5/63 | 0.00079 | 0.0046 | STAT5B;GAB1;FRS2;PLCG1;FGFR3 |
| MAP2K and MAPK activation_Homo sapiens_R-HSA-5674135 | 4/38 | 0.00093 | 0.0053 | FGB;SRC;FN1;WDR83 |
| Signaling by FGFR1 in disease_Homo sapiens_R-HSA-5655302 | 4/38 | 0.00093 | 0.0053 | STAT5B;GAB1;FRS2;PLCG1 |
| Activation of NF-kappaB in B cells_Homo sapiens_R-HSA-1169091 | 5/66 | 0.00097 | 0.0055 | PSMC6;PSMC3;PSMC1;REL;CARD11 |
| APC/C:Cdc20 mediated degradation of Securin_Homo sapiens_R-HSA-174154 | 5/66 | 0.00097 | 0.0055 | PSMC6;PSMC3;PSMC1;UBE2E1;UBE2D1 |
| CLEC7A (Dectin-1) signaling_Homo sapiens_R-HSA-5607764 | 6/99 | 0.0010 | 0.0056 | PSMC6;PSMC3;SRC;PSMC1;UBE2D1;CARD11 |
| Signaling by PTK6_Homo sapiens_R-HSA-8848021 | 5/67 | 0.0010 | 0.0058 | ERBB3;AKT1;CBL;CRK;BCAR1 |
| Elastic fibre formation_Homo sapiens_R-HSA-1566948 | 4/41 | 0.0012 | 0.0068 | EFEMP2;MFAP2;FBLN1;FBLN2 |
| IRS activation_Homo sapiens_R-HSA-74713 | 2/5 | 0.0013 | 0.0071 | INSR;IRS2 |
| APC/C:Cdh1 mediated degradation of Cdc20 and other APC/C:Cdh1 targeted proteins in late mitosis/early G1_Homo sapiens_R-HSA-174178 | 5/71 | 0.0014 | 0.0073 | PSMC6;PSMC3;PSMC1;UBE2E1;UBE2D1 |
| Cdc20:Phospho-APC/C mediated degradation of Cyclin A_Homo sapiens_R-HSA-174184 | 5/71 | 0.0014 | 0.0073 | PSMC6;PSMC3;PSMC1;UBE2E1;UBE2D1 |
| Netrin-1 signaling_Homo sapiens_R-HSA-373752 | 4/42 | 0.0014 | 0.0073 | SRC;AGAP2;PTPN11;PLCG1 |
| APC:Cdc20 mediated degradation of cell cycle proteins prior to satisfation of the cell cycle checkpoint_Homo sapiens_R-HSA-179419 | 5/72 | 0.0014 | 0.0077 | PSMC6;PSMC3;PSMC1;UBE2E1;UBE2D1 |
| Signaling by TGF-beta Receptor Complex_Homo sapiens_R-HSA-170834 | 5/73 | 0.0015 | 0.0081 | UBE2D1;CBL;SKIL;UCHL5;TGFBR1 |
| Gastrin-CREB signalling pathway via PKC and MAPK_Homo sapiens_R-HSA-881907 | 13/432 | 0.0016 | 0.0081 | FGB;SRC;SHC1;FN1;IRS2;WDR83;FRS2;PSMC6;PSMC3;ERBB3;PSMC1;GNAQ;FGFR3 |
| APC/C:Cdc20 mediated degradation of mitotic proteins_Homo sapiens_R-HSA-176409 | 5/74 | 0.0016 | 0.0085 | PSMC6;PSMC3;PSMC1;UBE2E1;UBE2D1 |
| Extracellular matrix organization_Homo sapiens_R-HSA-1474244 | 10/283 | 0.0017 | 0.0087 | FGB;EFEMP2;TTR;ITGA3;MFAP2;FN1;FBLN1;TIMP1;A2M;FBLN2 |
| Activation of APC/C and APC/C:Cdc20 mediated degradation of mitotic proteins_Homo sapiens_R-HSA-176814 | 5/75 | 0.0017 | 0.0088 | PSMC6;PSMC3;PSMC1;UBE2E1;UBE2D1 |
| Negative epigenetic regulation of rRNA expression_Homo sapiens_R-HSA-5250941 | 5/76 | 0.0018 | 0.0093 | HDAC2;SIN3A;SUV39H1;H2AFX;SAP30 |
| CTLA4 inhibitory signaling_Homo sapiens_R-HSA-389513 | 3/22 | 0.0020 | 0.0099 | SRC;AKT1;PTPN11 |
| Antigen activates B Cell Receptor (BCR) leading to generation of second messengers_Homo sapiens_R-HSA-983695 | 4/47 | 0.0021 | 0.010 | SHC1;SH3KBP1;PLCG1;CBL |
| Regulation of APC/C activators between G1/S and early anaphase_Homo sapiens_R-HSA-176408 | 5/79 | 0.0022 | 0.011 | PSMC6;PSMC3;PSMC1;UBE2E1;UBE2D1 |
| SHC-mediated cascade:FGFR4_Homo sapiens_R-HSA-5654719 | 3/23 | 0.0022 | 0.011 | SHC1;PTPN11;FRS2 |
| Regulation of TP53 Activity through Acetylation_Homo sapiens_R-HSA-6804758 | 3/23 | 0.0022 | 0.011 | HDAC2;AKT1;EP300 |
| 2-LTR circle formation_Homo sapiens_R-HSA-164843 | 2/7 | 0.0027 | 0.013 | XRCC6;BANF1 |
| Vpu mediated degradation of CD4_Homo sapiens_R-HSA-180534 | 4/51 | 0.0028 | 0.013 | CD4;PSMC6;PSMC3;PSMC1 |
| Apoptosis_Homo sapiens_R-HSA-109581 | 7/163 | 0.0028 | 0.014 | PSMC6;PSMC3;MAGED1;PSMC1;AKT1;TRAF2;VIM |
| APC/C-mediated degradation of cell cycle proteins_Homo sapiens_R-HSA-174143 | 5/85 | 0.0030 | 0.014 | PSMC6;PSMC3;PSMC1;UBE2E1;UBE2D1 |
| Regulation of mitotic cell cycle_Homo sapiens_R-HSA-453276 | 5/85 | 0.0030 | 0.014 | PSMC6;PSMC3;PSMC1;UBE2E1;UBE2D1 |
| Programmed Cell Death_Homo sapiens_R-HSA-5357801 | 7/166 | 0.0031 | 0.015 | PSMC6;PSMC3;MAGED1;PSMC1;AKT1;TRAF2;VIM |
| Regulation of mRNA stability by proteins that bind AU-rich elements_Homo sapiens_R-HSA-450531 | 5/86 | 0.0032 | 0.015 | HSPA8;PSMC6;PSMC3;PSMC1;AKT1 |
| SHC-mediated cascade:FGFR2_Homo sapiens_R-HSA-5654699 | 3/26 | 0.0032 | 0.015 | SHC1;PTPN11;FRS2 |
| TNFR1-induced NFkappaB signaling pathway_Homo sapiens_R-HSA-5357956 | 3/26 | 0.0032 | 0.015 | TRAF2;TRAF1;GNB2L1 |
| Antigen processing: Ubiquitination & Proteasome degradation_Homo sapiens_R-HSA-983168 | 9/260 | 0.0033 | 0.015 | PSMC6;TRIM41;PSMC3;UBE2E3;PSMC1;UBE2U;UBE2E1;UBE2D1;UBE2G2 |
| AUF1 (hnRNP D0) binds and destabilizes mRNA_Homo sapiens_R-HSA-450408 | 4/54 | 0.0034 | 0.016 | HSPA8;PSMC6;PSMC3;PSMC1 |
| M Phase_Homo sapiens_R-HSA-68886 | 9/268 | 0.0040 | 0.018 | GOLGA2;PSMC6;PSMC3;PSMC1;RAB1B;H2AFX;UBE2E1;UBE2D1;BANF1 |
| HIV Infection_Homo sapiens_R-HSA-162906 | 8/222 | 0.0043 | 0.019 | CD4;NPM1;XRCC6;PSMC6;PSMC3;PSMC1;RCC1;BANF1 |
| PTK6 Regulates RTKs and Their Effectors AKT1 and DOK1_Homo sapiens_R-HSA-8849469 | 2/9 | 0.0045 | 0.020 | AKT1;CBL |
| Integration of provirus_Homo sapiens_R-HSA-162592 | 2/9 | 0.0045 | 0.020 | XRCC6;BANF1 |
| The role of GTSE1 in G2/M progression after G2 checkpoint_Homo sapiens_R-HSA-8852276 | 4/59 | 0.0047 | 0.021 | CDKN1A;PSMC6;PSMC3;PSMC1 |
| SCF(Skp2)-mediated degradation of p27/p21_Homo sapiens_R-HSA-187577 | 4/59 | 0.0047 | 0.021 | CDKN1A;PSMC6;PSMC3;PSMC1 |
| DNA Damage/Telomere Stress Induced Senescence_Homo sapiens_R-HSA-2559586 | 4/59 | 0.0047 | 0.021 | CDKN1A;H2AFX;TINF2;TERF1 |
| TNFR2 non-canonical NF-kB pathway_Homo sapiens_R-HSA-5668541 | 5/95 | 0.0048 | 0.021 | PSMC6;PSMC3;TNFSF14;PSMC1;TRAF2 |
| Cell Cycle Checkpoints_Homo sapiens_R-HSA-69620 | 7/182 | 0.0052 | 0.022 | CDKN1A;PSMC6;PSMC3;PSMC1;H2AFX;UBE2E1;UBE2D1 |
| Regulation of TNFR1 signaling_Homo sapiens_R-HSA-5357905 | 3/31 | 0.0053 | 0.023 | TRAF2;TRAF1;GNB2L1 |
| Packaging Of Telomere Ends_Homo sapiens_R-HSA-171306 | 3/31 | 0.0053 | 0.023 | H2AFX;TINF2;TERF1 |
| Netrin mediated repulsion signals_Homo sapiens_R-HSA-418886 | 2/10 | 0.0056 | 0.023 | SRC;PTPN11 |
| eNOS activation_Homo sapiens_R-HSA-203615 | 2/10 | 0.0056 | 0.023 | DDAH2;AKT1 |
| POU5F1 (OCT4), SOX2, NANOG repress genes related to differentiation_Homo sapiens_R-HSA-2892245 | 2/10 | 0.0056 | 0.023 | TSC22D1;POU5F1 |
| TGF-beta receptor signaling activates SMADs_Homo sapiens_R-HSA-2173789 | 3/32 | 0.0058 | 0.024 | CBL;UCHL5;TGFBR1 |
| Signal amplification_Homo sapiens_R-HSA-392518 | 3/32 | 0.0058 | 0.024 | SRC;GNAQ;GNAI3 |
| p53-Dependent G1 DNA Damage Response_Homo sapiens_R-HSA-69563 | 4/63 | 0.0060 | 0.024 | CDKN1A;PSMC6;PSMC3;PSMC1 |
| p53-Dependent G1/S DNA damage checkpoint_Homo sapiens_R-HSA-69580 | 4/63 | 0.0060 | 0.024 | CDKN1A;PSMC6;PSMC3;PSMC1 |
| Cell Cycle_Homo sapiens_R-HSA-1640170 | 14/566 | 0.0061 | 0.025 | CDKN1A;NPM1;RAB1B;H2AFX;TINF2;UBE2E1;UBE2D1;TERF1;GOLGA2;PSMC6;PSMC3;PSMC1;EP300;BANF1 |
| G1/S DNA Damage Checkpoints_Homo sapiens_R-HSA-69615 | 4/65 | 0.0067 | 0.027 | CDKN1A;PSMC6;PSMC3;PSMC1 |
| Interleukin-6 signaling_Homo sapiens_R-HSA-1059683 | 2/11 | 0.0068 | 0.027 | PTPN11;CBL |
| Platelet degranulation_Homo sapiens_R-HSA-114608 | 5/105 | 0.0073 | 0.029 | FGB;FN1;TIMP1;A2M;SERPINA4 |
| Cyclin E associated events during G1/S transition_Homo sapiens_R-HSA-69202 | 4/68 | 0.0078 | 0.031 | CDKN1A;PSMC6;PSMC3;PSMC1 |
| COPII (Coat Protein 2) Mediated Vesicle Transport_Homo sapiens_R-HSA-204005 | 4/68 | 0.0078 | 0.031 | GOLGA2;LMAN2L;TFG;RAB1B |
| Cyclin A:Cdk2-associated events at S phase entry_Homo sapiens_R-HSA-69656 | 4/69 | 0.0082 | 0.032 | CDKN1A;PSMC6;PSMC3;PSMC1 |
| TCF dependent signaling in response to WNT_Homo sapiens_R-HSA-201681 | 7/199 | 0.0083 | 0.032 | PSMC6;PSMC3;PSMC1;H2AFX;RSPO2;AKT1;EP300 |
| Orc1 removal from chromatin_Homo sapiens_R-HSA-68949 | 4/70 | 0.0086 | 0.033 | CDKN1A;PSMC6;PSMC3;PSMC1 |
| Switching of origins to a post-replicative state_Homo sapiens_R-HSA-69052 | 4/70 | 0.0086 | 0.033 | CDKN1A;PSMC6;PSMC3;PSMC1 |
| Response to elevated platelet cytosolic Ca2+_Homo sapiens_R-HSA-76005 | 5/110 | 0.0089 | 0.034 | FGB;FN1;TIMP1;A2M;SERPINA4 |
| Class I MHC mediated antigen processing & presentation_Homo sapiens_R-HSA-983169 | 9/305 | 0.0090 | 0.034 | PSMC6;TRIM41;PSMC3;UBE2E3;PSMC1;UBE2U;UBE2E1;UBE2D1;UBE2G2 |
| FCERI mediated NF-kB activation_Homo sapiens_R-HSA-2871837 | 5/111 | 0.0092 | 0.035 | PSMC6;PSMC3;PSMC1;UBE2D1;CARD11 |
| Downregulation of ERBB2:ERBB3 signaling_Homo sapiens_R-HSA-1358803 | 2/13 | 0.0094 | 0.035 | ERBB3;AKT1 |
| AKT phosphorylates targets in the cytosol_Homo sapiens_R-HSA-198323 | 2/13 | 0.0094 | 0.035 | CDKN1A;AKT1 |
| Signal regulatory protein (SIRP) family interactions_Homo sapiens_R-HSA-391160 | 2/13 | 0.0094 | 0.035 | SRC;PTPN11 |
| Removal of licensing factors from origins_Homo sapiens_R-HSA-69300 | 4/72 | 0.0095 | 0.035 | CDKN1A;PSMC6;PSMC3;PSMC1 |
| Costimulation by the CD28 family_Homo sapiens_R-HSA-388841 | 4/72 | 0.0095 | 0.035 | CD4;SRC;AKT1;PTPN11 |
| NoRC negatively regulates rRNA expression_Homo sapiens_R-HSA-427413 | 4/73 | 0.010 | 0.036 | HDAC2;SIN3A;H2AFX;SAP30 |
| Positive epigenetic regulation of rRNA expression_Homo sapiens_R-HSA-5250913 | 4/73 | 0.010 | 0.036 | HDAC2;MYBBP1A;H2AFX;EP300 |
| Nuclear signaling by ERBB4_Homo sapiens_R-HSA-1251985 | 3/39 | 0.010 | 0.037 | STAT5B;PTPN11;ESR1 |
| PKMTs methylate histone lysines_Homo sapiens_R-HSA-3214841 | 3/40 | 0.011 | 0.039 | ATF7IP;SETDB1;SUV39H1 |
| Golgi Cisternae Pericentriolar Stack Reorganization_Homo sapiens_R-HSA-162658 | 2/14 | 0.011 | 0.039 | GOLGA2;RAB1B |
| Regulation of DNA replication_Homo sapiens_R-HSA-69304 | 4/75 | 0.011 | 0.039 | CDKN1A;PSMC6;PSMC3;PSMC1 |
| TNF signaling_Homo sapiens_R-HSA-75893 | 3/41 | 0.012 | 0.041 | TRAF2;TRAF1;GNB2L1 |
| Senescence-Associated Secretory Phenotype (SASP)_Homo sapiens_R-HSA-2559582 | 4/77 | 0.012 | 0.042 | CDKN1A;H2AFX;UBE2E1;UBE2D1 |
| Prolactin receptor signaling_Homo sapiens_R-HSA-1170546 | 2/15 | 0.013 | 0.044 | STAT5B;PTPN11 |
| RIG-I/MDA5 mediated induction of IFN-alpha/beta pathways_Homo sapiens_R-HSA-168928 | 4/79 | 0.013 | 0.046 | UBE2D1;EP300;TRAF2;ATG12 |
| Signaling by FGFR2 in disease_Homo sapiens_R-HSA-5655253 | 3/43 | 0.013 | 0.046 | GAB1;FRS2;PLCG1 |
| Iron uptake and transport_Homo sapiens_R-HSA-917937 | 3/43 | 0.013 | 0.046 | FTH1;LCN2;STEAP2 |
| PI3K events in ERBB2 signaling_Homo sapiens_R-HSA-1963642 | 2/16 | 0.014 | 0.049 | ERBB3;GAB1 |
| NRAGE signals death through JNK_Homo sapiens_R-HSA-193648 | 3/45 | 0.015 | 0.051 | MAGED1;ARHGEF17;ITSN1 |
| Mitotic Anaphase_Homo sapiens_R-HSA-68882 | 6/173 | 0.015 | 0.051 | PSMC6;PSMC3;PSMC1;UBE2E1;UBE2D1;BANF1 |
| p75 NTR receptor-mediated signalling_Homo sapiens_R-HSA-193704 | 4/83 | 0.015 | 0.052 | HDAC2;MAGED1;ARHGEF17;ITSN1 |
| Mitotic Metaphase and Anaphase_Homo sapiens_R-HSA-2555396 | 6/174 | 0.016 | 0.052 | PSMC6;PSMC3;PSMC1;UBE2E1;UBE2D1;BANF1 |
| Chromatin modifying enzymes_Homo sapiens_R-HSA-3247509 | 7/226 | 0.016 | 0.053 | ATF7IP;HDAC2;SETDB1;SUV39H1;H2AFX;EP300;SAP30 |
| Chromatin organization_Homo sapiens_R-HSA-4839726 | 7/226 | 0.016 | 0.053 | ATF7IP;HDAC2;SETDB1;SUV39H1;H2AFX;EP300;SAP30 |
| TNF receptor superfamily (TNFSF) members mediating non-canonical NF-kB pathway_Homo sapiens_R-HSA-5676594 | 2/17 | 0.016 | 0.053 | TNFSF14;TRAF2 |
| Peptide chain elongation_Homo sapiens_R-HSA-156902 | 4/84 | 0.016 | 0.053 | RPL37A;RPSA;RPL28;RPL6 |
| Viral mRNA Translation_Homo sapiens_R-HSA-192823 | 4/84 | 0.016 | 0.053 | RPL37A;RPSA;RPL28;RPL6 |
| Chromosome Maintenance_Homo sapiens_R-HSA-73886 | 4/86 | 0.017 | 0.057 | NPM1;H2AFX;TINF2;TERF1 |
| Death Receptor Signalling_Homo sapiens_R-HSA-73887 | 3/48 | 0.018 | 0.057 | TRAF2;TRAF1;GNB2L1 |
| TP53 Regulates Transcription of Cell Cycle Genes_Homo sapiens_R-HSA-6791312 | 3/48 | 0.018 | 0.057 | CDKN1A;NPM1;EP300 |
| SHC-mediated cascade:FGFR3_Homo sapiens_R-HSA-5654704 | 2/18 | 0.018 | 0.057 | SHC1;FGFR3 |
| Tie2 Signaling_Homo sapiens_R-HSA-210993 | 2/18 | 0.018 | 0.057 | SHC1;PTPN11 |
| Regulation of signaling by CBL_Homo sapiens_R-HSA-912631 | 2/18 | 0.018 | 0.057 | CBL;CRK |
| SHC1 events in ERBB2 signaling_Homo sapiens_R-HSA-1250196 | 2/18 | 0.018 | 0.057 | ERBB3;SHC1 |
| Eukaryotic Translation Termination_Homo sapiens_R-HSA-72764 | 4/87 | 0.018 | 0.057 | RPL37A;RPSA;RPL28;RPL6 |
| Selenocysteine synthesis_Homo sapiens_R-HSA-2408557 | 4/87 | 0.018 | 0.057 | RPL37A;RPSA;RPL28;RPL6 |
| Cell Cycle, Mitotic_Homo sapiens_R-HSA-69278 | 11/462 | 0.018 | 0.058 | GOLGA2;CDKN1A;PSMC6;PSMC3;PSMC1;RAB1B;H2AFX;UBE2E1;UBE2D1;EP300;BANF1 |
| Regulation of activated PAK-2p34 by proteasome mediated degradation_Homo sapiens_R-HSA-211733 | 3/49 | 0.019 | 0.058 | PSMC6;PSMC3;PSMC1 |
| Cross-presentation of soluble exogenous antigens (endosomes)_Homo sapiens_R-HSA-1236978 | 3/49 | 0.019 | 0.058 | PSMC6;PSMC3;PSMC1 |
| Eukaryotic Translation Elongation_Homo sapiens_R-HSA-156842 | 4/89 | 0.019 | 0.059 | RPL37A;RPSA;RPL28;RPL6 |
| Nonsense Mediated Decay (NMD) independent of the Exon Junction Complex (EJC)_Homo sapiens_R-HSA-975956 | 4/89 | 0.019 | 0.059 | RPL37A;RPSA;RPL28;RPL6 |
| Regulation of Apoptosis_Homo sapiens_R-HSA-169911 | 3/50 | 0.020 | 0.059 | PSMC6;PSMC3;PSMC1 |
| CDK-mediated phosphorylation and removal of Cdc6_Homo sapiens_R-HSA-69017 | 3/50 | 0.020 | 0.059 | PSMC6;PSMC3;PSMC1 |
| Ubiquitin-dependent degradation of Cyclin D1_Homo sapiens_R-HSA-69229 | 3/50 | 0.020 | 0.059 | PSMC6;PSMC3;PSMC1 |
| Ubiquitin-dependent degradation of Cyclin D_Homo sapiens_R-HSA-75815 | 3/50 | 0.020 | 0.059 | PSMC6;PSMC3;PSMC1 |
| Regulation of ornithine decarboxylase (ODC)_Homo sapiens_R-HSA-350562 | 3/50 | 0.020 | 0.059 | PSMC6;PSMC3;PSMC1 |
| Phosphorylation of the APC/C_Homo sapiens_R-HSA-176412 | 2/19 | 0.020 | 0.059 | UBE2E1;UBE2D1 |
| Conversion from APC/C:Cdc20 to APC/C:Cdh1 in late anaphase_Homo sapiens_R-HSA-176407 | 2/19 | 0.020 | 0.059 | UBE2E1;UBE2D1 |
| HDL-mediated lipid transport_Homo sapiens_R-HSA-194223 | 2/19 | 0.020 | 0.059 | SCARB1;A2M |
| Autodegradation of the E3 ubiquitin ligase COP1_Homo sapiens_R-HSA-349425 | 3/51 | 0.021 | 0.062 | PSMC6;PSMC3;PSMC1 |
| Oxidative Stress Induced Senescence_Homo sapiens_R-HSA-2559580 | 4/91 | 0.021 | 0.062 | PHC2;CBX6;H2AFX;PHC3 |
| Inactivation of APC/C via direct inhibition of the APC/C complex_Homo sapiens_R-HSA-141430 | 2/20 | 0.022 | 0.062 | UBE2E1;UBE2D1 |
| Inhibition of the proteolytic activity of APC/C required for the onset of anaphase by mitotic spindle checkpoint components_Homo sapiens_R-HSA-141405 | 2/20 | 0.022 | 0.062 | UBE2E1;UBE2D1 |
| eNOS activation and regulation_Homo sapiens_R-HSA-203765 | 2/20 | 0.022 | 0.062 | DDAH2;AKT1 |
| Metabolism of nitric oxide_Homo sapiens_R-HSA-202131 | 2/20 | 0.022 | 0.062 | DDAH2;AKT1 |
| p53-Independent DNA Damage Response_Homo sapiens_R-HSA-69610 | 3/52 | 0.022 | 0.062 | PSMC6;PSMC3;PSMC1 |
| p53-Independent G1/S DNA damage checkpoint_Homo sapiens_R-HSA-69613 | 3/52 | 0.022 | 0.062 | PSMC6;PSMC3;PSMC1 |
| Ubiquitin Mediated Degradation of Phosphorylated Cdc25A_Homo sapiens_R-HSA-69601 | 3/52 | 0.022 | 0.062 | PSMC6;PSMC3;PSMC1 |
| Nonhomologous End-Joining (NHEJ)_Homo sapiens_R-HSA-5693571 | 3/52 | 0.022 | 0.062 | XRCC6;RIF1;H2AFX |
| mRNA Splicing - Minor Pathway_Homo sapiens_R-HSA-72165 | 3/52 | 0.022 | 0.062 | SF3B4;SF3B5;SNRNP35 |
| Vif-mediated degradation of APOBEC3G_Homo sapiens_R-HSA-180585 | 3/53 | 0.023 | 0.065 | PSMC6;PSMC3;PSMC1 |
| GPVI-mediated activation cascade_Homo sapiens_R-HSA-114604 | 3/53 | 0.023 | 0.065 | SHC1;AKT1;PTPN11 |
| Factors involved in megakaryocyte development and platelet production_Homo sapiens_R-HSA-983231 | 5/141 | 0.024 | 0.066 | HDAC2;SIN3A;ABL1;EP300;SH2B2 |
| Mitotic Spindle Checkpoint_Homo sapiens_R-HSA-69618 | 2/21 | 0.024 | 0.067 | UBE2E1;UBE2D1 |
| Degradation of AXIN_Homo sapiens_R-HSA-4641257 | 3/54 | 0.024 | 0.067 | PSMC6;PSMC3;PSMC1 |
| SCF-beta-TrCP mediated degradation of Emi1_Homo sapiens_R-HSA-174113 | 3/54 | 0.024 | 0.067 | PSMC6;PSMC3;PSMC1 |
| Stabilization of p53_Homo sapiens_R-HSA-69541 | 3/54 | 0.024 | 0.067 | PSMC6;PSMC3;PSMC1 |
| Formation of a pool of free 40S subunits_Homo sapiens_R-HSA-72689 | 4/96 | 0.025 | 0.069 | RPL37A;RPSA;RPL28;RPL6 |
| Hh mutants that don't undergo autocatalytic processing are degraded by ERAD_Homo sapiens_R-HSA-5362768 | 3/55 | 0.025 | 0.070 | PSMC6;PSMC3;PSMC1 |
| Synthesis of DNA_Homo sapiens_R-HSA-69239 | 4/97 | 0.026 | 0.070 | CDKN1A;PSMC6;PSMC3;PSMC1 |
| Generic Transcription Pathway_Homo sapiens_R-HSA-212436 | 16/812 | 0.026 | 0.070 | BNIP3L;CDKN1A;HDAC2;NPM1;ZNF274;ZNF324B;UBE2D1;NOC2L;ESR1;AR;TRIM28;RHEB;AKT1;EP300;ZNF468;SKIL |
| Degradation of DVL_Homo sapiens_R-HSA-4641258 | 3/56 | 0.027 | 0.072 | PSMC6;PSMC3;PSMC1 |
| Hh mutants abrogate ligand secretion_Homo sapiens_R-HSA-5387390 | 3/57 | 0.028 | 0.075 | PSMC6;PSMC3;PSMC1 |
| Meiotic synapsis_Homo sapiens_R-HSA-1221632 | 3/57 | 0.028 | 0.075 | H2AFX;TINF2;TERF1 |
| APC/C:Cdc20 mediated degradation of Cyclin B_Homo sapiens_R-HSA-174048 | 2/23 | 0.028 | 0.076 | UBE2E1;UBE2D1 |
| Downregulation of SMAD2/3:SMAD4 transcriptional activity_Homo sapiens_R-HSA-2173795 | 2/23 | 0.028 | 0.076 | UBE2D1;SKIL |
| CDT1 association with the CDC6:ORC:origin complex_Homo sapiens_R-HSA-68827 | 3/58 | 0.029 | 0.077 | PSMC6;PSMC3;PSMC1 |
| NIK-->noncanonical NF-kB signaling_Homo sapiens_R-HSA-5676590 | 3/58 | 0.029 | 0.077 | PSMC6;PSMC3;PSMC1 |
| B-WICH complex positively regulates rRNA expression_Homo sapiens_R-HSA-5250924 | 3/58 | 0.029 | 0.077 | MYBBP1A;H2AFX;EP300 |
| Degradation of GLI2 by the proteasome_Homo sapiens_R-HSA-5610783 | 3/59 | 0.030 | 0.079 | PSMC6;PSMC3;PSMC1 |
| Degradation of GLI1 by the proteasome_Homo sapiens_R-HSA-5610780 | 3/59 | 0.030 | 0.079 | PSMC6;PSMC3;PSMC1 |
| GLI3 is processed to GLI3R by the proteasome_Homo sapiens_R-HSA-5610785 | 3/59 | 0.030 | 0.079 | PSMC6;PSMC3;PSMC1 |
| Telomere Maintenance_Homo sapiens_R-HSA-157579 | 3/59 | 0.030 | 0.079 | H2AFX;TINF2;TERF1 |
| Growth hormone receptor signaling_Homo sapiens_R-HSA-982772 | 2/24 | 0.031 | 0.080 | STAT5B;IRS2 |
| DNA Replication_Homo sapiens_R-HSA-69306 | 4/105 | 0.033 | 0.084 | CDKN1A;PSMC6;PSMC3;PSMC1 |
| Dectin-1 mediated noncanonical NF-kB signaling_Homo sapiens_R-HSA-5607761 | 3/61 | 0.033 | 0.084 | PSMC6;PSMC3;PSMC1 |
| Cell death signalling via NRAGE, NRIF and NADE_Homo sapiens_R-HSA-204998 | 3/61 | 0.033 | 0.084 | MAGED1;ARHGEF17;ITSN1 |
| APC-Cdc20 mediated degradation of Nek2A_Homo sapiens_R-HSA-179409 | 2/25 | 0.033 | 0.084 | UBE2E1;UBE2D1 |
| Constitutive Signaling by AKT1 E17K in Cancer_Homo sapiens_R-HSA-5674400 | 2/25 | 0.033 | 0.084 | CDKN1A;AKT1 |
| ADP signalling through P2Y purinoceptor 1_Homo sapiens_R-HSA-418592 | 2/25 | 0.033 | 0.084 | SRC;GNAQ |
| Synthesis of IP3 and IP4 in the cytosol_Homo sapiens_R-HSA-1855204 | 2/25 | 0.033 | 0.084 | INPP5D;PLCG1 |
| 3' -UTR-mediated translational regulation_Homo sapiens_R-HSA-157279 | 4/106 | 0.034 | 0.085 | RPL37A;RPSA;RPL28;RPL6 |
| L13a-mediated translational silencing of Ceruloplasmin expression_Homo sapiens_R-HSA-156827 | 4/106 | 0.034 | 0.085 | RPL37A;RPSA;RPL28;RPL6 |
| Nonsense Mediated Decay (NMD) enhanced by the Exon Junction Complex (EJC)_Homo sapiens_R-HSA-975957 | 4/106 | 0.034 | 0.085 | RPL37A;RPSA;RPL28;RPL6 |
| Nonsense-Mediated Decay (NMD)_Homo sapiens_R-HSA-927802 | 4/106 | 0.034 | 0.085 | RPL37A;RPSA;RPL28;RPL6 |
| GTP hydrolysis and joining of the 60S ribosomal subunit_Homo sapiens_R-HSA-72706 | 4/107 | 0.035 | 0.086 | RPL37A;RPSA;RPL28;RPL6 |
| Mitotic Prophase_Homo sapiens_R-HSA-68875 | 4/107 | 0.035 | 0.086 | GOLGA2;RAB1B;H2AFX;BANF1 |
| SRP-dependent cotranslational protein targeting to membrane_Homo sapiens_R-HSA-1799339 | 4/107 | 0.035 | 0.086 | RPL37A;RPSA;RPL28;RPL6 |
| Downregulation of TGF-beta receptor signaling_Homo sapiens_R-HSA-2173788 | 2/26 | 0.036 | 0.086 | UCHL5;TGFBR1 |
| Cellular response to hypoxia_Homo sapiens_R-HSA-2262749 | 2/26 | 0.036 | 0.086 | UBE2D1;EP300 |
| Regulation of Hypoxia-inducible Factor (HIF) by oxygen_Homo sapiens_R-HSA-1234174 | 2/26 | 0.036 | 0.086 | UBE2D1;EP300 |
| PTK6 Regulates RHO GTPases, RAS GTPase and MAP kinases_Homo sapiens_R-HSA-8849471 | 2/26 | 0.036 | 0.086 | CRK;BCAR1 |
| Attenuation phase_Homo sapiens_R-HSA-3371568 | 2/26 | 0.036 | 0.086 | HSPA8;EP300 |
| PD-1 signaling_Homo sapiens_R-HSA-389948 | 2/26 | 0.036 | 0.086 | CD4;PTPN11 |
| Asymmetric localization of PCP proteins_Homo sapiens_R-HSA-4608870 | 3/63 | 0.036 | 0.086 | PSMC6;PSMC3;PSMC1 |
| Hedgehog ligand biogenesis_Homo sapiens_R-HSA-5358346 | 3/64 | 0.037 | 0.090 | PSMC6;PSMC3;PSMC1 |
| EGFR downregulation_Homo sapiens_R-HSA-182971 | 2/27 | 0.038 | 0.091 | SH3KBP1;CBL |
| Caspase activation via extrinsic apoptotic signalig pathway_Homo sapiens_R-HSA-5357769 | 2/27 | 0.038 | 0.091 | MAGED1;TRAF2 |
| Interleukin-6 family signaling_Homo sapiens_R-HSA-6783589 | 2/27 | 0.038 | 0.091 | PTPN11;CBL |
| ER-Phagosome pathway_Homo sapiens_R-HSA-1236974 | 3/65 | 0.039 | 0.092 | PSMC6;PSMC3;PSMC1 |
| Selenoamino acid metabolism_Homo sapiens_R-HSA-2408522 | 4/111 | 0.039 | 0.093 | RPL37A;RPSA;RPL28;RPL6 |
| Separation of Sister Chromatids_Homo sapiens_R-HSA-2467813 | 5/162 | 0.040 | 0.093 | PSMC6;PSMC3;PSMC1;UBE2E1;UBE2D1 |
| G1/S Transition_Homo sapiens_R-HSA-69206 | 4/112 | 0.040 | 0.094 | CDKN1A;PSMC6;PSMC3;PSMC1 |
| Regulation of RAS by GAPs_Homo sapiens_R-HSA-5658442 | 3/67 | 0.042 | 0.097 | PSMC6;PSMC3;PSMC1 |
| Assembly of the pre-replicative complex_Homo sapiens_R-HSA-68867 | 3/67 | 0.042 | 0.097 | PSMC6;PSMC3;PSMC1 |
| Integrin cell surface interactions_Homo sapiens_R-HSA-216083 | 3/67 | 0.042 | 0.097 | FGB;ITGA3;FN1 |
| Cap-dependent Translation Initiation_Homo sapiens_R-HSA-72737 | 4/114 | 0.043 | 0.098 | RPL37A;RPSA;RPL28;RPL6 |
| Eukaryotic Translation Initiation_Homo sapiens_R-HSA-72613 | 4/114 | 0.043 | 0.098 | RPL37A;RPSA;RPL28;RPL6 |
| Major pathway of rRNA processing in the nucleolus_Homo sapiens_R-HSA-6791226 | 5/166 | 0.043 | 0.099 | PDCD11;RPL37A;RPSA;RPL28;RPL6 |
| CDO in myogenesis_Homo sapiens_R-HSA-375170 | 2/29 | 0.044 | 0.099 | ABL1;BNIP2 |
| Myogenesis_Homo sapiens_R-HSA-525793 | 2/29 | 0.044 | 0.099 | ABL1;BNIP2 |
| Transcriptional Regulation by TP53_Homo sapiens_R-HSA-3700989 | 8/348 | 0.049 | 0.110 | BNIP3L;CDKN1A;HDAC2;NPM1;RHEB;AKT1;EP300;NOC2L |

**Supplementary table S17.** Previously reported IPF transcriptome data for comparative analysis of interactome network between HDLI and IPF

| Gene Symbol | Gene Name | Unigene ID |
| --- | --- | --- |
| AGTR1 | angiotensin II receptor type 1(AGTR1) | Hs.744837 |
| DOK5 | docking protein 5(DOK5) | Hs.473133 |
| ARHGAP31 | Rho GTPase activating protein 31(ARHGAP31) | Hs.668218 |
| RAB17 | RAB17, member RAS oncogene family(RAB17) | Hs.44278 |
| FUT2 | fucosyltransferase 2(FUT2) | Hs.579928 |
| TRAF5 | TNF receptor associated factor 5(TRAF5) | Hs.523930 |
| ROR2 | receptor tyrosine kinase like orphan receptor 2(ROR2) | Hs.98255 |
| HYAL1 | hyaluronoglucosaminidase 1(HYAL1) | Hs.75619 |
| APOBEC3H | apolipoprotein B mRNA editing enzyme catalytic subunit 3H(APOBEC3H) | Hs.440515 |
| MVB12B | multivesicular body subunit 12B(MVB12B) | Hs.162659 |
| MGLL | monoglyceride lipase(MGLL) | Hs.277035 |
| CLDN1 | claudin 1(CLDN1) | Hs.439060 |
| ITSN2 | intersectin 2(ITSN2) | Hs.432562 |
| ALDH1A3 | aldehyde dehydrogenase 1 family member A3(ALDH1A3) | Hs.459538 |
| FITM2 | fat storage inducing transmembrane protein 2(FITM2) | Hs.632269 |
| GALNT6 | polypeptide N-acetylgalactosaminyltransferase 6(GALNT6) | Hs.505575 |
| EFNA5 | ephrin A5(EFNA5) | Hs.288741 |
| IL1RN | interleukin 1 receptor antagonist(IL1RN) | Hs.81134 |
| FBLN2 | fibulin 2(FBLN2) | Hs.198862 |
| KIAA1211 | KIAA1211(KIAA1211) | Hs.596667 |
| NEDD9 | neural precursor cell expressed, developmentally down-regulated 9(NEDD9) | Hs.37982 |
| LRRN3 | leucine rich repeat neuronal 3(LRRN3) | Hs.3781 |
| CCL13 | C-C motif chemokine ligand 13(CCL13) | Hs.414629 |
| PLA2G7 | phospholipase A2 group VII(PLA2G7) | Hs.584823 |
| QKI | QKI, KH domain containing RNA binding(QKI) | Hs.510324 |
| ASB2 | ankyrin repeat and SOCS box containing 2(ASB2) | Hs.510327 |
| C6orf223 | chromosome 6 open reading frame 223(C6orf223) | Hs.733478 |
| TOX2 | TOX high mobility group box family member 2(TOX2) | Hs.26608 |
| LOC100128170 | uncharacterized LOC100128170(LOC100128170) | Hs.26608 |
| MOXD1 | monooxygenase DBH like 1(MOXD1) | Hs.6909 |
| PRX | periaxin(PRX) | Hs.205457 |
| PLAU | plasminogen activator, urokinase(PLAU) | Hs.77274 |
| TMEM45A | transmembrane protein 45A(TMEM45A) | Hs.658956 |
| IRF4 | interferon regulatory factor 4(IRF4) | Hs.401013 |
| SERPINB13 | serpin family B member 13(SERPINB13) | Hs.241407 |
| HOPX | HOP homeobox(HOPX) | Hs.619396 |
| SMAD6 | SMAD family member 6(SMAD6) | Hs.153863 |
| KL | klotho(KL) | Hs.524953 |
| SULF1 | sulfatase 1(SULF1) | Hs.409602 |
| FZD5 | frizzled class receptor 5(FZD5) | Hs.17631 |
| NTN1 | netrin 1(NTN1) | Hs.660885 |
| GORASP1 | golgi reassembly stacking protein 1(GORASP1) | Hs.721946 |
| PALM2-AKAP2 | PALM2-AKAP2 readthrough(PALM2-AKAP2) | Hs.591908 |
| AKAP2 | A-kinase anchoring protein 2(AKAP2) | Hs.591908 |
| PALM2 | paralemmin 2(PALM2) | Hs.591908 |
| LIMS2 | LIM zinc finger domain containing 2(LIMS2) | Hs.469881 |
| PDK4 | pyruvate dehydrogenase kinase 4(PDK4) | Hs.8364 |
| SLN | sarcolipin(SLN) | Hs.334629 |
| TMEM156 | transmembrane protein 156(TMEM156) | Hs.374147 |
| PLAG1 | PLAG1 zinc finger(PLAG1) | Hs.14968 |
| CLIC5 | chloride intracellular channel 5(CLIC5) | Hs.734348 |
| DLL4 | delta like canonical Notch ligand 4(DLL4) | Hs.511076 |
| KIAA1462 | KIAA1462(KIAA1462) | Hs.533953 |
| ADAMTS16 | ADAM metallopeptidase with thrombospondin type 1 motif 16(ADAMTS16) | Hs.661915 |
| WWC2 | WW and C2 domain containing 2(WWC2) | Hs.333179 |
| CLDN22 | claudin 22(CLDN22) | Hs.333179 |
| CFI | complement factor I(CFI) | Hs.312485 |
| VTCN1 | V-set domain containing T cell activation inhibitor 1(VTCN1) | Hs.546434 |
| VEPH1 | ventricular zone expressed PH domain containing 1(VEPH1) | Hs.658046 |
| PDLIM3 | PDZ and LIM domain 3(PDLIM3) | Hs.701364 |
| FMO1 | flavin containing monooxygenase 1(FMO1) | Hs.1424 |
| PLCH2 | phospholipase C eta 2(PLCH2) | Hs.170156 |
| NFATC2 | nuclear factor of activated T-cells 2(NFATC2) | Hs.744148 |
| STXBP6 | syntaxin binding protein 6(STXBP6) | Hs.508958 |
| APOBEC3C | apolipoprotein B mRNA editing enzyme catalytic subunit 3C(APOBEC3C) | Hs.441124 |
| PPL | periplakin(PPL) | Hs.192233 |
| cpa6 | carboxypeptidase A6(CPA6) | Hs.658850 |
| TMEM87A | transmembrane protein 87A(TMEM87A) | Hs.744941 |
| TMEM204 | transmembrane protein 204(TMEM204) | Hs.459652 |
| PARD3 | par-3 family cell polarity regulator(PARD3) | Hs.131489 |
| COLEC11 | collectin subfamily member 11(COLEC11) | Hs.735715 |
| EFR3B | EFR3 homolog B(EFR3B) | Hs.4892 |
| VAMP5 | vesicle associated membrane protein 5(VAMP5) | Hs.534373 |
| ENG | endoglin(ENG) | Hs.76753 |
| INPP5K | inositol polyphosphate-5-phosphatase K(INPP5K) | Hs.632238 |
| CCL19 | C-C motif chemokine ligand 19(CCL19) | Hs.50002 |
| TSPAN13 | tetraspanin 13(TSPAN13) | Hs.364544 |
| FGFBP2 | fibroblast growth factor binding protein 2(FGFBP2) | Hs.98785 |
| ARNTL2 | aryl hydrocarbon receptor nuclear translocator like 2(ARNTL2) | Hs.445447 |
| SRGAP2 | SLIT-ROBO Rho GTPase activating protein 2(SRGAP2) | Hs.744555 |
| SRGAP2B | SLIT-ROBO Rho GTPase activating protein 2B(SRGAP2B) | Hs.744555 |
| HRH1 | histamine receptor H1(HRH1) | Hs.1570 |
| TMEM154 | transmembrane protein 154(TMEM154) | Hs.518900 |
| OSGIN2 | oxidative stress induced growth inhibitor family member 2(OSGIN2) | Hs.436445 |
| GABBR2 | gamma-aminobutyric acid type B receptor subunit 2(GABBR2) | Hs.198612 |
| USP44 | ubiquitin specific peptidase 44(USP44) | Hs.646421 |
| ZBTB16 | zinc finger and BTB domain containing 16(ZBTB16) | Hs.682144 |
| PTGFRN | prostaglandin F2 receptor inhibitor(PTGFRN) | Hs.418093 |
| ADAMTSL3 | ADAMTS like 3(ADAMTSL3) | Hs.459162 |
| SMAD7 | SMAD family member 7(SMAD7) | Hs.465087 |
| SEMA6A | semaphorin 6A(SEMA6A) | Hs.156967 |
| PCDH7 | protocadherin 7(PCDH7) | Hs.479439 |
| TXNIP | thioredoxin interacting protein(TXNIP) | Hs.533977 |
| ACAT2 | acetyl-CoA acetyltransferase 2(ACAT2) | Hs.571037 |
| PNOC | prepronociceptin(PNOC) | Hs.88218 |
| CCDC68 | coiled-coil domain containing 68(CCDC68) | Hs.120790 |
| VIP | vasoactive intestinal peptide(VIP) | Hs.53973 |
| TNS3 | tensin 3(TNS3) | Hs.520814 |
| SFRP4 | secreted frizzled related protein 4(SFRP4) | Hs.658169 |
| SPRYD7 | SPRY domain containing 7(SPRYD7) | Hs.44235 |
| tmem125 | transmembrane protein 125(TMEM125) | Hs.104476 |
| APCDD1 | APC down-regulated 1(APCDD1) | Hs.293274 |
| SREBF1 | sterol regulatory element binding transcription factor 1(SREBF1) | Hs.592123 |
| ABCA3 | ATP binding cassette subfamily A member 3(ABCA3) | Hs.26630 |
| RAB32 | RAB32, member RAS oncogene family(RAB32) | Hs.287714 |
| SPOCK1 | SPARC/osteonectin, cwcv and kazal like domains proteoglycan 1(SPOCK1) | Hs.596136 |
| TGFB3 | transforming growth factor beta 3(TGFB3) | Hs.736373 |
| PYM1 | PYM homolog 1, exon junction complex associated factor(PYM1) | Hs.524488 |
| DGKA | diacylglycerol kinase alpha(DGKA) | Hs.524488 |
| SEMA3B | semaphorin 3B(SEMA3B) | Hs.82222 |
| VIPR1 | vasoactive intestinal peptide receptor 1(VIPR1) | Hs.348500 |
| SGCG | sarcoglycan gamma(SGCG) | Hs.37167 |
| RARRES1 | retinoic acid receptor responder 1(RARRES1) | Hs.131269 |
| KRT5 | keratin 5(KRT5) | Hs.433845 |
| HYAL2 | hyaluronoglucosaminidase 2(HYAL2) | Hs.76873 |
| PAPSS2 | 3'-phosphoadenosine 5'-phosphosulfate synthase 2(PAPSS2) | Hs.524491 |
| PARD6B | par-6 family cell polarity regulator beta(PARD6B) | Hs.589848 |
| LILRA1 | leukocyte immunoglobulin like receptor A1(LILRA1) | Hs.710507 |
| LRRC32 | leucine rich repeat containing 32(LRRC32) | Hs.151641 |
| FERMT1 | fermitin family member 1(FERMT1) | Hs.472054 |
| RBP5 | retinol binding protein 5(RBP5) | Hs.246046 |
| SLC39A6 | solute carrier family 39 member 6(SLC39A6) | Hs.79136 |
| S100A2 | S100 calcium binding protein A2(S100A2) | Hs.516484 |
| SLC28A3 | solute carrier family 28 member 3(SLC28A3) | Hs.535966 |
| CCL11 | C-C motif chemokine ligand 11(CCL11) | Hs.54460 |
| TNC | tenascin C(TNC) | Hs.143250 |
| IGLL5 | immunoglobulin lambda like polypeptide 5(IGLL5) | Hs.713252 |
| TRPS1 | transcriptional repressor GATA binding 1(TRPS1) | Hs.657018 |
| NOVA2 | NOVA alternative splicing regulator 2(NOVA2) | Hs.375439 |
| FAM84B | family with sequence similarity 84 member B(FAM84B) | Hs.741352 |
| EPB41L5 | erythrocyte membrane protein band 4.1 like 5(EPB41L5) | Hs.369232 |
| CHEK1 | checkpoint kinase 1(CHEK1) | Hs.24529 |
| PGM2L1 | phosphoglucomutase 2 like 1(PGM2L1) | Hs.26612 |
| LSS | lanosterol synthase (2,3-oxidosqualene-lanosterol cyclase)(LSS) | Hs.596543 |
| MGST1 | microsomal glutathione S-transferase 1(MGST1) | Hs.389700 |
| SECISBP2L | SECIS binding protein 2 like(SECISBP2L) | Hs.9997 |
| NCOA3 | nuclear receptor coactivator 3(NCOA3) | Hs.592142 |
| P3H2 | prolyl 3-hydroxylase 2(P3H2) | Hs.374191 |
| CD28 | CD28 molecule(CD28) | Hs.443123 |
| ADRA2A | adrenoceptor alpha 2A(ADRA2A) | Hs.249159 |
| PPP2R5A | protein phosphatase 2 regulatory subunit B'alpha(PPP2R5A) | Hs.744012 |
| itga7 | integrin subunit alpha 7(ITGA7) | Hs.524484 |
| CTNNBIP1 | catenin beta interacting protein 1(CTNNBIP1) | Hs.463759 |
| SEC11C | SEC11 homolog C, signal peptidase complex subunit(SEC11C) | Hs.45107 |
| ENC1 | ectodermal-neural cortex 1(ENC1) | Hs.744844 |
| MATN3 | matrilin 3(MATN3) | Hs.656199 |
| CASQ1 | calsequestrin 1(CASQ1) | Hs.632476 |
| PKIB | protein kinase (cAMP-dependent, catalytic) inhibitor beta(PKIB) | Hs.741340 |
| DAPK1 | death associated protein kinase 1(DAPK1) | Hs.380277 |
| MAP2 | microtubule associated protein 2(MAP2) | Hs.368281 |
| BMP4 | bone morphogenetic protein 4(BMP4) | Hs.68879 |
| SCAI | suppressor of cancer cell invasion(SCAI) | Hs.59504 |
| GOLGA1 | golgin A1(GOLGA1) | Hs.59504 |
| CROT | carnitine O-octanoyltransferase(CROT) | Hs.125039 |
| PLXDC1 | plexin domain containing 1(PLXDC1) | Hs.125036 |
| SYNJ2 | synaptojanin 2(SYNJ2) | Hs.434494 |
| POSTN | periostin(POSTN) | Hs.721018 |
| MDK | midkine (neurite growth-promoting factor 2)(MDK) | Hs.82045 |
| FAM83B | family with sequence similarity 83 member B(FAM83B) | Hs.152423 |
| BMP2 | bone morphogenetic protein 2(BMP2) | Hs.73853 |
| GBA2 | glucosylceramidase beta 2(GBA2) | Hs.443134 |
| CD200 | CD200 molecule(CD200) | Hs.79015 |
| CD38 | CD38 molecule(CD38) | Hs.479214 |
| CLIC3 | chloride intracellular channel 3(CLIC3) | Hs.64746 |
| FXYD6-FXYD2 | FXYD6-FXYD2 readthrough(FXYD6-FXYD2) | Hs.744850 |
| FXYD6 | FXYD domain containing ion transport regulator 6(FXYD6) | Hs.744850 |
| CLDN5 | claudin 5(CLDN5) | Hs.505337 |
| COL6A3 | collagen type VI alpha 3 chain(COL6A3) | Hs.233240 |
| EMCN | endomucin(EMCN) | Hs.152913 |
| PRELP | proline and arginine rich end leucine rich repeat protein(PRELP) | Hs.632481 |
| CHRM3 | cholinergic receptor muscarinic 3(CHRM3) | Hs.7138 |
| gcnt2 | glucosaminyl (N-acetyl) transferase 2, I-branching enzyme (I blood group)(GCNT2) | Hs.519884 |
| CA2 | carbonic anhydrase 2(CA2) | Hs.155097 |
| MACF1 | microtubule-actin crosslinking factor 1(MACF1) | Hs.692278 |
| BACE2 | beta-site APP-cleaving enzyme 2(BACE2) | Hs.529408 |
| ABCC6 | ATP binding cassette subfamily C member 6(ABCC6) | Hs.442182 |
| ACTG2 | actin, gamma 2, smooth muscle, enteric(ACTG2) | Hs.516105 |
| RNASE2 | ribonuclease A family member 2(RNASE2) | Hs.728 |
| LEF1 | lymphoid enhancer binding factor 1(LEF1) | Hs.743478 |
| ESYT3 | extended synaptotagmin 3(ESYT3) | Hs.477711 |
| THRA | thyroid hormone receptor, alpha(THRA) | Hs.724 |
| NR1D1 | nuclear receptor subfamily 1 group D member 1(NR1D1) | Hs.724 |
| RASSF7 | Ras association domain family member 7(RASSF7) | Hs.72925 |
| DENND3 | DENN domain containing 3(DENND3) | Hs.18166 |
| MFAP3L | microfibrillar associated protein 3 like(MFAP3L) | Hs.593942 |
| GPC3 | glypican 3(GPC3) | Hs.644108 |
| INPP5D | inositol polyphosphate-5-phosphatase D(INPP5D) | Hs.601911 |
| HAPLN3 | hyaluronan and proteoglycan link protein 3(HAPLN3) | Hs.447530 |
| GLB1L3 | galactosidase beta 1 like 3(GLB1L3) | Hs.721382 |
| TBX5 | T-box 5(TBX5) | Hs.381715 |
| RRAS | related RAS viral (r-ras) oncogene homolog(RRAS) | Hs.515536 |
| CTSK | cathepsin K(CTSK) | Hs.632466 |
| S1PR1 | sphingosine-1-phosphate receptor 1(S1PR1) | Hs.154210 |
| osr2 | odd-skipped related transciption factor 2(OSR2) | Hs.253247 |
| TUBB2A | tubulin beta 2A class IIa(TUBB2A) | Hs.654543 |
| rhov | ras homolog family member V(RHOV) | Hs.447901 |
| baat | bile acid-CoA:amino acid N-acyltransferase(BAAT) | Hs.284712 |
| MYLIP | myosin regulatory light chain interacting protein(MYLIP) | Hs.484738 |
| COL24A1 | collagen type XXIV alpha 1 chain(COL24A1) | Hs.659516 |
| COL14A1 | collagen type XIV alpha 1 chain(COL14A1) | Hs.657621 |
| LINC01290 | long intergenic non-protein coding RNA 1290(LINC01290) | Hs.531561 |
| EMP2 | epithelial membrane protein 2(EMP2) | Hs.531561 |
| NOTCH4 | notch 4(NOTCH4) | Hs.436100 |
| ZNF267 | zinc finger protein 267(ZNF267) | Hs.460645 |
| MMP1 | matrix metallopeptidase 1(MMP1) | Hs.83169 |
| RAPGEF5 | Rap guanine nucleotide exchange factor 5(RAPGEF5) | Hs.713217 |
| BCL11A | B-cell CLL/lymphoma 11A(BCL11A) | Hs.370549 |
| SKAP1 | src kinase associated phosphoprotein 1(SKAP1) | Hs.316931 |
| TMEM98 | transmembrane protein 98(TMEM98) | Hs.3447 |
| LTBP1 | latent transforming growth factor beta binding protein 1(LTBP1) | Hs.619315 |
| LLGL2 | LLGL2, scribble cell polarity complex component(LLGL2) | Hs.514477 |
| RHOH | ras homolog family member H(RHOH) | Hs.738915 |
| SNAP25 | synaptosome associated protein 25(SNAP25) | Hs.167317 |
| TNIP3 | TNFAIP3 interacting protein 3(TNIP3) | Hs.208206 |
| krt17 | keratin 17(KRT17) | Hs.2785 |
| LAMA3 | laminin subunit alpha 3(LAMA3) | Hs.436367 |
| syt8 | synaptotagmin 8(SYT8) | Hs.738918 |
| CBSL | cystathionine-beta-synthase like(CBSL) | Hs.533013 |
| CBS | cystathionine-beta-synthase(CBS) | Hs.533013 |
| EPAS1 | endothelial PAS domain protein 1(EPAS1) | Hs.468410 |
| EXOC3L1 | exocyst complex component 3 like 1(EXOC3L1) | Hs.647356 |
| FAM46C | family with sequence similarity 46 member C(FAM46C) | Hs.356216 |
| IGDCC4 | immunoglobulin superfamily DCC subclass member 4(IGDCC4) | Hs.458607 |
| BCHE | butyrylcholinesterase(BCHE) | Hs.420483 |
| MUC5B | mucin 5B, oligomeric mucus/gel-forming(MUC5B) | Hs.523395 |
| SMOC2 | SPARC related modular calcium binding 2(SMOC2) | Hs.487200 |
| CYP51A1 | cytochrome P450 family 51 subfamily A member 1(CYP51A1) | Hs.417077 |
| PTHLH | parathyroid hormone like hormone(PTHLH) | Hs.591159 |
| TPBG | trophoblast glycoprotein(TPBG) | Hs.71947 |
| NINJ1 | ninjurin 1(NINJ1) | Hs.494457 |
| CKAP2 | cytoskeleton associated protein 2(CKAP2) | Hs.444028 |
| FAM105A | family with sequence similarity 105 member A(FAM105A) | Hs.155085 |
| UGCG | UDP-glucose ceramide glucosyltransferase(UGCG) | Hs.593014 |
| RHBDL2 | rhomboid like 2(RHBDL2) | Hs.524626 |
| GRK5 | G protein-coupled receptor kinase 5(GRK5) | Hs.524625 |
| DOCK9 | dedicator of cytokinesis 9(DOCK9) | Hs.596105 |
| ITGA11 | integrin subunit alpha 11(ITGA11) | Hs.458733 |
| LIFR | leukemia inhibitory factor receptor alpha(LIFR) | Hs.657602 |
| LIFR-AS1 | LIFR antisense RNA 1(LIFR-AS1) | Hs.657602 |
| GM2A | GM2 ganglioside activator(GM2A) | Hs.483873 |
| aldh3a1 | aldehyde dehydrogenase 3 family member A1(ALDH3A1) | Hs.531682 |
| ADAMTS14 | ADAM metallopeptidase with thrombospondin type 1 motif 14(ADAMTS14) | Hs.352156 |
| CD27 | CD27 molecule(CD27) | Hs.355307 |
| WNT7A | Wnt family member 7A(WNT7A) | Hs.72290 |
| TDO2 | tryptophan 2,3-dioxygenase(TDO2) | Hs.593955 |
| SLAIN1 | SLAIN motif family member 1(SLAIN1) | Hs.349955 |
| ST6GALNAC5 | ST6 N-acetylgalactosaminide alpha-2,6-sialyltransferase 5(ST6GALNAC5) | Hs.303609 |
| DUSP4 | dual specificity phosphatase 4(DUSP4) | Hs.417962 |
| LRRN1 | leucine rich repeat neuronal 1(LRRN1) | Hs.163244 |
| TNS4 | tensin 4(TNS4) | Hs.438292 |
| ITGB4 | integrin subunit beta 4(ITGB4) | Hs.632226 |
| IGFBPL1 | insulin like growth factor binding protein like 1(IGFBPL1) | Hs.349705 |
| COMP | cartilage oligomeric matrix protein(COMP) | Hs.1584 |
| CLCA2 | chloride channel accessory 2(CLCA2) | Hs.241551 |
| SLIT2 | slit guidance ligand 2(SLIT2) | Hs.29802 |
| SLC1A1 | solute carrier family 1 member 1(SLC1A1) | Hs.444915 |
| MPP5 | membrane palmitoylated protein 5(MPP5) | Hs.652312 |
| DUSP8 | dual specificity phosphatase 8(DUSP8) | Hs.41688 |
| BLVRB | biliverdin reductase B(BLVRB) | Hs.515785 |
| SMO | smoothened, frizzled class receptor(SMO) | Hs.437846 |
| CSF3R | colony stimulating factor 3 receptor(CSF3R) | Hs.524517 |
| ANXA3 | annexin A3(ANXA3) | Hs.480042 |
| KDR | kinase insert domain receptor(KDR) | Hs.479756 |
| HSD17B6 | hydroxysteroid 17-beta dehydrogenase 6(HSD17B6) | Hs.524513 |
| slc39a8 | solute carrier family 39 member 8(SLC39A8) | Hs.288034 |
| CD79A | CD79a molecule(CD79A) | Hs.631567 |
| CDH5 | cadherin 5(CDH5) | Hs.76206 |
| ALDH1B1 | aldehyde dehydrogenase 1 family member B1(ALDH1B1) | Hs.743532 |
| DSC3 | desmocollin 3(DSC3) | Hs.41690 |
| PLLP | plasmolipin(PLLP) | Hs.632215 |
| ID1 | inhibitor of DNA binding 1, HLH protein(ID1) | Hs.504609 |
| LMTK2 | lemur tyrosine kinase 2(LMTK2) | Hs.444179 |
| CXCL14 | C-X-C motif chemokine ligand 14(CXCL14) | Hs.483444 |
| CPNE5 | copine 5(CPNE5) | Hs.657869 |
| slc9a3r2 | SLC9A3 regulator 2(SLC9A3R2) | Hs.440896 |
| LAMC3 | laminin subunit gamma 3(LAMC3) | Hs.201805 |
| MRVI1 | murine retrovirus integration site 1 homolog(MRVI1) | Hs.680194 |
| BIRC3 | baculoviral IAP repeat containing 3(BIRC3) | Hs.127799 |
| ZNF215 | zinc finger protein 215(ZNF215) | Hs.523457 |
| SPRY4 | sprouty RTK signaling antagonist 4(SPRY4) | Hs.323308 |
| SORCS2 | sortilin related VPS10 domain containing receptor 2(SORCS2) | Hs.479099 |
| IMPA2 | inositol monophosphatase 2(IMPA2) | Hs.743311 |
| MYOM1 | myomesin 1(MYOM1) | Hs.464469 |
| COL15A1 | collagen type XV alpha 1 chain(COL15A1) | Hs.409034 |
| FDPS | farnesyl diphosphate synthase(FDPS) | Hs.335918 |
| GPR146 | G protein-coupled receptor 146(GPR146) | Hs.718624 |
| MMP11 | matrix metallopeptidase 11(MMP11) | Hs.143751 |
| SLC11A1 | solute carrier family 11 member 1(SLC11A1) | Hs.591607 |
| NFIA | nuclear factor I A(NFIA) | Hs.740757 |
| PDLIM4 | PDZ and LIM domain 4(PDLIM4) | Hs.424312 |
| WFDC2 | WAP four-disulfide core domain 2(WFDC2) | Hs.2719 |
| SERPINF1 | serpin family F member 1(SERPINF1) | Hs.532768 |
| CAPN13 | calpain 13(CAPN13) | Hs.660911 |
| SERPINB5 | serpin family B member 5(SERPINB5) | Hs.55279 |
| SP140 | SP140 nuclear body protein(SP140) | Hs.632549 |
| RIMKLA | ribosomal modification protein rimK like family member A(RIMKLA) | Hs.420244 |
| KRT23 | keratin 23(KRT23) | Hs.9029 |
| SSPN | sarcospan(SSPN) | Hs.736914 |
| TNNC1 | troponin C1, slow skeletal and cardiac type(TNNC1) | Hs.118845 |
| MAP4K2 | mitogen-activated protein kinase kinase kinase kinase 2(MAP4K2) | Hs.534341 |
| AGER | advanced glycosylation end-product specific receptor(AGER) | Hs.534342 |
| SUSD2 | sushi domain containing 2(SUSD2) | Hs.131819 |
| ENTPD7 | ectonucleoside triphosphate diphosphohydrolase 7(ENTPD7) | Hs.28326 |
| Cox15 | COX15, cytochrome c oxidase assembly homolog(COX15) | Hs.28326 |
| DZIP1 | DAZ interacting zinc finger protein 1(DZIP1) | Hs.742077 |
| ARID5B | AT-rich interaction domain 5B(ARID5B) | Hs.535297 |
| ACAN | aggrecan(ACAN) | Hs.2159 |
| DOCK4 | dedicator of cytokinesis 4(DOCK4) | Hs.654652 |
| B3GALNT1 | beta-1,3-N-acetylgalactosaminyltransferase 1 (globoside blood group)(B3GALNT1) | Hs.606694 |
| RASGRP1 | RAS guanyl releasing protein 1(RASGRP1) | Hs.591127 |
| VLDLR | very low density lipoprotein receptor(VLDLR) | Hs.370422 |
| ACVRL1 | activin A receptor like type 1(ACVRL1) | Hs.591026 |
| FAM153A | family with sequence similarity 153 member A(FAM153A) | Hs.368516 |
| FAM153B | family with sequence similarity 153 member B(FAM153B) | Hs.368516 |
| LOC100507387 | uncharacterized LOC100507387(LOC100507387) | Hs.368516 |
| ROR1 | receptor tyrosine kinase like orphan receptor 1(ROR1) | Hs.128753 |
| NBEAL2 | neurobeachin like 2(NBEAL2) | Hs.437043 |
| AK4 | adenylate kinase 4(AK4) | Hs.10862 |
| AARS | alanyl-tRNA synthetase(AARS) | Hs.315137 |
| MZB1 | marginal zone B and B1 cell specific protein(MZB1) | Hs.409563 |
| c1orf198 | chromosome 1 open reading frame 198(C1orf198) | Hs.520494 |
| SLED1 | proteoglycan 3 pseudogene(SLED1) | Hs.689630 |
| PTPN21 | protein tyrosine phosphatase, non-receptor type 21(PTPN21) | Hs.437040 |
| WNT10A | Wnt family member 10A(WNT10A) | Hs.121540 |
| MFAP2 | microfibrillar associated protein 2(MFAP2) | Hs.389137 |
| TRIM2 | tripartite motif containing 2(TRIM2) | Hs.435711 |
| PLXNA4 | plexin A4(PLXNA4) | Hs.511454 |
| CCND2 | cyclin D2(CCND2) | Hs.376071 |
| ATP8B4 | ATPase phospholipid transporting 8B4 (putative)(ATP8B4) | Hs.511311 |
| SLCO2A1 | solute carrier organic anion transporter family member 2A1(SLCO2A1) | Hs.518270 |
| DIO3 | deiodinase, iodothyronine type III(DIO3) | Hs.49322 |
| DRD5 | dopamine receptor D5(DRD5) | Hs.380681 |
| PCDH12 | protocadherin 12(PCDH12) | Hs.439474 |
| IGFBP4 | insulin like growth factor binding protein 4(IGFBP4) | Hs.462998 |
| AQP5 | aquaporin 5(AQP5) | Hs.298023 |
| MMP7 | matrix metallopeptidase 7(MMP7) | Hs.2256 |
| ADRB1 | adrenoceptor beta 1(ADRB1) | Hs.99913 |
| CD248 | CD248 molecule(CD248) | Hs.195727 |
| CPXM1 | carboxypeptidase X, M14 family member 1(CPXM1) | Hs.659346 |
| IL13RA2 | interleukin 13 receptor subunit alpha 2(IL13RA2) | Hs.336046 |
| RBP2 | retinol binding protein 2(RBP2) | Hs.655516 |
| TWIST1 | twist family bHLH transcription factor 1(TWIST1) | Hs.644998 |
| CXCL13 | C-X-C motif chemokine ligand 13(CXCL13) | Hs.100431 |
| MTSS1 | MTSS1, I-BAR domain containing(MTSS1) | Hs.336994 |
| PDE1A | phosphodiesterase 1A(PDE1A) | Hs.742174 |
| ZMAT3 | zinc finger matrin-type 3(ZMAT3) | Hs.371609 |
| TAOK2 | TAO kinase 2(TAOK2) | Hs.291623 |
| BNC2 | basonuclin 2(BNC2) | Hs.740070 |
| PPP4R4 | protein phosphatase 4 regulatory subunit 4(PPP4R4) | Hs.259599 |
| CDKN2B-AS1 | CDKN2B antisense RNA 1(CDKN2B-AS1) | Hs.512599 |
| CDKN2A | cyclin dependent kinase inhibitor 2A(CDKN2A) | Hs.512599 |
| CLCN4 | chloride voltage-gated channel 4(CLCN4) | Hs.495674 |
| ZXDC | ZXD family zinc finger C(ZXDC) | Hs.711046 |
| PTPRZ1 | protein tyrosine phosphatase, receptor type Z1(PTPRZ1) | Hs.489824 |
| REPS2 | RALBP1 associated Eps domain containing 2(REPS2) | Hs.186810 |
| CASP3 | caspase 3(CASP3) | Hs.141125 |
| SYNPO2 | synaptopodin 2(SYNPO2) | Hs.655519 |
| LXN | latexin(LXN) | Hs.478067 |
| CDO1 | cysteine dioxygenase type 1(CDO1) | Hs.442378 |
| HMGCS1 | 3-hydroxy-3-methylglutaryl-CoA synthase 1(HMGCS1) | Hs.397729 |
| LRRC4C | leucine rich repeat containing 4C(LRRC4C) | Hs.745123 |
| HECW2 | HECT, C2 and WW domain containing E3 ubiquitin protein ligase 2(HECW2) | Hs.654742 |
| OCLN | occludin(OCLN) | Hs.592605 |
| FKBP11 | FK506 binding protein 11(FKBP11) | Hs.655103 |
| CD19 | CD19 molecule(CD19) | Hs.652262 |
| ABCB6 | ATP binding cassette subfamily B member 6 (Langereis blood group)(ABCB6) | Hs.107911 |
| PODNL1 | podocan like 1(PODNL1) | Hs.448497 |
| ARHGEF26 | Rho guanine nucleotide exchange factor 26(ARHGEF26) | Hs.240845 |
| EZH1 | enhancer of zeste 1 polycomb repressive complex 2 subunit(EZH1) | Hs.194669 |
| PRKCD | protein kinase C delta(PRKCD) | Hs.155342 |
| STARD8 | StAR related lipid transfer domain containing 8(STARD8) | Hs.95140 |
| CXCL12 | C-X-C motif chemokine ligand 12(CXCL12) | Hs.522891 |
| HIF1A-AS2 | HIF1A antisense RNA 2(HIF1A-AS2) | Hs.597216 |
| HIF1A | hypoxia inducible factor 1 alpha subunit(HIF1A) | Hs.597216 |
| SLC27A2 | solute carrier family 27 member 2(SLC27A2) | Hs.11729 |
| THBS2 | thrombospondin 2(THBS2) | Hs.371147 |
| HGF | hepatocyte growth factor(HGF) | Hs.396530 |
| SOGA3 | SOGA family member 3(SOGA3) | Hs.319247 |
| KIAA0408 | KIAA0408(KIAA0408) | Hs.319247 |
| PCYOX1 | prenylcysteine oxidase 1(PCYOX1) | Hs.567502 |
| CR2 | complement C3d receptor 2(CR2) | Hs.445757 |
| IGFBP2 | insulin like growth factor binding protein 2(IGFBP2) | Hs.438102 |
| GLT8D2 | glycosyltransferase 8 domain containing 2(GLT8D2) | Hs.631650 |
| EMB | embigin(EMB) | Hs.561411 |
| JAM3 | junctional adhesion molecule 3(JAM3) | Hs.150718 |
| EDNRB | endothelin receptor type B(EDNRB) | Hs.743195 |
| CP | ceruloplasmin(CP) | Hs.558314 |
| HPS3 | HPS3, biogenesis of lysosomal organelles complex 2 subunit 1(HPS3) | Hs.558314 |
| ECHDC3 | enoyl-CoA hydratase domain containing 3(ECHDC3) | Hs.22242 |
| CLEC1A | C-type lectin domain family 1 member A(CLEC1A) | Hs.29549 |
| SNCAIP | synuclein alpha interacting protein(SNCAIP) | Hs.426463 |
| GCOM1 | GRINL1A complex locus 1(GCOM1) | Hs.437256 |
| MYZAP | myocardial zonula adherens protein(MYZAP) | Hs.437256 |
| polr2m | RNA polymerase II subunit M(POLR2M) | Hs.437256 |
| THBS4 | thrombospondin 4(THBS4) | Hs.211426 |
| CDH3 | cadherin 3(CDH3) | Hs.191842 |
| PSCA | prostate stem cell antigen(PSCA) | Hs.652235 |
| EYA2 | EYA transcriptional coactivator and phosphatase 2(EYA2) | Hs.722040 |
| EGFL6 | EGF like domain multiple 6(EGFL6) | Hs.12844 |
| FCMR | Fc fragment of IgM receptor(FCMR) | Hs.58831 |
| IL24 | interleukin 24(IL24) | Hs.58831 |
| CALCOCO2 | calcium binding and coiled-coil domain 2(CALCOCO2) | Hs.514920 |
| CATSPERB | cation channel sperm associated auxiliary subunit beta(CATSPERB) | Hs.131755 |
| ADAM23 | ADAM metallopeptidase domain 23(ADAM23) | Hs.591643 |
| ABCA12 | ATP binding cassette subfamily A member 12(ABCA12) | Hs.134585 |
| SLC4A7 | solute carrier family 4 member 7(SLC4A7) | Hs.250072 |
| spdef | SAM pointed domain containing ETS transcription factor(SPDEF) | Hs.485158 |
| NKD1 | naked cuticle homolog 1(NKD1) | Hs.187578 |
| PLA1A | phospholipase A1 member A(PLA1A) | Hs.437451 |
| PKNOX2 | PBX/knotted 1 homeobox 2(PKNOX2) | Hs.278564 |
| PLCE1 | phospholipase C epsilon 1(PLCE1) | Hs.655033 |
| CH25H | cholesterol 25-hydroxylase(CH25H) | Hs.47357 |
| PINK1 | PTEN induced putative kinase 1(PINK1) | Hs.389171 |
| SSR4 | signal sequence receptor subunit 4(SSR4) | Hs.409223 |
| FAM83A | family with sequence similarity 83 member A(FAM83A) | Hs.379821 |
| MUC4 | mucin 4, cell surface associated(MUC4) | Hs.369646 |
| FAT1 | FAT atypical cadherin 1(FAT1) | Hs.481371 |
| Hist1h4j | histone cluster 1 H4 family member j(HIST1H4J) | Hs.278483 |
| TTC39C | tetratricopeptide repeat domain 39C(TTC39C) | Hs.128576 |
| SSTR2 | somatostatin receptor 2(SSTR2) | Hs.514451 |
| MAN2C1 | mannosidase alpha class 2C member 1(MAN2C1) | Hs.26232 |
| LOC101929340 | uncharacterized LOC101929340(LOC101929340) | Hs.173840 |
| ESAM | endothelial cell adhesion molecule(ESAM) | Hs.173840 |
| TSPAN11 | tetraspanin 11(TSPAN11) | Hs.505141 |
| KCNMA1 | potassium calcium-activated channel subfamily M alpha 1(KCNMA1) | Hs.144795 |
| HLA-DOB | major histocompatibility complex, class II, DO beta(HLA-DOB) | Hs.1802 |
| PROM2 | prominin 2(PROM2) | Hs.469313 |
| SH2D3C | SH2 domain containing 3C(SH2D3C) | Hs.306412 |
| KANK3 | KN motif and ankyrin repeat domains 3(KANK3) | Hs.745036 |
| FNDC1 | fibronectin type III domain containing 1(FNDC1) | Hs.520525 |
| AMPD1 | adenosine monophosphate deaminase 1(AMPD1) | Hs.89570 |
| SLC27A3 | solute carrier family 27 member 3(SLC27A3) | Hs.438723 |
| INTS3 | integrator complex subunit 3(INTS3) | Hs.438723 |
| SERINC2 | serine incorporator 2(SERINC2) | Hs.270655 |
| CTLA4 | cytotoxic T-lymphocyte associated protein 4(CTLA4) | Hs.247824 |
| FLNC | filamin C(FLNC) | Hs.58414 |
| STEAP1 | STEAP family member 1(STEAP1) | Hs.61635 |
| SLCO1B3 | solute carrier organic anion transporter family member 1B3(SLCO1B3) | Hs.504966 |
| SCARA3 | scavenger receptor class A member 3(SCARA3) | Hs.128856 |
| ZBP1 | Z-DNA binding protein 1(ZBP1) | Hs.302123 |
| CFH | complement factor H(CFH) | Hs.363396 |
| TNFSF13B | tumor necrosis factor superfamily member 13b(TNFSF13B) | Hs.525157 |
| HELB | DNA helicase B(HELB) | Hs.505941 |
| C1ORF106 | chromosome 1 open reading frame 106(C1orf106) | Hs.518997 |
| cyp24a1 | cytochrome P450 family 24 subfamily A member 1(CYP24A1) | Hs.89663 |
| ZNF385D | zinc finger protein 385D(ZNF385D) | Hs.21026 |
| NHEJ1 | non-homologous end joining factor 1(NHEJ1) | Hs.225988 |
| MMP16 | matrix metallopeptidase 16(MMP16) | Hs.492187 |
| ARHGAP29 | Rho GTPase activating protein 29(ARHGAP29) | Hs.483238 |
| UGT1A8 | UDP glucuronosyltransferase family 1 member A8(UGT1A8) | Hs.554822 |
| UGT1A7 | UDP glucuronosyltransferase family 1 member A7(UGT1A7) | Hs.554822 |
| UGT1A6 | UDP glucuronosyltransferase family 1 member A6(UGT1A6) | Hs.554822 |
| ugt1a5 | UDP glucuronosyltransferase family 1 member A5(UGT1A5) | Hs.554822 |
| UGT1A10 | UDP glucuronosyltransferase family 1 member A10(UGT1A10) | Hs.554822 |
| UGT1A9 | UDP glucuronosyltransferase family 1 member A9(UGT1A9) | Hs.554822 |
| UGT1A1 | UDP glucuronosyltransferase family 1 member A1(UGT1A1) | Hs.554822 |
| UGT1A3 | UDP glucuronosyltransferase family 1 member A3(UGT1A3) | Hs.554822 |
| UGT1A4 | UDP glucuronosyltransferase family 1 member A4(UGT1A4) | Hs.554822 |
| CACNA2D3 | calcium voltage-gated channel auxiliary subunit alpha2delta 3(CACNA2D3) | Hs.656687 |
| DPYSL3 | dihydropyrimidinase like 3(DPYSL3) | Hs.519659 |
| STEAP3 | STEAP3 metalloreductase(STEAP3) | Hs.647822 |
| LRIG3 | leucine rich repeats and immunoglobulin like domains 3(LRIG3) | Hs.253736 |
| KCNK3 | potassium two pore domain channel subfamily K member 3(KCNK3) | Hs.645288 |
| vsig10 | V-set and immunoglobulin domain containing 10(VSIG10) | Hs.187624 |
| angptl2 | angiopoietin like 2(ANGPTL2) | Hs.653262 |
| olfml2a | olfactomedin like 2A(OLFML2A) | Hs.357004 |
| KCNN4 | potassium calcium-activated channel subfamily N member 4(KCNN4) | Hs.10082 |
| GPR87 | G protein-coupled receptor 87(GPR87) | Hs.591292 |
| MMP13 | matrix metallopeptidase 13(MMP13) | Hs.2936 |
| SIDT1 | SID1 transmembrane family member 1(SIDT1) | Hs.591291 |
| LCN2 | lipocalin 2(LCN2) | Hs.204238 |
| SEMA5A | semaphorin 5A(SEMA5A) | Hs.27621 |
| SPOCK2 | SPARC/osteonectin, cwcv and kazal like domains proteoglycan 2(SPOCK2) | Hs.523009 |
| MS4A15 | membrane spanning 4-domains A15(MS4A15) | Hs.207465 |
| ZNF436 | zinc finger protein 436(ZNF436) | Hs.293798 |
| FUT1 | fucosyltransferase 1 (H blood group)(FUT1) | Hs.69747 |
| PAM | peptidylglycine alpha-amidating monooxygenase(PAM) | Hs.369430 |
| SLC6A4 | solute carrier family 6 member 4(SLC6A4) | Hs.29792 |
| SHE | Src homology 2 domain containing E(SHE) | Hs.591481 |
| GPD1L | glycerol-3-phosphate dehydrogenase 1-like(GPD1L) | Hs.82432 |
| SESTD1 | SEC14 and spectrin domain containing 1(SESTD1) | Hs.30977 |
| FBLN5 | fibulin 5(FBLN5) | Hs.332708 |
| ASPN | asporin(ASPN) | Hs.435655 |
| AATK | apoptosis associated tyrosine kinase(AATK) | Hs.514575 |
| HSH2D | hematopoietic SH2 domain containing(HSH2D) | Hs.631617 |
| COCH | cochlin(COCH) | Hs.21016 |
| SYTL2 | synaptotagmin like 2(SYTL2) | Hs.369520 |
| PTGES | prostaglandin E synthase(PTGES) | Hs.146688 |
| PAMR1 | peptidase domain containing associated with muscle regeneration 1(PAMR1) | Hs.55044 |
| APOD | apolipoprotein D(APOD) | Hs.522555 |
| BHLHE22 | basic helix-loop-helix family member e22(BHLHE22) | Hs.745052 |
| hirip3 | HIRA interacting protein 3(HIRIP3) | Hs.567370 |
| CD36 | CD36 molecule(CD36) | Hs.120949 |
| CAV1 | caveolin 1(CAV1) | Hs.74034 |
| DOK4 | docking protein 4(DOK4) | Hs.279832 |
| TXNRD1 | thioredoxin reductase 1(TXNRD1) | Hs.654922 |
| CHI3L2 | chitinase 3 like 2(CHI3L2) | Hs.514840 |
| LGI2 | leucine rich repeat LGI family member 2(LGI2) | Hs.12488 |
| f2rl2 | coagulation factor II thrombin receptor like 2(F2RL2) | Hs.42502 |
| MXRA5 | matrix remodeling associated 5(MXRA5) | Hs.369422 |
| LY9 | lymphocyte antigen 9(LY9) | Hs.403857 |
| TEK | TEK receptor tyrosine kinase(TEK) | Hs.89640 |
| IGSF9 | immunoglobulin superfamily member 9(IGSF9) | Hs.591472 |
| POU2AF1 | POU class 2 associating factor 1(POU2AF1) | Hs.654525 |
| ECEL1P2 | endothelin converting enzyme like 1 pseudogene 2(ECEL1P2) | Hs.452575 |
| RXFP1 | relaxin/insulin like family peptide receptor 1(RXFP1) | Hs.591686 |
| TRPV4 | transient receptor potential cation channel subfamily V member 4(TRPV4) | Hs.506713 |
| CDH19 | cadherin 19(CDH19) | Hs.42771 |
| CBLN4 | cerebellin 4 precursor(CBLN4) | Hs.741877 |
| ITLN2 | intelectin 2(ITLN2) | Hs.385631 |
| RAP1GAP | RAP1 GTPase activating protein(RAP1GAP) | Hs.148178 |
| GCNT3 | glucosaminyl (N-acetyl) transferase 3, mucin type(GCNT3) | Hs.194710 |
| VAT1L | vesicle amine transport 1 like(VAT1L) | Hs.461405 |
| DNAJC12 | DnaJ heat shock protein family (Hsp40) member C12(DNAJC12) | Hs.260720 |
| CRTAC1 | cartilage acidic protein 1(CRTAC1) | Hs.500736 |
| HHIP | hedgehog interacting protein(HHIP) | Hs.507991 |
| FAM150A | family with sequence similarity 150 member A(FAM150A) | Hs.527111 |
| c4a | complement C4A (Rodgers blood group)(C4A) | Hs.534847 |
| c4b | complement C4B (Chido blood group)(C4B) | Hs.534847 |
| C4A-AS1 | C4A antisense RNA 1(C4A-AS1) | Hs.534847 |
| IKZF2 | IKAROS family zinc finger 2(IKZF2) | Hs.604950 |
| ASRGL1 | asparaginase like 1(ASRGL1) | Hs.535326 |
| DAPP1 | dual adaptor of phosphotyrosine and 3-phosphoinositides 1(DAPP1) | Hs.708484 |
| C2 | complement C2(C2) | Hs.408903 |
| SYN2 | synapsin II(SYN2) | Hs.445503 |
| CYTL1 | cytokine like 1(CYTL1) | Hs.13872 |
| PCOLCE2 | procollagen C-endopeptidase enhancer 2(PCOLCE2) | Hs.8944 |
| NEDD4L | neural precursor cell expressed, developmentally down-regulated 4-like, E3 ubiquitin protein ligase(NEDD4L) | Hs.185677 |
| LAX1 | lymphocyte transmembrane adaptor 1(LAX1) | Hs.272794 |
| CALCRL | calcitonin receptor like receptor(CALCRL) | Hs.470882 |
| PPFIBP1 | PPFIA binding protein 1(PPFIBP1) | Hs.172445 |
| CCDC80 | coiled-coil domain containing 80(CCDC80) | Hs.477128 |
| EPHB2 | EPH receptor B2(EPHB2) | Hs.380705 |
| CRLF1 | cytokine receptor like factor 1(CRLF1) | Hs.114948 |
| NEBL | nebulette(NEBL) | Hs.5025 |
| GIMAP1-GIMAP5 | GIMAP1-GIMAP5 readthrough(GIMAP1-GIMAP5) | Hs.647079 |
| GIMAP5 | GTPase, IMAP family member 5(GIMAP5) | Hs.647079 |
| GIMAP1 | GTPase, IMAP family member 1(GIMAP1) | Hs.647079 |
| CGN | cingulin(CGN) | Hs.591464 |
| FAM174B | family with sequence similarity 174 member B(FAM174B) | Hs.702316 |
| COX4I2 | cytochrome c oxidase subunit 4I2(COX4I2) | Hs.277101 |
| RNF182 | ring finger protein 182(RNF182) | Hs.111164 |
| SFRP2 | secreted frizzled related protein 2(SFRP2) | Hs.481022 |
| CARHSP1 | calcium regulated heat stable protein 1(CARHSP1) | Hs.632184 |
| TMPRSS4 | transmembrane protease, serine 4(TMPRSS4) | Hs.161985 |
| PDIA4 | protein disulfide isomerase family A member 4(PDIA4) | Hs.93659 |
| STARD13 | StAR related lipid transfer domain containing 13(STARD13) | Hs.721224 |
| IL17RE | interleukin 17 receptor E(IL17RE) | Hs.390823 |
| CAV2 | caveolin 2(CAV2) | Hs.603096 |
| ANKRD36B | ankyrin repeat domain 36B(ANKRD36B) | Hs.532921 |
| SDPR | serum deprivation response(SDPR) | Hs.26530 |
| ACAT1 | acetyl-CoA acetyltransferase 1(ACAT1) | Hs.232375 |
| CFB | complement factor B(CFB) | Hs.69771 |
| FSTL3 | follistatin like 3(FSTL3) | Hs.529038 |
| TP63 | tumor protein p63(TP63) | Hs.137569 |
| OGN | osteoglycin(OGN) | Hs.109439 |
| CADPS | calcium dependent secretion activator(CADPS) | Hs.654933 |
| MET | MET proto-oncogene, receptor tyrosine kinase(MET) | Hs.132966 |
| DES | desmin(DES) | Hs.594952 |
| NECAB1 | N-terminal EF-hand calcium binding protein 1(NECAB1) | Hs.719466 |
| BDNF | brain derived neurotrophic factor(BDNF) | Hs.502182 |
| SLC19A3 | solute carrier family 19 member 3(SLC19A3) | Hs.221597 |
| DIO2 | deiodinase, iodothyronine type II(DIO2) | Hs.708526 |
| SPP1 | secreted phosphoprotein 1(SPP1) | Hs.313 |
| PMM1 | phosphomannomutase 1(PMM1) | Hs.75835 |
| SLC4A11 | solute carrier family 4 member 11(SLC4A11) | Hs.105607 |
| ITGB8 | integrin subunit beta 8(ITGB8) | Hs.592171 |
| RDX | radixin(RDX) | Hs.741211 |
| PCDH1 | protocadherin 1(PCDH1) | Hs.79769 |
| CCNB1IP1 | cyclin B1 interacting protein 1(CCNB1IP1) | Hs.741214 |
| FUT8 | fucosyltransferase 8(FUT8) | Hs.654961 |
| ITM2C | integral membrane protein 2C(ITM2C) | Hs.111577 |
| ST8SIA4 | ST8 alpha-N-acetyl-neuraminide alpha-2,8-sialyltransferase 4(ST8SIA4) | Hs.308628 |
| SEMA3C | semaphorin 3C(SEMA3C) | Hs.269109 |
| CNTN6 | contactin 6(CNTN6) | Hs.387300 |
| CARD11 | caspase recruitment domain family member 11(CARD11) | Hs.648101 |
| DLC1 | DLC1 Rho GTPase activating protein(DLC1) | Hs.738560 |
| fgfr4 | fibroblast growth factor receptor 4(FGFR4) | Hs.165950 |
| GJB2 | gap junction protein beta 2(GJB2) | Hs.714494 |
| TMEM100 | transmembrane protein 100(TMEM100) | Hs.173233 |
| FCRL5 | Fc receptor like 5(FCRL5) | Hs.415950 |
| PTPRB | protein tyrosine phosphatase, receptor type B(PTPRB) | Hs.434375 |
| ANTXR1 | anthrax toxin receptor 1(ANTXR1) | Hs.165859 |
| AGTPBP1 | ATP/GTP binding protein 1(AGTPBP1) | Hs.719980 |
| SUSD4 | sushi domain containing 4(SUSD4) | Hs.497841 |
| SYCP2L | synaptonemal complex protein 2 like(SYCP2L) | Hs.729128 |
| SACS | sacsin molecular chaperone(SACS) | Hs.159492 |
| SCG5 | secretogranin V(SCG5) | Hs.156540 |
| SEL1L3 | SEL1L family member 3(SEL1L3) | Hs.479384 |
| NDRG4 | NDRG family member 4(NDRG4) | Hs.322430 |
| F8 | coagulation factor VIII(F8) | Hs.632836 |
| CXCL6 | C-X-C motif chemokine ligand 6(CXCL6) | Hs.164021 |
| TRIM29 | tripartite motif containing 29(TRIM29) | Hs.504115 |
| DAPK2 | death associated protein kinase 2(DAPK2) | Hs.237886 |
| FNDC4 | fibronectin type III domain containing 4(FNDC4) | Hs.732184 |
| CA4 | carbonic anhydrase 4(CA4) | Hs.89485 |
| PSD3 | pleckstrin and Sec7 domain containing 3(PSD3) | Hs.434255 |
| WFS1 | wolframin ER transmembrane glycoprotein(WFS1) | Hs.518602 |
| CRABP2 | cellular retinoic acid binding protein 2(CRABP2) | Hs.405662 |
| CD1A | CD1a molecule(CD1A) | Hs.1309 |
| CDH13 | cadherin 13(CDH13) | Hs.661776 |
| ARHGEF10 | Rho guanine nucleotide exchange factor 10(ARHGEF10) | Hs.98594 |
| SFRP1 | secreted frizzled related protein 1(SFRP1) | Hs.213424 |
| BAIAP2 | BAI1 associated protein 2(BAIAP2) | Hs.128316 |
| PAX9 | paired box 9(PAX9) | Hs.609574 |
| MEOX1 | mesenchyme homeobox 1(MEOX1) | Hs.438 |
| ARRB1 | arrestin beta 1(ARRB1) | Hs.625320 |
| AOX1 | aldehyde oxidase 1(AOX1) | Hs.406238 |
| RGS2 | regulator of G-protein signaling 2(RGS2) | Hs.78944 |
| HHLA2 | HERV-H LTR-associating 2(HHLA2) | Hs.225968 |
| MS4A1 | membrane spanning 4-domains A1(MS4A1) | Hs.712553 |
| STX3 | syntaxin 3(STX3) | Hs.180711 |
| VSNL1 | visinin like 1(VSNL1) | Hs.444212 |
| ENPP3 | ectonucleotide pyrophosphatase/phosphodiesterase 3(ENPP3) | Hs.486489 |
| GPM6A | glycoprotein M6A(GPM6A) | Hs.75819 |
| COL18A1 | collagen type XVIII alpha 1 chain(COL18A1) | Hs.517356 |
| p2ry1 | purinergic receptor P2Y1(P2RY1) | Hs.79881 |
| ROBO1 | roundabout guidance receptor 1(ROBO1) | Hs.744218 |
| COL10A1 | collagen type X alpha 1 chain(COL10A1) | Hs.520339 |
| ANKS1A | ankyrin repeat and sterile alpha motif domain containing 1A(ANKS1A) | Hs.656492 |
| PDZD2 | PDZ domain containing 2(PDZD2) | Hs.481819 |
| ATP1B1 | ATPase Na+/K+ transporting subunit beta 1(ATP1B1) | Hs.291196 |
| ACTN1 | actinin alpha 1(ACTN1) | Hs.509765 |
| GREM2 | gremlin 2, DAN family BMP antagonist(GREM2) | Hs.98206 |
| INMT | indolethylamine N-methyltransferase(INMT) | Hs.632629 |
| ESR2 | estrogen receptor 2(ESR2) | Hs.734416 |
| DHCR24 | 24-dehydrocholesterol reductase(DHCR24) | Hs.498727 |
| RAB27B | RAB27B, member RAS oncogene family(RAB27B) | Hs.25318 |
| TUFT1 | tuftelin 1(TUFT1) | Hs.489922 |
| SLAMF7 | SLAM family member 7(SLAMF7) | Hs.517265 |
| TNFRSF21 | TNF receptor superfamily member 21(TNFRSF21) | Hs.443577 |
| TSPAN12 | tetraspanin 12(TSPAN12) | Hs.16529 |
| SOX13 | SRY-box 13(SOX13) | Hs.201671 |
| NFATC1 | nuclear factor of activated T-cells 1(NFATC1) | Hs.701518 |
| CPXM2 | carboxypeptidase X, M14 family member 2(CPXM2) | Hs.656887 |
| TNS1 | tensin 1(TNS1) | Hs.471381 |
| THY1 | Thy-1 cell surface antigen(THY1) | Hs.644697 |
| BPIFB1 | BPI fold containing family B member 1(BPIFB1) | Hs.65551 |
| ABHD12B | abhydrolase domain containing 12B(ABHD12B) | Hs.271896 |
| EPHA3 | EPH receptor A3(EPHA3) | Hs.123642 |
| HEY1 | hes related family bHLH transcription factor with YRPW motif 1(HEY1) | Hs.234434 |
| BCL11B | B-cell CLL/lymphoma 11B(BCL11B) | Hs.709690 |
| PDK1 | pyruvate dehydrogenase kinase 1(PDK1) | Hs.470633 |
| EFNB1 | ephrin B1(EFNB1) | Hs.144700 |
| MAGED4B | MAGE family member D4B(MAGED4B) | Hs.571729 |
| Maged4 | MAGE family member D4(MAGED4) | Hs.571729 |
| BOC | BOC cell adhesion associated, oncogene regulated(BOC) | Hs.591318 |
| SLC29A3 | solute carrier family 29 member 3(SLC29A3) | Hs.438419 |
| JPH1 | junctophilin 1(JPH1) | Hs.657367 |
| hivep1 | human immunodeficiency virus type I enhancer binding protein 1(HIVEP1) | Hs.567284 |
| COL5A2 | collagen type V alpha 2 chain(COL5A2) | Hs.445827 |
| slc2a5 | solute carrier family 2 member 5(SLC2A5) | Hs.530003 |
| SLC6A1 | solute carrier family 6 member 1(SLC6A1) | Hs.443874 |
| ADRB2 | adrenoceptor beta 2(ADRB2) | Hs.2551 |
| TNFRSF17 | TNF receptor superfamily member 17(TNFRSF17) | Hs.2556 |
| SCN7A | sodium voltage-gated channel alpha subunit 7(SCN7A) | Hs.596087 |
| FAM167A | family with sequence similarity 167 member A(FAM167A) | Hs.124299 |
| DKK2 | dickkopf WNT signaling pathway inhibitor 2(DKK2) | Hs.211869 |
| CHRDL2 | chordin like 2(CHRDL2) | Hs.432379 |
| SNORA66 | small nucleolar RNA, H/ACA box 66(SNORA66) | Hs.180946 |
| FAM69A | family with sequence similarity 69 member A(FAM69A) | Hs.180946 |
| PLA2G2A | phospholipase A2 group IIA(PLA2G2A) | Hs.466804 |
| PFKP | phosphofructokinase, platelet(PFKP) | Hs.26010 |
| VEGFA | vascular endothelial growth factor A(VEGFA) | Hs.73793 |
| CES1 | carboxylesterase 1(CES1) | Hs.558865 |
| PAPPA | pappalysin 1(PAPPA) | Hs.643599 |
| CNN1 | calponin 1(CNN1) | Hs.465929 |
| IL2RA | interleukin 2 receptor subunit alpha(IL2RA) | Hs.231367 |
| AOC3 | amine oxidase, copper containing 3(AOC3) | Hs.198241 |
| TTYH1 | tweety family member 1(TTYH1) | Hs.268728 |
| CCR7 | C-C motif chemokine receptor 7(CCR7) | Hs.370036 |
| DCLK1 | doublecortin like kinase 1(DCLK1) | Hs.728619 |
| TRIM36 | tripartite motif containing 36(TRIM36) | Hs.519514 |
| LOC728254 | uncharacterized LOC728254(LOC728254) | Hs.519514 |
| DFNA5 | DFNA5, deafness associated tumor suppressor(DFNA5) | Hs.520708 |
| PCTP | phosphatidylcholine transfer protein(PCTP) | Hs.285218 |
| DERL3 | derlin 3(DERL3) | Hs.593679 |
| CNOT8 | CCR4-NOT transcription complex subunit 8(CNOT8) | Hs.26703 |
| BEND7 | BEN domain containing 7(BEND7) | Hs.498740 |
| TMEM106C | transmembrane protein 106C(TMEM106C) | Hs.596726 |
| CGNL1 | cingulin like 1(CGNL1) | Hs.148989 |
| PXMP4 | peroxisomal membrane protein 4(PXMP4) | Hs.654857 |
| SLCO1A2 | solute carrier organic anion transporter family member 1A2(SLCO1A2) | Hs.46440 |
| HELLPAR | HELLP associated long non-coding RNA(HELLPAR) | Hs.160562 |
| IGF1 | insulin like growth factor 1(IGF1) | Hs.160562 |
| SERPIND1 | serpin family D member 1(SERPIND1) | Hs.474270 |
| CRYM | crystallin mu(CRYM) | Hs.924 |
| SLC2A1 | solute carrier family 2 member 1(SLC2A1) | Hs.473721 |
| MX2 | MX dynamin like GTPase 2(MX2) | Hs.926 |
| LOC100131190 | uncharacterized LOC100131190(LOC100131190) | Hs.926 |
| NRGN | neurogranin(NRGN) | Hs.524116 |
| MPP3 | membrane palmitoylated protein 3(MPP3) | Hs.396566 |
| CDCA7 | cell division cycle associated 7(CDCA7) | Hs.470654 |
| ROBO4 | roundabout guidance receptor 4(ROBO4) | Hs.524121 |
| BDKRB2 | bradykinin receptor B2(BDKRB2) | Hs.726620 |
| GPX7 | glutathione peroxidase 7(GPX7) | Hs.43728 |
| DNM2 | dynamin 2(DNM2) | Hs.211463 |
| TMED1 | transmembrane p24 trafficking protein 1(TMED1) | Hs.211463 |
| SYT15 | synaptotagmin 15(SYT15) | Hs.696346 |
| FHL2 | four and a half LIM domains 2(FHL2) | Hs.443687 |
| SPTBN1 | spectrin beta, non-erythrocytic 1(SPTBN1) | Hs.503178 |
| GPR4 | G protein-coupled receptor 4(GPR4) | Hs.17170 |
| PDE7B | phosphodiesterase 7B(PDE7B) | Hs.744230 |
| CTHRC1 | collagen triple helix repeat containing 1(CTHRC1) | Hs.405614 |

**Supplementary table S18.** Pulmonary fibrosis-related canonical pathways of rat lung tissues exposed to PHMG based on IPA analysis

| Predicted confidence | Pathway | Z-score | *P*-value | Refs (PMID No.) |
| --- | --- | --- | --- | --- |
| Activation | p53 signaling | 2.12 | 0.013 | 28722352, 27942595 |
|  | Integrin signaling | 1.94 | 0.018 | 26391549, 28397850, 27535718 |
|  | IL-8 signaling | 1.81 | 0.022 | 28860143, 20952496 |
|  | Leukocyte extravasation signaling | 1.81 | 0.002 | 24972347, 14561169, 9648948 |
|  | HMGB1 signaling | 1.51 | 0.026 | 25284457, 28339089, 26126535 |
|  | TGF-β signaling | 1.13 | 0.013 | 28432134, 27942595 |
|  | Phospholipases | 0.71 | 0.004 | 31090437, 21131602 |
|  |  |  |  |  |
| Inhibition | Relaxin signaling | -2.45 | 0.026 | 21983071, 27250825, 20826567 |
|  | BMP signaling | -1.89 | 0.018 | 31050552, 26585589 |
|  | Coagulation system | -0.82 | 0.002 | 14582899, 23912648 |
|  | STAT3 pathway | -0.71 | 0.039 | 30257954, 30658076, 26324850 |
|  | Endothelin-1 | -0.54 | 0.030 | 19717811, 21057104, 29947539 |
|  | Wnt/β-catenin signaling | -0.30 | 0.003 | 28588349, 30802451, 27112840 |
